# Analysis of lineage-specific protein family variability in prokaryotes combined with evolutionary reconstructions

**DOI:** 10.1186/s13062-022-00337-7

**Published:** 2022-08-30

**Authors:** Svetlana Karamycheva, Yuri I. Wolf, Erez Persi, Eugene V. Koonin, Kira S. Makarova

**Affiliations:** National Center for Biotechnology Information, National Library of Medicine, Bethesda, MD 20894 USA

**Keywords:** Variability, Clusters of orthologous genes, Evolutionary reconstructions, Paralogs

## Abstract

**Background:**

Evolutionary rate is a key characteristic of gene families that is linked to the functional importance of the respective genes as well as specific biological functions of the proteins they encode. Accurate estimation of evolutionary rates is a challenging task that requires precise phylogenetic analysis. Here we present an easy to estimate protein family level measure of sequence variability based on alignment column homogeneity in multiple alignments of protein sequences from Clade-Specific Clusters of Orthologous Genes (csCOGs).

**Results:**

We report genome-wide estimates of variability for 8 diverse groups of bacteria and archaea and investigate the connection between variability and various genomic and biological features. The variability estimates are based on homogeneity distributions across amino acid sequence alignments and can be obtained for multiple groups of genomes at minimal computational expense. About half of the variance in variability values can be explained by the analyzed features, with the greatest contribution coming from the extent of gene paralogy in the given csCOG. The correlation between variability and paralogy appears to originate, primarily, not from gene duplication, but from acquisition of distant paralogs and xenologs, introducing sequence variants that are more divergent than those that could have evolved in situ during the lifetime of the given group of organisms. Both high-variability and low-variability csCOGs were identified in all functional categories, but as expected, proteins encoded by integrated mobile elements as well as proteins involved in defense functions and cell motility are, on average, more variable than proteins with housekeeping functions. Additionally, using linear discriminant analysis, we found that variability and fraction of genomes carrying a given gene are the two variables that provide the best prediction of gene essentiality as compared to the results of transposon mutagenesis in *Sulfolobus islandicus*.

**Conclusions:**

Variability, a measure of sequence diversity within an alignment relative to the overall diversity within a group of organisms, offers a convenient proxy for evolutionary rate estimates and is informative with respect to prediction of functional properties of proteins. In particular, variability is a strong predictor of gene essentiality for the respective organisms and indicative of sub- or neofunctionalization of paralogs.

**Supplementary Information:**

The online version contains supplementary material available at 10.1186/s13062-022-00337-7.

## Background

The determinants of protein evolution rates have been studied for decades, with the rate estimates typically based on evolutionary distances between orthologous proteins in pairs of closely related organisms [1–7]. When functional (transcriptomic and proteomic) data were available, protein abundance or expression level was found to be the strongest correlate for protein conservation, suggesting that the physics of protein folding, and in particular, the probability of misfolding could be among the most important factors limiting protein variability during evolution [8, 9]. On the opposite side of the evolutionary conservation range, very fast sequence divergence was associated with evolution driven by positive selection [10, 11], often limited to specific regions or sites within proteins or acting for relatively short periods of time [12–14].

High protein variability and evolutionary fluidity appear to be often associated with the protein’s role in various biological conflict scenarios [15, 16], sometimes serving as a hallmark for the discovery of novel defense and offence systems in prokaryotes [17, 18]. Discovery of multiple diversity-generating mechanisms [19–21], which target gene regions that need to adapt particularly rapidly, underscore the importance of this phenomenon.

Quantification of sequence variability is a non-trivial task. Measures based on the distribution of amino acids in alignments (from the number of different characters to the Shannon entropy of an alignment column) do not take into account amino acid properties, effectively assigning the same weight to all mismatches. Measures based on explicit evolutionary reconstructions (tree distances and numbers of mutational events) are highly computationally expensive and require a careful choice of the evolutionary model [22–25]. Previously, we described a site homogeneity measure [26] that provides a compromise, taking into account an amino acid similarity matrix and using sequence weights to mitigate the effect of uneven distribution of sequences across the range of phylogenetic distances.

Evolutionary distances themselves or homogeneity, used as their proxy, estimate sequence conservation in absolute terms. If different alignments need to be compared to each other, it is important to keep the context as uniform as possible (i.e., using alignments representing comparable evolutionary depth) or to find a way to take the context into account explicitly. Here we suggest a measure of protein variability in the context of alignments of clade-specific orthologs and survey the distribution of the estimated variability in several selected lineages of archaea and bacteria. We explore the genomic features associated with protein variability and investigate gene families with unusual patterns of sequence variation.

## Results

### Estimation of gene variability

**Fig. 1:**
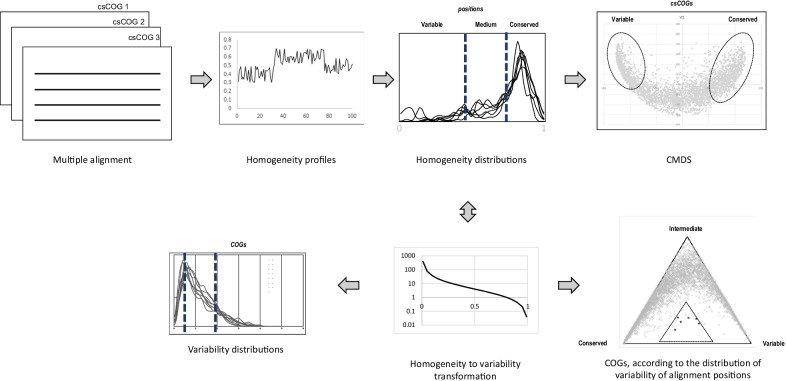
Pipeline for protein variability analysis. Homogeneity values are calculated for each position of multiple alignments of clade-specific COG (csCOG) sequences (top left). Homogeneity profiles along the sequences are smoothed and converted to distributions of the homogeneity values (top middle). Distances between the homogeneity value distributions are used to embed csCOGs into a metric space (top right). Homogeneity values, scaled by the average homogeneity across the clade, are transformed into variabilities (bottom middle). csCOG-specific values form clade-level distributions (bottom left). Position-specific variability values allow to categorize alignment sites into conserved, intermediate, and variable; relative frequency of these classes, plotted on a simplex diagram, identifies csCOG with unusual conservation patterns (bottom right)

**Fig. 2:**
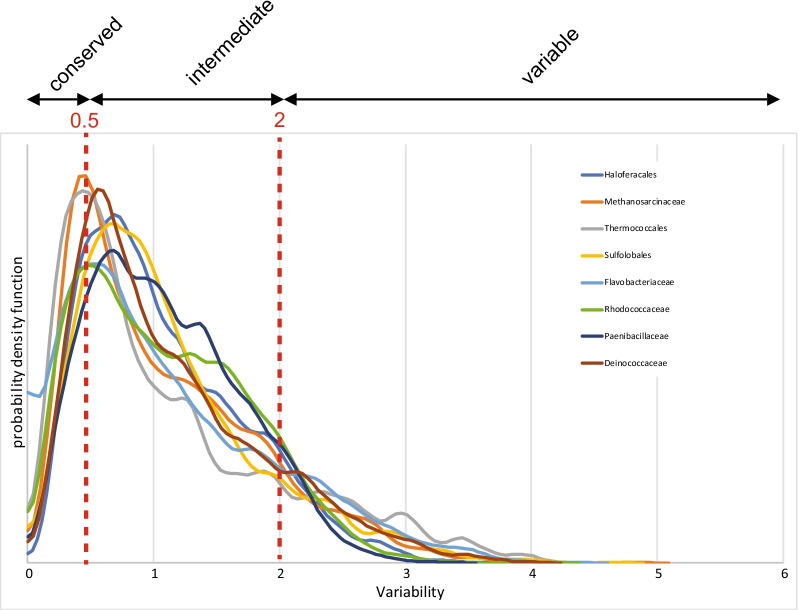
Distribution of variability values across clade-specific COGs. Gaussian kernel-smoothed probability density functions for variability values in clade-specific pangenomes (plots for eight clades are shown). Threshold values for conserved (variability *v* < 0.5), intermediate (0.5 < *v* < 2), and variable (*v* > 2) csCOGs are indicated

We selected 8 taxonomically diverse lineages of archaea and bacteria at genus or family level, with 30–60 genomes in each. Namely, archaea: Haloferacales, Sulfolobales, Thermococcales, and Methanosarcinales; bacteria: Flavobacteriales, Deinococcales, Paenibacillus, and Rhodococcus. The majority of these lineages include at least one representative amenable to genetic manipulation [27–31], facilitating future validation of functional predictions. For each of these sets of genomes we built clade-specific csCOGs (see Methods for details). Phyletic patterns of these csCOGs, along with the genome tree, were then used to reconstruct the history of gene gains and losses for each csCOG. Multiple protein sequence alignments of all csCOGs were constructed; for alignments containing 8 or more non-identical sequences, the homogeneity values were calculated for each alignment column. This data was used to obtain csCOG- and position-specific variability estimates that relate the csCOG-specific or position-specific homogeneity to the mean across the clade (Fig. 1, Additional file 1, see Methods for details). The distributions of the variability values for all 8 lineages were closely similar (Fig. 2), suggesting that these values indeed are comparable across lineages. csCOGs (or individual alignment positions) with the relative variability $v<0.5$ were classified as conserved, and those with relative variability $v>2$ were classified as variable.

### Gene features defining variability

**Fig. 3:**
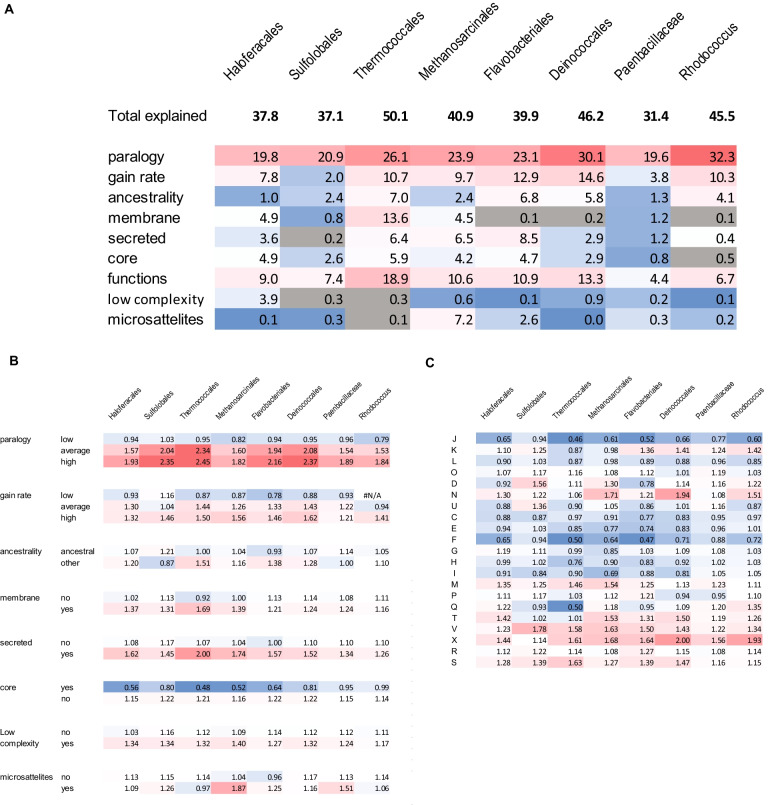
Association of protein variability with other genomic and biological features. **A** Fraction (in percent) of variance of protein variability explained by other properties. The “total explained” fraction is estimated using multivariable regression. The fraction explained by individual properties is estimated using ANOVA. The cells, corresponding to properties, excluded by Akaike criterion based stepwise reduction of multivariable regression model, are shaded in gray. **B** Average variability of subsets of genes categorized by other properties. **C** Average variability of subsets of genes categorized by COG functional categories. Functional categories are the following: J—Translation, ribosomal structure and biogenesis; K—Transcription; L—Replication, recombination and repair; D—Cell cycle control, cell division, chromosome partitioning; V—Defense mechanisms; T—Signal transduction mechanisms; M—Cell wall/membrane/envelope biogenesis; N—Cell motility; W—Extracellular structures; O—Posttranslational modification, protein turnover, chaperones; X—Mobilome: prophages, transposons; C—Energy production and conversion; G—Carbohydrate transport and metabolism; E—Amino acid transport and metabolism; F—Nucleotide transport and metabolism; H—Coenzyme transport and metabolism; I—Lipid transport and metabolism; P—Inorganic ion transport and metabolism; Q—Secondary metabolites biosynthesis, transport and catabolism; R—General function prediction only; S—Function unknown; Color scale from blue to red is proportional to the value

The first question we addressed was to what extent csCOG variability could be explained by a combination of features that are expected to affect or correlate with protein evolution rate. For this analysis, the following features were chosen: membership in bacterial (320 prokaryotic COGs) or archaeal core (218 arCOGs [32]); inferred number of gains in the history of the csCOG; number of paralogs in the csCOG, ancestrality of the csCOG relative to the clade (categorized as ancestral, intermediate and terminal branch acquisition), presence of transmembrane segments (categorized as present if predicted for at least 1/3 of proteins in a csCOG), presence of signal peptide (categorized as present if predicted for at least 1/3 of proteins in a csCOG), fraction of low complexity regions (average across the csCOG members), fraction of microsatellite-like repeats in the respective genes (average across the csCOG members) and functional classification into one of the 21 COG functional groups (Additional file 1: Table S1). Only up to 50% of the variance in the csCOG variability estimates could be explained from all these features combined (Fig. 3A). Next, we examined the correlations between variability and each individual feature. The number of paralogs showed the strongest correlation, explaining from 19 to 30% of the variability values variance, followed by gene gain rate and functional classification (Fig. 3A). Surprisingly, average low complexity masking fraction and microsatellite-like repeats fraction only weakly correlated with variability, comparable with the weak correlation observed for membrane proteins (Fig. 3A).

Although this trend is common for most lineages, the strength of association with variability for some of the features varied substantially. For example, presence of a transmembrane segment explained less than 1% of variability value variance in Flavobacteriales and Deinococcales, but 14% in Thermococcales, and gene gain rate explained 2% of the variance in Sulfolobales but 15% in Deinococcaceae; there were more examples of contrasting associations like this (Fig. 3A).

To gain additional information on differences in variability with respect to the above features of protein families, we analyzed distinct subsets of csCOGs, grouped by each feature separately. To this end, we computed mean variability for each subset and estimated the statistical significance of the differences of variability between the analyzed subsets for each value using ANOVA (Fig. 3B). All the differences were significant (p value < 0.01). Specifically, paralogy numbers were separated into three bins (1–1.25—low; 1.25–3—medium, > 3—high). As expected, mean variability increased with the increase of the number of paralogs in almost all lineages. In 4 lineages, the high-paralogy subset of the csCOGs showed mean variability twofold higher than the clade-specific average. The same trend was observed for three bins of gene gain rate (0–0.5—low; 0.5–2—medium and > 2—high) and ancestrality measure (ancestral vs all other) although the association with variability was weaker for most of the lineages compared with that for the number of paralogs. The association with variability was comparable and weak for secreted and membrane proteins, consistent with previous observations indicating that membrane and surface proteins generally evolve faster than soluble ones [33]. Perhaps surprisingly, archaea have slightly more variable secreted and membrane proteins than bacteria. As expected [8], core genes are significantly less variable than non-core ones and, as a group, show the lowest mean variability among all analyzed cohorts.

We next analyzed mean variability for the 21 functional categories of genes assigned by comparing the csCOGs to prokaryotic COGs and, for archaea, to arCOGs (Fig. 3C). The resulting estimates were qualitatively closely similar to those obtained by analysis of genome flux data for more closely related subsets of bacterial and archaeal genomes [34]. Specifically, the categories X (mobilome), V (defense and offense systems), M (cell wall/membrane/envelope biogenesis) and N (cell motility) tend to have higher mean variability values, whereas categories J (translation, ribosomal structure and biogenesis) and F (nucleotide transport and metabolism) have the lowest values (Fig. 3C).

**Fig. 4:**
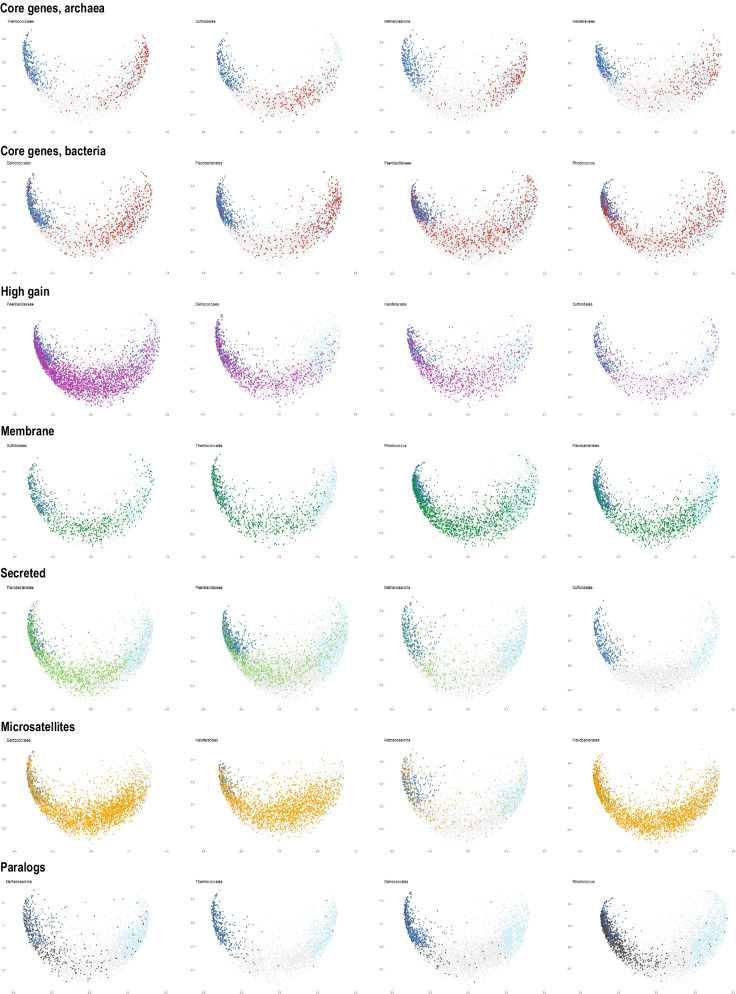
Multidimensional scaling analysis of variability values and selected features. Homogeneity distribution density was calculated for each csCOG as described in Material and Methods. Classical multidimensional scaling (*cmdscale* function in R) was applied to visualize the relationship between csCOGs. Hellinger distance (one of the conceptually simplest distance measures which is also symmetrical and metric) was used to quantify the similarity between each two probability distributions. Results for the first two dimensions were used to construct plots. Variability of the data points are shown as follows: Conserved (0–0.5): light blue; medium (0.5–2.0): light gray; variable (> 2.0)” dark blue. The following features are overlayed onto points: presence in the set of core genes—red dots; high gain rate (> 2.5)—magenta dots; membrane (csCOGs with the average fraction of proteins with predicted transmembrane segments > 0.333)—dark green dots; secreted (csCOGs with the average fraction of proteins with signal peptide > 0.333), microsatellite like regions (the average fraction of protein sequences in the csCOG identified >= 0.15)—orange dots; high paralogy (> 2.0)—dark gray dots

Despite some strong associations described above, each feature showed high dispersion of variability values (Fig. 4). Among core and ancestral families, there are highly variable ones, and conversely, there are conserved membrane proteins, secreted proteins and proteins with large fraction of low complexity or microsattelite-like regions (Additional file 1).

### Protein families enriched in variable csCOGs

**Fig. 5:**
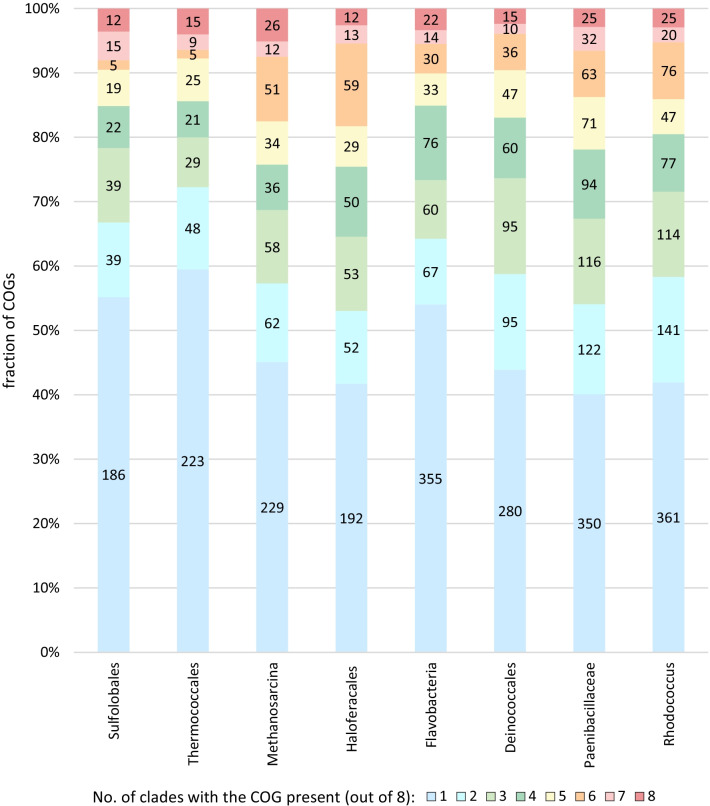
Breakdown of high variability protein families by presence in 1–8 other analyzed lineages. Numbers on the plot indicate the actual number of csCOGs with high variability (> 2.0) that are present in the given number of genomes; the plots for each family are scaled to 100%

Based on assignments of variable csCOGs to prokaryotic COG families, we estimated abundance of the COG families in the csCOGs (Fig. 5). About 40 to 60% of the variable csCOGs were found to be unique to the respective lineage, whereas the remaining ones were assigned to prokaryotic COGs that are represented in at least one other bacterial or archaeal lineage, including conserved COGs those that are present in 7 or even all 8 groups analyzed here (Table 1). As expected, csCOGs assigned to these families typically have many paralogs and a high gain rate (Additional file 1). Furthermore, we also observed substantial variation of the variability estimates for csCOGs that are assigned to the same prokaryotic COG. Many of such paralogs are not variable, but moderately or even strongly conserved (Table 1, Additional file 1), suggesting functional diversification and/or structural flexibility within the variable csCOGs. Indeed, many of these csCOGs consist of enzymes with diverse and broad specificities, such as RimI-like N-acetyltransferases, COG0456 [35] and class I UbiE/MenG-like methyltransferases, COG2226 [36, 37]. Some of these diverse functions are associated with small molecule modification pathways that are involved in xenobiotic detoxification, production of virulence factors or toxins, or other defense and offence mechanisms, functions that are enriched among variable families (see above).

**Table 1:** COGs that are among hypervariable families among both bacteria and archaea

COG number	Function	Gene name	Description	Number of csCOGs*							
				Deinococcus-V1	Deinococcus-V2	Flavobacteriales—V1	Flavobacteriales—V2	Haloferacales—V1	Haloferacales—V2	Methanosarcinales—V1	Methanosarcinales—V2
COG0438	M	RfaB	Glycosyltransferase involved in cell wall biosynthesis	6	5	14	10	8	4	4	10
COG0456	J	RimI	Ribosomal protein S18 acetylase RimI and related acetyltransferases	10	3	7	7	15	2	5	3
COG0463	M	WcaA	Glycosyltransferase involved in cell wall biosynthesis	2	1	12	5	8	2	5	3
COG0531	E	PotE	Serine transporter YbeC, amino acid:H + symporter family	0	0	4	1	1	1	5	1
COG0671	I	PgpB	Membrane-associated phospholipid phosphatase	2	1	3	1	1	1	0	1
COG0747	E	DdpA	ABC-type transport system, periplasmic component	9	1	1	0	3	3	1	1
COG0842	V	YadH	ABC-type multidrug transport system, permease component	2	0	4	2	1	1	2	2
COG1131	V	CcmA	ABC-type multidrug transport system, ATPase component	5	1	8	1	8	1	3	2
COG1216	G	WcaE	Glycosyltransferase, GT2 family	0	1	6	1	0	2	0	1
COG1846	K	MarR	DNA-binding transcriptional regulator, MarR family	9	3	3	1	6	2	7	1
COG2226	H	UbiE	Ubiquinone/menaquinone biosynthesis C-methylase UbiE/MenG	8	3	3	2	16	2	14	7
COG2244	M	RfbX	Membrane protein involved in the export of O-antigen and teichoic acid	1	2	4	2	0	2	6	4
COG2814	G	AraJ	Predicted arabinose efflux permease AraJ, MFS family	23	4	3	3	18	2	6	2

COG number	Function	Gene name	Description	Number of csCOGs*							
				Paenbacillus—V1	Paenbacillus—V2	Rhodococcus—V1	Rhodococcus—V2	Sulfolobales—V1	Sulfolobales—V2	Thermococcales—V1	Thermococcales—V2
COG0438	M	RfaB	Glycosyltransferase involved in cell wall biosynthesis	23	6	7	4	11	3	3	5
COG0456	J	RimI	Ribosomal protein S18 acetylase RimI and related acetyltransferases	31	13	9	2	2	1	7	0
COG0463	M	WcaA	Glycosyltransferase involved in cell wall biosynthesis	16	4	3	3	2	4	7	2
COG0531	E	PotE	Serine transporter YbeC, amino acid:H + symporter family	9	4	4	2	17	3	0	1
COG0671	I	PgpB	Membrane-associated phospholipid phosphatase	6	1	3	1	1	0	2	1
COG0747	E	DdpA	ABC-type transport system, periplasmic component	4	2	2	4	2	2	2	1
COG0842	V	YadH	ABC-type multidrug transport system, permease component	8	2	6	2	7	2	5	1
COG1131	V	CcmA	ABC-type multidrug transport system, ATPase component	24	5	15	0	11	2	6	3
COG1216	G	WcaE	Glycosyltransferase, GT2 family	7	0	8	1	1	2	1	2
COG1846	K	MarR	DNA-binding transcriptional regulator, MarR family	23	5	16	8	4	3	2	0
COG2226	H	UbiE	Ubiquinone/menaquinone biosynthesis C-methylase UbiE/MenG	20	6	13	5	7	1	2	4
COG2244	M	RfbX	Membrane protein involved in the export of O-antigen and teichoic acid	7	2	2	3	4	1	2	3
COG2814	G	AraJ	Predicted arabinose efflux permease AraJ, MFS family	38	7	46	10	10	3	13	1

^*^V1—low and medium variability csCOGs; V2—high variability csCOGs; Haloferacales family, Sulfolobales family, Thermococcales family, Methanosarcinales family and bacteria—Flavobacteriales family, Deinococcus genus, Paenbacillus genus, Rhodococcus genus

**Fig. 6:**
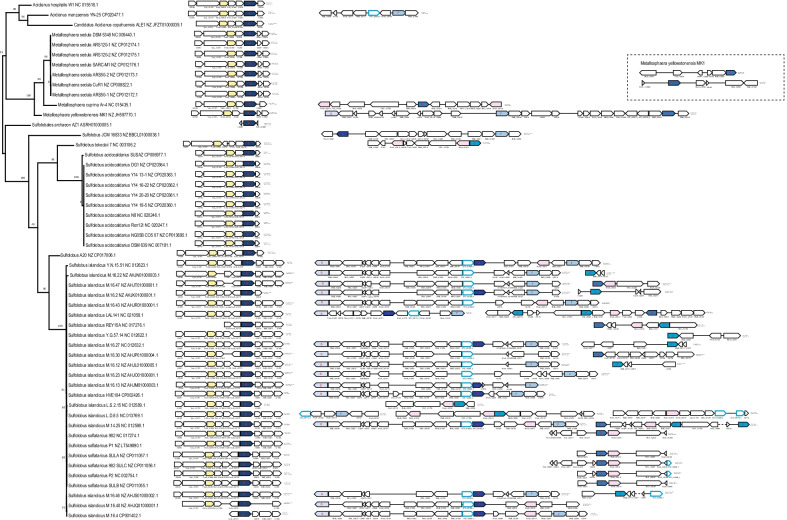
Evolutionary history of sulfo9.00007 family of WcaE-like glycosyltransferases. The neighborhood of all genes from sulfo9.00007 are mapped to 16S rRNA tree of Sulfolobales genomes analyzes in this work. For each gene neighborhood, the genbank accession and coordinates of the locus are indicated on the right. Genes are shown by block arrows, roughly to scale. csCOG number is indicated for all genes and follow gene name (if available). For the genes that are in respective arCOGs the cluster number corresponds to the respective arCOG number. Memebers of sulfo9.00007 are colored by blue shades according to phylogenetic analysis of WcaE-like glycosyltransferases (clades A-E, Additional file 3: Fig. S2). Other glycosyltransferases assigned to COG1216, but not to sulfo9.00007 are shown by blue outline. Closest most frequent gene neighbors are shown by yellow (FabG) and pink (WsaA)

Three of these variable prokaryotic COGs belong to distinct families of glycosytransferases, WcaA, WcaE, (GT-A fold, GT-2 family, COG0463 and COG1216 respectively) and RfaB (GT-B fold, GT-1 family, COG0438) (Table 1). These enzymes are among the most diverse in prokaryotes and catalyze transfer of various sugar moieties from activated donors to acceptor molecules, forming glycoside bonds [36, 37]. Glycosytransferases are typically associated with other genes encoding enzymes involved in cell wall biosynthesis and surface proteins glycosylation. csCOGs that are assigned to these glycosytransferase families also typically include many paralogs and have a high gain rate although some of them appear to be ancestral (Additional file 1). To explore the potential causes of variability in these families, we selected the csCOG sulfo9.00007 from the Sulfolobales group, which is present in all 52 genomes of this group and consists of 127 proteins (2.4 paralogs per genome on average, variability of 4.9). For all genes in this csCOG, we analyzed the genomic context and performed phylogenetic analysis that also included members of the prokaryotic COG1216 (see Material and Methods for details). Phylogenetic analysis showed that proteins from sulfo9.00007 belonged to at least 6 distinct clades (A-F), but because none of these genes is present in all genomes, they formed a “para-COG”, with many proteins of different origins ending up in their respective genomes as a result of multiple events of gene displacement by distant homologs (xenologs) (Fig. 6 and Additional file 2: Fig. S1). For example, Sulfolobus JCM 16833 lost the clade E gene, but possesses A and F clade genes instead. Generally, genes of all clades except clade E show an extremely patchy distribution in the Sulfolobales genomes and are encoded in different neighborhoods suggesting that most loci encoding sulfo9.00007 genes are hot spots of gene shuffling and recombination. It appears that horizontal gene transfer, and in particular, xenologous gene displacement, is also responsible for the high variability of other csCOGs with multiple paralogs that are predicted to be ancestral based on their phyletic pattern. Functionally, this frequent gene exchange might be relevant for changing the glycosylation pattern of surface proteins to avoid virus attachment as well as other variations related to biological conflicts.

### Identification of variable regions in multiple alignments

All estimates described above were based on average variability values calculated for the complete multiple alignment of each csCOG. It is expected, however, that in some proteins, different regions or domains evolve at substantially different rates. To visualize the fraction of positions in multiple alignments with different variability values, we plotted the fractions of conserved, medium and variable positions for each csCOG (Additional file 3: Fig. S2). These plots reveal csCOGs with the unusual prevalence of highly conserved and highly variable positions, but with relatively scarce medium variable positions. We analyzed in detail multiple alignments of several of these csCOGs, focusing on those that are ancestral with few paralogs (Table 2). There are only a few such csCOGs in most of the studied groups of organisms, and Sulfolobales have none. These csCOGs differed among lineages, the only exception being MutL which made the list in both Halobacteria and Paenibacillus. Most of the respective csCOG are ancestral, and many have important and even essential house-keeping functions (Table 2, Additional file 1). Visual examination of the identified variable regions showed that many of them contained variable-length runs of the same amino acid or short repeats and multiple insertions-deletions (Additional file 4: Fig. S3). In order to characterize these regions in greater detail, we performed additional analyses focusing on 34 ancestral families, in which the region of variability was maintained throughout the evolution of an entire lineage. First, we checked whether the respective genes contained an increased fraction of microsatellite-like regions, which might be responsible for polymerase slippage and tandem repeat genome instability [38]. The results of this analysis demonstrate considerable heterogeneity of the average fraction of such regions in these proteins, ranging from none to one third, with the average of 8% across the 34 csCOGs (Additional file 1). Such variation implies that different processes likely contribute to the high variability of these regions. Principal Component Analysis of amino acid frequencies in variable and conserved positions of these csCOGs showed that variable regions are enriched in proline, serine, threonine, aspartate and glutamate (Additional file 5: Fig. S4), that is amino acids with a low propensity for secondary structure formation, suggesting that these regions are unstructured. Indeed, using IUpred [39], all variable regions were predicted to be structurally disordered (Additional file 4: Fig. S3). Function of any of these disordered regions is not known. As could be expected, in protein structures that were available for members of 14 csCOGs in this set, the variable regions either formed insertions or terminal regions that were either unresolved/disordered (as in RpoD), or the structure was solved for separate domains of the protein containing a variable region (for example, MutL) that are connected by a supposedly disordered variable linker, or the structure was solved for homologs that lacked the variable region (for example, Rho and FtsY).

**Table 2:** Protein families with high fraction of conserved and variable positions

csCOG identifier	COG	Func	Gene	Description	Comment
flavo9.00376	COG1158	K	Rho	Transcription termination factor Rho	Mostly Bacteroidetes
flavo9.00582	COG1314	U	SecG	Protein translocase subunit SecG	All bacteroidetes, but also in some other bacteria such as Chlorobia, some Proteobacteria, Spirochaetes; others do not possess the variable tail
flavo9.00756	–	–	–	–	xre family HTH (N-terminal), the loop is present mostly in Bacteroidetes, but seen in some Bacilli too
flavo9.00944	COG4807	S	YehS	Uncharacterized conserved protein YehS, DUF1456 family	Specific for Flavobacterium
deino9.00350	–	–	–	–	An artefact: wrong ORFs start in some of these genes
deino9.00475	COG1722	L	XseB	Exonuclease VII small subunit	Variable tail in other bacteria too
deino9.00842	COG0511	I	AccB	Biotin carboxyl carrier protein	PA-rich, present in most bacteria
deino9.01337	–	–	–	–	Uncharacterized, small, Deinococcus specific
deino9.01490	COG0568	K	RpoD	DNA-directed RNA polymerase, sigma subunit (sigma70/sigma32)	Specific N-terminal extension in Deinococci and Truepera, although partially low complexity region is present in Thermus
deino9.03407	COG0199	J	RpsN	Ribosomal protein S14	Xenologous gene displacement by zinc finger variant in some Deinococci
paen9.00611	COG1937	K	FrmR	DNA-binding transcriptional regulator, FrmR family	Copper-sensitive operon repressor, variable N-terminal region is present in many other Firmicutes
paen9.00802	–	–	–	YycC-like protein, PF14174.7	Paenibacillus specific variable tail
paen9.00805	COG3874	S	YtfJ	Uncharacterized spore protein YtfJ	Sporulation protein YtfJ; variable region is present in many sporulating Bacilli, but variable tail is rather specific for Paenibacillus
paen9.00958	COG1674	D	FtsK	DNA segregation ATPase FtsK/SpoIIIE or related protein	Variable insertion is present in all Bacilli and other bacteria, in Paenibacillus these regions are longer
paen9.01226	COG0323	L	MutL	DNA mismatch repair ATPase MutL	Common feature among some archaea and some bacteria
paen9.01699	COG4467	L	YabA	Regulator of replication initiation timing YabA	Variable insertion is present in all Firmicutes and other bacteria, in Paenibacillus these regions is longer [66]
paen9.02368	COG0532	J	InfB	Translation initiation factor IF-2, a GTPase	Variable insertion is present in all Firmicutes (very different lengths), in Paenibacillus these regions are longer, but not the longest among Firmicutes. In many other bacteria the insertion is much smaller [67]
rhodo7.000637	COG1826	U	TatA	Twin-arginine protein secretion pathway components TatA and TatB	Variable tail is specific for at least actinobacteria
rhodo7.001015	COG5416	S	YrvD	Uncharacterized integral membrane protein YrvD	Variable N-terminal region specific for actinobacteria, but not others
rhodo7.001149	COG2409	S	YdfJ	Predicted lipid transporter YdfJ, MMPL/SSD domain, RND superfamily	Variable tail region specific for actinobacteria, but not others, sometime the tail is missing in actinobacteria too
rhodo7.001169	–	–	–	lipid droplet-associated protein	Found in lipid droplets in *Mycobacterium tuberculosis* [68]; two variable internal regions specific for actinobacteria
rhodo7.001269	COG1158	K	Rho	Transcription termination factor Rho	N-terminal variable region specific for actinobacteria
rhodo7.001344	COG0328	L	RnhA	Ribonuclease HI	Variable region is present in many bacteria
rhodo7.001562	COG1862	U	YajC	Protein translocase subunit YajC	Variable region is present in many bacteria
rhodo7.001949	COG0305	L	DnaB	Replicative DNA helicase	Some contain intein
thermo9.00277	(arCOG04026)	–	–	Pilin/Flagellin, contains class III signal peptide	Thermococcus specific, not present elsewhere
halo9.00332	COG0323	L	MutL	DNA mismatch repair enzyme (predicted ATPase)	Common feature among some archaea and some bacteria
halo9.00351	COG1885	S	–	Uncharacterized protein, DUF555 family	Uncharacterized, variable tail present in Methanosarcina, but not in a few other euryarchaea
halo9.00421	COG4530	S	–	Uncharacterized protein	Uncharacterized DUF5806, specific for Halobacteria variable N-terminal region, some have CxxCxHxxH motif, variable N-terminal region
halo9.00587	COG0805	U	–	Sec-independent protein translocase protein TatC	Specific for Halobacteria variable N-terminal region
halo9.00602	COG0552	U	–	Signal recognition particle-docking protein FtsY	N-terminal variable region present in many euryarchaea
halo9.00879	COG1474	L	–	orc1/cdc6 family replication initiation protein	N-terminal region specific for Haloferacales
halo9.00317	COG0358	L	DnaG	DNA primase (bacterial type)	Common feature among euryarchaea
methano7.000496	COG1311	L	HYS2	Archaeal DNA polymerase II, small subunit/DNA polymerase delta, subunit B	Specific for Methanosarcina

We further sought to determine whether the variable protein regions were specific to the respective lineages or originated earlier during evolution. To this end, we ran PSI-BLAST against a collection of prokaryotic genomes and visually examined the outputs. In 19 of the 34 analyzed csCOGs, the variable regions originated prior to the appearance of the respective lineage whereas in the remaining 12 cases, these regions seemed to be lineage-specific (Table 2). In many families, the variable regions were found to be absent in orthologous proteins from other lineages. Examples include MutL (no variable region in Deinococcus/Thermus bacteria), SecG (no variable tail in Deinococcus/Thermus and Firmicutes bacteria), Rho and FtsY (no variable region in Proteobacteria) and more (Additional file 4: Fig. S3). These observations indicate that variable regions appear in different lineages of prokaryotes and persist in these for considerable evolutionary time but are dispensable in other lineages. In three cases, however, the observed variability of protein regions appears to be due to other causes (Table 2). In one case, erroneous prediction of the ORF start resulted in caused an artifactual high variability value (deino9.00350); another variable region is located in the region of intein insertion in several DnaB proteins from Haloferacales; and finally, in one case (Ribosomal protein S14 in *Deinococcus*), the variable region apparently resulted from xenologous gene displacement (Table 2).

### Variability and protein function

High protein variability poorly correlates with csCOGs functional categories, which makes it a weak predictor of protein function although variability can be considered an additional, indirect indication, along with other lines of evidence, such as suggestive genomic context, for such functions as mobilome (X), defense (V) and cell motility (N) (Fig. 3). However, both high and low variability assignments can be helpful in functional analysis of ancestral protein families. Specifically, high variability might indicate subfunctionalization or neofunctionalization of a paralog. For example, among variable proteins in Sulfolobales, there is a tRNA splicing endonuclease SEN2 (sulfo9.01015), which is present in 51 of the 52 genomes in this lineage and belongs to COG1676. This variable protein has a slowly evolving paralog (sulfo9.00331), which is encoded in all these genomes (Additional file 1). The proteins have been studied experimentally, and it has been shown that tRNA splicing endonuclease SEN2 in Sulfolobus is a heterodimer, in which one subunit is inactivated and poorly conserved, and that both are required for the enzyme function, a characteristic case of subfunctionalization [40]. Four paralogs of CdvB/ESCRTIII family (Additional file 6: Table S2) in Sulfolobales are another example of potential subfunctionalization. All these proteins can form filaments [41] but only two (sulfo9.01480 and sulfo9.00714) are essential in *Sulfolobus islandicus* [42], and only one of these has been experimentally shown to be recruited by CdvA [43]. Thus, the actual function of three of the four paralogs remains unclear. Other prominent examples for both archaea and bacteria are listed in Additional file 6: Table S2, and their functional specialization could be of interest for future experimental studies.

Low variability, along with the presence of a gene in a high fraction of genomes in a large group of organisms, appears to be an important indicator of gene essentiality. Based on the observations above and previous analyses [44], xenologous gene displacement and acquisition of additional paralogs substantially contribute to the observed variability of protein families. Slowly evolving genes are expected to be least prone to displacement by genes from distant species. Indeed, using linear discriminant analysis, we found that variability and fraction of genomes carrying the gene are the two variables that provide the best prediction of essentiality based on the transposon mutagenesis in *S. islandicus* [42], with the peak performance of 66.8% true predictions (Additional file 7: Fig. S5). Thus, using these variables, it is possible to identify numerous uncharacterized protein families that are expected to be important and possibly essential for the respective organisms. Table 3 lists 5 such families for each lineage. Two of these families in Sulfolobales were indeed found to be essential in *S. islandicus* [42]. Furthermore, csCOGs corresponding to two uncharacterized families (DUF424 and DUF555 in the PFAM database) were independently identified as low variability in Thermococcales and Methanosarcinales. According to the arCOG database, DUF424 (arCOG04051) is present in the majority of archaea and DUF555 (arCOG02119) is found in most euryarchaea, which is compatible with the essentiality of these genes. In Methanosarcinales, two families DUF2112 and DUF2102, annotated as methanogenesis markers 5 and 6, respectively, form a conserved operon, which is highly specific to methanogenic archaea. Other uncharacterized families (DUFs) conforming to these two criteria were also identified in bacteria (Table 3). Additionally, several csCOGs were conserved in a narrower group of organisms and are not assigned to any family in the current CDD database (Table 3). These include deino9.00587, deino9.00288 and deino9.01656, which are also shared with Thermus, and are likely to be important in most bacteria of the Thermus/Deinococcus lineage. Notably, none of the proteins that considered to be determinants of radiation resistance specific for the Deinococcus genus, were in this list [45–47]. Four families that are implicated in radiation resistance and satisfy the two criteria of essentiality are RecA recombinase, Holliday junction resolvasome helicase RuvB, radiation response regulon transcription factor DdrO, Excinuclease ATPase subunit UvrA, RecO and RecF recombination proteins are all common in other bacteria (Additional file 1) [48]. Thus, Deinococcus-specific protein families contributing to radiation resistance could be dispensable under standard growth conditions, which is indeed the case for several of these families where experimental data is available [49, 50], and additionally, most of these genes are not found in all Deinococcus species (Additional file 1).

**Table 3:** Selected functionally uncharacterized protein families with low variability and presence in 85% or more genomes in respective lineage

csCOG	Genome number	Proteins number	Varia-bility	COG (arCOG)*	Pfam (DUF)	Comment
sulfo9.02117	52	52	0.25	COG1698 (arCOG04308)		Essential [42]; PDB: 2QZG, linked to Zn-finger protein
sulfo9.02278	52	52	0.26	(arCOG08212)		
sulfo9.01977	52	52	0.29	(arCOG05886)		Essential [42]
sulfo9.00722	52	52	0.57	COG1888 (arCOG04140)		PDB: 3BPD; ferredoxin fold
sulfo9.01763	52	52	0.61	COG4755 (arCOG04123)	DUF2153	Linked to Trm112 RNA methyltransferase activating protein
halo9.02555	37	40	0.36	COG1885 (arCOG02119)	DUF555	Single CxxC, weak similarity to CREN7
halo9.01859	37	37	0.38	(arCOG04616)	DUF5800	
halo9.01783	37	37	0.39	(arCOG04777)		
halo9.02264	36	36	0.28	(arCOG04587)		Linked to glutaredoxin family protein
halo9.02689	32	32	0.23	(arCOG03655)		Linked to Anion-transporting ATPase ArsA
halo9.02039	37	37	0.49	COG2412 (arCOG04051)	DUF424	PDB: 2QYA; linked to TPR repeats containing protein
thermo9.00526	40	40	0.46	(arCOG04849)		Linked to Ribosome biogenesis GTPase A
thermo9.01167	41	41	0.3	COG2412 (arCOG04051)	DUF424	linked to NMD protein affecting ribosome stability and mRNA decay
thermo9.01884	41	41	0.32	(arCOG05846)		Linked to Transcription initiation factor IIE, alpha subunit
thermo9.01623	41	41	0.36	COG1885 (arCOG02119)	DUF555	Linked to Uncharacterized protein, DUF357 family
thermo9.02768	42	43	0.2	COG1888 (arCOG04140)		Linked to ArsR transcriptional regulators; PDB: 2X3D [69]
thermo9.01533	42	42	0.31	COG1531 (arCOG01302)		Linked to MBL-fold metallohydrolase superfamily; predicted RNA cyclic group end recognition domain [70]
thermo9.01369	42	42	0.42	(arCOG05869)		PDB: 2K4N; linked 23S rRNA G2069 N7-methylase RlmK or C1962 C5-methylase RlmI;
methano7.000565	41	48	0.48	COG4744 (arCOG03208)	DUF2149	Membrane protein; linked to biopolymer transport protein TolQ
methano7.001417	41	41	0.48	COG3377 (arCOG04424)	DUF1805	PDB: 1QW2; linked to tRNA G10 N-methylase Trm11
methano7.001273	41	41	0.45	COG4050 (arCOG04903)	DUF2112	In a conserved context with uncharacterized protein, DUF2102 family and others; single CxxC motif; methanogenesis maker 5
methano7.001697	41	41	0.4	(arCOG04388)		Linked to Uncharacterized protein, DUF2551 family
methano7.001273	41	41	0.45	COG4050 (arCOG04903)	DUF2102	Methanogenesis maker 6; linked to DUF2112
flavo9.00782	50	50	0.47	–	DUF4286	Linked to outer membrane protein assembly factor BamD
flavo9.01459	50	50	0.45	–		Linked to RuvX, Holliday junction resolvase; SRPBCC domain, Hsp90 cochaperone in yeast [71, 72]; putative hydrophobic ligand binding site
flavo9.00789	50	50	0.45	–	DUF2797	Linked to GH3 auxin-responsive promoter; contains Zn ribbon
flavo9.01638	50	50	0.30	–		SRPBCC domain, also see flavo9.01459
flavo9.02618	50	50	0.30	–	DUF4254	Linked to ADP-heptose:LPS heptosyltransferase, RfaF
deino9.00587	33	33	0.34	–		Annotated as quinate 5-dehydrogenase; present in Thermus and other bacteria
deino9.01277	33	33	0.35	–	DUF4385	Linked to DNA-binding ferritin-like protein Dps; present in Thermus
deino9.00288	33	33	0.45	–		Linked to uncharacterized membrane protein, Outer membrane protein assembly factor BamB, contains PQQ-like beta-propeller repeat; secreted; present in Thermus
deino9.01656	33	33	0.49	–		
deino9.02309	32	32	0.33	–	DUF1844	Linked to D-Tyr-tRNA(Tyr) deacylase
paen9.03935	66	66	0.22	COG4472	DUF965	Linked to Alanyl-tRNA synthetase, AlaS; homolog of IreB, acting a negative regulator of cephalosporin resistance [73]
paen9.05835	66	66	0.34	–		Next uncharacterized protein YrrD, contains PRC-barrel domain and Cysteine sulfinate desulfinase/cysteine desulfurase or related enzyme; Zn ribbon domain
paen9.02641	66	66	0.37	–		YokU-like protein, putative antitoxin RelE fold family
paen9.02767	66	66	0.39	–		Linked to uncharacterized membrane protein SpoIIM, required for sporulation
paen9.02361	66	66	0.4	–	DUF1499	
rhodo7.006964	53	53	0.07	–	DUF2469	Often found in Actinomycetes clustered with signal peptidase and/or RNAse HII
rhodo7.004823	53	53	0.14	–	DUF3039	Possibly metal-binding; Hx(20)C…CxxC motif
rhodo7.005227	53	54	0.159	–	DUF3151	Linked to Uncharacterized membrane protein YgaE, UPF0421/DUF939 family
rhodo7.003034	53	53	0.253	–	DUF4191	2TM domain, in operon with Lipoate synthase LipA
rhodo7.002008	53	53	0.615	–	DUF3090	Contain CxxC..HxC motif, putative metal-binding protein

## Conclusions

In this work, we developed a quantitative measure of sequence variability in protein families and investigated the connections between variability and various genomic and biological features. Overall, the association of variability with other genomic features follows the expected trends that were previously established in other contexts [8, 9]. Approximately half of the variance in variability values can be explained by the analyzed features, of which gene paralogy is most impactful. Correlation between paralogy and variability likely comes from acquisition of distant paralogs and xenologs introducing sequence variants that are more distant than those that could have evolved in situ during the lifetime of the clade. Notably, more than 50% of the highly variable ($V>2$) csCOGs in each clade have homologs in at most one other clade of the 8 analyzed, and more than half (872 out of 1732) of the non-ancestral highly variable csCOGs have more than 1.25 paralogs per genome (Fig. 5, Additional file 8: Table S3). These observations suggest that HGT is a major evolutionary force that shapes the distribution of family-level variability in prokaryotic genomes.

At the level of individual alignments, the distribution of variability across the alignment columns is typically smooth and centered around a value characteristic of the given csCOG. Protein families that combine low-variability and high-variability regions within the same alignment are relatively rare, with highly variable segments often located in indel-rich regions. Such regions are typically lineage-specific and often are completely absent in orthologs from other taxa. The apparent high density of indels also makes alignment reconstruction locally uncertain even between closely related organisms, obscuring the differences between substitution- and indel-generated diversity. Microsatellite-like and low-complexity regions only weakly correlate with protein family variability, suggesting that polymerase slippage is not the major mechanism generating variability at the individual protein level.

Comprehensive analysis of evolutionary regimes requires careful phylogenetic reconstruction, is subject to constraints on evolutionary distances, alignment quality, and confidence in ortholog detection, and could be highly sensitive to evolutionary rate variability between lineages. Here we show that csCOG-level variability estimates can serve as the first approximation for the relative evolutionary rate and appear to be useful in partitioning genome-scale datasets according to sequence conservation as well as for identification of essential genes and subfunctionalized paralogs.

## Methods

### Genome sets and genome phylogeny

Genome assemblies were downloaded from Genbank (Additional file 9: Table S4)*.* The 16S rRNA sequences were aligned using MUSCLE [51] and the tree was reconstructed using FastTree [52] with the GTR evolutionary model, and discrete gamma model with 20 rate categories; the tree topology was used as the proxy for the genome history.

### Construction of clade-specific clusters of orthologous genes (csCOGs)

Initial clusters of protein sequences were obtained using MMSEQS2 [53] with the similarity threshold of 0.5. Multiple alignments of cluster members were generated using MUSCLE [51] and compared to each other using HHSEARCH [54]. Clusters that aligned to each other along most of the protein lengths (HHSEARCH hit covering ≥ 75% of the cluster consensus length) were merged using HHALIGN [53]. Approximate maximum-likelihood phylogenetic trees were built for each merged cluster using FastTree [52] with WAG evolutionary model, and gamma-distributed site rates. Trees were parsed into subtrees that maximize the tradeoff between the number of paralogs and the representation of genomes. Formally, within a tree including leaves coming from $S$ different genomes, a clade that contains ${P}_{C}$ leaves from ${S}_{C}$ different genomes is defined to have the paralogy ratio of ${P}_{C}/{S}_{C}$ and genome coverage ratio of ${S}_{C}/S$. The clade with the maximum coverage-paralogy tradeoff index ${S}_{C}^{2}/({P}_{C}S)$, if distinct from the tree root, is considered a csCOG and is removed from the tree, after which the procedure is repeated with the pruned tree until convergence.

### Protein sequence analysis and phylogenetic reconstruction

Multiple sequence alignment of prokaryotic COG [48] and Pfam [55] profiles in the CDD database (as of 2019) were used as queries for Position-Specific Iterated BLAST program [56]. The search against the database, consisting of proteins sequences encoded in our set of genomes, was run at e-value cutoff of 0.0001; the best hits were used to annotate the sequences. Membrane proteins were predicted using TMHMM [57], secreted proteins using SignalP [58], low complexity regions were identified using SEGmasker program [59]. Disordered loops in proteins were predicted using IUPred2A [39]. When the entire csCOG, rather than an individual protein, needed to be characterized by a particular feature (e.g., prevalence of transmembrane segments or signal peptides), the fraction of proteins with this feature was calculated. Multiple alignments for selected csCOGs were generated using MUSCLE [51]. Approximate maximum-likelihood phylogenetic unrooted tree was built for each alignment using FastTree with JTT evolutionary model, and 20 discrete rate categories [52].

### Evolutionary history reconstructions

The binary phyletic pattern of csCOGs (presence-absence of the given gene across the species) within each lineage was analyzed using GLOOME [60]. Differences of posterior probabilities of ancestral presence between the parent and descendant nodes of ≥ 0.5 were interpreted as either gains or losses depending on the sign. At least one gain event was detected for an overwhelming majority of the extant genes. Genes with the posterior probability ≥ 0.5 at the tree root were classified as ancestral; many genes were gained multiple times in the history of a given csCOG (in some cases, re-acquired after a loss). The rare exceptions are those genes for which the phyletic pattern did not allow a specific gain point to be inferred. The total number of gains and losses in a csCOG history, regardless of their precise location, was estimated as the sum of positive and negative differences of ancestral posterior probabilities, respectively. One of the important characteristics of a csCOG history is whether it is inferred to be ancestral in the given clade or to have been acquired later in the history of the corresponding group of genomes.

### Protein variability estimation

For each csCOG alignment with at least 8 non-identical protein sequences and at least 60 aligned columns (excluding singular insertions), homogeneities of all alignment columns were calculated ([26] and Additional file 2). Specifically, all sequences in an alignment of $N$ sequences were assigned equal weights ${w}_{i}=1/N$. Next we introduce an amino acid score against an alignment column; for any given amino acid $x$, ${Q}_{x}=\sum_{i=1}^{N}{w}_{i}{S}_{{a}_{i},x}$, where ${a}_{i}$ is the amino acid in the *i*th sequence of the column and ${S}_{{a}_{i},x}$ is the score for amino acids ${a}_{i}$ and $x$ according to the chosen pairwise score matrix (here BLOSUM62 [61]). The amino acid $c$, satisfying the $c=\underset{x}{\mathrm{argmax}}{Q}_{x}$, is selected as the effective consensus amino acid for this alignment position (i.e. the one which is most similar to the assortment of amino acids in the column). To calibrate the consensus score ${Q}_{c}$, the expectation of the score is calculated, comparing the alignment column against a random assortment of amino acids, ${Q}_{R}=\sum_{b}{f}_{b}{Q}_{b}$, where ${f}_{b}$ represents relative frequencies of amino acids across the entire protein database (frequencies summing up to 1). The homogeneity of an alignment column is calculated as $h=\mathit{max}(\frac{{Q}_{c}-{Q}_{R}}{{S}_{c,c}-{Q}_{R}},0)$. When gaps were present in the column, the homogeneity was calculated using the scores for non-gap characters. The homogeneity measure $h$ is confined within the range $0\le h\le 1$, where a random column has homogeneity of 0 and a column containing an invariant amino acid has homogeneity of 1.

The (arithmetic) mean homogeneity values were calculated across all alignments in the given clade ${h}_{T}$ and across each csCOG alignment ${h}_{C}$. Relative variability of a csCOG was calculated as ${v}_{C}=\frac{(1-{h}_{C}){h}_{T}}{(1-{h}_{T}){h}_{C}}$, valid under the reasonable assumptions of ${0<h}_{C}\le 1$ and ${0<h}_{T}<1$ (at least some alignment columns in any given csCOG match better than expected by pure chance and at least some alignment columns across all csCOGs are less than perfectly homogeneous). This transformation places variability in the range of $0\le {v}_{C}\le \infty$, and a csCOG with ${h}_{C}={h}_{T}$ would have ${v}_{C}=1$ (that is, a csCOG with mean homogeneity equal to the clade-wide mean has a relative variability of 1). The same calculation can be performed for each individual alignment column with $h>0$, obtaining the position-specific variability estimate (columns with $h=0$ can be assigned arbitrarily high variability value).

To obtain the csCOG-specific distributions of homogeneity values, first, the homogeneity profile along the sequence was smoothed using a Gaussian kernel with bandwidth of $b=20$ (${h}_{i}={\sum }_{j}{h}_{j}{K}_{i,j}/{\sum }_{j}{K}_{i,j}$ where ${K}_{i,j}=\mathrm{exp}({(\left(k-i\right)/b)}^{2}$ for homogeneity in the *i*th position). Then, these values were used to evaluate the probability density function (p.d.f.) for 101 points in the range of $0\le h\le 1$ [62]. The Hellinger distances between all pairs of distributions were calculated using these p.d.f. estimates (Additional file 2). These distances were embedded into a 2-dimensional plane using Classical Multidimensional Scaling (*cmdscale* function in R).

### Search for microsatellite-like regions in protein coding sequences

Microsatellite-like regions (MSRs) were identified in protein coding nucleotide sequences using the compositional order approach by detecting irregular recurrences of short *k*-mers [63–65]. In random sequences, the probability of identifying a motif of length *k* that recurs more than *n* time in a sequence of length *L* is determined by the binomial distribution, $P\left(L,p;\ge n\right)=\sum_{i=n}^{L-k+1}\left(\genfrac{}{}{0pt}{}{L}{i}\right){p}^{i}{(1-p)}^{L-i}$, where *p* is the probability of selecting a *k*-mer over an alphabet *A*, such that $p=1/{A}^{k}$. Here, we define non-random recurrences of a *k*-mer as those that recur with $P<{10}^{-6}$, locally (that is within a window of 1000 characters). Thus, for example, for nucleotide sequences ($A=4$), and using $k=6$ (hexamers), this definition translates into the search of non-random recurrence of hexamers that occur at least 6 times within 1000 bp, at least 5 times within 500 bp, at least 4 times within 200 bp and at least 3 times within 80 bp.

To extract MSR, we identify all the non-random locations of all *k*-mers, allowing motifs to overlap, and define MSR as the coverage of non-random recurrences with interval distance between consecutive recurrences of the same motif ($I$) smaller than the motif length $k$ (i.e., $I\le k$). For example, using $k=6$ in the sequence AAAAAAAAA, the hexamer AAAAAA recurs 4 times, with $I=1$ between consecutive recurrences, capturing runs of nucleotides. Similarly, in the sequence ATATATATATA the dinucleotide tandem repeats are captured by the hexamers ATATAT and TATATA, each recurring 3 times, with distance interval $I=2$ (recurring twice each), and so forth up to $I=6$. To ensure that all non-random patterns are identified, this procedure is done for all $k$ up to 6 (i.e., $k=1..6$). MSR regions that are separated from each other by less than *k*, are merged into a single region. Using this definition, MSRs in nucleotide sequences include the conventionally defined regions of microsatellite instability (i.e., tracks of units composed of a few bp, typically 1–5 bp). The MSR and LCR measures are correlated, but distinct across the csCOGs (Additional file 10: Table S5).

### Statistical analysis

Each csCOG within each lineage was classified with respect to several quantitative or qualitative features (see the list of features in Additional file 1: Table S1). Variability was analyzed as a quantitative (real number continuous) variable; the rest were represented as categorical variables. Associations between variability and each of the other features were analyzed using the ANOVA test for the distribution of variability of csCOGs within the categories and between the categories; the significance of the association was estimated using the F-statistics (ratio of between- to within-group variances); the strength of association was estimated as the relative decrease of the total variance due to grouping csCOGs into feature categories. The total explanatory power of all features was estimated as the unadjusted ${R}^{2}$ of the generalized linear model, predicting the variability of a csCOG given the categorical values of all 9 features using the *lm* function in R. Each model was subject to stepwise reduction using the *step* function in R, which attempts to remove low-impact explanatory variables based on the Akaike Information Criterion; successful reduction attempts were reported.

## Supplementary Information

**Additional file 1: Table S1**. Features used for association analysis.

**Additional file 2: Fig. S1**. The scheme of phylogenetic tree for WcaE-like glycosyltransferases of COG1216. Approximate maximum likelihood phylogenetic tree was built using FastTree (WAG evolutionary model, gamma distributed site rates) [52] based on multiple alignment of 1423 COG1216 sequences from complete genomes of archaea and bacteria. Six branches (A-D, colored red) belong to same csCOG (sulfo9.00007) and are indicated on the Fig. 6 for respective genes. Other archaeal sequences or branches are colored yellow and bacterial—black.

**Additional file 3: Fig. S2**. Fractions of conserved, medium and variable positions in each csCOG by lineage. Red dots correspond to 34 families described in the Table 2.

**Additional file 4: Fig. S3**. Selected multiple alignments for 34 families with high fraction of conserved and variably positions. A. The plots below alignment show the propensity for disorder or order: red line—disordered loops (IUPred2); Blue line—ordered structures (ANCHOR2). Sequences identified by protein accessions. csCOG number and protein family description is indicated for each alignment. B. Several alignments of orthologous protein subfamilies without hypervariable regions. Alignments were colored using http://www.bioinformatics.org/sms2/color_align_cons.html server with default amino acid groups with 100% consensus.

**Additional file 5: Fig. S4**. Amino acid frequency PCA for conserved and variable positions for families from Table 2. High- (V > 2) and low-variable (V < 0.5) sites were extracted from the alignments of 34 csCOGs (Additional file 4: Fig. S3); relative frequencies of amino acids were computed for all 68 (34 × 2) subsets. Principal Component Analysis of amino acid frequencies was performed using the prcomp function of R package. The plot shows the location of the high- (red circles) and low-variable (cyan triangles) and the contributions of individual amino acids (blue arrows) in the plane of the first two principal components.

**Additional file 6: Table S2**. Selected examples of potential subfunctionalization of paralogs (proteins which belong to the same COG). Selected by the following criteria: (1) present in most genomes in the respective lineage; (2) have small number of paralogs (3) have low and high variability estimates.

**Additional file 7: Fig. S5**. Linear Discriminant Analysis of gene essentiality in *S. islandicus*.

**Additional file 8: Table S3**. Number of local COGs, broken down by ancestrality, paralogy and variability.

**Additional file 9: Table S4**. Genomes accessions and summary of genomic data used in this work.

**Additional file 10: Table S5**. Correlation between the fraction of microsatellite regions (MSR) and low complexity regions (LCR) across csCOGs.

## Data Availability

Additional file 1: Raw data used for analyses in this work. Available at: https://ftp.ncbi.nlm.nih.gov/pub/makarova/Supplement/Variability. Additional file 2: Code to calculate homogeneity, variability and the Hellinger distance between homogeneity distributions. Available at: https://ftp.ncbi.nlm.nih.gov/pub/makarova/Supplement/Variability. csCOG data is available by request from authors.
