# Supplementary material for: Analysis of lineage-specific protein family variability in prokaryotes combined with evolutionary reconstructions
Source: Biol Direct. 2022 Aug 30;17:22. doi: 10.1186/s13062-022-00337-7 (PMC9425974; doi:10.1186/s13062-022-00337-7)
Supplement: Supplementary file 1 — Additional file 1: Table S1. Features used for association analysis. [file 13062_2022_337_MOESM1_ESM.docx]

| **Character** | **Estimate** | **Categories** |
| --- | --- | --- |
| Variability | Calculated from the average homogeneity of the COG alignment | Quantitative |
| Paralogy | Ratio between the number of proteins and the number of genomes in a COG | P0 if *x*<1.25; P1 if 1.25<*x*<2; P2 if *x*>2 |
| Gain propensity | Number of intra-lineage gene gains in the history of the COG | G0 if *x*<0.5; G1 if 0.5<*x*<2.5; G2 if *x*>2.5 |
| Ancestrality | The earliest appearance of the GOG in the history of the lineage | A0 if ancestral to the lineage; A1 if acquired on an internal tree branch; A2 if acquired on the terminal branch |
| Membrane | Fraction of COG members with at least one TM segment | M0 if *x*<0.333; M1 if *x*>0.333 |
| Secreted | Fraction of COG members with predicted signal peptide | S0 if *x*<0.333; S1 if *x*>0.333 |
| Core | Does the COG belong to the bacterial or archaeal core? | C0 if yes; C1 if no |
| Function | COG functional category | Categorical; genes with no COG assignment were counted as the S category (unknown) |
| LCR | The average fraction of protein sequences in the COG identified as low complexity regions | L0 if *x*<0.15; L1 if *x*>0.15 |
| MSR | The average fraction of protein sequences in the COG identified as microsatellite-like regions | R0 if *x*<0.15; R1 if *x*>0.15 |
