# Supplementary figures and images for: Analysis of lineage-specific protein family variability in prokaryotes combined with evolutionary reconstructions
Source: Biol Direct. 2022 Aug 30;17:22. doi: 10.1186/s13062-022-00337-7 (PMC9425974; doi:10.1186/s13062-022-00337-7)

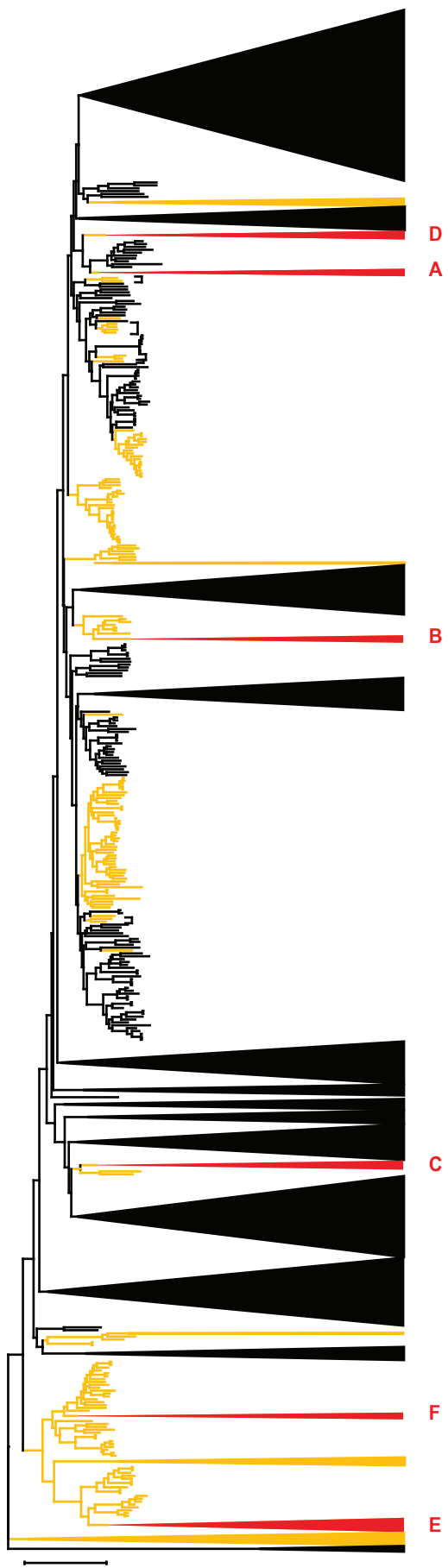

Supplement: Supplementary file 2 — Additional file 2: Fig. S1. The scheme of phylogenetic tree for WcaE-like glycosyltransferases of COG1216. Approximate maximum likelihood phylogenetic tree was built using FastTree (WAG evolutionary model, gamma distributed site rates) [52] based on multiple alignment of 1423 COG1216 sequences from complete genomes of archaea and bacteria. Six branches (A-D, colored red) belong to same csCOG (sulfo9.00007) and are indicated on the Fig. 6 for respective genes. Other archaeal sequences or branches are colored yellow and bacterial—black. [file 13062_2022_337_MOESM2_ESM.pdf]

PCA – Biplot

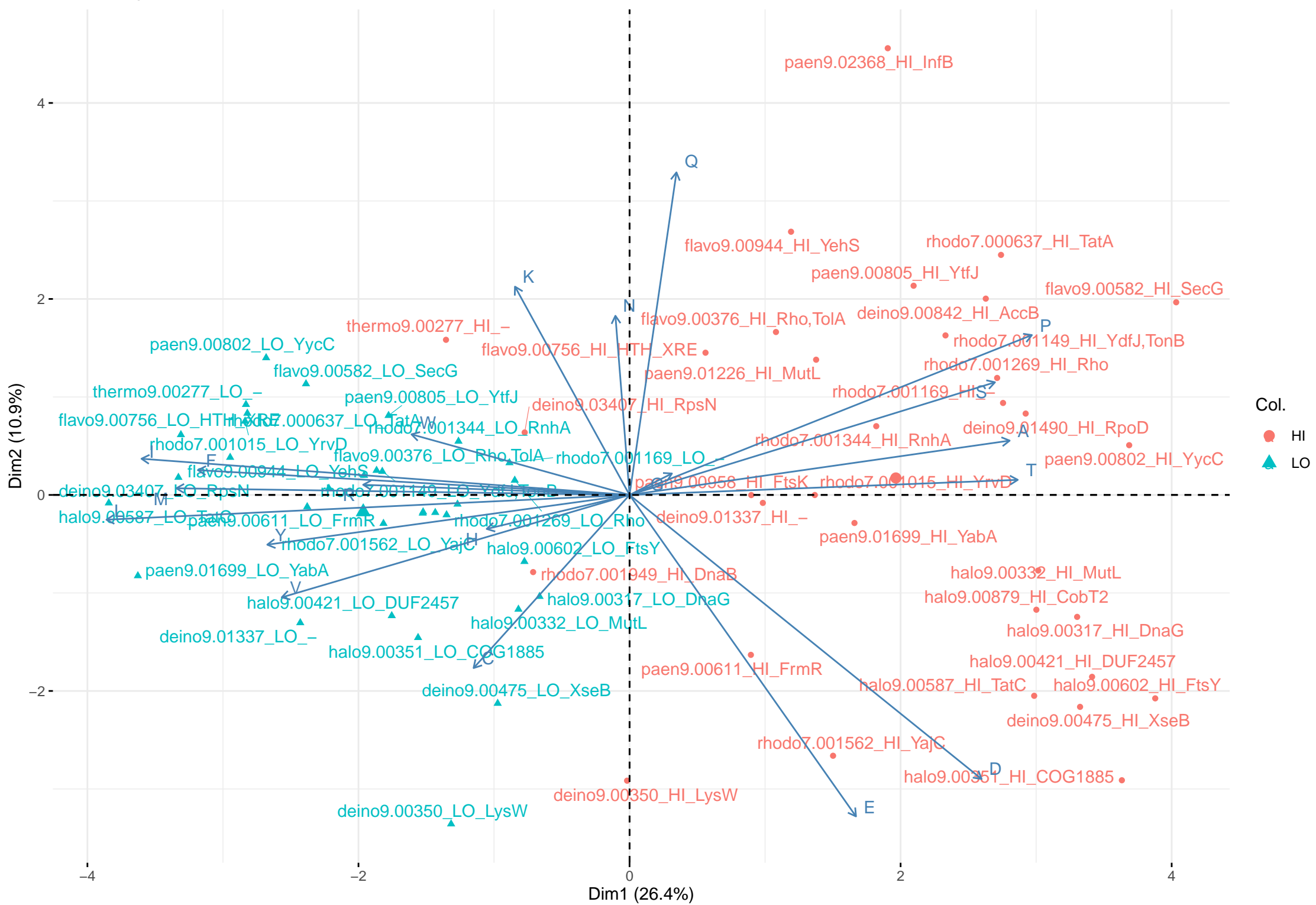

Supplement: Supplementary file 5 — Additional file 5: Fig. S4. Amino acid frequency PCA for conserved and variable positions for families from Table 2. High- (V > 2) and low-variable (V < 0.5) sites were extracted from the alignments of 34 csCOGs (Additional file 4: Fig. S3); relative frequencies of amino acids were computed for all 68 (34 × 2) subsets. Principal Component Analysis of amino acid frequencies was performed using the prcomp function of R package. The plot shows the location of the high- (red circles) and low-variable (cyan triangles) and the contributions of individual amino acids (blue arrows) in the plane of the first two principal components. [file 13062_2022_337_MOESM5_ESM.pdf]

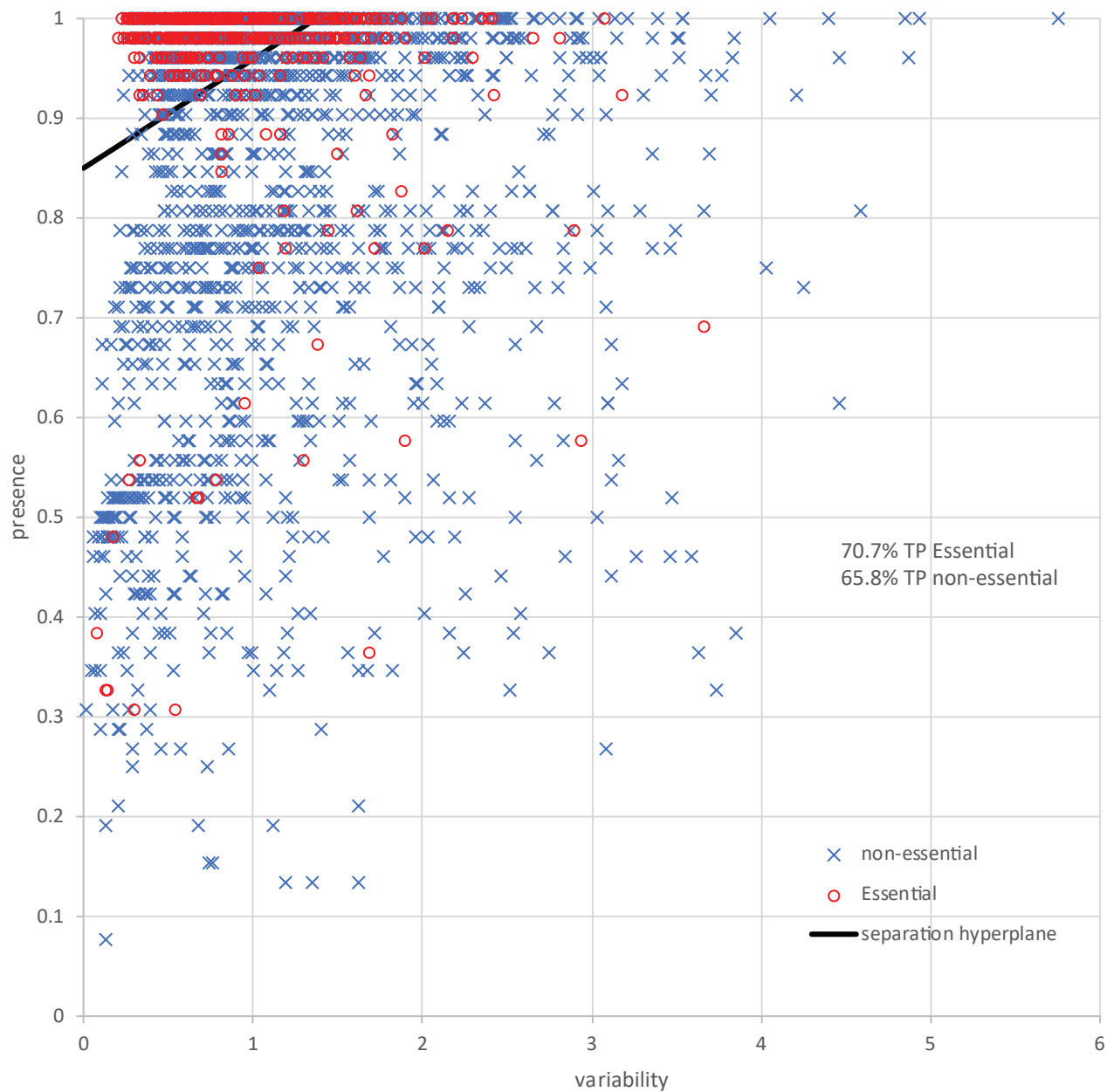

Supplement: Supplementary file 7 — Additional file 7: Fig. S5. Linear Discriminant Analysis of gene essentiality in S. islandicus. [file 13062_2022_337_MOESM7_ESM.pdf]
