# Supplementary material for: Analysis of lineage-specific protein family variability in prokaryotes combined with evolutionary reconstructions
Source: Biol Direct. 2022 Aug 30;17:22. doi: 10.1186/s13062-022-00337-7 (PMC9425974; doi:10.1186/s13062-022-00337-7)
Supplement: Supplementary file 3 — Additional file 3: Fig. S2. Fractions of conserved, medium and variable positions in each csCOG by lineage. Red dots correspond to 34 families described in the Table 2. [file 13062_2022_337_MOESM3_ESM.pdf]

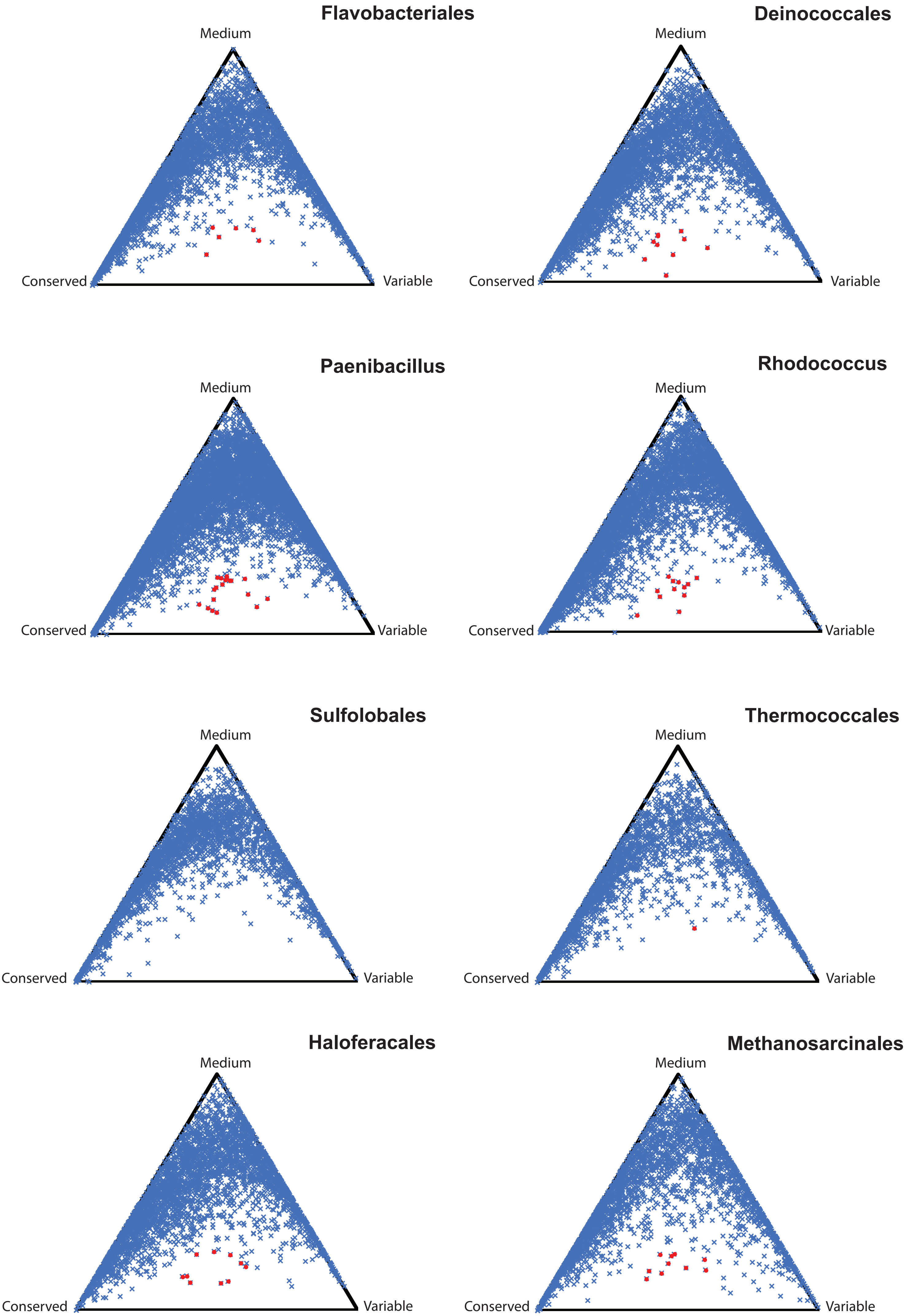
Flavobacteriales
Medium
Conserved
Variable
Deinococcales
Medium
Conserved
Variable
Paenibacillus
Medium
Conserved
Variable
Rhodococcus
Medium
Conserved
Variable
Sulfolobales
Medium
Conserved
Variable
Thermococcales
Medium
Conserved
Variable
Haloferacales
Medium
Conserved
Variable
Methanosarcinales
Medium
Conserved
Variable
