# Supplementary material for: Analysis of lineage-specific protein family variability in prokaryotes combined with evolutionary reconstructions
Source: Biol Direct. 2022 Aug 30;17:22. doi: 10.1186/s13062-022-00337-7 (PMC9425974; doi:10.1186/s13062-022-00337-7)
Supplement: Supplementary file 4 — Additional file 4: Fig. S3. Selected multiple alignments for 34 families with high fraction of conserved and variably positions. A. The plots below alignment show the propensity for disorder or order: red line—disordered loops (IUPred2); Blue line—ordered structures (ANCHOR2). Sequences identified by protein accessions. csCOG number and protein family description is indicated for each alignment. B. Several alignments of orthologous protein subfamilies without hypervariable regions. Alignments were colored using http://www.bioinformatics.org/sms2/color_align_cons.html server with default amino acid groups with 100% consensus. [file 13062_2022_337_MOESM4_ESM.docx]

**Supplementary Figure 3. Selected multiple alignments for 34 families with high fraction of conserved and variably positions.**

A. The plots below alignment show the propensity for disorder or order: red line - disordered loops (IUPred2); Blue line - ordered structures (ANCHOR2). Sequences identified by protein accessions. csCOG number and protein family description is indicated for each alignment.

B. Several alignments of orthologous protein subfamilies without hypervariable regions.

Alignments were colored using http://www.bioinformatics.org/sms2/color_align_cons.html server with default amino acid groups with 100% consensus.

**A.**

**flavo9.00376, Transcription termination factor Rho**

WP_072941811_1 MFDISALKEMKLAELQEIAKAAKTIKFNGVKKETLIGQILEHQAN-HL---AP-KP-------ETKP--EA-DA-------EESK---------PK--RARI--V----------P-----------------------VKK--AVIQKNKT-APVVIKEEKSIE----SS--A-DVPQE-L------DFSAAET----- 110

WP_091525983_1 MFDKNVLKGMKLPELKEIAKKIGVEKLN-LKKDELIDTILKLQDE-FQ-K-NQ-PK-AETPV-KEEP--AV-SN--QPTE-KKEK-------------RKRI--H----------N----------------------------ATEEQTEQ----PSLPENPQP----------------------------------- 95

WP_091096797_1 MFDKNVLKGMKLPELKELAKTIGVEKIN-LKKDELIDTILTMQDR-FQ-G-NE-PS-AE-----NQP--PK-NA--AP---KKEK-------------RKRI--N----------P----------------------------STEETQDQ----NLFSESVAE----------------------------------- 89

WP_054406085_1 MFDISELKEMKLTELQDIAKKANISKFRGLKKDELIYQILDHQAA-NP---AK-IQ-PL----FEDP--AT------DAEAAAPK---------AK--RARI--S----------K---------------PAADAVPQTAT--AEPVTEAP----AEKPVRAKK--------A-DIAEQ-------------------- 109

WP_091315979_1 MFDTSILKGMKLSDLQEIAKVANIQKFKTLKKDELITQILDAQN--SS---------------EDKP--------------KAEK-------P-AK--KVTP--V----------A------K----------------AVK--EVAEVQEV----APVVADNDA--------K-------------------------- 85

WP_026992511_1 MFDISELKEMKLPELQEIAKKAKISKFRGLKKDELVYQILDHQAA-NP---AI-IQ-PL----FEET--ATAAPLVADDAAAENK---------TK--RARM--T----------KNAAEEAKILATMPETPARTEKPEETP--AHPIIEPV----PARQPRADK--------A-FVKKDDA------------------ 132

WP_073363513_1 MFDISTLKTMKLSELQDIAKLAQTIKFAGVKKEALIELIIAHQEA-VD---RG-ET-------PPST-----------------F---------SK--RGRR--P----------R-----------------------ISA--QESASGNL-FTADVPTEVTGQ-EASTE-EK-RVEEP-K-RRGR-KPKQPES-APTP 115

WP_014388556_1 MFDQSTLKEMKLSDLQEIAKVANIKKFKTLKKEELITQILDVQET-SQ---------------ADKT--------------TEKKKPALKSTT-DK--KAKS--N----------V------N----------------PVV--AENVVEEP----TPVQEKKKA--------S-------------------------- 93

WP_014166209_1 MFDISVLKEMKLNELQEIAKAAKIKNFKTLKKDDLIYQILDFQAS-NP---EK-LT-PE----ANST--SE----------DTSK---------PK--RTRM--V----------K-----------------------DKKEVSEPEKVDA----I-----QET--------T-LFTEE-L------------------ 95

WP_072784887_1 MFDIEVLKEMKLSDLQEIAKVAQIKKYRSLKKDELIYQILDMQAA-NP---ET-VK-------KEEQ--------------VEEK----TAVK-PK--RLRI--S----------A-----------------------KVE--S----EST----ERIVPKDQE--------E-------V------------------ 89

WP_014084047_1 MFDHTALKQMKLTELQEIAKLAKTIKIANVKKDTLITMILDHQAK-T-------NQ-------DQNV--VS-EP-------VAEK---------PK--RNRI--VAPKDEKIQATP-----------------------KKT--ADKPNKKQ----HVETKVQQE--I-VE--D-KVDAP-V------MDATPII-ENKS 119

WP_026715962_1 MFDIEKLNEMKLSDLHEIAKAAKLKKVSALKKDELIYQILDHQAL-NPIPVDV-VK-------TDAS--------------IEKR----AKVTTPK--AKKP--V----------A-----------------------KKA--TKPVVETT----TKATPTEET--------P-------I------------------ 97

YP_001295636_1 MFDISVLKEMKLPELQEIAKVAKIAKFKTLKKDELVYQILDYQAA-NP---EK-IA-PI----ATETI-SV----------IDEK---------PK--RARV--V----------E-----------------------AKE--AKPIVSKK----VIAKPPVQQ--------E-VITFE-E------------------ 99

WP_011962876_1 MFDISVLKEMKLPELQEIAKVAKIAKFKTLKKDELVYQILDYQAA-NP---EK-IA-PI----ATETI-SV----------IDEK---------PK--RARV--V----------E-----------------------AKE--AKPIVSKK----VIAKPPVQQ--------E-VITFE-E------------------ 99

WP_034097990_1 MFDISVLKEMKLPELQEIAKVAKIAKFKTLKKDELVYQILDYQAA-NP---EK-IA-PI----ATETI-SV----------IDEK---------PK--RARV--V----------E-----------------------AKE--AKPIVSKK----VIAKPPVQQ--------E-VITFE-E------------------ 99

WP_091477336_1 MFDISVLKEMKLTELQEIAKVAKIAKFKTLKKDELVYQILDYQAA-NP---EK-IA-PI----VPETT-PV----------VDEK---------PK--RARI--V----------E-----------------------PKV--AKTITPKA----VVT-PPVQQ--------E-VISFE-V------------------ 98

WP_035126216_1 MFDSSALKGMKLTELQEIAKLAKTIKFNGVKKDALIEQILQHQAA-TA---TE-KS-------QEKVV----AP-------KEEK---------AK--RARI--A----------P-----------------------DAS--PKIEKNST----DLFSEEVAN----------------------------------- 90

WP_073312099_1 MFDISVLKEMKLSELQEIAKIAKISKFKTLKKDELVYQILDYQAA-NP---EK-ISNPD----TSSA--PV----------QEAK---------QK--RARI--V----------K-----------------------ETK----PPVVNK----V-----EDQ--------Q-AIT---F------------------ 90

WP_091464749_1 MFDTSTLKEMKLSDLQEIAKVAKIQKFKTLKKDDLIAQILELQNE-SS---------------EEKP--------------KAAK-------A-SK--KAA-------------------------------------------EKPVVEEQ----VSEVVVKEG--------K-------------------------- 79

WP_023574714_1 MFDISVLKEMKLPELQEIAKIAKINKYRNLKKDELVYQILDYQAA-NP---EV-VK-PA----LQET--PQ----------PEEK---------AK--RARIKPV----------K-----------------------EES--EQPAQAEK----KTFTPKLHK--------K-VAETE-L------------------ 100

WP_026712226_1 MFEISVLKEMKLSELQEIAKAAKTIKFNGVKKDDLIQKILETQAQ-SV---AA-PV-------QKE------SA-------EADK---------PK--RARI--V----------A-----------------------KPK---SEEKAQP----GLFAEEPAV--------------------------------QVE 90

WP_091140744_1 MFDISALKTMKLPELQEIAKSAHAIKVAGKKKEVLIEEILKRQES-AQ---GT-AS-------EPSA-----------------K---------PK--RARI--G----------A-----------------------DKK--PTETAQNL-FS---------------------------------EPQTPT------ 83

WP_091398316_1 MFDISVLKEMKLPELQDIAKS-KKIKFNGVKKEVLVNQILDQQAS-EP---KE-NT-------SPTD-----ET-------AAEK---------PK--RNRI--A----------A-----------------------ETK--PAISKASK----TLFSASDET----------------------------------- 88

WP_066334474_1 MFDISALKEMKLTELQQIAKATKSIKFNGVKKEILIGHILEYQKN-SA---DP-TS-------DNSV--KD-AV-------VEAK---------PQ--KTVV--A----------P-----------------------SEK--PIVQKPRI-QP-NRAKNNIQN----NT--Q-DPAES-I---LK-ESAVLDT---ME 113

WP_023571963_1 MFDISVLKEMKLPELQEIAKVAKINKYRYLKKDELVYQILDYQAA-NP---EV-VK-PA----LAEQ--PQ----------QEEK---------AK--RARIAPA----------K-----------------------GQV--KTPAENKK----TPFVPKSDK--------KEIKEIA-A------------------ 101

WP_026979818_1 MFDIDVLKEMKLPELQEIAKVAKINKYRYLKKEELIYQILDLQAA-NP---DV-IE-PAKPAPATAP--EV----------KQEK---------SK--RARI------------------------------------------APKAEAEK----TVQAPATKE--------V-------------------------- 91

WP_024982133_1 MFDIAALKELKLSELQEIAKKAKTIKFNGVKKETLIGLILEYQNA-AV---ATVPT-------KETV--QD-KA-------EIEK---------PKKERVRI--L----------P-----------------------SKK--EKLVVNSS-EA-TLQETVIEE----NVLNR-EVQNP-L------------------ 107

WP_026705806_1 MFDISVLKEMKLAELQEIAKL-AKIKATGVKKETLISQILERQVA-TQ---EV-AT-------KDNTI----TE-------DTTK---------SK--RARI--A----------P-----------------------AKKNPATTTQENS----DLFSKAENN----TP-------------------------ETVQ 97

WP_022828308_1 MFEISALKQMKLAELQDIAKL-AKIKSAGVKKDLLVEQILQKQSE-AK---PS-PA-------AETVQ----TP-------SDES---------PK--RTRI--V----------P-----------------------TKK--QDDAASDL----NLAATDVQK----------------------------------- 89

WP_035657734_1 MFDISALKEMKLSELQQIAKAAKTIKFTGVKKEVLIANILDFQKS-LA---AE-NV-------TEEKAIPV-AD-------ADDT---------AK--RARI--V----------P-----------------------AKK--PANPKIKA----KVAVEKEEQ----VV--E-KDSEK-L------DFPAE------- 107

WP_089059798_1 MFDISALKEMKLSELQEIAKLAKTIKFNGVKKDTLITQILAHQEA-AS---AA-PE-------PTIV--EK-EI-------TDDK---------PK--RARI--A----------P-----------------------TKK--I-VSKNAP----VLEFDKIEE----AP--K-EIETP-A------AVENKET----- 106

WP_012026627_1 MFDISALKEMKLSELQEIAKLAKTIKINGVKKDTLITQILAHQEA-AA---AP-PA-------ETIA--EK-EV-------NTEK---------PK--RARI--A----------P-----------------------AKK--T-VSKSAP----VLEFDAVEE----AP--K-EIVTP-AAV---------------- 101

WP_072972573_1 MFDISALKEMKLSELQEIAKLAKTIKFNGVKKETLISQILAHQET-TE---KP-PV-------NVAP--QA-EI-------ADDK---------PK--RARI--L----------P-----------------------VKK--AAISKDAP----VLEFNKVEE----TA--E-KIEEA-PEKVVTPKISKPNA----- 113

WP_029273001_1 MFDISALKEMKLSELQEIAKLAKTIKFNGVKKEALISQILSHQEA-TI---EP-PV-------TAVA--NA-EI-------ADDK---------PK--RARI--A----------P-----------------------VKK--AAISKNAP----VLEFDKVEE----QA--Q-EEKEV-P------VVSKPNS----- 107

WP_099708795_1 MFDISALKEMKLSELQEIAKLAKTIKFNGVKKETLISQILAHQES-TV---AP-PA-------NAVS--EK--V-------EDDK---------PK--RARI--V----------P-----------------------AKK--TPIAKDAP----VLEFDKVEE----AP--K-QVKTP-AISKATPKAQQASKVAEIQ 117

WP_026713087_1 MFDISALKEMKLSELQQIAKAAKTIKYNGVKKEALITSILEFQST-LA---AT-VV-------ADEA--PV-AE-------ADNK---------PK--RVRI--T----------P-----------------------AKK--QSAPK-NE----VVAHEETAE----AV--Q-TD-EK-T------AFQADTV----- 105

WP_035640151_1 MFDISVLKEMKLAELQEIAKTAKTIKFNGVKKDTLINLILDHQAA-TV---VV-ES-------VTEK-----TT-------EEKK---------PK--RARI--V----------P-----------------------EKK--AVVPKNEI----PLLFDSEEKKEIVEA-------------------------VIVQ 99

WP_035619884_1 MFDISALKEMKLSELQEIAKLAKTIKFNGVKKDTLISQILAHQEE-TA---QP-PV-------AQKV--NE-VV-------EDDK---------PK--RARI--V----------P-----------------------VKK--AAIQKNTVQKNLVFEEEKESK----TT--E-ILDTP-I------TPEAPEA--PVA 114

WP_073371274_1 MFDISVLKEMKLSELQEIAKAAKTIKFNGVKKEALISQILELQSA-SS---DADPK-------NKKE--EK-KA-------EDDK---------PK--RTRI--V----------A-----------------------EKK--PAIQKPVN----ASLF--AEE----TP--V-ETVAP-V------------------ 99

WP_091429370_1 MFDISALKEMKLAELQEIAKSAKTIKFNGVKKDTLIGAILDHQNS-TV---DS-TS-------EKQA--VN-NT-------EDDK---------PK--RARI--V----------P-----------------------VKK--AAIQKATT----KPLFSKKED----AV-DT-ELQAK-I------EVPAEVT----- 108

WP_074723071_1 MFDISALKEMKLSELQAIAKAAKTIKFNGVKKETLIGQILEQQTT-RV---EP-IL-------DLKI--ES-VV-------EDDK---------PK--RARI--V----------P-----------------------LKK--AAIQKAKV----IPT-NSEDT----LV-DT-DLQAK-I------DVPSEVR----- 107

WP_035671177_1 MFDISALKEMKLSELQEIAKSAKTIKFNGVKKDTLISQILEHQVATNV---DS-AL-------DSKK--ES-NV-------EDDK---------PK--RARI--V----------P-----------------------VKK--VAIQKVVK----NTLFEK-EE----TA--K-EISTP-V------EENIPSV----- 107

WP_099714848_1 MFDISALKEMKLSELQEIAKLAKTIKFNGVKKETLISQILEHQVV-NV---DS-AS-------DTKK--EN-NL-------DDDK---------PK--RVRI--V----------P-----------------------VKK--AAIQKAVP----NSLFEK-EE----VA--A-EINAP-V------ENNRP-I----- 105

WP_072941811_1 -----ASELIP-DAAAPETIT-PVEK----------KAAKVI--KF-NKSAY-E--KKIALKKD---KENEKEA-VAD---N---------------GGVSDKEIVVEST----IV-----------PTTEAI-V-PN--KKI--N-PH--------------DR------------------KR---------TDDANQ 202

WP_091525983_1 ---V-AEEVTT-AAPQNPSAE-KQRK---PQN----KNQNQR-----NKTNN-Q--PQVQAEPI---KAENDVV-----------------------AEIPAEEIV--------VV-----------AEKQIQ------------N-PN--------------QN----------------------------------- 167

WP_091096797_1 -----NSETKP-EAPSTETVE-KRNK---P-N----KNQKQY-----TKSTN-T--QEVATEVN---VDADKTL-----------------------SKEPMTDEA--------VA--------------ETE------------K-PA--------------QN----------------------------------- 156

WP_054406085_1 ----------------PVAPE-TPVA-------AP-ATAETA--ET-PKTDK----PRKEFKDK---KEQQPQP-QQQ---N------------------KQRQPL--------TD----EERR---AQIERA-N-AN-------N-PNHPSYKKRLAELQAAAA----------------------------ANGEPIP 199

WP_091315979_1 -------TQRA-RIKKPVVKS-SN------------RAQETL--SF-ELDAE----VKSESYSQ---QPKQVVE-EKK---EKNGNSDTNENTQNVVAEPSNEETR--------GEDESSEAKA---ETTEQE-R-NN-------QNPN----------F---------------------------------------- 180

WP_026992511_1 -P---KPRTAK-TIITPADST-APAP-EAKADIAD-AKPATE--RA-PQKDD----RRKILPAK---DSAQPRP-ADG---T---------------APARTRPQL--------TE----EERL---AQIERA-N-AN-------N-PNHPSYKKNL------AV----------------------------QNGQNTP 236

WP_073363513_1 -----VAEATP-PAAPAEETP-KSFV----------REGRENAPER-QRKPF-Q--RNIVTPNI---TGAPVDY-EPF-M-P---------------PQITTESLP-----SD-FM-----------EPLRSA-K-QE-VRKPMGT-PP--------------QK------SRPEFVPPGP--NVDIPI----AEPAYVS 226

WP_014388556_1 -------EPRK-RIKKSSEIS-YP------------DFQEEI--TF-DDEQI----KSTKVENK---AVVKPIS-EEL---P---------------TTPNSSDS---------------ESKT---EDLDNV-E-KQ-------N-PN----------F---------------------------------------- 165

WP_014166209_1 -E---DKKTSK-KVTNKKTST-HSEEVTEQKKEAFLKKPKNQ--SF-SKDKKKTSETEENITQSFSTEEVNVII-DPQ---K---------------DQTERTIEV--------IE----TTSEQP-NDSNTE-T-EQ-------T-QS--------------------------------------------------- 193

WP_072784887_1 -K---EVKEVK-E-----------------------KPKRPV--KN-TKQPE-L--PLEEPKLA---EKAPTES-KPT---E---------------ADNDKKLDA--------RQ-----------QQIIKA-N-QN-------N-PNHPSYKK--------------------------------------------- 159

WP_014084047_1 -E---EIKIIN-AEKTLFSKD-INPK----------PYIKPA--KF-KKAEY-E--KKLASKGN---KRALFNK-KTE---N---------------SEIENPNVS-ENDAI--VS-----------ESIVVT-V-AI-EEDS--K-PS--------------LE----------LENPNI--DNLENI-TA-VTEDPNK 227

WP_026715962_1 -E-----KAPT-T-----------------------KPVAKP--KF-TKETK-IA-PKQEAIDF---EENP----KPE---K---------------KAPQLTEEQ--------RK-----------AIIEKA-N-QN-------N-PNHPSYIG--------------------------------------------- 163

YP_001295636_1 -P---KAQTTP-ELEKPVVEA-IQKQ----------RAPKVI--RF-TKDEN----NVTKVDNS---HRNKKIV-KPV---I---------------VPANTEEGV--------SE----ILEN-P-ETVQKE-K-SS-------N-PN--------------------------------------------------- 179

WP_011962876_1 -P---KAQTTP-ELEKPVVEA-IQKQ----------RAPKVI--RF-TKDEN----NVTKVDNS---HRNKKIV-KPV---I---------------VPANTEEGV--------SE----ILEN-P-ETVQKE-K-SS-------N-PN--------------------------------------------------- 179

WP_034097990_1 -P---KAQTTP-ELEKPVVEA-IQKQ----------RAPKVI--RF-TKDEN----NVTKVDNS---HRNKKIV-KPV---I---------------VPANTEEGV--------SE----ILEN-P-ETVQKE-K-SS-------N-PN--------------------------------------------------- 179

WP_091477336_1 -A---KIETTP-ELEKPVVEA-IQKQ----------RPPKVI--RF-TKDEN----NVTKVDNS---HRNKKIV-KPV---I---------------APTTTDEVA--------SE----APEN-A-EPVQKE-K-PV-------N-PN--------------------------------------------------- 178

WP_035126216_1 --------VAQ-PTSVAEEVP-EVKE----------VSVKAE--KFQKKPKF-I--KPVKVASE---EQPSQEA-PQE---E---------------VATTETASE--------TT-----------EENSVK---PK-------N-PN--------------QN-------------PNQKHKN--------QNQNPNQ 178

WP_073312099_1 -E---EEKPTE-KIEKEPVKD-FKDN----------RNPKNN--RF-QKNNK-LKNPKANIDNE---NQAIPVL-ETE---A---------------EQVQTPLQE--------VE----VKTE---TESTEK-G-FK-------N-NN--------------------------------------------------- 172

WP_091464749_1 -------SQRA-RIKKGSTPE-KV------------AAQETL--SF-DDVVQ----VAQEVEIE---KTETPIS-ESV---E--------------------------------------EIKS---ETSDQD-K-SN-------Q--N----------F---------------------------------------- 142

WP_023574714_1 -K---EEVV-----EKPTTEA-SEPK----------RENFVS--K--KKEFV----KKPHFTKE---SNQNTKI-RPA---Q---------------NKEVEETAS--------VA----EVAE-PITEAPVA-K-PQ-------N-PN--------------------------------------------------- 176

WP_026712226_1 --------QPS-AESKKEEET-TFAP----------AKDKKL--PF-KKNKF-E--KKKNFEAQ---PTPV-----AA---E---------------NTESSSEAP--------IV-----------SEEGAA---EQ-------T-PS--------------K-----------------------------AQNNQPF 165

WP_091140744_1 --------ATE-PARPQANPA-PAFD----------NKQRKN--KF-KKGDF-D--RR-NKPEA---AEPRAEF-QPT-V-E---------------E-------P-----AA-AV-----------ESNQAE-T-PE--RKP---------------------------------------------------QSDHYK 156

WP_091398316_1 -A---PDVVAE-PKQENDNQQ-AKIE----------TVPKAI--KF-KKADY-E--KKIAPKKQ---QEPVARD-EVA---E---------------QPAAENTEP--------KQ-----------PEKKIN---PNQLHKL--A-KN--------------SNQSGNGNGNQNANNPNANNQNANNPNANNPNANGNQ 205

WP_066334474_1 -----ELDTNT-TLAPEKDNT-VPEN----------KPTKTV--KF-SKTAY-Q--NKIALKKS---KELATDT-TKE-------------------TEILANEIPVSKTL---VN-----------DAIATP---KA--KTT--N-AP--------------AV------------------KK----------ANPNQ 203

WP_023571963_1 -P---DAIA-----EKPAAEA--ETK----------REPFVN--K--KKEFV----QKTRFVKE---NNPNVAI-RPK---Q---------------NTETPEAAE--------TA----TA-----IETPVA-K-PQ-------N-PN--------------------------------------------------- 172

WP_026979818_1 ------STAEP-TAEKEAGKE-QKRE----------SFTKKK--EF-QQKQQ-------RFSKE-----NNPNF-QPR---L---------------QNTEIA------------------------EEPATE-K-AE-------I-PA--------------------------------------------------- 154

WP_024982133_1 -----PAAPEA-KVDQPKEGK-VPVA----------KTNKTV--KF-NKAAY-E--QKMALQRE---KEAAKLE-KPA-TNG---------------DADTITTNAKEVVAVD-ST-----------DAPEKP-Q-LI--KKI--N-PN--------------QL------------------KKQNQN----SNPNANQ 209

WP_026705806_1 -E---KEVPAK-EVTQETPVA-PEVP----------KERKVI--KF-NKAKY-E--NKINNAIQ---KEKVDN---II---E---------------KIPAEPTAE--------KV-----------ETANPA---ETKIQK---N-PN--------------QK----------------------------PNPNQSQ 183

WP_022828308_1 -------------NETTTEAE-VKIA----------PERKVI--KF-NKAKY-E--SKVNAAVE---RKSADEG-VKE---V---------------TATSENLES--------AT-----------SEEETK---PK-------K-PV--------------HA------------------QK--------ANPNSKI 167

WP_035657734_1 -------EVVA-KEELKAENE-AVAV----------EKPKAV--KF-NKATY-E--KKIALKKD---KENKDAG-TEN---P---------------VAAEGKQED--------AV-----------AEVVAP-AAPQ--KKI--N-PN--------------QQ------------------NKQNP-------NQQNQ 196

WP_089059798_1 -----VQVQEK-AESKTAENK-TAEK----------KAPKIV--KF-NKSAY-E--KKVALQKE---KEAAKEI-VSN---E---------------ETSETSETPVIAA----NT-----------EKSETP-A-PA--KKI--V-PN--------------QN------------------KN--------QNSNPNQ 199

WP_012026627_1 -----EEKQPA-EAEEVSEDK-TAEK----------KGPKVV--KF-NKSAY-E--KKVAQQKE---KEAVKEVISTE---E---------------TAENTTTAP--------VA-----------EKTENT-A-PV--KKI--N-PN--------------QN------------------KNQNPN----QNQNPNQ 195

WP_072972573_1 -----KIQPAP-KAHEAAESA-PVEK----------KGPKIV--KF-NKSAY-E--KKVALQKE---KEVTSEQ-TTE---I---------------EAETIAPAP--------VT-----------EKVEGA-A-PV--KKI--N-PN--------------QN------------------KNQNPN----QNPNQNP 206

WP_029273001_1 -----KIQAAP-KVQETAETA-PIEK----------KGPKIV--KF-NKSAY-E--KKVALQKE---KEAVKEV-STEEAAE---------------EAETAPPAP--------TA-----------EKIEQP-A-PV--KKP--N-PN--------------QN------------------KN--PN----QNPNQNP 201

WP_099708795_1 KEEIQKEEVTP-EVQETSENK-EIQK----------KGPKIV--KF-NKSAY-E--KKVALQKE---KEATKEV-GSE---E---------------VGETAPEAQITTEEKKEIT-----------EKTEVT-A-PV--KKI--N-PN--------------QN------------------KNQNPN----QNPNSNQ 223

WP_026713087_1 -----VNDVVA-GESDKKSEE-TAAA----------EKPKAV--KF-NKANY-E--KKVALKKN---KENKEAT-NDN---P---------------VAVETAQMD--------VV-----------SEVATP-A-PQ--KKI--N-PH--------------ER------------------KR--T-------DEVNQ 193

WP_035640151_1 EK---ETVVSS-EDNLVKEAE-TSVK----------KEGKAV--KF-SKSAY-E--KKMALQRE---KEALKEV-AVA---E---------------ETLSESNSE--------AV-----------HEKGEN---PVLEKKI--N-PQ--------------ER-----NRADDVTQPNKKGQK--------PNIKSNP 204

WP_035619884_1 -----ESKETV-EPVKTSEKI-VPEK----------KGSKII--KF-NKSAY-E--KKVALQKE---KEAAKEV-APE-AEI---------------SNENSAETQNETSTNT-TS-----------EKGEAP-V-QV--KKT--N-PN--------------QN------------------KQ-----------NTNQ 209

WP_073371274_1 --------VET-TNTEEKAPE-IAEK----------KVGKVI--KF-NKSAY-E--KKMALQKQ---KEEAKLA-SGE---N---------------ESTETNEVNSEAETPENTE-----------TTPEVS-A-PA--KKI--N-PN--------------QL------------------HK--------QNQNPNQ 193

WP_091429370_1 -S---QVEAVNTQTETPDEAP-VVEK----------KVGKVI--KF-NKSAY-E--KKIALKKD---KEEAKEV-SKE--------------------NEEATDTE--------TK-----------STQEVI-A-PV--KKV--N-PN--------------QL------------------NK--------------- 190

WP_074723071_1 -S---QVEAMN-ETKSLDESS-VPEK----------KVGKII--KF-NKSAY-E--KKIALKKD---KEEAKEL-NKE--------------------TEEVAAND--------AS-----------EMQEIV-A-PV--KKA--N-PN--------------QL------------------NK--------------- 188

WP_035671177_1 -E---PTPKQE-EVSPIVEQPVDGEK----------KVGKII--KF-NKSAY-E--KKIALKKD---KEEIKEA-TKE--------------------NVEATDVA--------TE-----------DKTEVPAA-PV--KKI--N-PN--------------QL------------------HK----------QNPNQ 195

WP_099714848_1 -E---AVSKEE-ESAPKPESE-GNEK----------KVGKII--KF-NKSAY-E--KKIALKKD---KEEVKEA-STE--------------------NVIVADTA--------TE-----------EKTEAPVA-PV--KKI--N-PN--------------QL------------------HK----------QNPNQ 192

WP_072941811_1 ---------LNK---------QNQ-NQ-------NQ-NQNP------------------NF--K------------NKK--N-N------FRDSDFEFDGIIESEGVLEMMPDG--YGFLRSSDYNYLASPDDIYLSTSQIRLFGLKTGDTVKGVVRPPKEGEKFFPLVRVLKINGHDPQVVRDRVSFEHLTPVFPSEKF 332

WP_091525983_1 ---------------------RNK-------------NQNP------------------NQ---------------KKQ----N------YRDPDYEFEGIIECEGVLEVPKDGGSHGYLRSSDYNYRKSPDDVYISQSQIRLFGLKKGDTIKGIVRPPKGNEQHFPLVRITKINGHEPDEVRDRPSFEHLTPLFPKDKF 290

WP_091096797_1 ---------------------QNR-------------NKNQ------------------NQ---------------KKQ----N------YRDPDYEFEGIIECEGVLEVPKDGGSHGYLRSSDYNYRKSPDDIYISQSQIRLFGLKKGDTIKGIVRPPKGNEQHFPLVRITKINGHEPDEVRDRPSFEHLTPLFPKDKF 279

WP_054406085_1 TELPKANVAKPGEQVDEPKAVREP-KEPREPKE----QREP------------------KE--QVEVKVQREPKAPKEI----N------YREPDYEFDGIIESEGVLEMMPDG--YGFLRSSDYNYLASPDDIYLSTSQIRLFGLKTGDTVKGVVRPPKEGEKYFPLVKVLKINGHDPQVVRDRVSFEHLTPLFPNEKF 362

WP_091315979_1 ---------------------KKNNNQNNNPKF----NKNN------------------GN--K-------------------N------FRESDFEFDGIIESEGVLEMMPDG--YGFLRSSDYNYLASPDDIYLSQSQVRLFGLKTGDTVKGVVRPPKEGEKFFPLVRVLKINGHDPQVIRDRISFEHLTPLFPEERF 308

WP_026992511_1 -NIP-------------PIISNKE-NVPSQPAA----AAQP------------------VQ--N------------KRQ----N------FREPDFEFDGIIESEGVLEMMPDG--YGFLRSSDYNYLASPDDIYLSNSQIRLFGLKTGDTVKGVVRPPKEGEKYFPLVKVNKINGHDPQVVRDRVSFEHLTPLFPEEKF 373

WP_073363513_1 ---------NAN---------GQG-NPNANGNT-NG-NGNS------------------SY--A------------NQN--N-KFKKTQNVRDADYEFDGIIDCEGVLEMMPDG--YGFLRSSDYNYLASPDDIYLSTSQIRLFGLKTGDTVKGVVRPPKESEKFFPLVKVLKINGHDPQVVRDRVSFDHLTPVFPQEKF 368

WP_014388556_1 ---------------------KKN-KFNKNPHN----NQNS------------------NNNQK-------------------N------YRDNDFEFDGIIETEGVLEMMPDG--YGFLRSSDYNYLASPDDIYLSQSQIRLFGLKTGDTVKGVVRPPKEGEKFFPLVKVLKINGYDPQVVRDRIAFEHLTPLFPEQKL 294

WP_014166209_1 ---------------------TEL-NKSKHSQN----NQNP------------------KY--K------------KTT----N------FREPDYEFEGIIESEGVLEMMPDG--YGFLRSSDYNYLASPDDIYLSTSQIRLFGLKTGDTVKGVVRPPKEGEKYFPLVKVLKINGHNPQVVRDRVSFDHLTPVFPTEKF 323

WP_072784887_1 ---------------------NNQ-AANN--------NGGG------------------NA--K--------P----------N------YREPDYEFDGIIESEGVLEMMPDG--YGFLRSSDYNYLASPDDIYLSQSQIRLFGLKTGDTVKGVVRPPKEGEKYFPLVKVLKINGFDPQVVRDRISFEHLTPIFPKEKF 283

WP_014084047_1 ---------PEE---------SNF-KKQHPPHP-KN-PKNP------------------KF--N------------NKK--N-N------FRDADYEFDGIIESEGVLEMMPDG--YGFLRSSDYNYLASPDDIYLSTSQIRLFGLKTGDTVKGVVRPPKEGEKFFPLIKVLKINGHEPQVVRDRVSFEHLTPVFPTEKF 363

WP_026715962_1 ---------------------PKA-TNGDTQPK-KEIKSTP------------------HK--K--------N----------N------YREPDYEFDGIIESEGVLEMMPDG--YGFLRSSDYNYLASPDDIYLSTSQIRLFGLKTGDTVKGVVRPPKEGEKFFPLVKVSKINGHDPQVIRDRISFEHLTPIFPKEKF 294

YP_001295636_1 ---------------------QNQ-KKPQERKRADEVNQNP------------------NA--K------------NKN----N------FREPDYEFEGIIESEGVLEMMPDG--YGFLRSSDYNYLASPDDIYLSTSQVRLFGLKTGDTVKGVVRPPKEGEKYFPLVRVLKINGHDPQVVRDRVSFEHLTPIFPTEKF 313

WP_011962876_1 ---------------------QNQ-KKPQERKRADEVNQNP------------------NA--K------------NKN----N------FREPDYEFEGIIESEGVLEMMPDG--YGFLRSSDYNYLASPDDIYLSTSQVRLFGLKTGDTVKGVVRPPKEGEKYFPLVRVLKINGHDPQVVRDRVSFEHLTPIFPTEKF 313

WP_034097990_1 ---------------------QNQ-KKPQERKRTDEVNQNP------------------NA--K------------NKN----N------FREPDYEFEGIIESEGVLEMMPDG--YGFLRSSDYNYLASPDDIYLSTSQVRLFGLKTGDTVKGVVRPPKEGEKYFPLVRVLKINGHDPQVVRDRVSFEHLTPIFPTEKF 313

WP_091477336_1 ---------------------PNQ-KNPNQNP-----NQNP------------------NA--K------------HKN----N------FREPDYEFEGIIESEGVLEMMPDG--YGFLRSSDYNYLASPDDIYLSTSQVRLFGLKTGDTVKGVVRPPKEGEKYFPLVRVLKINGHDPQVVRDRVSFEHLTPIFPTEKF 307

WP_035126216_1 ---------NPN---------QNP-NQNGNGNG-NP-NQNP-NQNPNQK-----------H--K------------NQK--SNN------FRDADYEFDGIIESEGVLEMMPDG--YGFLRSSDYNYLASPDDIYLSTSQIRLFGLKTGDTVKGVVRPPKEGEKFFPLVRVLKINGHDPQVVRDRVSFEHLTPIFPQEKF 321

WP_073312099_1 ---------------------PNQ-NPNPNQNQ----NPNQ------------------KH--K------------KTT----N------FREPDYEFEGIIESEGVLEMMPDG--YGFLRSSDYNYLASPDDIYLSTSQIRLFGLKTGDTVKGVVRPPKEGEKYFPLVKVLKINGHDPQVVRDRVSFEHLTPVFPTEKF 302

WP_091464749_1 ---------------------KKN---TNNPKF----NKNQ-------------------N--K-------------------N------FRESDFEFDGIIESEGVLEMMPDG--YGFLRSSDYNYLASPDDIYLSQSQIRLFGLKTGDTVKGVVRPPKEGEKFFPLVKVLKINGHDPQVIRDRIAFEHLTPLFPEEKF 266

WP_023574714_1 ---------------------QGG-ANQNTPNQ----NQNP------------------SQ--K------------HKKQ---N------FREPEYEFDGIIESEGVLEMMPDG--YGFLRSSDYNYLASPDDIYLSTSQIRLFGLKTGDTVKGVVRPPKEGEKYFPLVKVLKINGHDPQVVRDRISFEHLTPVFPTEKF 307

WP_026712226_1 ---------AKQ---------NNG-NGNGNGNG-NG-HHNN---------------P--NF--K------------HKK--Q-N------YRDADYEFDGIIESEGVLEMMADG--YGFLRSSDYNYLASPDDIYLSTSQIRLFGLKTGDTVKGVVRPPKEGEKFFPLVKVLKINGHDPQVVRDRVSFEHLTPIFPEEKF 302

WP_091140744_1 ---------NRN---------AQG-H--ANANQ-NG-NGNP------------------NY--K------------NKK--N-N----NNFRDADYEFDGIIESEGVLEMMPDG--YGFLRSSDYNYLASPDDIYLSTSQIRLFGLKTGDTVKGVVRPPKEGEKFFPLVRVLKINGHDPQIVRDRVSFEHLTPVFPQEKF 292

WP_091398316_1 ---------AAN---------GNQ-AANGNQAA-NG-NQNP-NQNPNQNGPNGNVNP--NF--K------------NKK--SNN------FRDADFEFDGIIESEGVLEMMPDG--YGFLRSSDYNYLASPDDIYLSVSQIKLFGLKTGDTVKGVVRPPKEGEKFFPLVRVLKINGHDPQVVRDRVSFEHLTPVFPSEKF 357

WP_066334474_1 ---------TNK---------QNP-NQ-------NP-NQNP------------------NF--K------------NKK--N-N------FRDADFEFDGIIESEGVLEMMADG--YGFLRSSDYNYLASPDDIYLSTSQIRLFGLKTGDTVKGVVRPPKEGEKFFPLVRVLKINGHDPQVVRDRVSFEHLTPVFPSEKF 333

WP_023571963_1 ---------------------QNQ-NQNQNPNQ----NQNP------------------NQ--K------------HKKQ---N------FRESDYEFDGIIESEGVLEMMPDG--YGFLRSSDYNYLASPDDIYLSTSQIRLFGLKTGDTVKGVVRPPKEGEKYFPLVKVLKINGHDPQVVRDRISFEHLTPVFPTEKF 303

WP_026979818_1 ---------------------PVQ-NQQNQNP-----NQNP------------------NQ--K------------NKKQ---N------FREPDYEFDGIIESEGVLEMMPDG--YGFLRSSDYNYLASPDDIYLSQSQIRLFGLKTGDTVKGVVRPPKEGEKYFPLVKVLKINGHDPQVVRDRISFEHLTPLFPEEKF 284

WP_024982133_1 ---------NPN---------ANT-NQNGNA---AG-NQNP------------------NF--K------------NKK--N-N------FKDADFEFDGIIESEGVLEMMPDG--YGFLRSSDYNYLASPDDIYLSTSQIRLFGLKTGDTVKGVVRPPKEGEKYFPLVRVLKINGHDPQVVRDRVSFEHLTPVFPSEKF 343

WP_026705806_1 ---------KPN---------PNQ-NQNQNQNQ-NG-NQTP-NGNQN---------P--NF--K------------GKK--N-N------FRDADFEFDGIIESEGVLEMMPDG--YGFLRSSDYNYLASPDDIYLSNSQVRLFGLKTGDTVKGVVRPPKEGEKFFPLVRVLKINGHDPQVVRDRISFEHLTPVFPTEKF 325

WP_022828308_1 ---------NPS---------QNP-NQNSNGNG-NA-PQNP-SQ-------------------K------------NKK--N-N------YRDSDFEFDGIIESEGVLEMMPDG--YGFLRSSDYNYLASPDDIYLSNSQVRLFGLKTGDTVKGVVRPPKEGEKFFPLVRVLKINGHDPQVVRDRVSFEHLTPVFPDEKF 303

WP_035657734_1 ---------NSN---------QNG-NQNP-----NQ-NQNP------------------NF--K------------NKK-NN-N------FRDSDFEFDGIIESEGVLEMMPDG--YGFLRSSDYNYLASPDDIYLSTSQIRLFGLKTGDTVKGVVRPPKEGEKFFPLVRVLKINGHDPQVVRDRISFEHLTPIFPSEKF 329

WP_089059798_1 ---------SPA---------QNP-NQSGNGNGNNG-NQNPNQN------------PNQNH--K------------NKK--N-N------FRDSDFEFDGIIESEGVLEMMPDG--YGFLRSSDYNYLASPDDIYLSTSQIRLFGLKTGDTVKGVVRPPKEGEKFFPLVRVLKINGHDPQVVRDRVSFEHLTPVFPSEKF 342

WP_012026627_1 ---------NQN---------QNP-NQNGNGNGNNG-NQNQ------------------NH--K------------NKKNNN-N------FRDSDFEFDGIIESEGVLEMMPDG--YGFLRSSDYNYLASPDDIYLSTSQIRLFGLKTGDTVKGVVRPPKEGEKFFPLVRVLKINGHDPQVVRDRVSFEHLTPVFPSEKF 334

WP_072972573_1 ---------NQN---------TNG-N--GNGNGNNG-NQNP------------------NH--K------------NKK--N-N------FRDSDFEFDGIIESEGVLEMMPDG--YGFLRSSDYNYLASPDDIYLSTSQIRLFGLKTGDTVKGVVRPPKEGEKFFPLVRVLKINGHDPQVVRDRVSFEHLTPVFPSEKF 341

WP_029273001_1 ---------NQN---------GNG-NVNGNGNGSNG-NQNP------------------NH--K------------NKK--N-N------FRDSDFEFDGIIESEGVLEMMPDG--YGFLRSSDYNYLASPDDIYLSTSQIRLFGLKTGDTVKGVVRPPKEGEKFFPLVRVLKINGHDPQVVRDRVSFEHLTPVFPSEKF 338

WP_099708795_1 ---------NPN---------QNP-NQNGNGNGNNG-NQNP------------------NH--K------------NKK--N-N------FRDSDFEFDGIIESEGVLEMMPDG--YGFLRSSDYNYLASPDDIYLSTSQIRLFGLKTGDTVKGVVRPPKEGEKFFPLVRVLKINGHDPQVVRDRVSFEHLTPVFPSEKF 360

WP_026713087_1 ---------NKQ---------NQN-QNNP-----NQ-NQNQ------------------NH--K------------NKK-NT-N------FRDADFEFDGIIESEGVLEMMPDG--YGFLRSSDYNYLASPDDIYLSTSQIRLFGLKTGDTVKGVVRPPKEGEKFFPLVRVLKINGHDPQVVRDRISFEHLTPVFPSEKF 326

WP_035640151_1 ---------NQN---------GNS-NQNGNSNQ-NS-NQNQ-NQNQNQN-------P--NF--K------------NKK--S-N------FKDADFEFDGIIESEGVLEMMPDG--YGFLRSSDYNYLASPDDIYLSTSQVRLFGLKTGDTVKGVVRPPKEGEKFFPLVRVLKINGHDPQVVRDRVSFEHLTPVFPSEKF 348

WP_035619884_1 ---------NPN---------QNP-NQNPNQNGNNG-NQNP------------------NF--K------------NKK--N-N------FRDSDFEFDGIIESEGVLEMMPDG--YGFLRSSDYNYLASPDDIYLSTSQIRLFGLKTGDTVKGVVRPPKEGEKFFPLVRVLKINGHDPQVVRDRVSFEHLTPVFPSEKF 346

WP_073371274_1 ---------NPH---------ERK-RTDEVNQNGNG-NVNP------------------NF--K------------NKK--N-N------FRDSDFEFDGIIESEGVLEMMPDG--YGFLRSSDYNYLASPDDIYLSTSQIRLFGLKTGDTVKGVVRPPKEGEKFFPLVRVLKINGHDPQVVRDRVSFEHLTPVFPSEKF 330

WP_091429370_1 -----------------------------------Q-NQNP------------------TF--K------------AKK--S-N------FRDSDFEFDGIIESEGVLEMMADG--YGFLRSSDYNYLASPDDIYLSTSQIRLFGLKTGDTVKGVVRPPKEGEKFFPLVKVLKINGHDPQVVRDRVSFEHLTPVFPSEKF 311

WP_074723071_1 -----------------------------------Q-NQNP------------------NF--K------------SKK--S-N------FRDSDFEFDGIIESEGVLEMMADG--YGFLRSSDYNYLASPDDIYLSTSQIRLFGLKTGDTVKGVVRPPKEGEKFFPLVKVLKINGHDPQVVRDRVSFEHLTPVFPSEKF 309

WP_035671177_1 ---------NSN---------QNP-NQN-------G-NQNP------------------NF--K------------NKK--S-N------FAASDFEFDGIIESEGVLEMMPDG--YGFLRSSDYNYLASPDDIYLSTSQIRLFGLKTGDTVKGVVRPPKEGEKFFPLVRVLKINGHDPQVVRDRVSFEHLTPVFPSEKF 325

WP_099714848_1 ---------NPN---------QNP-NQNGNG---NG-NQNP------------------NF--K------------NKK--S-N------FAASDFEFDGIIESEGVLEMMPDG--YGFLRSSDYNYLASPDDIYLSTSQIRLFGLKTGDTVKGVVRPPKEGEKFFPLVRVLKINGHDPQVVRDRVSFEHLTPVFPSEKF 326

WP_072941811_1 KLAEKQSTISTRIIDLFSPIGKGQRGMIVAQPKTGKTMLLKEIANAIAANHPEVYLIVLLIDERPEEVTDMQRSVRGEVIASTFDREPQEHVKIANIVLEKAKRLVECGHDVVILLDSITRLARAYNTVQPASGKVLSGGVDANALQKPKRFFGAARNVENGGSLSIIATALTETGSKMDEVIFEEFKGTGNMELQLDRK 532

WP_091525983_1 NLSGNNSKLSTRIIDLFAPIGKGQRGMIVAQPKTGKTMMLKEIANAIADNHPEVYQIVLLIDERPEEVTDMQRNVRAEVVASTFDEEARYHVELADMVLEKAKRLVECGHDVVILLDSITRLARAYNTVQPASGRVLSGGVDANALQKPKRFFGAARNIENGGSLTIIATALTDTGSKMDDVIFEEFKGTGNMELQLDRK 490

WP_091096797_1 NLSGNNSRLSTRIIDLFAPIGKGQRGMIVAQPKTGKTMMLKEIANAIADNHPEVYQIVLLIDERPEEVTDMQRNVRAEVVASTFDEEARYHVELADMVLEKAKRLVECGHDVVILLDSITRLARAYNTVQPASGRVLSGGVDANALQKPKRFFGAARNIENGGSLTIIATALTDTGSKMDDVIFEEFKGTGNMELQLDRK 479

WP_054406085_1 NLADKHAPISTRIIDLFSPIGKGQRGMIVAQPKTGKTMLLKDIANSIAANHPEVYLIVLLIDERPEEVTDMQRSVRGEVIASTFDEPAERHVKVANIVLEKAKRLVECGHDVVILLDSITRLARAYNTVQPASGKVLSGGVDANALQKPKRFFGAARNIEGGGSLSIIATALVDTGSKMDEVIFEEFKGTGNMELQLDRK 562

WP_091315979_1 NLSGKNTSISTRIIDMFSPIGKGQRAMIVAQPKTGKTMLLKDIANSIAANHPEVYLIVLLIDERPEEVTDMQRSVRAEVIASTFDEPADRHVKVANIVLEKAKRLVECGHDVVILLDSITRLARAYNTVQPASGKVLSGGVDANALQKPKRFFGAARNIENGGSLSIIATALTETGSKMDEVIFEEFKGTGNMELQLDRK 508

WP_026992511_1 NLADKQSTISTRIIDLFSPIGKGQRGMIVAQPKTGKTMLLKDIANSIAANHPEVYLIVLLIDERPEEVTDMQRSVRGEVIASTFDEPAERHVKVANIVLEKAKRLVECGHDVVILLDSITRLARAYNTVQPASGKVLSGGVDANALQKPKRFFGAARNIEGGGSLSIIATALTETGSKMDEVIFEEFKGTGNMELQLDRK 573

WP_073363513_1 KMDAKQASVSTRIIDLFSPIGKGQRGMIVAQPKTGKTMLLKDIANAIAANHPEVYLLVLLIDERPEEVTDMQRSVRGEVIASTFDREPQEHVKIANIVLEKAKRLVECGHDVVILLDSITRLARAYNTVQPASGKVLSGGVDANALQKPKRFFGAARNVENGGSLSIIATALTETGSKMDEVIFEEFKGTGNMELQLDRK 568

WP_014388556_1 NLADRSKSISTRIIDLFAPIGKGQRAMIVAQPKTGKTMLLKDIANSIAANHPEVYMIVLLIDERPEEVTDMQRSVRAEVIASTFDEPAERHVKVANIVLEKAKRLVECGHDVVILLDSITRLARAYNTVQPASGKVLSGGVDANALQKPKRFFGAARNIENGGSLSIIATALTETGSKMDEVIFEEFKGTGNMELQLDRK 494

WP_014166209_1 KLAEKGSSISTRIIDLFSPIGKGQRGMIVAQPKTGKTMLLKDIANAIAANHPEVYMIVLLIDERPEEVTDMQRSVRAEVVASTFDREPQEHVKIANIVLEKAKRLVECGHDVVILLDSITRLARAYNTVQPASGKVLSGGVDANALQKPKRFFGAARNVENGGSLSIIATALTETGSKMDEVIFEEFKGTGNMELQLDRK 523

WP_072784887_1 NLAERSSTISTRVIDLFSPIGKGQRGMIVAQPKTGKTMLLKDVANAIAANHPEVYMIVLLIDERPEEVTDMQRSVKAEVVASTFDREPQEHVKIANIVLEKAKRLTECGHDVVILLDSITRLARAYNTVQPASGKVLSGGVDANALQKPKRFFGAARNVENGGSLSIIATALTETGSKMDEVIFEEFKGTGNMELQLDRR 483

WP_014084047_1 KLAEMGSSVSTRIIDLFSPIGKGQRGMIVAQPKTGKTMLLKDIANAIAANHPEVYLLVLLIDERPEEVTDMIRNVKGEVIASTFDREPQEHVKIANIVLEKAKRLVECGHDVVVLLDSITRLARAYNTVQPASGKVLSGGVDANALQKPKRFFGAARNIENGGSLSIIATALTETGSKMDEVIFEEFKGTGNMELQLDRK 563

WP_026715962_1 NLAERGSTISTRILDLFAPIGKGQRGMIVAQPKTGKTMLLKEIANAIATNHPEVYLIVLLIDERPEEVTDMQRSVKGEVIASTFDREPQEHVKIANIVLEKAKRLTECGHDVVILLDSITRLARAYNTVQPASGKVLSGGVDANALQKPKRFFGAARNVENGGSLSIIATALTDTGSKMDEVIFEEFKGTGNMELQLDRR 494

YP_001295636_1 RLAEKGSSISTRIIDLFSPIGKGQRGMIVAQPKTGKTMLLKEIANAIAANHPEVYLIVLLIDERPEEVTDMQRSVRGEVVASTFDREPQEHVKIANIVLEKAKRLVECGHDVVILLDSITRLARAYNTVQPASGKVLSGGVDANALQKPKRFFGAARNVENGGSLSIIATALTETGSKMDEVIFEEFKGTGNMELQLDRK 513

WP_011962876_1 RLAEKGSSISTRIIDLFSPIGKGQRGMIVAQPKTGKTMLLKEIANAIAANHPEVYLIVLLIDERPEEVTDMQRSVRGEVVASTFDREPQEHVKIANIVLEKAKRLVECGHDVVILLDSITRLARAYNTVQPASGKVLSGGVDANALQKPKRFFGAARNVENGGSLSIIATALTETGSKMDEVIFEEFKGTGNMELQLDRK 513

WP_034097990_1 RLAEKGSSISTRIIDLFSPIGKGQRGMIVAQPKTGKTMLLKEIANAIAANHPEVYLIVLLIDERPEEVTDMQRSVRGEVVASTFDREPQEHVKIANIVLEKAKRLVECGHDVVILLDSITRLARAYNTVQPASGKVLSGGVDANALQKPKRFFGAARNVENGGSLSIIATALTETGSKMDEVIFEEFKGTGNMELQLDRK 513

WP_091477336_1 RLAEKGSSISTRIIDLFSPIGKGQRGMIVAQPKTGKTMLLKEIANAIAANHPEVYLIVLLIDERPEEVTDMQRSVRGEVVASTFDREPQEHVKIANIVLEKAKRLVECGHDVVILLDSITRLARAYNTVQPASGKVLSGGVDANALQKPKRFFGAARNVENGGSLSIIATALTETGSKMDEVIFEEFKGTGNMELQLDRK 507

WP_035126216_1 NIAGKQASTSTRIIDLFSPIGKGQRGMIVAQPKTGKTMLLKDIANAIAANHPEVYLIVLLIDERPEEVTDMQRSVRGEVIASTFDREPMEHVKIANIVLEKSKRLVECGHDVVILLDSITRLARAYNTVQPASGKVLSGGVDANALQKPKRFFGAARNVENGGSLSIIATALTETGSKMDEVIFEEFKGTGNMELQLDRK 521

WP_073312099_1 KLAEKGSSISTRIIDLFSPIGKGQRGMIVAQPKTGKTMLLKDIANAIAANHPEVYMIVLLIDERPEEVTDMQRSVRAEVVASTFDREPQEHVKIANIVLEKAKRLVECGHDVVILLDSITRLARAYNTVQPASGKVLSGGVDANALQKPKRFFGAARNVENGGSLSIIATALTETGSKMDEVIFEEFKGTGNMELQLDRK 502

WP_091464749_1 NLADKNSSISTRIIDLFSPIGKGQRAMIVAQPKTGKTMLLKDIANSIAANHPEAYLIVLLIDERPEEVTDMQRSVRGEVIASTFDEPADRHVKVANIVLEKAKRLVECGHDVVILLDSITRLARAYNTVQPASGKVLSGGVDANALQKPKRFFGAARNIENGGSLSIIATALTETGSKMDEVIFEEFKGTGNMELQLDRK 466

WP_023574714_1 RLAERQSTISTRVIDLFSPIGKGQRGMIVAQPKTGKTMLLKDIANAIAANHPEVYLIVLLIDERPEEVTDMQRSVRGEVIASTFDREPQEHVKIANIVLEKAKRLVECGHDVVVLLDSITRLARAYNTVQPASGKVLSGGVDANALQKPKRFFGAARNVENGGSLSIIATALTETGSKMDEVIFEEFKGTGNMELQLDRK 507

WP_026712226_1 KLAEKQSSVSTRIIDLFSPIGKGQRGMIVAQPKTGKTVLLKDIANSIAANHPEVYLIVLLIDERPEEVTDMQRNVRGEVIASTFDEPADRHVKVANIVLEKAKRLVECGHDVVILLDSITRLARAYNTVQPASGKVLSGGVDANALQKPKRFFGAARNIENGGSLSIIATALTDTGSKMDEVIFEEFKGTGNMELQLDRK 502

WP_091140744_1 RIAEKGSSISTRIIDLFSPIGKGQRGMIVAQPKTGKTMLLKDIANAIAANHPEVYLLVLLIDERPEEVTDMQRSVRGEVIASTFDREPQEHVKIANIVLEKAKRLVECGHDVVILLDSITRLARAYNTVQPASGKVLSGGVDANALQKPKRFFGAARNVENGGSLSIIATALTETGSKMDEVIFEEFKGTGNMELQLDRK 492

WP_091398316_1 RLAEKQSTISTRIIDLFSPIGKGQRGMIVAQPKTGKTMLLKDIANAIAANHPEVYLLVLLIDERPEEVTDMQRSVRGEVIASTFDREPQEHVKIANIVLEKAKRLVECGHDVVILLDSITRLARAYNTVQPASGKVLSGGVDANALQKPKRFFGAARNVENGGSLSIIATALTETGSKMDEVIFEEFKGTGNMELQLDRK 557

WP_066334474_1 NLADRQSTISTRIIDLFSPLGKGQRGMIVAQPKTGKTMLLKEIANAIAANHPEVYLLVLLIDERPEEVTDMQRSVRGEVIASTFDREPQEHVKIANIVLEKAKRLVECGHDVVILLDSITRLARAYNTVQPASGKVLSGGVDANALQKPKRFFGAARNVENGGSLSIIATALTETGSKMDEVIFEEFKGTGNMELQLDRK 533

WP_023571963_1 RLAEKQSTISTRIIDLFSPIGKGQRGMIVAQPKTGKTMLLKDIANAIAANHPEVYLIVLLIDERPEEVTDMQRSVRGEVIASTFDREPQEHVKIANIVLEKSKRLVECGHDVVILLDSITRLARAYNTVQPASGKVLSGGVDANALQKPKRFFGAARNVENGGSLSIIATALTETGSKMDEVIFEEFKGTGNMELQLDRK 503

WP_026979818_1 RLAERQSTISTRIIDLFSPIGKGQRGMIVAQPKTGKTMLLKDIANTIAANHPEVYLIVLLIDERPEEVTDMQRSVRGEVIASTFDEPAERHVKVANIVLEKAKRLVECGHDVVILLDSITRLARAYNTVQPASGKVLSGGVDANALQKPKRFFGAARNIENGGSLSIIATALTETGSKMDEVIFEEFKGTGNMELQLDRK 484

WP_024982133_1 KLAEKQSTISTRIMDLFSPIGKGQRGMVVAQPKTGKTMLLKDIANAIAANHPEVYLIVLLIDERPEEVTDMQRSVRGEVIASTFDREPQEHVKIANIVLEKAKRLVECGHDVVILLDSITRLARAYNTVQPASGKVLSGGVDANALQKPKRFFGAARNVENGGSLSIIATALTETGSKMDEVIFEEFKGTGNMELQLDRK 543

WP_026705806_1 RIAEKQSNISTRIIDLFSPIGKGQRGMIVAQPKTGKTMLLKDIANAIAANHPEVYLIVLLIDERPEEVTDMQRSVRGEVIASTFDREPQEHVKIANIVLEKAKRLVECGHDVVILLDSITRLARAYNTVQPASGKVLSGGVDANALQKPKRFFGAARNVENGGSLSIIATALTETGSKMDEVIFEEFKGTGNMELQLDRK 525

WP_022828308_1 RLAEKESTISTRIIDLFSPIGKGQRGMIVAQPKTGKTMLLKDIANAIAANHPEVYLIVLLIDERPEEVTDMQRSVRGEVVASTFDREPQEHVKIANIVLEKAKRLVECGHDVVILLDSITRLARAYNTVQPASGKVLSGGVDANALQKPKRFFGAARNVENGGSLSIIATALTETGSKMDEVIFEEFKGTGNMELQLDRK 503

WP_035657734_1 KLAERDSSISTRIIDLFSPIGKGQRGMIVAQPKTGKTVLLKEIANAIAANHPEVYLIVLLIDERPEEVTDMQRSVRGEVIASTFDREPQEHVKIANIVLEKSKRLVECGHDVVILLDSITRLARAYNTVQPASGKVLSGGVDANALQKPKRFFGAARNVEKGGSLSIIATALTETGSKMDEVIFEEFKGTGNMELQLDRK 529

WP_089059798_1 KLAEKGSSVSTRIIDLFSPIGKGQRGMIVAQPKTGKTMLLKDIANAIAANHPEVYLIVLLIDERPEEVTDMQRSVRGEVIASTFDREPQEHVKIANIVLEKAKRLVECGHDVVILLDSITRLARAYNTVQPASGKVLSGGVDANALQKPKRFFGAARNVENGGSLSIIATALTETGSKMDEVIFEEFKGTGNMELQLDRK 542

WP_012026627_1 KLAEKGSSISTRIIDLFSPIGKGQRGMIVAQPKTGKTMLLKDIANAIAANHPEVYLIVLLIDERPEEVTDMQRSVRGEVIASTFDREPQEHVKIANIVLEKSKRLVECGHDVVILLDSITRLARAYNTVQPASGKVLSGGVDANALQKPKRFFGAARNVENGGSLSIIATALTETGSKMDEVIFEEFKGTGNMELQLDRK 534

WP_072972573_1 KLAEKGSSVSTRIIDLFSPIGKGQRGMIVAQPKTGKTMLLKDIANAIAANHPEVYLIVLLIDERPEEVTDMQRSVRGEVIASTFDREPQEHVKIANIVLEKAKRLVECGHDVVILLDSITRLARAYNTVQPASGKVLSGGVDANALQKPKRFFGAARNVENGGSLSIIATALTETGSKMDEVIFEEFKGTGNMELQLDRK 541

WP_029273001_1 KLAEKGSSVSTRIIDLFSPIGKGQRGMIVAQPKTGKTMLLKDIANAIAANHPEVYLIVLLIDERPEEVTDMQRSVRGEVIASTFDREPQEHVKIANIVLEKAKRLVECGHDVVILLDSITRLARAYNTVQPASGKVLSGGVDANALQKPKRFFGAARNVENGGSLSIIATALTETGSKMDEVIFEEFKGTGNMELQLDRK 538

WP_099708795_1 KLAEKGSSVSTRIIDLFSPIGKGQRGMIVAQPKTGKTMLLKDIANAIAANHPEVYLIVLLIDERPEEVTDMQRSVRGEVIASTFDREPQEHVKIANIVLEKAKRLVECGHDVVILLDSITRLARAYNTVQPASGKVLSGGVDANALQKPKRFFGAARNVENGGSLSIIATALTETGSKMDEVIFEEFKGTGNMELQLDRK 560

WP_026713087_1 KLAERESTISTRIIDLFSPIGKGQRGMIVAQPKTGKTMLLKEIANAIAANHPEVYLIVLLIDERPEEVTDMQRSVRGEVIASTFDREPQEHVKIANIVLEKSKRLVECGHDVVILLDSITRLARAYNTVQPASGKVLSGGVDANALQKPKRFFGAARNVEKGGSLSIIATALTETGSKMDEVIFEEFKGTGNMELQLDRK 526

WP_035640151_1 RLAEKQSTISTRIIDLFSPIGKGQRGMIVAQPKTGKTMLLKEIANAIAANHPEIYLIVLLIDERPEEVTDMQRSVRGEVIASTFDREPQEHVKIANIVLEKAKRLVECGHDVVVLLDSITRLARAYNTVQPASGKVLSGGVDANALQKPKRFFGAARNVENGGSLSIIATALTETGSKMDEVIFEEFKGTGNMELQLDRK 548

WP_035619884_1 KLAEKQSTISTRIIDLFSPIGKGQRGMIVAQPKTGKTMLLKDIANAIAANHPEVYLIVLLIDERPEEVTDMQRSVRGEVIASTFDREPQEHVKIANIVLEKAKRLVECGHDVVILLDSITRLARAYNTVQPASGKVLSGGVDANALQKPKRFFGAARNVENGGSLSIIATALTETGSKMDEVIFEEFKGTGNMELQLDRK 546

WP_073371274_1 KLAERQSTISTRIIDLFSPIGKGQRGMIVAQPKTGKTMLLKDIANAIAANHPEVYLIVLLIDERPEEVTDMQRSVRGEVIASTFDREPQEHVKIANIVLEKAKRLVECGHDVVILLDSITRLARAYNTVQPASGKVLSGGVDANALQKPKRFFGAARNVENGGSLSIIATALTETGSKMDEVIFEEFKGTGNMELQLDRK 530

WP_091429370_1 NLAARESTISTRIIDLFSPIGKGQRGMIVAQPKTGKTMLLKEIANAIAANHPEVYLIVLLIDERPEEVTDMQRSVRGEVIASTFDREPQEHVKIANIVLEKAKRLVECGHDVVILLDSITRLARAYNTVQPASGKVLSGGVDANALQKPKRFFGAARNVENGGSLSIIATALTETGSKMDEVIFEEFKGTGNMELQLDRK 511

WP_074723071_1 NLAERQSTISTRIIDLFSPIGKGQRGMIVAQPKTGKTMLLKEIANAIAANHPEVYLIVLLIDERPEEVTDMQRSVRGEVIASTFDREPQEHVKIANIVLEKAKRLVECGHDVVILLDSITRLARAYNTVQPASGKVLSGGVDANALQKPKRFFGAARNVENGGSLSIIATALTETGSKMDEVIFEEFKGTGNMELQLDRK 509

WP_035671177_1 KLAEKQSTISTRIIDLFSPIGKGQRGMIVAQPKTGKTMLLKEIANAIAANHPEVYLIVLLIDERPEEVTDMQRSVRGEVIASTFDREPQEHVKIANIVLEKAKRLVECGHDVVILLDSITRLARAYNTVQPASGKVLSGGVDANALQKPKRFFGAARNVENGGSLSIIATALTETGSKMDEVIFEEFKGTGNMELQLDRK 525

WP_099714848_1 KLAEKQSTISTRIIDLFSPIGKGQRGMIVAQPKTGKTMLLKEIANAIAANHPEVYLIVLLIDERPEEVTDMQRSVRGEVIASTFDREPQEHVKIANIVLEKAKRLVECGHDVVILLDSITRLARAYNTVQPASGKVLSGGVDANALQKPKRFFGAARNVENGGSLSIIATALTETGSKMDEVIFEEFKGTGNMELQLDRK 526

WP_072941811_1 IANKRIFPAIDLTSSSTRRDDMLLDQKTLQRMWIMRKYLSDMNPVEAMDFINDRFKKTRNNDEFLIS-MND- 602

WP_091525983_1 IANKRIFPAVDLTSSSTRRDDLLQDRFTNSKMVVLRNILADMNPVEAITFIHDRIKNTKSNEEFFDS-MRD- 560

WP_091096797_1 IANKRIFPAVDLTSSSTRRDDLLQDRFTNSKMLVLRNILADMNPVEAITFIHDRIKNTKSNEEFFDS-MRD- 549

WP_054406085_1 IANRRIFPAIDLTSSSTRRDDLLQDEKTIQRMWIMRKYLADMNPVEAMDFINDRFKKTRNNEEFLIS-MND- 632

WP_091315979_1 ISNKRIFPAIDLVSSSTRRDDLLLDKSTLQHMWILRKYLADMNPVEAMEFIESRIKRTKNNEEFLIS-MND- 578

WP_026992511_1 IANRRIFPAIDLTSSSTRRDDLLLDEKTIQRMWIMRKYLADMNPVEAMDFINDRFRKTRNNEDFLLS-MND- 643

WP_073363513_1 IANRRIFPAIDLTSSSTRRDDLLLDEKTLQRMWILRKFLADMNPVEAMDTINDKIKKTRTNDEFLIS-MND- 638

WP_014388556_1 IANKRIFPAIDLVSSSTRRDDLLLDEKTIQRMWILRKYLADMNPVEAMEFIESRIKGTRNNEEFLIS-MND- 564

WP_014166209_1 IANRRIFPSVDLTSSSTRRDDLLLDEKTIQRMWIMRKYLADMNSVEAMEFINDRFKKTRNNEEFLIS-MND- 593

WP_072784887_1 IANKRIFPAIDLVSSSTRRDDLLLDEKTIQRMWIMRKYLADMNPVEAMDFIFDRFKKTRNNEEFLIS-MNE- 553

WP_014084047_1 IANKRIFPAIDLTSSSTRRDDLLLDTQTLQRMWIMRKYLADMNPVEAMDFINERFKGTRNNEEFLISMMND- 634

WP_026715962_1 IANKRIFPAIDLVSSSTRRDDLLLDEQTIQRMWIMRKYLADMNPVEAMDFINDRFKKTKNNEEFLIS-MNN- 564

YP_001295636_1 IANRRIFPAIDLTSSSTRRDDLLLDEKTIQRMWIMRKYLADMNPVEAMEFINDRFKKTRNNEEFLIS-MND- 583

WP_011962876_1 IANRRIFPAIDLTSSSTRRDDLLLDEKTIQRMWIMRKYLADMNPVEAMEFINDRFKKTRNNEEFLIS-MND- 583

WP_034097990_1 IANRRIFPAIDLTSSSTRRDDLLLDEKTIQRMWIMRKYLADMNPVEAMEFINDRFKKTRNNEEFLIS-MND- 583

WP_091477336_1 IANRRIFPAIDLTSSSTRRDDLLLDEKTIQRMWIMRKYLADMNPVEAMEFINDRFKKTRNNEEFLIS-MND- 577

WP_035126216_1 IANRRIFPAIDLTSSSTRRDDLLLDENTLKRMWILRKFLADMNPVEAMDTINDKIKKTRNNEEFLIS-MND- 591

WP_073312099_1 IANRRIFPSVDLTSSSTRRDDLLLDEKTIQRMWIMRKYLADMNPVEAMEFINDRFKKTRNNEEFLIS-MND- 572

WP_091464749_1 IANKRIFPAIDLVSSSTRRDDLLLDEKTIQHMWIMRKYLADMNPVEAMEFLESRIKRTRNNEEFLIS-MND- 536

WP_023574714_1 IANRRIFPAIDLTSSSTRRDDLLLDENTIQRMWIMRKYLADMNPVEAMDFINDRFKKTRNNEEFLIS-MND- 577

WP_026712226_1 IANKRIFPAIDLTSSSTRRDDLLLDENTIHRMWIMRKYLADMNPVEAMDFINDRFKKTRNNEEFLIS-MND- 572

WP_091140744_1 IANRRIFPAIDLTSSSTRRDDLLLPKDVIQRMWIMRKYLSDMNPVEAMDFINDRFSKTRNNEEFLIS-MND- 562

WP_091398316_1 IANKRIFPAIDLTSSSTRRDDMLLDEKTLQRMWIMRKYLSDMNPVEAMDFINDRFKKTRNNEEFLIS-MND- 627

WP_066334474_1 IANKRIFPAIDLTSSSTRRDDLLLDENTLQRMWIMRKYLSDMNPVEAMSFINDRFRKTKNNEEFLIS-MND- 603

WP_023571963_1 IANRRIFPAIDLTSSSTRRDDLLLDENTIQRMWIMRKYLADMNPVEAMDFINDRFKKTRNNQEFLIS-MND- 573

WP_026979818_1 IANRRIFPAIDLTSSSTRRDDLLLDENTIQRMWIMRKYLADMNPVEAMEFINDRFKKTRNNEEFLIS-MND- 554

WP_024982133_1 IANKRIFPAIDLTSSSTRRDDLLLDQNTLHRMWIMRKYLSDMNPIEAMDFINDRFKKTRNNEEFLIS-MND- 613

WP_026705806_1 IANKRIFPAIDLTSSSTRRDDMLLDEKTLQRMWIMRKYLSDMNPVEAMDFINDRFKKTRNNEEFLIS-MND- 595

WP_022828308_1 IANKRIFPAIDLTSSSTRRDDLLLDEKTLQRMWIMRKYLSDMNPVEAMDFINDRFKKTRNNEEFLIS-MNQG 574

WP_035657734_1 IANKRIFPAVDLTSSSTRRDDLLLDKDTLQRMWIMRKYLSDMNPVEAMEFINDRFKKTRNNEEFLIS-MND- 599

WP_089059798_1 IANKRIFPAIDLTSSSTRRDDLLLDEKTLQRMWIMRKYLSDMNPVESMDFVNDRFKKTRNNEEFLIS-MND- 612

WP_012026627_1 IANKRIFPAIDLTSSSTRRDDLLLDEKTLQRMWIMRKYLSDMNPVESMDFVNDRFKKTKNNEEFLIS-MND- 604

WP_072972573_1 IANKRIFPAIDLTSSSTRRDDLLLDEKTLQRMWIMRKYLSDMNPVESMDFVNDRFKKTRNNEEFLIS-MND- 611

WP_029273001_1 IANKRIFPAIDLTSSSTRRDDLLLDEKTLQRMWIMRKYLSDMNPVESMDFVNERFKKTRNNEEFLIS-MND- 608

WP_099708795_1 IANKRIFPAIDLTSSSTRRDDLLLDEKTLQRMWIMRKYLSDMNPVESMDFVNDRFKKTRNNEEFLIS-MND- 630

WP_026713087_1 IANKRIFPAIDLTSSSTRRDDLLLDDNTLQRMWIMRKYLSDMNPIEAMEFINDRFRKTRNNEEFLIS-MND- 596

WP_035640151_1 IANKRIFPAIDLTSSSTRRDDLLLDQKTLQRMWIMRKYLSDMNPVEAMDFINDRFKKTKNNDEFLIS-MND- 618

WP_035619884_1 IANKRIFPAIDLTSSSTRRDDMLLDEKTLQRMWIMRKYLSDMNPVEAMDFINDRFKKTKNNEEFLIS-MND- 616

WP_073371274_1 IANKRIFPAIDLTSSSTRRDDLLLDQKTLQRMWIMRKYLSDMNPVEAMDFINDRFKKTKNNEEFLIS-MND- 600

WP_091429370_1 IANKRIFPAIDLTSSSTRRDDLLLDEKTLQRMWIMRKYLADMNPVEAMSFINDRFRQTRNNEEFLIS-MND- 581

WP_074723071_1 IANKRIFPAIDLTSSSTRRDDLLLDQKTLQRMWIMRKYLADMNPVEAMSFINDRFRQTKNNEEFLIS-MND- 579

WP_035671177_1 IANKRIFPAIDLTSSSTRRDDMLLDQKTLQRMWIMRKYLSDMNPVEAMDFINDRFKKTKNNEEFLIS-MND- 595

WP_099714848_1 IANKRIFPAIDLTSSSTRRDDMLLDQKTLQRMWIMRKYLSDMNPVEAMDFINDRFKKTKNNEEFLIS-MND- 596

**flavo9.00582, Protein translocase subunit SecG**

WP_084139998_1 -MNSMG-FSVFLVLITIVCFLLIVVIMVQNPKGGGLSSTFG-NSQQIGGVQKTTDFLDKSTWTLGIILIALIMLSSLAF-N-G---KSEAITIEK-GDAAPAK-TE----VTV--------------P-AM-----E--APATE-KPAE---TAPN-TEEQK----- 121

WP_041253276_1 -MS--T-FTIFLVLITIVCFLLVVVIMVQNPKGGGLSSTFG-SSQMMGGVQKTTDFLDKSTWYLAGTLLVLILLSSLSF-N-GTSNDTGSKLIDE-SA-APAA-------IP---A-----------A-SGT-------NGKTA-TPST--PVKAA-TDEKK----- 120

WP_091309830_1 -MS----FTIFLALIIIVCFLLILTIMVQNPKSGGLSSSFG-GGGQMGGVKNTTDFLEKTTWVLGGSLILLILLSSLSF-N-G--STGGSKLVDE-NATPATE-------KSA--V-----------P-APA-------KTPAA-KPVD--------SAK------- 112

WP_054409500_1 -----MGFTIFLVLITIVSFLLVVVIMVQNPKGGGLSSSIG-GPQIMGGVQKTTDFLDKSTWTLGTILVILILLSSLSF-G-T-FASNDG-IIDE-GAVAP-K-TE----APA--KTG------V----PAQ--P-G--AAQPA-APAQ----PAN----------- 117

WP_041252093_1 MIP----FTIFLALIVIVCFLLILVIMVQNPKSGGLSSSFG-GGGQMGGVQKTTDFLEKSTWFLGGALILLILLSTLSN-A-G--GNGGSKLIDD-AT--TAA-------KPA--P-----------V-PTT-------APAAS-QPAA--------DTTKK----- 113

WP_026990478_1 -----MEFSIFLVLITIVSFLLVVVIMVQNPKGGGLSSSIG-GPQIMGGVQKTTDFLDKSTWTLATLLVVLILLSSLSF-S-TSFSGETP-ITDE-SAV-P-A-AP----APA--VNG------TTAP-AAQ--PQG--TTAPA-PATT----PAQ----------- 121

WP_073363129_1 -MS--T-FTIFLVLITIVCFLLIIVVMVQNPKGGGLSSALG-GTQQLGGVQKTTDFLDKSTWVLGAALIVLVLLSSMSF-P-GS-GGSTDKIIDA-SQSAAPA------------------------P-EDA--KEA--KPGDA-TPKP--AETPA-TPEKK----- 120

WP_023570748_1 -----M-FSIFLVLITIVCFLLVVVVMVQNPKGGGLSSAIG-GSQMMGGVQKTNDFLDKSTWTLAGLLIALILLSSLSF-T-GTLSDTGSKIIDN-STPAPVA-AP----APA--T-A------T--P-EAA--KQE--TPATT-DAAQ----PAA-TEEAK----K 126

WP_072784571_1 -----M-FTGFLIAITIVCFLLILAIMVQNPKGGGLSSTFGAGAGQLGGVQKTTDFLDKSTWTLAGVLLALILLSSLSF-G-S--SSTDSKVLGT-DDVTIPA-VQ----TPA--A-----------P-EAN-------TATGQ-TTDNAATTSEA-TEEAA----K 124

WP_091466306_1 -MS----FTIFLALIIIVCFLLILTIMVQNPKSGGLSSSFG-GGAQMGGVQKTTDFLEKSTWVLAGALIVLILLSALSF-N-G--STGGSKLVDE-NA--PVS-------APA--T-----------P-AAS-------KPAAT-TPAA--------DSTKK----- 112

WP_026718351_1 -MG----FTGFLIAITIVCFLLILAIMVQNPKGGGLSSSFG-GTQQIGGVQKTTDFLDKSTWYLGIALIVLIFLSTLSFEN-G--GVNGSKIVSE-SEITAPV-------VPT--T-----------P-ATN-------AQPAA-ATQA--------NDTTK----- 115

YP_001296849_1 -MS--T-FTIFLVLITIVCFLLIVVIMVQNPKGGGLSSSIS-GSTQMGGVQKTTDFLDKSTWYLGAILIVLILMSSLSF-N-GNNSDTGSKVIDE-SALAAPA-------IPT--A-----------P-QATPAA----TKTTE-TPAT----KPV-TEGTK----K 124

WP_011964085_1 -MS--T-FTIFLVLITIVCFLLIVVIMVQNPKGGGLSSSIS-GSTQMGGVQKTTDFLDKSTWYLGAILIVLILMSSLSF-N-GNNSDTGSKVIDE-SALAAPA-------IPT--A-----------P-QATPAA----TKTTE-TPAT----KPV-TEGTK----K 124

WP_035125549_1 -MS--T-FSIFLVLITIVCFLLIVVIMVQNPKGGGLSSSIG-GSQMLGGVQKTTDFLDKSTWVLATSLIVLVLLSSLSF-T-GTLSDTGSKVIEDAPATSAPAPTTPKA-DAA--T-----------T-TTA-------QPAAT-TTTETAPPPPP-TTEKK----- 131

WP_026979527_1 -----M-FSIFLVLITIVCFLLVVVIMVQNPKGGGLSSAIG-GSQMMGGVQKTNDFLDKSTWTLAGLLIVLILLSSLSF-T-GALSDNGSKLIDG-STPAPAA-TT----APA--A-------------TDA--KQE--TPATTPDAAQ----PAV-TDEAK----K 124

WP_073368546_1 -MS--T-FSIFLVLITIVCFLLVVVIMVQNPKGGGLSSSIG-GSQMLGGVQKTTDFLDKSTWTLATVLIALILLSSLSF-D-GSLSDTDSKIIEK-SETTTAP-----A-QNA--A-----------P-VQS-------TPAAE-PVKK------------------ 113

WP_023573503_1 -----M-FSIFLVLITIVCFLLVVVVMVQNPKGGGLSSSIG-GSQMIGGVQKTNDFLDKSTWTLAGLLIALILLSSLSF-T-GNLGENASKIIDN-STAVPAA-TP----APA--APA------T--P-EAS--KQE--APAAT-DAAQ----PAAATEEAK----K 128

WP_026704078_1 -----M-FSIFLVLITIVCFLLVVVIMVQNPKGGGLSSTLG-GSQQLGGVQKTTDFLDKSTWTLATVLILLILFSSLSF-T----SGSETKIIDD-SEIAAPK-PA----TPA--TPA------T--P-ATE--TAP--ATETT-PATQ----PAE----------- 118

WP_091391390_1 -MT--T-FSIFLVLITIVCFLLIVVIMVQNPKGGGLSSSIG-GSQMLGGVQKTTDFLDKSTWALGTILIVLILLSSLSF-T-GTLSDNDSKIIDNVNKEAAPAPKT--A-TPA--A-----------P-TTE-------KPAAA-TTEPAATPAPQ-PAK------- 127

WP_073310090_1 -MS--T-FSIFLVLITIVCFLLVVVIMVQNPKGGGLSSTFG-SSQMMGGVQKTTDFLDKSTWYLGATLIALILLSSLSF-N-GGNSDNGSKVIDE-SAVTTPA-------IPS-SA-----------P-AAT-------ENKAT-TPAE--TTKPA-TEEAK----K 124

WP_035658485_1 -MS--T-FSIFLVLITIVCFLLIVVIMVQNPKGGGLSSSLG-GSTHLGGVQKTTDFLDKSTWTLAIALIALILLSSLSF-T-GSLSDNDSKIIDN-TEAAAPA-------TPA--A-----------TNQPA-------APATP-APAK------------------ 113

WP_091143007_1 -MS--T-FSIFLVLITIVCFLLIIVIMVQNPKGGGLSSSLG-GSQMLGGVQKTTDFLDKSTWTLGTILIVLIMLSSLSF-S-GSLNDNSSKIIDEIPAAEAPA-----A-ASA--T-----------P-ATE-------TPASD-AAKTEAAPAEA-TPATEAPATK 131

WP_091473472_1 -MS--T-FTIFLVLITIVCFLLIVVIMVQNPKGGGLSSSLG-GSTQMGGVQKTTDFLDKSTWYLAGALIVLILMSSLSF-N-GGSSDTGSKVIDD-SAVTAPA-------IPT--A-----------P-QATPEAAKTETKATE-TPATTEAAKPV-TEEAK----K 132

WP_029273329_1 -MS--T-FSIFLVLITIVCFLLIVVIMVQNPKGGGLSSTIS-GTQMLGGVQKTTDFLDKSTWTLATILIALILLSSLSF-S-GSLSDTDSKIIEK-TE--APA-----NNAPV--A-----------P-VQQ-------TPAPA-TPAAK----------------- 113

WP_089053992_1 -MS--T-FSIFLVLITIVCFLLIVVIMVQNPKGGGLSSTIS-GTQMLGGVQKTTDFLDKSTWTLATILIVLILLSSLSF-T-GSLNDTGSKIIEK-TE--APA-----ATTPA--A-----------P-AQQ-------TPAPA-APATK----------------- 113

WP_012023921_1 -MS--T-FSIFLVLITIVCFLLIVVIMVQNPKGGGLSSTIS-GTQMLGGVQKTTDFLDKSTWTLATILIALILLSSLSF-T-GALGDTGSKIIEK-GE--APA-----ATAPA--A-----------P-AQQ-------TPAPA-APAK------------------ 112

WP_099710971_1 -MS--T-FSIFLVLITIVCFLLIVVIMVQNPKGGGLSSTIS-GTQMLGGVQKTTDFLDKSTWTLATILIALILLSSLSF-S-GSLSDTDSKIIEK-TE--APA-----N-TPA--A-----------P-VQQ-------TPAPA-TPAAK----------------- 112

WP_026985706_1 -MS--T-FSIFLVLITIVCFLLIVVIMVQNPKGGGLSSTIS-GTQMLGGVQKTTDFLDKSTWTLATILIALILLSSLSF-T-GSLSDTDSKLIEK-TE--APA-----T-TPA--A-----------P-VQQ-------TPAPA-TPAAK----------------- 112

WP_035670231_1 -MS--T-FSIFLVLITIVCFLLIVVIMVQNPKGGGLSSTIG-GSQMLGGVQKTTDFLDKSTWTLATILIVLILLSSLSF-T-GALSDNDSKIIEQ-TGTAAPA-----TATPA--S-----------P-VQN-------TPAET-TPAK------------------ 114

WP_026712294_1 -MS--T-FSIFLVLITIVCFLLIVVIMVQNPKGGGLSSSIG-GSQMLGGVQKTTDFLDKSTWTLAIVLIALIILSSLSF-T-GAVSDTNSKIIDN-TEAAVPA-------TPA--A-----------S-QPA-------APATP-APAK------------------ 112

WP_074721500_1 -MS--T-FSIFLVLITIVCFLLIVVIMVQNPKGGGLSSTIG-GSQMLGGVQKTTDFLDKSTWTLATILIALILLSSLSF-T-GTLSDTDSKIIDN-TENAAPV-------VPT--T-----------P-IQD-------VPATK----------------------- 108

WP_024982278_1 -MS--T-FSIFLVLITIVCFLLIVVIMVQNPKGGGLSSAIG-GSQMLGGVQKTTDFLDKSTWTLATILIALILLSSLSF-T-SSMGDSDSKIIQK-TETAAPQ-----T-APV--Q-----------N-APA-------TPAAP-ATK------------------- 112

WP_035623319_1 -MS--T-FSIFLVLITIVCFLLIVVIMVQNPKGGGLSSTIS-GSQMLGGVQKTTDFLDKSTWTLATILIALILLSSLSF-T-GSLSDVESKIIEK-SATTAPV-----T-TPA--A-----------P-TQE-------APAAT-NPAK------------------ 113

WP_066336350_1 -MS--T-FSIFLVLITIVCFLLIVVIMVQNPKGGGLSSTLG-GSTQMGGVQKTTDFLDKSTWTLATILIALILLSSLSF-T-GTLSDTDSKIIDN-TQVATPA-------APVQNT-----------P-APA-------TPAK------------------------ 109

WP_091432647_1 -MS--T-FSIFLVLITIVCFLLIVVIMVQNPKGGGLSSTLG-GSTQMGGVQKTTDFLDKSTWTLATILIALILLSSLSF-T-GTLSDTDSKIIDN-TQTAAPV-------APV--T-----------P-VQD-------APAAE----------------------- 108

WP_072942645_1 -MS--T-FSIFLVLITIVCFLLIVVIMVQNPKGGGLSSTLG-GSTQMGGVQKTTDFLDKSTWTLATILIALVLLSSLSF-T-GTLSDTDSKIIDN-TETAAP--------APV--Q-----------N-VPA-------NPAK------------------------ 106

WP_051877763_1 -----M-FSIFLVLITIVCFLLIVVIMVQNPKGGGLSSTIS-GSQMLGGVQKTTDFLDKSTWTLATILIALILLSGLSF-T-GTLSDSESKIIEK-SETAAPA-----T-APA--Q-----------N-APA-------TPATP-APAK------------------ 111

WP_099714465_1 -MS--T-FSIFLVLITIVCFLLIVVIMVQNPKGGGLSSTIG-GSQMLGGVQKTTDFLDKSTWTLATILIALILLSSLSF-T-GTLSDTDSKIIEN-TGSAAPA-----TNTPA--A-----------P-AQN-------TPAAT-NPAE------------------ 114

WP_014084450_1 -----M-FSIFLILITIVCLLLIVVIMVQNPKGGGLSSSIG-GSQMLGGVQKTTDFLDKSTWTLGAVLLVLILLSSLSF-I-G--NDTASKIIDE-KEIAAPK-PT----TPA--A-----------P-AAP-------APATE-KK-------------------- 108

WP_022826783_1 -----M-FSIFLVLIVIVCFLLIVVIMVQNPKGGGLSSTLG-GSQQMGGVQKTTDFLDKSTWTLGILLIVLIMFSSLSF-NSG---NSEVKILDN-KQVAPAK-TE----VPA--------------P-ATT--NNA--TPAVE-VPAE------------------ 112

WP_091522514_1 -MN--M-FTIFLVLITIVAVLLIIVIMIQNPKGGGLDSSLG-GSTSIGGVQNTNKFLDKSTWTLAAALVILVLVSSLSF-Q-GG-YSTDSKILDP-NAVPAQP-AA----LPT--A-GAAQGQAQ--P-AQN-------TPSEQ-APAN--------TPAQP--A-N 128

WP_091098166_1 -----M-FTLFLVLITIVSLLLIIVIMIQNPKGGGLDSSLG-GSTSVGGVQNTNKFLDKSTWTLAAALVILVLVSSLSF-T-SD-YSADSKILDP-NTVITQP--T----LPN--A-A-----------PQS-------TPSQE-TPVT--------QEQQP--A-N 117

**flavo9.00756, predicted transcriptional regulator, contains HTH domain**

WP_091523840_1 ----MKFSKQLQKIMHYFGLSTTELADRILVPKATISHLISERNKPSLEFIMKLHTTFPTLNLEWLIYEKEPFLI--TE--IH-PKS-IE-KDQIETPVLDE---ILEVETVNE----I-----ENEDQNK-----IET-EEIEPKNISQNITENPKN-----ILSFQSK-----EIDCIVIFYNDGSFKKYNP--------- 155

WP_091097287_1 --------------MHHFSLTTTELADKITVPKATISHLISERNKPSLEFIMKLHTHFPTLNLEWLIYGKEPFLI--TE--NF-VQK-NE-PEEKPVAVLNE---KTDLETTPE----M-----EDE-------------IESHPENIEQNTSKKEEN-----ILSFQSK-----EIDCIVIFYNDGSFKQYKP--------- 138

WP_041252031_1 MMQSEEFIKRLEKVFDYYGMSASSFADKIQVQRSSISHLLSGRNKPSLDFILKIVEHFSEVDIMWLLKGYGDFPK--NN------------NEKDVTTTLSS---PEINNIKTP----------LQQDLFT-T---EH--EFSKNELFKNTIETKPTM-SKI-EKNEKEN-----EIEQIVVFYKDGSFKLYK---------- 153

WP_026981527_1 MVNIDDFVKRLEIIFEYYGLSASGFADRIGVQRSSLSHLLSGRNKPSLDFVMKITEVFPEVDLYWILNGKGNFPK--SE--NN-EPV--IQKPETVSTLFSE---NRNEVSKDQ----NEQ-----PDLFT-----KP--SVEKNRVNENSEEKFSNT-RNF-ETLKNNS-----DIDRIVVFYKNGTFKNYIPE-------- 162

WP_091434673_1 MVNTEEFIKRLEVILDYYSLNASAFADKIGVQRSSLSHLLSGRNKPSLDFILKILDVFPDVDLYWILNGKGSFPK--NN--ET-IQM----QEEKQHKEITP---STSDAISEP----L-----PTQNLFS-K---EN--DTNQQNKVSPKKEEIQNV-----AGRAVSE-----EVEKIVFFYKDGTFKEFKPN-------- 158

WP_035625171_1 MVNIEDFIKRLETILDYYGLNASSFADKIGVQRSSMSHLLSGRNKPSLDFILKIIEIFPDVDLYWVLNGKGSFPK--NE--NI-EVV----TNSVPEEIEKP---ITPIFPNKN----F-----IPEDLFL-E---IE--SPTEKKIIEKNTPEIKNVIPNSSPSYSSSE-----EIEKIVLFYKNGTFKIYTP--------- 162

WP_024981768_1 MVNSDDFNKRLEIILEYYGLTAASFADQIGVQRSSLSHLLSGRNKPSLDFVLKLIAVFPEVDLYWILLGNGNFPK--QE--MV-VDK------------------SN---HTKT----L-----PSPTDTS-T---VL--SNEKTVMTLKQDKKKPAL------QTVSEE-----QVEQLILIYADGSFKTLRPKDGLLGLPL 151

WP_074723368_1 MVNTEDFIKRLEIILDYYSLNASAFADKIGVQRSSLSHLLSGRNKPSLDFILKILDVFPDVDLYWILNGKGSFPK--ND--EP-IQN----QEKKLDEIIKP---NAPAPILES----I-----APQNLFS-N---EK--NDGDKNRAVAKSENIKNA-----VNESISG-----EVEKIVFFYKNGTFKVFKPN-------- 158

WP_035672400_1 MVNIDDFIKRLEIILDYYGLNASSFADKIGVQRSSLSHLLSGRNKPSLDFILKILDVFPDVDLYWILNGKGTFPK--NT--AQ-INT----KENVFEEVVKQ---NIPTPLNNE----I-----IPENLFS-E---IK--NVNPINTIETKKVESQNI-----AKEFFSG-----EIDKIVIFYKNGTFKSYVPD-------- 158

WP_072783983_1 MINTDDFIKRLEAIFEFYGLSASTFADKIGVQRSSLSHLLSGRNKPSLDFILKVDEVFEEVDLYWLLNGTGTFPK--SE--KK-ITE----AIKEVEKNSNDVL-RENLKINDN----F------SQDLFS-E---VS--EASTSTPIEKITNELITE-KKI-SETIIES-----DIDYIVIFFKNGKFKKYTP--------- 161

WP_035657570_1 MVNTDDFIKRLEIILDYYSINASAFADKIGVQRSSLSHLLSGRNKPSLDFILKILEVYPEVDLYWILNGKGNFPK--ND--EN------------LNDLKVA---TNSAPALET----VISSLKPAKDLFS---------NNPTEEKTAISFKKTNEA-----LQNNSEK-----EIEKIVFFYKDGTFKTFKPD-------- 153

WP_072970706_1 MVNIDDFVKRLEIILDYYGLNASSFADKIGVQRSSMSHLLSGRNKPSLDFVMKILDVFPDVDLYWILNGKGSFPK--AE--DQILNT----NNEIATEIVKP---LTPISSSED----F-----IGGDLFS-E---IN--YKEEVKTETRNAVEVKNS-----NFISKEG-----EVEKIVFFYKNGTFKVYIP--------- 158

WP_026710521_1 MQEGNDFVEKLEKILAYFQLSASAFADKIGVQRSSLSHLLSGRNKPSLEFAIKVTETFPEVDLYWFLMNKGSFPK----------------------------S-INSTPVNSS----I---------------------EKNNSKPEEKSNTNEPKS--FF-APSSTSE-----NIEKIIVFYTNGTFTTHHPK------K- 134

WP_014084187_1 MLNIDEFTKRLEKIFDYYSISASGFADKIGVQRSSLSHLLSGRNKPSLEFILKIVDVFPEVDLYWILNGKGTFPK--TI--TP--------PPPPVEKKSIP-Q-TTPKVIQET----L---FDTTTDYPT-I---EP--TPIKNPVFEKKQDSISNT-----ATMEGSL-----EIEKIVFFYKNGTFKTYQPQ-------- 158

WP_066331229_1 MLNTEDFIKRLEIILEYYGLNASTFADKIGVQRSSLSHLLSGRNKPSLDFVLKILDVYPEVDLYWILNGKGSFPE--SA--ST-VSD------------------QPALPQTEN----L-----VGPSLFD-G---IE--PQTETPTDIPEKKPEAML-----PKTYTQA-----GIDKIVVFYQDGTFMSYVPK-------- 147

WP_099715606_1 MVNIEDFIKRLEIILDYYSLNASSFADKIGVQRSSLSHLLSGRNKPSLDFILKILEVFPDVDLYWILNGKGNFPK--NS--EQ-FDK----KENSVEQIVKQ---NIATPPLTE----I-----IPENLFS-E---IK--IPNPIPALETKKIENQNS-----SKESHSD-----EIDKIVIFYKNGTFKSYIPD-------- 158

WP_026713573_1 MVNTDDFIKRLEIILDYYGMNASAFADKIGVQRSSLSHLLSGRNKPSLDFILKILDVYPEIDLYWILNGKGNFPK--TN--SN------------SPEKETG---TTSTPIHTS----IDTVSDANKELFS---------ETTNPNKID---KKESDS-----FP-SFDE-----NIEKIVFFYKDGTFKTYKPNS------- 150

WP_026715563_1 MVNIEDFIKRLETVLEYYSLTASSFADKISVQRSSMSHLLSGRNKPSLDFIMKLAEEFPEVTLEWILNGKGSFPK--DD--FT-PAP--T-FIQPEKKIEQP---TKEDLFSNK----L-----EEKNNLL---------EANKATQISTPENQIRNQ-----NTIQTES-----EIERIVVFFKNGTFKNYIH--------- 155

WP_072943264_1 MVNIEDFIKRLEIILDYYGLNASAFADKIGVQRSSLSHLLSGRNKPSLDFILKILDVFPDVDLYWILNGKGVFPK--SP--GE-TYN----PENKIAEVVNS---TIPSPTQSD----L-----IPEDLFT-A---AN--PATKPEEEITKTKENPNR-----PIYPNSG-----EVEKIVFFYKNGTFKVFNPN-------- 158

WP_029270355_1 MVNIDDFVKRLEIILDYYALNASSFADKIGVQRSSMSHLLSGRNKPSLDFVMKILEVFPDVDLYWILNGKGTFPK--SN--DESLNS----KTDSSSEITKS---TSPISLNEN----F-----GSTDLFS-E---IN--YQEEQKTESKNFSENKNS-----NLDSDER-----EIEKIVFFYKNGTFKVYVP--------- 158

WP_091313841_1 MINTDDFVKRLETIFDYYGLTASSFADKINVQRSSISHLLSGRNKPSLDFILKVIENFPDVELLWLLNGKGNFPK--GENYNP--------EKMEITPIQKN---EKEEEINSS-------------DLFS-NNV-SSI-ENEKNRVFENISKKFSNT-EVF-NNPTNALNNNS-DIEQIVIFYKDGTFKTYSQK--NPNLK- 168

WP_099718286_1 MVNIDDFVKRLESILDYYGLNASSFADKIGVQRSSMSHLLSGRNKPSLDFVMKILDVFPDVDLYWILNGKGNFPK--SD--HESTIS----KADNFVENRNL---NSPSPSNDN----F-----SGEDLFS-Q---IE--YETE-NPQTKKIELKKNS-----NLTVEED-----EIEKIVFFYKNGTFKVYTP--------- 157

WP_099712513_1 MVNIDDFVKRLEIILDYYGINASSFADRIGVQRSSMSHLLSGRNKPSLDFVMKILDVFPDVDLYWLLNGKGNFPK--NE--EENSKV----KNYETLETEKT---NPPISSNDN----F-----VAGDLFS-Q---IN--YKEEEKAPIRYSSEVKNQ-----NLIPEEG-----EIEKIVIFYKNGTFKAYAP--------- 158

WP_073371427_1 MVNTEDFIKRLEIILDYYSLNASSFADKIGVQRSSLSHLLSGRNKPSLDFMLKILDVFPEVDLYWVLNGKGTFPK--SE--SE-------------NNINLS---T-PTPIAET-------KIEKATDLFSVE---LSNKKNPVEEKLATSISNTQNS-----VLSKKNS-----EIDQIVIFYKNGTFKTYSPE-------- 154

WP_091464881_1 MLNIDEFVKRLEIIFDYYGLTASSFADKINVQRSSISHLLSGRNKPSLDFIMKLIETFPEVDLIWILNGTGKFPK--SE--NP--------IQTISSTEIKL---ETEEKIADSNENSF---TNLQADLFS-TPIHEKI-EIEKNRVNENDFKNFSNT-EVL-TTTNFDTNSDQLEIEKIVIFYTNGTFSSYKQK--NPNSK- 178

WP_012024864_1 MVNIDDFVKRLEIILDYYGLNASSFADKIGVQRSSMSHLLSGRNKPSLDFVMKILDVFPEVDLYWMLLGKGNFPK--SE--NETLKF----EPKSDIKSE-----DSPASSNEN----H-----SEIDLFS-Q---LH--YEEE-KTPVKNYAEPKRS-----NISFEED-----EIEKIVFFYKNGTFKAYVP--------- 155

YP_001295757_1 MVNIDDFIKRLEIILDYYGLSASGFADKVGVQRSSLSHLLSGRNKPSLDLILKINENFPEVDLYWILNGKGNFPE--LE--IK-TEP----NIQNTTPILNS---NIEENMPED----F-------PNLFS-D---ED--QNVKNPVFENIKNNFSNT-GNT-SNAKHNS-----EIERIVVFYKNGSFKNYLPE-------- 159

WP_011962997_1 MVNIDDFIKRLEIILDYYGLSASGFADKVGVQRSSLSHLLSGRNKPSLDLILKINENFPEVDLYWILNGKGNFPE--LE--IK-TEP----NIQNTTPILNS---NIEENMPED----F-------PNLFS-D---ED--QNVKNPVFENIKNNFSNT-GNT-SNAKHNS-----EIERIVVFYKNGSFKNYLPE-------- 159

WP_041332228_1 MVNIDDFIKRLEIILDYYGFSASGFADKVGVQRSSLSHLLSGRNKPSLDLILKINENFPEVDLYWILNGKGNFPE--LE--IK-TEP----NIQNTTPILNS---NIEENMPED----F-------PNLFS-D---ED--QNVKNPVFENIKNNFSNT-GNT-SNAKHNS-----EIERIVVFYKNGSFKNYLPE-------- 159

WP_091477437_1 MVNIDDFIKRLEIILDYYALSASGFADKVGVQRSSLSHLLSGRNKPSLDLILKIVENFPEVDLYWILNGKGNFPK--SE--IK-MES----DFQTATPILKN---TIEENVTED----F-------PNLFS-D---EN--ENQKNPVFENIKNNFSNT-GNM-SSAKNES-----EIERIVVFYKNGSFKNYLPE-------- 159

WP_023572553_1 MVNIDDFVKRLETILDYYGLSASGFADKIGVQRSSLSHLLSGRNKPSLDFIMKIIQEFPEVDLYWILNGKGNFPK--SD--FS-EESVLIEKPAAPASLFSDIGTEKKSETKNE----I-Q-----PDLFS-T---EP--SVEKNRIFENQKDFFSNT-RNF-EEVQNNQ-----EIDRIVVFYKNGTFKSYSPE-------- 167

WP_023571388_1 MVNTEDFVKRLEIILEYYGLSASGFADKIGVQRSSLSHLLSGRNKPSLDFILKIIEVFPEVDLYWILNGKGNFPK--SE--PT-EKS--ISVSDPTPTLFSE---NKTDEVKFE----N-Q-----PDLFS-A---EP--TVEKNRVFENHEENFSNT-RNF-RIPKTDS-----DIDQIVVFYKNGTFKNYFPE-------- 162

WP_022828575_1 MLNTEDFIKRLEFIMDHYSLSASSFADKIGVQRSSISHLLSGRNKPSLDFVMKILDLFPELNLYWFLDGKGSYLK--ND--SE-QIS--V-EEAQNTP--TP---IFEEVKKEE------------ESAMK-V-------ENAEELKSDNPPSSYANL---L-NQNSSTK-----QIDTIVFFYNDGTFYDYKPS--NPK--- 156

WP_026705126_1 MVNTDDFIKRLEILMEHFGMNASSFADKIGVQRSSISHLLSGRNKPSLDFVLKIMDIFPEVNLYWILNGKGNLVK--SE--TD-FSE--K-NKAKNENLHTP---ILSQDLSSE------------KN--N-R-------SESEAIISKNKPDHF--------QISKTDS-----EIFKIVVFYKDGTFKDFDPS-------- 149

WP_054406311_1 MLNIEDFIRRIQILMDYYNISASVFADKLTVQRSSLSHLMSGRNKPSLDFIMRITEAYPEADLYWLLYGQGEFPKNSSQ--SQ-SQF----TGNPQESFKSQ---SQFTAATAN--T-V---AENELDLFS-R-------EESVQPEFSEA--QQLQT-----ETTTENL-----EIERIVIFYTNGTFKNYNPG------Q- 160

WP_026992135_1 MVNAEDFIKRLEILLDYYSLSASLFADKLGVQRSGLSHLMSGRNKPSLDFVMKIVENFPEVDLYWLLAGVGEFPK--TE--NQ-PHF----FTRPTPAVNTN---DVIKGTDAP-------------DLFS-V-------GKEVTESKSNAVDANLNS-----VLAADNK-----DIERIVIFYSNGSFKSYTSG------H- 153

**flavo9.00944, Uncharacterized conserved protein YehS, DUF1456 family**

WP_091519626_1 MTNNDILKKLRVALQLRDDQILDILELVDFRISKGEIGNFFRTEDHPKYVECQDQILRNFLNGLVIYLRGTKENPKNPKDVLAEISA-------SKV----------------A----LTD---K-PKVE-NKPKAI--------------S-ATK---------KP------TTAKPAFEGKNNATIKSNTISPTK-------KD----ILGNVKFNNGKPKKK-D 153

WP_026705667_1 MNNNDVFKKLRVALQLRDEQIIEILALVDFRMEKPELNALFRAQDHPNFMECGDQVLRNFLNGLIIHLRGTKEEPKNAMDVINKNRD-EVKAR----------------------------------LSS-DKPKST--------------G--AP---------KT--F---TKKKPASNPKSREKK------PAP---K---VQ----VVEKVQYKNGKK----- 140

WP_035673822_1 MTNNDIFKKLRVALMLRDDQIVEILELVDFRITKSELGAFFRDEKHENYMECGDQVLRNFLNGLVIHLRGTKENPKNPNDVLAKHKA-LIPVK-EGA----------------S--E-------R-AEFK-AKPRDE--------------E-RSR---GDE---SP------SKSKPAAKKPFKKQF------SKG---TPK-VQ----VVEKVKYNFGKNKKS-- 155

WP_099715997_1 MTNNDIFKKLRVALMLRDDQIVEILELVDFRITKSELGAFFRDEKHENYMECGDQVLRNFLNGLVIHLRGTKENPKNPNDVLAKHKA-QIPVK-EGA----------------S--E-------R-TEFK-AKPRDE--------------E-KSR---GDE---SP------SKSKPAAKKPFKKQF------TKG---TPK-VQ----VVEKVKFNFGKNKKS-- 155

WP_072941114_1 MTNNDIFKKLRVALMLRDDQIVEILELVDFRITKSELGAFFRDEKHENYMECGDQVLRNFLNGLVIHLRGTKENPKNPNDVLAKHKA-QIPAK-QSA----------------K--E-------R-PEFK-AKPRDE--------------E-KSR---GDE---AP------SKSKPAAKKPSKKQY------PKG---NSK-VQ----VVEKVKFNFGKNKKS-- 155

WP_070786681_1 MTNNDIFKKLRVALMLRDDQIVEILELVDFRITKSELGAFFRDEKHENYMECGDQVLRNFLNGLVIHLRGTKENPKNPTAVLAKHKA-QIPAK-TTT----------------K--E-------R-AEFK-ATPRDE--------------E-KSR---ADQ---SP------SES-GAAKKTSKKQY------PKG---NKK-VQ----VVEKVKFNFGKNKKP-- 154

WP_040473818_1 MTNNDIFKKLRVALMLRDDQIVEILELVDFRITKSELGAFFRDEKHENYMECGDQVLRNFLNALVIHLRGTKENPKNPNDVLAKHKA-QIPAK-EGA----------------K--E-------R-PEFK-AKPRDE--------------E-KSR---GDQ---SP------SKSKSATKKPSKKQY------PKG---NAK-VQ----VVEKVKYNFGKDKK--- 154

WP_074723960_1 MTNNDIFKKLRVALMLRDDQIVEILELVDFRITKSELGAFFRDEKHENYMECGDQVLRNFLNALVIHLRGTKENPKNPNDVLAKHKA-QIPAK-EGS----------------K--D-------R-PEFK-AKPKDQ--------------E-KSR---GDE---AP------SKTKSATKKPSKKDY------PKG---NSK-TQ----VVEKVKYNFGKNKK--- 154

WP_022827878_1 MNNNDIFKKLRVALQLRDEQILEILALVDFRMDKPELNALFRAEDHPNFMECGDQVLRNFLNGLIIHLRGTKEEPKNAMDVISKNRD-EVNNRDQSAKPKATSTAPKGKPSSSG----------K-PYSA-NKPYST--------------G--KP---------SS--S---TGKPDAKSSSARPKK------KEP---K---ID----VVEKVRYKNGKK----- 163

WP_029268637_1 MTNNDILKKLRVALMLRDDQIVEILELVDFRITKSELGAFFRAEDHENYMECGDQVLRNFLNGLVIHLRGTKENPKNPNDVLAKHKA-EIPKK-EST----------------K--E-------R-PEFK-ASPKDA--------------E-KSR---GDQ---KP------SKSGTAAKKPFKKNN------SKA---TPK-IQ----VVEKVIYKNGKNKK--- 154

WP_099711269_1 MTNNDILKKLRVALMLRDDQIVEILELVDFRITKSELGAFFRAEDHENYMECGDQVLRNFLNGLVIHLRGTKENPKNPNDVLAKHKA-EIPKK-DST----------------K--E-------R-PEFK-ASAKDS--------------E-RGR---GDK---AP------SKSGPAAKKPKKKEF------PKG---NGK-TS----VVEKVVYKNGKNKK--- 154

WP_072975878_1 MTNNDILKKLRVALMLRDDQIVEILELVDFRISKSELGAFFRAEDHENYMECGDQVLRNFLNGLVIHLRGTKENPKNPNDVLAKHKA-EIPKK-DST----------------K--E-------R-PEFK-ASPKDA--------------E-KGR---GDK---AP------SKTGPAAKKPFKKK--------PA---TPK-IQ----VVEKVVYKNGKNKK--- 152

WP_091308913_1 MNNNDIFKKLRVALQLRDDQIVEILNLVDFKMSKGELGDIFRAEDHANFMECGDQVLRNFLNGLIIHLRGTKENPKNPRDVIKSHKD-YETQT-ERT----------------D----------K-TIYE-PKKPYNKTDSKTQFKPRTGDK-KPFAKDGDKKEFKP--K---TGESSDKKPAFGKNKFGNDKKPAK-------QN----PVEKFKFKDGKK----- 175

WP_012024233_1 MTNNDILKKLRVALMLRDDQIVEILELVDFRISKSELGAFFRAEDHPNYMECGDQVLRNFLNGLVIHLRGTKENPKNPNDVLAKHKS-EIPKK-ETS----------------K--E-------R-PEFK-AAPKDS--------------E-KYR---GDQ---SS------SKSGSSAGKPKKKAF------PKG---NGK-PA----VVEKVVFKNGKNKK--- 154

WP_099719966_1 MTNNDILKKLRVALMLRDDQIVEILELVDFRISKSELGAFFRAEDHPNYMECGDQVLRNFLNGLVIHLRGTKENPKNPTDVLAKHKS-EIPKK-ETS----------------K--E-------R-PEFK-ASQKDS--------------E-KYR---GDQ---SS------SKSGSSAGKPKKKAF------PKG---NGK-PA----VVEKVVFKNGKNKK--- 154

WP_023573934_1 MTNNDIFKKLRVALQLRDDQIVSILELVDFRISKGEIGNFFRNEDHPKYMECGDQVLRNFLNALIIHLRGTKDSPKNPLDVIKSHKN-DAPKT-EEG----------------R----------R-TDDK-PKFSSEKPKNTGS---------------------KP--K---SKTDFKKPASKGKPA------PKK-------EP----IVERVKFTLGKKKDN-K 153

WP_091395781_1 MDNNDIFKKLRVALQLRDEQILEILQLVDFRISPAELGAFFRKQDHPNYMECGDQVLRNFLNGLVIHLRGTKENPKNAMDVISENNK-NVQAS--------------------A----------P-RFPK-DQPIKN--------------TFPKP---------KP------AGTKPFKKQAPKKPA------PKI---E---------VVERVKFNNGKNKK--- 144

WP_054405970_1 MNNNDIFKKLRVALQLRDDQIVDILQLVDFRITKAELGAFFRNADHPNYMECGDQVLRNFLNGLVIHLRGTKENPKNPQEVLAQNKA-AAATA--------------------P----------KLKTDA-KQPSKG--------------A-PAP---------RP--K---AKTQATKTTSKGKKF------TKP---AKP-AP----VVEQVKFNNGKGKTK-- 150

WP_026993161_1 MNNNDVFKKLRVALQLRDDQIVAILELVDFRISKAELGAFFRAEDHPNFMECGDQVLRNFLNGLVIHLRGTKENPTNAMDVIEKNRA-AIKSQ--------------------S----------A-PTDR-PAPSKG--------------A-PAP---------RP--K---AKKPAAKGPSQNKKF------DKA---KKP-AP----IVEQVKYNNGKNKKG-- 149

WP_035622315_1 MTNNDILKKLRVALMLRDDQIVEILELVDFRISKSELGAFFRAEDHENYMECGDQVLRNFLNGLVIHLRGTKENPKNPNEVLAKHKA-EIPAK-GST----------------K--E-------R-PEFK-AKPKDE--------------E-GAR---GDQ---RP------SKSGTGAKKPFKKSN------SKS---TPK-IQ----VVEKVKFNFGKNKKS-- 155

WP_035633770_1 MTNNDIFKKLRVALMLRDDQIVEILELVDFRISKSELGAFFRDEKHPNYMECGDQVLRNFLNGLVIHLRGTKENPKNPNEVIAKHKA-QIPAK-EGD----------------S--K-------R-PEFK-PKAKDP--------------E-KSR---GDQ---AP------GKSDGAAKKPAKKKF------PKG---NGK-QS----VVEKVKFNFGKNKKS-- 155

WP_073371643_1 MTNNDIFKKLRVALMLRDDQIVEILELVDFRISKTELSAFFRAEDHENYVECGDQVLRNFLNGLVIHLRGTKENPKNPTAVLAKHKA-EIPVK-EGQ----------------K--E-------R-TEFK-AKPKDE--------------E-RAR---GDQ---KP------GQKSTAAK---KKPF------KKP---APK-VQ----VVEKVKFNFGKNKKS-- 152

WP_073311955_1 MNNNEIFKKLRVALQLRDDQIVEIMQLVDFRMSKGEVGNFFRNDDHPKYMECGDQVLRNFLNGLVIHLRGTKDNPKNPTEVL--KKN-------QAE----------------A----------K-SFSK-SNPKTD----------------------------KP--KSDKPKTGSFKKNFNSKSS--ST--QKK-------EP----VVQNVKFKNGKNKTNKN 145

WP_091466793_1 MNNNDIFKKLRVALQLRDDQIVEIFELVDFRISKGEIGNFFRNEDHPKYVDCGDQVLRNFLNALVIHLRGTKENPKNPMDVIKSHKD-NQPQT-ERS----------------E----------K-NIYE-PKKPYTKTGNKSAFKPKTGDK-KTF---------KP--K---TESSSTDKKNTGKPKF-SEKKPAK-------QN----PVEKFKFSQGKPKK--- 167

WP_024982634_1 MTNNDIFKKLRVALMLRDDQIVEILELVDFRISKSELGAFFRDEKHPNYVECGDQVLRNFLNGLVIHLRGTKENPKVPTEVLAQHKA-QIPVK-EGD----------------S--K-------R-AEFK-PKAKDQ--------------E-RGR---GDE---SP------SKKKPAAKKPFKKQF------PKG---NDK-IQ----VVEKVKFNYGKNKKS-- 155

WP_073364872_1 MNNNDIFKKLRVALQLRDDQIVAILDLVDFRISKGELGNFFRDAEHPKYMECGDQILRNFLNGLIIHLRGTKDAPKNPLEVIATHKK-TPPKQ-EVS----------------D----------K------PKGP------------------------------TP--K---SYGKKPVKKFNAKPVI----------------------VEKVNYKNGKKKE--K 134

WP_014165313_1 MTNNDIFKKLRVALQLRDDQIVEILELVDFRISKGELGNIFRNEDHPKYMECGDQILRNFLNALIIYLRGNKEISKNPLEILNANKQ-------NIK----------------K----LPS---D-KSIE-KKPA----------------D-KIF---------KP------INKQASFKKNTK----------TS-------NP----VFEPVRFKNGKKKG--- 139

WP_091146741_1 MNNNDIFKKLRVALQLRDDQIVDILELVDFRISKGEIGNFFRNPDHPKFVECGDQVLRNFLNGLVIHLRGTKESPKNAMDSIAQNKA-QVKSA-EKQ----------------RFPKSIGTKTGA-SGTK-PKPAAH----------------------------KP---------------TAKKPT------TKK-------PE----IVENVKFKNGKNKKS-- 145

WP_026715876_1 MTNNDIFKKLRVALQLRDEQIVEILELVDFRISKGELGNVFRKEDHPSYMECGDQLLRNFLNGLVIKLRGTKEDPKEASEELSKIAN-SKIDL-ETG----------------E--K-LGT-T-K-KPVA-KKPYTK------DFP-----K-KKY----DD---KP------KYDDKPKYDSKKKPS--YKDKPKK-------VE----IVEKVKFNNGKPKK--- 161

WP_035125921_1 MTNNDIFKKLRVALMLRDDQIEQILMLVDFRMSKGEIGNFFRNADHPKYVECGDQVLRNFLNGLVIHLRGTKENPKIPMDVIAVHKQ-------EAR----------------P----------K-EFTK-SEKATA----------------------------PP--K--EFKAGDFKKSTAAKPK--FK--DKK-------PTKKIEIVEKVNYKNGKNPKG-- 147

WP_026711802_1 MNNNDIIKKLRVALQLRDDQIVEILQLVDFRISKAELGAFFRNADHPNYMECGDQVLRNFLNGLVIHLRGTKESPKNPMDVIKSHKA-NPSQP--------------------T----------E------QKPKQN--------------T-PPK---------KG--N---APKKPFQASTSKPKI------KKS---D---------VVEKVKYKNGKLKK--- 140

WP_026979775_1 MNNNDIFKKLRVALQLRDDQIVEILELVDFRISKGEIGNFFRNEDHPKYVDCGDQVLRNFLNGLVIHLRGTKENPKNPMEVLKGHKA-------EAQ----------------K----------N-DAAK-PKTAIQ----------------------------KP--K---ADFKKIAPKGKAKPA------PKK-------EP----VVQNVKFKNGKKKDS-K 141

WP_035657761_1 MTNNDIFKKLRVALMLRDDQIVEILELVDFRISKSELGAFFRDEKHPNYVECGDQVLRNFLNGLVIHLRGTKENPKNPNEVLAKHKA-EIPAK-AKE----------------I--P-------K-TQAITSKPDFK--------------K-KP-----SS---K-----------------------------KA---APK-IQ----VVEKVKYNNGKNKKS-- 137

WP_014389056_1 MTNNDIFKKLRVALQLRDDQIVEILELVDFRISKGEIGNFFRNEDHPKYVDCGDQVLRNFLNGLVIYLRGTKENPKNPMEVLKTNAANKIPLQKEEQ----------------K----------N-NPTS-KKPAAK----------------------------KPFGK---SKF--------ADKK------PVK-------SN----PVERFKFKPGK--DS-K 140

WP_014083905_1 MTNNDIFKKLRVALMLRDDQIVSILELVDFRITKSELGAFFRDEKHENYMECGDQVLRNFLNGLIIHLRGTKDNPKNPNEVLQNIKK-EATFK-PT----------------------------K-PIIK-NTPKSN--------------F-KKK---NTS---K-----------------------------AA---QPK-VQ----VVENVKFNNGKNKKK-- 135

WP_026713099_1 MTNNDIFKKLRVALMLRDDQIVEILELVDFRISKSELGAFFRDSKHPNYVECGDQVLRNFLNGLVIHLRGTKENPKNPKDVLAKHKT-EIPAK-KTT----------------V--S-------N-EPKV-AKTTFK--------------K-KP-----NF---K-----------------------------KA---APK-TP----VVEKVKFNNGKNKKS-- 136

YP_001295817_1 MTNNDIFKKLRVALMLRDDQIVEILELVDFRITKSEIGAFFRDEKHENYVECGDQILRNFLNGLVIHLRGTKENPKNPNDVLAKHRS-EIPKK-TSD----------------K--P-------K-TEFK-PKTDFK--------------K-KPA---SNN---K-----------------------------KV---EPK-VQ----VVEKVRFNNGKAKK--- 137

WP_011963057_1 MTNNDIFKKLRVALMLRDDQIVEILELVDFRITKSEIGAFFRDEKHENYVECGDQILRNFLNGLVIHLRGTKENPKNPNDVLAKHRS-EIPKK-TSD----------------K--P-------K-TEFK-PKTDFK--------------K-KPA---SNN---K-----------------------------KV---EPK-VQ----VVEKVRFNNGKAKK--- 137

WP_091472720_1 MTNNDIFKKLRVALMLRDDQIVEILELVDFRISKSEIGAFFRDEKHENYVECGDQILRNFLNGLVIHLRGTKENPKNPNDVLAKHRA-EIPKK-TSE----------------K--P-------K-TEFK-PKTDFK--------------K-KPA---SNN---K-----------------------------KA---EPK-VQ----VVEKVRFNNGKAKK--- 137

WP_034098272_1 MTNNDIFKKLRVALMLRDDQIVEILELVDFRITKSEIGAFFRDEKHENYVECGDQILRNFLNGLVIHLRGTKENPKNPNDVLAKHRS-EIPKK-TSD----------------K--P-------K-TEFK-TKTDFK--------------K-KPA---SNN---K-----------------------------KV---EPK-VQ----VVEKVRFNNGKAKK--- 137

WP_023569818_1 MTNNDIFKKLRVALQLRDDQIVEILELVDFRISKGEIGNFFRNEEHEKYMECGDQVLRNFLNALVIYLRGTKENPKNAMDVIKQNQA----------------------------------------AIQ-ARPASD----------------------------KP--K---TDF-KPKTDAKKKPA------PKKTFKSQKPEP----IVEKVRYNNGKKKDN-K 141

WP_072780979_1 MTNNDIFKKLRVALQLRDDQIVEILQLVDFRMSKGELGNFFRDEKHPKYMECGDQVLRNFLNGLVVYLRGTKESPKVPMEVL------------------------------------------A-THKK-QEIKTD----------------------------KP--K-------VTEKPKPKKKV--FV--EKK-------KP----VVQAVKFKNGKKKND-- 129

WP_091097160_1 MTNNDILKKLRVALQLRDDQIVEILNLVDFRISKGEIGNFFRNEDHPKYVECQNQILRNFLNGLVIYLRGTKENPKNPKEVLAAVAA-------SKK----------------P----LTD---K-PLVN-NKIKTD--------------K-KYS---------KP------T-VKPVPKTKTTTPL-------TQ-------KD----IMGNVKFNNGKTKEN-N 145

**deino9.00350, Uncharacterized protein**

WP_051964284_1 MNGMYQAFCEACGEELLFTDQDLNDFQDGDIITCDCCHTDLEIVKTEEGLELHRHVW--MTLCPKCGEEFELTDEMLTK-------------SLAQCPHCKYKFALEWE-E----------------------- 95

WP_017870442_1 MATL-EIDCPVCGEVLELTDSDRAELQVGDVLVCDSCNAEMEVTHNSG-EDFDLELLGILTVCPGCSEEFEVTEDMLAT-APVIESVDGVSVSLVDCPHCRARIELELT-EDV-----ATG-------L----- 113

WP_104991956_1 ------------------------------MIVCDSCHAEMEVTRNGGGEDFELELLGILTVCPACGEEFDVTEELLAA-SPTLEAGDGSAVSVVTCPHCRARIELEFE-DEG-----GGE-GLSDLRS----- 91

WP_062158117_1 MATL-MIECPVCAEVLELTEEDRADLPVGEVIVCDSCHSEMEVTRNDGGEDFELELLGAMTTCPNCDEEFEVTPELLQA-APMTRAQDGVEVALMTCPHCRAKFELELA-DEP-V------------------- 111

WP_107136537_1 MAIL-EVECPICEEVLELTDEDRAELAVGDVIVCASCHSEMEVTRNGGGEDFELDLLSAMTTCPHCDEEFEVTPDMLAA-APATRSQDGAEVSLMTCPHCKVKFELELT-EEQ-A------------------- 111

WP_103313545_1 ------------------------------MIVCDSCNAEMEVTRNGGGEDFELELLGILTTCPNCGEEFEVTEELVAT-APIIRGSDGVEVSVVPCPHCKARIELEFA-AED-----SAAV------------ 85

WP_013556507_1 MAIL-EVECPVCGDVLELTDEDRAELTVGDAIVCDNCHAEMEVTVN-DAQDFELELLGILTTCPNCGEEFDITDDMLDETG-----------THVECPHCHAEITLEYD-----------------------E- 97

WP_102127459_1 ------------------------------MIVCDSCNAEMEVTRNEGGEDFELELLGILTVCPNCGEEFDVTDDMLAA-APTVQGADGVEVSVVTCPHCRARIELEFE-DED-----GSRAG----------- 86

WP_085961130_1 ------------------------------MIVCDSCSAEMEVTRNGEGEDFELELLGILTECPNCGEEFEVTDDLLSA-APVIESADGAAVSLVDCPHCHARIELEFE-PDP-----AEP---SVSGR----- 89

WP_046842604_1 MATL-EIDCPICAEVLELTDQDRAELQVGDVIVCSSCHSEMEVTRNDGGEDFELELLGAMTTCPNCDEEFEVTAEMLQA-APMTRAQDGVEVALMTCPHCRAKFELELA-DEE-S------------------- 111

WP_058976664_1 MATL-EIDCPICAEVLELTDQDRAELQVGDVIVCSSCHSEMEVTRNDGGEDFELELLGAMTTCLNCDEEFEVTAEMLQA-APMTRAQDGVEVALMTCPHCRAKFELELA-DEE-G------------------- 111

WP_027480896_1 MAVL-EVECPICGEFLELTDEDRAQLELGDVIVCDSCNAEMEVTKN-EGDDFDLELLGILTTCPNCGEEFDVTEETLGE-G-----------DIVQCPHCKAYIQLEFE-----------------------E- 96

WP_103128665_1 ------------------------------MIVCDSCNAEMEVTRNGPGEDFELELLGVLTTCPSCGEEFDVTEDMLAE-APIIEAEAGHVASIVSCPHCRARIELEFE-EAE--------------------- 81

WP_055363032_1 MATL-EIECPVCAEVLELTDEDRAELMVGDVIVCDSCHSEMEVTRNGEGEDFDLELLGEMTTCPNCGEEFEVTEDMLAA-APV-QVLDGVEVSVVSCPHCKGLVALELM-DNP-DVI----------------- 112

WP_078299303_1 MATL-EIECPVCAEVLELTDEDRAELMVGDVIVCDSCHSEMEVTRNGEGEDFDLELLGEMTTCPNCGEEFEVTEDMLGA-APV-QVLDGVEVSVVSCPHCKGLVALELM-DDP-DVI----------------- 112

WP_039683633_1 MATL-EIECPVCAEVLELSDADRAELKVGDVIVCDSCNAEMEITRNGGGEDFELELLGILTLCPNCNEEFEVTEDVLAA-APAVQAADGSEVSLVNCPHCQASIELEFE-DEE-DRP-ALSRQ----------- 118

WP_011530287_1 MATL-EIVCPVCAEVLELTDADRSELQVGDVIVCDSCNAEMEVTRNGPGQEFDLVLLGVLTTCPSCGEEFDVTDEMLEA-APTIQNVDGTVASVVTCPHCRAQIELEFE-E--------VD------------- 110

WP_085931733_1 ------------------------------MIICDSCNAEMEVTRN-EGEDFDLELLGILTVCPNCSEEFDVTEEMISE-S-----------SIVQCPHCTAHIELEFE-----------------------DA 68

WP_022802021_1 MPIL-EIECPICAEVLELTDEDRAELSVGDVIVCDSCNAEMEVTRNGEGEDFELDLLGVLTVCPGCGEEFDVTDEMLAA-APLLESPDGSQVSVVECPHCRAKIELEFT-EGS-DAE----------------- 113

WP_056293781_1 MATL-EIDCPVCGEVLELSAEDRAELHPGDVIVCDSCSAEMEVTRNGGAEDFELELLGILTECPNCGEEFEVTDELLSA-APMIESADGAAVSLVACPHCQARIELEFE-AEG-----GEQ---NMSTL----- 118

WP_034384219_1 MATL-EIVCPVCAEVLELTDADRAELEVGDVIVCDSCNAEMEVTRNGPGEDFELELLGILTTCPRCGEEFDVTDELLRD-APTVESADGTVTSLVSCPHCRAQIELEFE-EDGLSRP----------------- 114

WP_019007909_1 MATL-EVECPVCEEILELDDQDRAQLEVGDVIVCDSCNAEMEVTRNGEGEDFELELLGVLTTCPNCGEEFDVTEDMLAA-APVVEGVDGEVVSIVDCPHCRARIELAFE-SDE---PEDTSAV----------- 117

WP_064014910_1 MATL-EVECPVCEEILELTDQDRAELEVGDVIVCDSCNAEMEVTRNGEGEDFELELLGVLTTCPNCGEEFDVTEDMLAA-APVVEGMDGEVVSIVDCPHCRARIELAFE-DEE---PEDVSLR----------- 117

WP_029480721_1 MATL-EIECPVCGEVLELSDQDRAELHPGDVIVCDSCSAEMEVTRNGGGEDFELELLGILTTCPNCGEEFEVTEDMLAT-APVVQASDGSEVSIVGCPHCQAAIELEFE-DEP-DSD-PLSSQ----------- 118

WP_012693407_1 MPTL-EIDCPVCAEVLELTDADRADLQVGDVIVCDSCNAEMEVTRNAPGDDFELELLGVLTLCPSCGEEFDVTDEMIDA-APVIESSDGATVSVVECPHCRARIELEFE-E-------PVEPV----------- 113

WP_029476823_1 MATL-EIECPVCAEVLELTDQDRAELQPGDVIVCDSCNAEMEITRNGGGEDFELELLGILTTCPNCAEEFEVTEELLAT-APTLHASDGSEVSVVNCPHCRASIELEFE-DGP-EGR-SLSSQ----------- 118

WP_027460770_1 MATL-EIDCPVCAEVLELTEEDRAELRPGDVIVCDSCHAEMEVTRNGGGEDFELELLGILTVCPACGEEFDVTEDLLAA-APTLEGPGDVSVSVVTCPHCRARIELEFEDDDP-GGA-PASPS----------- 119

WP_019585715_1 MATL-EIDCPVCAEVLELSDADRADLSVGDVIVCDSCNAEMEVTRNGPGEDFELELLGVLTTCPNCGEEFDVTEDMLAE-APIIEAQAGHVASIVSCPHCRARIELEFE-EAE--------------------- 110

WP_084048991_1 MTTL-EIECPVCAEVLELTDADRAELHVGDVIVCDSCNAEMEVTRSGEGEDFELELLGVLTTCPNCGEEFDVTEDMLAA-AGTVQSHDGVEVSVVTCPHCRARIELEFE-EAE--------------------- 110

WP_034357914_1 MATL-EIDCPVCAEVLELTDADRADLVVGDVIVCDSCNAEMEVTRNGAGEDFELELLGILTVCPNCGEEFDVTDDMLAA-APTVEGADGTVVSVVACPHCRAQIELEFE-ETD--------------------- 110

NP_295915_1 ------------------------------MLVCDSCNAEMEVTRNGGGEDFELELLGILTVCPACSEEFEVTEDMLLT-APVIESVDGVSVSLVDCPHCHARIELELT-DET-----PPD-------EEQPRR 90

**deino9.00475, Exonuclease VII small subunit XseB**

WP_013615611_1 -----------------M----PRAKAV--QEP-TYREAYATLSRIVTELENGETDLDRVLPLLEEARAAYEVCQGRIAAVAQAVGSAGWLED------GAEGT----EG-ME-EDGADGEEE--------- 87

WP_027483774_1 -----------------M------------S---EYREAYATLARIATRLESGDADIDEVLPLLEEARAAYEACRGRLEAV-RRALGDDLADT------DGDDE-DAADE-DE-DEDLDDDED------DPF 84

WP_081790739_1 MGRPEGTCLRVRRYPRGV----PDAPPA--L---TYREAYARLSRIAAELETGEADLDRVLPLLEEARAAYAACRERIEAV-RAVLAGDWMEA-EDG-EEDGGP----GA-GS-PELDADQEA--------- 105

WP_102127238_1 MAQGSGSVAERYARG--V----PD-AQP--L---TYREAYARLSRIAAELESGDADLDRVLPLLEEAKTAYAACRERIEAV-RAVLAGEWATT------EPDVD----ED-DE-ADDPPGDDP--DA--DPY 103

WP_013558249_1 MGGGSVRESLLYTAG--M--S-------------EYREAYATLARVARALEDGEADLDRVLPLVEEARAAYGVVRARIEAV-RAALGDDWEDE-------EGSA----AE----ADDLDDEDD--------- 90

WP_052326651_1 --------MERWTFRV-T------------I---KFKTHYETLARIAARLEEGDADIDEVLPLLEEAKAAYAACQERLEAV-RRALGDAPAEV------PDSDELEV------------------------- 76

NP_296305_1 -----------------MLYAGAMSAPT------SYRDAYARLSRIAAELETGEADLDRVLPLLEEARAAYAECKGRIEAV-RGVLSGDWGQD------AAQDP----EEGEE-------DED--D----AF 85

WP_056301710_1 -----------------M----SPNDPS--L---SYREAYARLSRIAAELEGGEADLDRVLPLLEEAREAYAACQSRIGAV-RAALSGGWDPA------RAADD----PS-AA-GDSSDPDEDSGED----- 88

WP_107138799_1 -----------------M----ARVSPPDAL---SYREAYARLSRIAAELEGGEADLDRVLPLLEEARAAYAGCRERIEAV-RAVLAGHWGDADEDG-PNTDGP----DD-ED-PDETADDED--DA----- 93

WP_019010562_1 -----------------M----PDVSAA--L---SYRDAYAKLSRIAAELESGEADLDRVLPLLDEAKAAYAACVGRIEAV-RAVLAGSWGTE-LVGIPETQSG----ED-PD-ADDEERDEE--DE---TA 93

WP_014686314_1 -----------------M----SPNEPS--L---SYREAYARLSRIAAELEGGEADLDRVLPLLEEAREAYAACQSRIGAV-RAALSGGWDPA------HPAGD----AE-DG-GGADEDSLG--------- 84

WP_034404111_1 -----------------M----PG--DP--L---TYREAYARLSRIAAELESGEADLDRVLPLLEEARAAYAACRERLAAV-QAALAEDWDGE------DGEGE----N-------TAETDEV-------DF 79

WP_034409634_1 -----------------M----SG--DP--L---TYREAYARLSRIAAELESGEADLDRVLPLLEEARAAYAACRERLAAV-QAALAEDWDGE------EGEGE----D-------TAETDEV-------DF 79

WP_017871512_1 -----------------M----GVSASA------SYRDAYARLSRIAAELESGEADLDRVLPLLEEARAAYAECRSRIEAV-RAVLSGEWGQG------EEEEV----ED-TE-------DEG--T----AF 80

WP_019588064_1 -----------------M----PEKSTP--L---SYREAYTRLSRIAAELETGEADLDRVLPLLEEARAAYAACRERIEAV-RAVLAGDWADA-ETG-PEDEDD----ED-EV-MGNGARDASGDDPYADPF 97

WP_012692143_1 -----------------M----PAMKG-------SYREAYTILSRIAAELEAGEADLDRVLPLLEEARQAYAVCRDRIEAV-RAVLAGEWAEV-RD---TAEAP----ED-PE-ADEAADDED-DLD----- 87

WP_103129224_1 -----------------M----PEKTPS--L---SYRAAYARLSRIAAELETGEADLDRVLPLLEEARAAYAACRGRIEAV-RAVLAGEWGGA-ETD-PEDEDD----AD-EA-AEDGAED----DPYADPF 93

WP_**078304764**_1 -----------------M----TEPQPTP-PGPVPYREAYARLSRIAAELESGEADLDRVLPLLEEAREAYAQCRERIEAV-RGVLAGDWADG------DPEDE----SD-AE------------------- 79

WP_099749842_1 -----------------M----TEPQPTP-PGPVPYREAYARLSRIAAELESGEADLDRVLPLLEEAREAYAQCRERIEAV-RGVLAGDWADG------DPEDE----SD-AD------------------- 79

WP_064013706_1 -----------------M----PDVSSP--L---AYRDAYAKLSRIAAELESGEADLDRVLPLLEEAKAAYAACHERIEAV-RAVLAGSWGAE-V-------AG----AD-AE-QDDAD-DAT--DD---PY 85

WP_011529178_1 -----------------M----SDTPTS--L---TYREAYARLSRIATELENGEADLDRVLPLLEEARAAYAACRERIEAV-RAVLAGDWTEG-------DWLG----AE-DA-VDDAENPEG--------- 83

WP_103310712_1 -----------------M----SSDAAL------SYREAYATLSRIAAELESGEADLDRVLPLLEEARVAYAGCRDRLEAV-RAVLAGTWAD--------EGSD----DE-AD-ADEYDEDES--------- 81

WP_104989507_1 -----------------M----PG--DP--L---TYREAYARLSRIAAELETGEADLDRVLPLLEEARAAYAACRERIAAV-QAALAGDWGED------EARPE----EE-DA-AQAPDPDDD-------PF 84

WP_084048039_1 ------------------------------M---TYREAYARLSRIAAELEGGEADLDRVLPLLEEARAAYAACRERIEAV-RAVLAGEWAEE------EEADA----AA-PA-LEQAATDEG--DS---PF 81

WP_022801253_1 -----------------M----T-RPET--L---SYREAYARLSRIAQELESGEADLDRVLPLLEEAREAYAACRERIEAV-RAVLAGDWAGA-------GDLP----DP-AD-PDDEDPGDD--------- 82

WP_062157410_1 -----------------M----TEPQPT------SYREAYARLSRIAAELESGEADLDRVLPLLEDARAAYAQCRERIEAV-RAVLAGDWADG------SDADE----TD-TDNTDDAE------------- 80

WP_058975274_1 -----------------M----TEPQPT------SYREAYARLSRIAAELESGEADLDRVLPLLEDARAAYAQCRGRIEAV-RAVLAGDWADD------SDADE----TD-TDDAE---------------- 77

WP_046843246_1 -----------------M----TEPQPT------SYREAYARLSRIAAELESGEADLDRVLPLLEDARAAYAQCRERIEAV-RAVLAGDWADG------EDGSD----ED-EE------------------- 74

WP_039685985_1 -----------------M----SADTSP------TYRDAYARLSRIAAELETGEADLDRVLPLLEEAREAYAQCRERIEAV-RAVLAGDWADG------VEADG----DE-EE------------------- 74

WP_029478945_1 -----------------M----SADTPT------AYRNAYARLSRIAAELETGEADLDRVLPLLEEAREAYAQCRERIEAV-RAVLAGDWADG------GEAEE----DE-E-------------------- 73

WP_029480803_1 -----------------M----SADIQT------SYRDAYARLSRIAAELETGEADLDRVLPLLEEAREAYAQCRERIEAV-RAVLAGDWADG------GEPEE----DE-E-------------------- 73

WP_034385787_1 -----------------M----PDPETP--L---TYREAYARLSRIAAELEAGEADLDRVLPLLEEARAAYAACRARIAAV-QAVLAGDWAGG------EEDGP----AQ-ED------------------- 75

WP_034342578_1 -----------------M----------------TYETQYQTLRRIAREMENPELPLDELVALLKEATEAYAACKTHLDAA-QEALEA------L----EDQAG---------------------------- 60

**deino9.00842, Biotin carboxyl carrier protein AccB**

WP_056295738_1 MHPDDLKKILDALSAADVREFALKTGSFDLALKRG--PQYA--A---APVATP---------QMQ-AP-QVYAAP--AP-----APLPAAPM-----PQASAADTAP-TP-APP-AP----AA-S-------DAPATTS-PEAA---APA-SAGTPVKAPIVGTFYASSSPDAPAYVKVGDKVEAGQVLCIIEAMKLMNE 150

WP_034346183_1 MDPRDLKKILDALSDAEFSEFSLKTSEYEISLKRG--AEQV----L-VQAPVQ---------TVA-AP-----QP--V-----VVQQPAVQA-----P--VVQSAPA-A--QPV-------AE-S-------APAAQAA-PA-----EDT-SKLVPVKAPIVGTFYSSSSPDATPYVQVGDRVQVGQVLCIVEAMKLMNE 139

WP_064013947_1 MNPEDLKRILDALSAADVREFSLKTGSFALDLKRG--PQAM--G---GPAFAP-SAS-----APS-GP-AAPQPP--A-----FAPAPAASA-----P--SGPSAPA-DV-AP--TP--A-AT-P-----A-APEAPTA-PA-----KAV-SAGTPVKAPIVGTFYASSSPDAAPFVKIGDSVSAGQVLCIIEAMKLMNE 150

WP_015234079_1 MDPRDLKRILDALIAADVREFGLKTGEYELNVKRG--PD----G---APVVYG-------------VP-APVQAP--V-----SAPVSAPVA-A---P----------VP-TPV-------SV-P-T---A-NAEASAV-PA------AA-ASGTPVKAPIVGTFYAASSPEMPPFVKVGDRVEVGQVLCIIEAMKLMNE 134

WP_102128294_1 MNPDDLKKILDALSAADVREFSLQTGSYTLDLKRG--PVAM--G-G-GTSATS-GGL-GG--GAPSAP-VPASQP--A-----PAATSAPAI-----P--PASAQTP----APV-------AA-P-----T-PEPAAPA-PS-T---PAR-SAGTPVKAPIVGTFYAASSPDAPAFVKVGDTVQAGQVLCIIEAMKLMNE 151

WP_013615111_1 MNPDDLKKILDALSYADVREFSLKTGSFDMELRRG--PLAS--G-G-SSLPMS-GAA-SH--AAP-AS-APMAAP--A-----PASAPVPAA-----P--AEASAAS-EV-APV-------PA-A-----A-SPAEATA-PA-A---APA-SAGTPVKAPIVGTFYAASSPDAAPYVKVGDRVEEGQVLCIIEAMKLMNE 152

WP_084049870_1 MNPDDLKKILDALSAADVREFSLQTGSFALDLKRG--PQAF--A---GPA----PVA-----APH-TPTVMSAAA--S-----FQPPAPSSA-----A--PAP-EAP--T-APA-TP--VLSE-A-----A-TPSAPAAAPA-----APAKSAGTPVKAPIVGTFYTASSPDAAPYVKVGDTVSAGQVLCIIEAMKLMNE 150

NP_293844_1 MNPDDLKKILDALTHADVREFALKTGSFDLALKRARRRSPQ--C---LPRSAP---------QLS-YP-QMPPLP--QPMMGGFAPMPAPQA-----P--AAPSAPA-AP-APE-AA----PA-P-------AAPAEAA-PAAASA-PVA-SAGTPVKAPIVGTFYSASSPDAAPYVKVGDRVESGQVLCIIEAMKLMNE 157

WP_019588141_1 MNPDDLKKILDALSVADVREFSLKTGSFALDLKRG--PQAV--S---GPAF---ALS-----APA-VP-MSAPTP--S-----FQPSAVSPE-----A--ASVQSPV-V--SEA-AP--T-PT-P-----A-ADPAPAA-PT-----KSA-SAGTPVKAPIVGTFYSASSPDAPAYVKVGDQVQPGQVLCIIEAMKLMNE 148

WP_012692274_1 MNPEDLKKILDALSVADVREFSLSTGSFAMDLKRG--PQAV--S---YPSPAP-S-A-PA--PIF-MP-GAGHAP--A-----APAAPAPVA-----P--A---TDA----APA-LPA-A-AT-P-------APEVEAA-PA---PVKPV-SAGTPVKAPIVGTFYASSSPDAPPYVKVGDTVQAGQVLCIIEAMKLMNE 149

WP_019011092_1 MNPEDLKRILDALSAADVREFSLKTGSFALDLKRG--PQAM--G---GPAFAP-SANFSSPNGSS-GP-AAPQPA--A-----FAPAPSAST-----P--SGPAAPA-EV-APAASA--P-AA-P-----A-TPETPAA-PA-----KAA-SAGTPVKAPIVGTFYASSSPDAAPFVKIGDSVSAGRVLCIIEAMKLMNE 157

WP_022800658_1 MNPEDLKKILDALSLADVREFSLTTGSFAMDLKRG--PQAF--A---APV----APA-PA--GFA-MPHLPA--P--T-----YA-MPAPAA-P---S--APTPAPA----AQS-APA-A-AA-PAAEKPAEAPA-APA-PE---A-KPA-SKGTPVKAPIVGTFYSASSPDAPPYVKVGDHVTAGQVLCIIEAMKLMNE 154

WP_029481492_1 MNPDDLKQMLDALKAADVREFSLQTGSFSLDLKRG--PQA---G---GNAAPS---------APA-AP-QPAFEA--A-----FDSAPTPPA-----P----------VS-APA-SE----AA-P-----A-APAASAP-AA-----PAA-ASGAPVKAPIVGTFYASSSPDAAPYVKVGDTVTAGQILCIIEAMKLMNE 139

WP_103128605_1 MNPDDLKKILDALSVADVREFSLKTGSFALDLKRG--PQAV--S---GPAF---APS-----APA-AP-MPAPAPMPS-----FQPSAAPAE-----T--AGAPAAS-MGESSS-AP--A-PATP-----T-ADPAPAA-PA-----KSA-SAGTPVKAPIVGTFYSASSPDAPPYVKVGDRVEPGQVLCIIEAMKLMNE 153

WP_034385307_1 MNPDDLKKILDALSAADVREFSLTTGSFALDLKRG--PVAV--S---SPAAVQ-SVQ-----SAP-LP-----AP--S-----FQPAPAPVS-----A--PAPAAPA-AE-LPA-AP--A----P-----A-AEQTAEA-P------KST-SKGTPVKAPIVGTFYAASSPDAPPYVKVGDTVSPGQVLCIIEAMKLMNE 144

WP_029476255_1 MNPDDLKQMLDALKAADVREFSLKTGSFSLDLKRG--PQAM--G---GPASQP---------SAA-AP-APRMET--A-----FDAAPES-N-----P----------NP-APS-AP----LA-S-----A-APAESAA-PA-----PSA-SKGAPVKAPIVGTFYASSSPDAAPYVKVGDTVAAGQILCIIEAMKLMNE 139

WP_058975216_1 MNPNDLKQILDALTYADVREFSLRTGSFDLSLKRG--PQAF--A---APTPMP-TPG-PA--PVA-AP-MPA--P--A-----FAPMPAPAMPAHAAP--APQVQDS----APA-APAQA-AA-P-------APA-AEV-PA---E-KPA-SKGTPVKAPIVGTFYASSSPDAAPYVKVGDTVAAGQVLCIIEAMKLMNE 155

WP_062159157_1 MNPNDLKQILDALTYADVREFSLRTGSFDLSLKRG--PQAF--A---APAPMP-ATG-PA--PVA-AP-MPA--P--A-----FAPMPAPAM-----P--APQVQDS----APA-APAQA-SA-P-------APTPAEA-PA---E-KPA-SKGTPVKAPIVGTFYASSSPDAAPYVKVGDTVAAGQVLCIIEAMKLMNE 151

WP_046843270_1 MNPNDLKQILDALTYADVREFSLRTGSFDLSLKRG--PQAF--A---APAPMP-TPG-PA--PVA-TP-MPA--P--A-----FAPMPAPTM-----P--APQVQDS----APA-QA--A-PA-P-------APTPAEA-PA---E-KPA-SKGTPVKAPIVGTFYASSSPDAAPYVKVGDTVAAGQVLCIIEAMKLMNE 149

WP_099748343_1 MNPNDLKQILDALTYADVREFSLRTGSFDLSLKRG--PQAF--A---APAPQP-APG-PA--PLA-AP-TPAGAP--A-----FAPMPAPSA-----P--APQPQDN----APA-APA-Q-AA-P-------AP---EA-PA---E-KPA-SKGTPVKAPIVGTFYASSSPDAPPYVKVGDTVAAGQVLCIIEAMKLMNE 149

WP_078300771_1 MNPNDLKQILDALTYADVREFSLRTGSFDLSLKRG--PQAF--A---APAPQP-APG-PA--PLA-AP-TPAGAP--A-----FAPMPAPSA-----P--A-QPEAS----APA-APA-Q-AA-P-------AP---EA-PA---E-KPA-SKGTPVKAPIVGTFYASSSPDAPPYVKVGDTVAAGQVLCIIEAMKLMNE 148

WP_104991371_1 MNPDDLKKILDALSQADVREFSLTTGSFALDLKRG--PQAA--G---MSALASAPAP-----APH--A-----AP--S-----FQPLAASSA-----A--PASAEAPSAP-APA-AS--A-NA-P-----A-ETPTPAASPA-----KPA-SAGTPVKAPIVGTFYSASSPDAAPYVKVGDTVSAGQVLCIIEAMKLMNE 149

WP_034357296_1 MNPDDLKKILDALSLADVREFSLKTGSFALDLKRG--PQAM--G---GPAF---APS-----APA-AP-MAPAAP--S-----FQPPATSTE-----G--AAPAPA-----ATE-AP--T-PA-P-----A-AEATPAA-PA-----KSA-SAGTPVKAPIVGTFYSASSPDAPAYVKVGDTVSAGQVLCIIEAMKLMNE 146

WP_034403926_1 MNPEDLKKILDALKAADVREFSLTTGSFALDLKRG--PQVA--G---MAA----PAP-----APT-LP-----AP--S-----FPPPAASSA-----APAPAPAEAP-AP-APV-TP--E-ST-P-----A-EPPAAAAPPA-----KPA-SAGTPVKAPIVGTFYAASSPDAPPYVKVGDTVQPGQVLCIIEAMKLMNE 147

WP_027459484_1 MNPDDLKKILDALKAADVREFSLTTGSFALDLKRG--PQVA--G---MAA----PAP-----APT-LP-----AP--S-----FPPPAASSA-----A--PAPAEAP-AP-APV-TP--E-ST-P-----A-EPPAAAAPPA-----KPA-SAGTPVKAPIVGTFYAASSPDAPPYVKVGDTVQPGQVLCIIEAMKLMNE 145

WP_103311850_1 MNPDDLKQILNALSAADVREFALRTGSFDLSLKRG--PQAA-SGATSGPAPVA---G-PA--PLA-AP-SAPAGP----------GAPTPAV-----G--AASDAAP----QAA-QA--T-DL-P-I---T-PPKVPTP--------------GTPVKAPIVGTFYASSSPDAPAYVKVGDTVAAGQVLCIIEAMKLMNE 144

WP_017869709_1 MNPDDLKKMLDALTHADVREFTLKTGSFDLALKRG-----------------P---------QAF-AP-AAPSAP--Q-MTGSFAP--APAV-----P--AAPSAPA-AP-APE-AT----PA-A-------KPAEEAA-PVA----PAA-SAGTPVKAPIVGTFYSASSPDAAPYVKVGDRVEAGQVLCIIEAMKLMNE 139

WP_011529612_1 MNPDDLKKILDALSAADVREFSLTTGSFALDLKRG--PVALGAA---APAASL-SAA-----PPP-VP-----AP--S-----FQPAPVPAS-----T--PAAAAPA-AE-APA-TP--A----P-----A-AEAAPEA-PA-----RSA-STGTPVKAPIVGTFYAASSPDAPPYVKVGDKVQPGQVLCIIEAMKLMNE 147

WP_107137653_1 MNPDDLKQILSALTSADVREFALRTGSFDLSLKRG--PQAA------GPAPLAPPQA-PQ--PLS-AP-A----P----------AAPTPAA-----P--APLAETP----APA-AA--A-GA-P-T---P-APEAPAAAPA-----PTA-SKGTPVKAPIVGTFYSASSPDAPAYVKVGDTVQAGQVLCIIEAMKLMNE 146

WP_014685890_1 MHPDDLKKILDALSAADVREFALKTGSFDLALKRG--PQAV--S---APLAAP---------QLP-AP-QAYAAP--APLAA--APLAQPQV-----P-ATPADVTP-AA-SPA-VS----EA-P-------ASPSTEA-PAPS---APA-SAGTPVKAPIVGTFYASSSPDAAPYVKVGDTVAAGQVLCIIEAMKLMNE 152

WP_056295738_1 IEAETGGVVRQILVKNAEPVEYGQTLFVIE-- 180

WP_034346183_1 IESEVAGIVRKILVNNAQPVEYGQDLFLIEPA 171

WP_064013947_1 IEAETGGVVREILVKNADPVEYGQTLFIIE-- 180

WP_015234079_1 IESETAGTVKQILVKNAEPVEYGQTLFVIE-- 164

WP_102128294_1 IEAELGGTVREILVKNAEPVEYGQTLFVIE-- 181

WP_013615111_1 IEAESSGVVREILVSNGEPVEFGQTLFIIE-- 182

WP_084049870_1 IEAEVSGTVREILVKNAEPVEYGQTLFIIE-- 180

NP_293844_1 IEAEQSGVIREILVKNAEPVEYGQTLFMIE-- 187

WP_019588141_1 IEAEVGGTVREVLVKNAEPVEYGQTLFIIE-- 178

WP_012692274_1 IEAEQGGTVREILVKNAEPVEYGQTLFIIE-- 179

WP_019011092_1 IEAETGGVVREILVKNADPVEYGQTLFIIE-- 187

WP_022800658_1 IEAETSGVIREISVQNAEPVEFGQTLFIIE-- 184

WP_029481492_1 IEAEMGGVVREILVKNAEPVEYGQTLFLIE-- 169

WP_103128605_1 IEAEVGGTVREILVKNAEPVEYGQTLFIIE-- 183

WP_034385307_1 IEAEVGGTVREILVKNAEPVEYGQTLFIIE-- 174

WP_029476255_1 IEAEAGGVVREILVKNAEPVEYGQTLFLIE-- 169

WP_058975216_1 IEAEQGGTIREILVKNAEPVEYGQTLFIIE-- 185

WP_062159157_1 IEAEQGGTIREILVKNAEPVEYGQTLFIIE-- 181

WP_046843270_1 IEAEQGGTIREILVKNAEPVEYGQTLFIIE-- 179

WP_099748343_1 IEAEQGGTVREILVKNAEPVEYGQTLFIIE-- 179

WP_078300771_1 IEAEQGGTIREILVKNAEPVEYGQTLFIIE-- 178

WP_104991371_1 IEAETGGVVREILVKNAEPVEYGQTLFIIE-- 179

WP_034357296_1 IEAEVSGTVREILVKNAEPVEYGQTLFIIE-- 176

WP_034403926_1 IEAETGGVVREILVKNAEPVEYGQTLFIIE-- 177

WP_027459484_1 IEAETGGVVREILVKNAEPVEYGQTLFIIE-- 175

WP_103311850_1 IEAETGGTVREILVKNAEPVEYGQTLFIIE-- 174

WP_017869709_1 IEAEQSGVIREILVKNAEPVEYGQTLFMIE-- 169

WP_011529612_1 IEAEVGGTVREILVKNAEPVEYGQTLFIIE-- 177

WP_107137653_1 IEAELGGTVREILVKNAEPVEYGQTLFIIE-- 176

WP_014685890_1 IEAETGGVVRQILVKNAEPVEYGQTLFVIE-- 182

**deino9.01337, uncharacterized protein**

WP_013614920_1 ----------MRAYRGIVKDGRVEL-VGGELPEGTQVTVTVGEPELLLATLLHWLRRGK-RIRISLGP-V-AGM-----GL-LR-LRRR---G------------QRHG--- 73

NP_294360_1 ------MERRVRAYPGIVENGVVVV-IGARLPEGTAVTVTVGETELLRARISNVIRRAP-KVRIRLKPQS-PRL--A--------LEKC---------------PPEPD-ED 77

WP_017870115_1 ----------MRAYPGIVENGVVVV-IGARLPEGTAVTVTVGETELLRARISNVIRRAP-KVKIRLKPQS-PRL--A--------LEQC---------------PPEPD-AD 73

WP_015235695_1 ----------MRAYRGVVVNGVVEL-QGVRLPEGTVVTVTVGEAELLRAKIASALRRPK-RVKIRLKP-AAPGL--G--------FEAP-E-G-ADVTP--E--IFARE--- 79

WP_062158557_1 ----------MKAYKGVVENGVVVI-IGGRLPEGTVVTVTVGEGELLRARITNVLKRPR-KVKVRLKP-N-TGL-----AT-SG-LGGA---G-GGI--------LGSD-D- 77

WP_013555652_1 MVPEEEF---VKAYKGIVEDGVVVLVEGVQLPEGTIVTVTVGEAELLRARITNALRGKR-KVKVRMKPVT-PGL-VM--NA----APTE-E--------------------- 78

WP_104991139_1 ----------MRAYKGIVEGGVVVL-VGARLPEGTVVTVTVGEAELLRARITSALTR-R-RVRVRIKP-L-PGL--A--AE-SA-LPGSVPQG------------RGED--- 76

WP_034402275_1 ----------MRAYKGIVEGGVVVL-VGARLPEGTVVTVTVGEAELLRARITSALTR-R-RVRVRLKP-L-PGL--A--AE-SA-LPGA-AQG------------GGED--- 75

WP_058976183_1 ----------MKAYKGVVENGVVVI-VGGRLPEGTVVTVTVGEGELLRARITNVLKRPR-KVRVRLKP-T-PGLAMQGLAM-EG-LVQS---GPGGI--------LGSD-D- 83

WP_046842829_1 ----------MKAYKGVVENGVVVI-VGGRLPEGTVVTVTVGEGELLRARITNVLKRPR-KVRVRLKP-T-PGL-----AM-EG-LVQS---GPGGI--------LGSD-D- 78

WP_045233674_1 ----------MRAYRGVVVNGVVEL-QGARLPEGTVVTVTVGEGELLRATIANALRRPK-RVRIRLKP-AAPGL--G--------FEAP------DLVPDVE--VVRSE--- 78

WP_102127709_1 ----------MRAYKGIVEDGVVVL-IGTRLPEGTVVTVTVGEAELLRARITSALKRPR-KVRVRLKP-T-PGL-VA--EA-AL-AGGV---N-----------DTGND--- 76

WP_029483386_1 ----------MRAYKGIVENGVVVV-LGTRLPEGTVVTVTVGEGELLRARITNVLKRPR-KVKVRIKP-N-VGL-----AA-TG-TDGA---GLMGANLTLTLLPSGVD-D- 86

WP_051668526_1 ----------MRAYKGIVENGVVVV-LGTRLPEGTVVTVTVGEGELLRARITNVLKRPR-KVKVRIKP-N-IGL-----AA-TG-TDGT---GLMGPRLTLSGSPSGVD-D- 86

WP_034385106_1 ----------MRAYKGIVENGVVVL-IGARLPEGTVVTVTVGEAELLRARITSALTRSR-KVRIRLKP-T-PGL-VA--EA-AA-LGSS---G-----------EAGND--- 76

WP_107136334_1 ----------MKAYKGVVENGVVVI-IGGRLPEGAVVTVTVGEGELLRARITNVLKRPR-KVRVRLKP-T-PGL-----AT-SA-LGSL---A-APH--------PGAD-D- 77

WP_052195445_1 ----------MRAYKGVVENGVVVV-LGTRLPEGTVVTVTVGEGELLRARITNVLKRPR-KVKVRLKP-N-VGL-----AA-TG-TDGA---GLVGTRLTLGGPPSGAD-D- 86

WP_011530768_1 ----------MRAYKGIVENGVVVL-IGARLPEGTVVTVTVGEAELLRARITSALNRPR-KVRIRLKP-A-PGL-VA--EAVPA-LGGP---G-----------EGGKD--- 77

WP_034352016_1 ----------MRAYKGIVEDGVVVL-IGARLPEGTVVTVTVGEAELLRARITSALNRPR-KVRIRLKP-T-PGL-VA--EAVPA-LGGL---G-----------NGGND--- 77

WP_084049753_1 ----------MRAYKGIVENGVVVL-IGARLPEGTSVTVTVGEAELLRARITNALNRPR-KVRVRLKP-T-PGL-LA--EA-AL-LTAV---G-----------EVGE---- 75

WP_019585128_1 ----------MRAYKGIVESGVVVL-IGARLPEGTVVTVTVGEAELLRARITSVLKRPR-KVRVRLKP-V-PGL-VA--QA-VP-AGGV---G-----------DAGE---- 75

WP_103128818_1 ----------MRAYKGIVESGVVVL-IGARLPEGTVVTVTVGEAELLRARITSVLKRPR-KVRVRLKP-V-PGL-VA--EA-VP-AGGV---S-----------EAGE---- 75

WP_022801773_1 ----------MRAYRGVVENGVVVL-VGARLPEGTVVTVTVGEGELLRAKITDVLRRSP-KIRIRLKP-D-PGL--A--------MQGA------PLDP-AG--GPETE--- 76

WP_019009720_1 ----------MRAYKGIVEDGVVVL-IGARLPEGTVVTVTVGETELLRARITSALKRPR-KVRVRVKP-T-PGL-AM--SH-WQ-LEGT---T-----------SRPPSDE- 78

WP_064014592_1 ----------MRAYKGIVEDGVVVL-IGARLPEGTVVTVTVGETELLRARITSALKRPR-KVRVRVKP-T-PGM-AM--GQ-LQ-LEGT---S-----------ARPQSDE- 78

WP_012693539_1 ----------MRAYKGIVENGVVVV-IGARLPEGTVVTVTVGEGELLRARITSVLKRPR-KIRVRLKP-T-PGL-----AM-EAGLDGS---Q-----------APGDH-D- 76

WP_103309863_1 ----------MRAYKGIIENGVVVV-IGARLPEGTVVTVTVGEGELLRARIVGVLKRPR-KVRIRLKP-S-PGL-----AL-QA----T---E-----------MPGND--- 71

WP_014684236_1 ----------MRAYKGIVENGVVVV-IGARLPEGTVVTVTVGETELLRARISNVIKRSSRKVRVRVKP-S-PGL--A--------LESQ---------------LPSRD-E- 72

WP_056303667_1 ----------MRAYKGIVENGVVVV-IGARLPEGTVVTVTVGETELLRARISNVIKRSSRKVRVRVKP-N-PGL--A--------LESR---------------LPSRD-E- 72

WP_034342181_1 ----------MKAYKGVVVDGVVVL-EGVKLPDGTVVTVTVGEAELLRATISNALRIRR-NKRAKVRIKT-QPI-YA--EK----LTLE----------------------- 69

**deino9.01490, DNA-directed RNA polymerase, sigma subunit (sigma70/sigma32) RpoD**

WP_062158448_1 MADSTTVRTRKKV-----------D----------AESGEPKAA--------ARARA-RVA---PGTTPKPVT---TATSPL-P----AK----PAPAKKVAP--KVEAA---PAV-QATPAEPAPSV---------------AAPVVATETVESPVKKPAAKKAAPKDDADK-----PAKKPAAKKTAAKA-------- 121

WP_046842731_1 MADSTTVRTRKKI----D-----------------AESGEPKAA--------ARARA-RVA---PGTTPKPVT---TATSPM-P----AK----PAAKKAA-P--KADAPEL-AQP-APEAAAPVVAA----------------ET-------VEAPKKAPAKKAAPKVAKAD-----APEKPAKK---PAA------K- 112

WP_058976273_1 MADSTTVRTRKKI----D-----------------AESGEPKAA--------ARARA-RVA---PGTTPKPVT---TATSPM-P----AK----PAAKKAA-P--KADAPEL-AQP-APEAAAPVVAA----------------ET-------VEAPKKAPAKKAAPKAAKAD-----APEKPAKK---PAA------K- 112

WP_015235107_1 ----------------------------------------------------------------------------------------MS----EADTRKTRT----------RAA-SEGKAKPART--------------------------KTPSDGAAKKSAAPK----------TPKAPKASAAKAAA------K- 54

WP_019009695_1 MADSTKPRTRSKVSAAAT--------LAAPEESRIKTPDKPRTR--------TQPRT-RAV---MPGTEGGPEAVQVAPVDT------------APTPDQTKP--VPKKAVA-PRA-ADQDTGAGAAA-----------------P-------AKAAPKKAVAKPKAAAPADGEDDSQGGSASEANAPKPAAKAKAAPK- 139

WP_084049792_1 MAEPTRARARSKV-----------P----------ATPQQAPTD--------APELG-GIK---TPAQPRTRA---AARPPK-PLA--AT----GADAGVTTP--EVPVA---PAT-EDAAKVP-----------------------------KSPARAAKA----PAADGEP-----APKKAAPKKAKAAP-------- 105

WP_029480413_1 MAESTKASPEQAT--------------DIPEAAPQAAEKKPRTR--VGG---AKPAA-ARA---APAEEVAAK---AAPVKK------------PAAKKA-------------ATA-KTDGDAPVKAA-----------------A-------KKPAAKKAVAEADAAEPKAKAPAKKAAPKKTATAAEATS------D- 117

WP_043801219_1 MTNDPKPRTRSKVPATPP------A--EVAAESKIKTPDKPRTR--------TQPRV-KPALIGAAPATGDVT---ATPSGA----D-LA----PGSVSGVGA--ASPAP---APA-PAKKAAPKKAS------AASATDPA---A-------EKPEKKAAPKKAAAPKTPKAA----APKAAAGEGP----------A- 134

WP_056302198_1 MTNDPKPRTRSKVPATPP------A--EVVAESKIKTPDKPRTR--------TQPRV-KPALHSAASAAGDVT---AAPAGA----D-LA----PDSVAGVGA--SSPAP---APV-PAKKAAPKKAS------AE-----G---A-------EKPEKKAAPKKAAAPK---------APKAAAGEGP----------A- 124

WP_064014742_1 MADSTKPRTRSKVSAAAT--------LAAPEESRIKTPDKPRTR--------TQPRT-RAV---MPDAESGAE---AVPVAA-EVAP-APAKAKPAPKKAAAP--KTEAAID-ADP-DSAEATAADVA-----------------P-------AKPAPKKAAAKPKAAKAAPATTDDGAAEADAGSDAGAEA------D- 140

WP_013557469_1 MTEKKIAARARKP----------------------KAEQDPDAA--------------AAP---APAASAPKT-R-KKPAVS------------TDDVNASVP--EPAQV---AEA-SASDVTPTPET-------------------------PKPTRKRAASSAVD-----------APAKAEKPAKAKAA-------- 97

WP_034407045_1 MAEPTRARARTKV----------------------SAPAAPAPE-------------------------GTDT---AAPTAS-P----------PKPRRAAKP--KAAAK---AATPAPEAEAPAATA-----------------K-------AAPKKAATRKKAAPAP-------EMAPAEPAESAP-AAA------P- 95

WP_017869017_1 MAEPVKSRVRPKVKPVAD---------EAPAESKIITPDKPRTRRKVADPATTGAAA-SAA---APQASADTA---QAPAAA-KPKA-APKKA-PAKKAATKP--ADAENVDTATV-NAADAAPVDAATPARKKAQSKAAGGAAEP-------SKPARKAAPKKAAQDTPAPAEDTEAAPVKKAAQDTSAPA------ED 165

WP_013615127_1 MAETKTAKTKKT----------------------------------------------------------------AAPAEA------------AAEKPARKP----------AAR-KPAARKPAEAG------AA--------------E--KPAAKKEAAKKPASKK------------------------------- 60

WP_034352241_1 MAEPTRARARSKVPASGTPVTGTPAPADSPEESKIKTPAQPRTR--------TQPRAAKTA---VTGETAAP----ARPVKA------------PATPKASKA--AP----T-GDD-PAQEAAPAKAA-----------------P-------KKAAPKKAAAKTAPASPED--------------------------K- 114

WP_034387103_1 MAEPTRARARSKV----------------------PATGTPVTG--------------------APVTADASE---ETPIKT-P----AQ----PRTRTAKTA--KAA-----KPP-AAEDTAPEAAP-----------------K-------KAPPKKAAPKKATAKP---------TPEEDS---------------- 89

WP_027459433_1 MAEPTRARARTKV----------------------SAPAAPAPE-------------------------GTDT---AAPAAARP----------PKPRRAAKP--KAAAP-------APEAEAPAPTA-----------------K-------AAPKKAATRKKAAPAP-------EMAPAEPAESASAAAA------P- 93

WP_011529620_1 MAEPTRTRARSKA-------------------------PAPAPQ--------VSGAS-------VPADTAEES---KIKTPA----------Q-PRPRTQTRA--GKTA----KAE-RPTAEAPVQAA------DP---------K-------KSAPKKAASKKTAAKA---------APASAEESAT----------D- 97

WP_103309804_1 MADSTKTRSKVGATPRVS------A--SAAAPA--KAADAPKTK---AT---TEPKA-KPS---AKPEAKTKD---SAPRTG------VK-AR-PARAGAAQA--APSAA---LTE-EQPTAAPAVKA-------------------------RKASKAEAAQTEAVDT---------VSVDPVASPATPTP-------- 121

WP_012693512_1 MADSTKPRTRTKV-----------A----------ATADAPKAE--------PVINT-PDK---PRTRVKAVG---TAVAEQ-PVTQEAK----AAP-KKAAA--DKTAK---PAA-KKAAAKPAA---------------------------DQPKTVEAASK--PAKTADK-----AAKAPAKKAAKAQG-------- 110

WP_104991102_1 MSEPTRARARTKA----------------------SAPAAPAPE-------------------------VAET---VAPAAA-P-----------KPRKAAKP--KAAAA---PAT-APEAEAALT---------------------------PAPKKAPARKKAAPAP-------E--AAAPTEDAT-PTA------A- 88

WP_103130723_1 MAEPTKARTRSKV---------------------------------------------------PASSAGVTE---GGPAEN-P----VK-AS-PAPRAPARPAAQNGEPDQ-TAA-AAHEVTPRKMA-----------------P----R--KATAKSASAKKAASQT---------DPAPNTDAGAEGAP-------- 97

WP_019585453_1 MAEPTKARTRSKV---------------------------------------------------PASSAGVTA---DGPAES-L----VK--T-PTSRAPALP---------------AEEVTPREAA-----------------P----R--KATAKSASAKKAAAST---------DPAPDTDAGAEGAP-------- 83

WP_078304160_1 MADSPTVRTRKKVEAPTD-----------------AAAPTPKPA--------VRARA-RVA---APREDGSDT---SAPTAA-PKAA-VK----PAAKPAAKP--AAPAAET-APA-QPAADQPAVTG----------------AP-------KAPAKKAAPKAAKPAKADTDTTDEAAPKKPARKAAEPKA------A- 128

WP_099747986_1 MADSPTVRTRKKVEAPTD-----------------AAAPTPKPA--------VRARA-RVA---APREDGSDT---SAPTAA-PKAA-VK----PAAKPAAKP--AAPAAET-APA-QPAADQPAVTG----------------TP-------KAPAKKAAPKAAKPAKADTDTTDEAAPKKPARKAAEPKA------A- 128

WP_107136096_1 MADSP-VRTRKKA--------------DAPDAAS-AAPGAPKTA--------ARARA-RVP---AGAAAPAEP---TAADAA-PTPV-AA----PAKKAAK-P--AAKA----KAA-SPEASAPAPKA-------------------------PKAEKPKATKKAAPASAPEG-----VAEAPALPEPAPKA------G- 118

**deino9.03407, Ribosomal protein S14 RpsN**

WP_027481541_1 ------------------MAKKSKIARQKQREQLVEKYAAKREAMKAAGDYQGLATLPRNASPTRLHNRCELTGRPRGYIRFFGVSRIVLREKVHKGELPGVKKSSW 89

WP_011530979_1 ------------------MANTSKVVKAARGHKF------------AVQNY----------------NRCSRCGRARGYYRFFGMCRICIRELAHKGELPGVKKASW 61

WP_012693992_1 ------------------MANTSKVVKAARGHKF------------AVQNY----------------SRCSRCGRARGYYRFFGMCRICIREMAHKGELPGVKKSSW 61

WP_014685985_1 ------------------MANTSKVVKAERGHKF------------AVQNY----------------NRCSRCGRARGYYRFFGMCRICIREMAHKGELPGVKKSSW 61

WP_019012086_1 ------------------MANTSKVVKAERGMKF------------AVQNY----------------NRCSRCGRARSYYRFFGLCRICIRELAHKGELPGVKKSSW 61

WP_022800309_1 ------------------MANTSKVVKAARGHKF------------AVQNY----------------NRCSRCGRARGYYRFFGMCRICIREMAHKGELPGVKKASW 61

WP_026332626_1 ------------------MANTSKVVKAARGSKF------------AVQNY----------------NRCSRCGRARGYYRFFGMCRICIRELAHKGELPGVKKASW 61

WP_027460639_1 ------------------MANTSKVVKAQRGSKF------------AVQNY----------------NRCSRCGRARGYYRFFGMCRICIRELAHRGELPGVKKASW 61

WP_029476463_1 ------------------MANTSKVVKAARGHKF------------KVQDY----------------SRCSRCGRARSYYRFFGMCRICIREMAHKGELPGVKKASW 61

WP_029483704_1 ------------------MANTSKVVKAARGHKF------------KVQDY----------------SRCSRCGRARSYYRFFGMCRICIREMAHKGELPGVRKASW 61

WP_034356044_1 ------------------MANTSKVVKAERGHKF------------AVQNY----------------NRCSRCGRARGYYRFFGMCRICIRELAHKGELPGMKKASW 61

WP_039682873_1 ------------------MANTSKVVKAARGHKF------------AVQNY----------------SRCSRCGRARGYYRFFGMCRICIREMAHKGELPGVRKASW 61

WP_046843203_1 ------------------MANTSKVVKAARGHKF------------AVQNY----------------NRCSRCGRARGYYRFFGLCRICIREMAHKGELPGVKKASW 61

WP_055362781_1 ------------------MANTSKVVKAARGHKY------------AVQNY----------------SRCSRCGRARGYYRFFGMCRICIREMAHKGELPGVKKSSW 61

WP_102127883_1 ------------------MAKTSKVVKAERGSKF------------AVQNY----------------NRCSRCGRARAYYRFFGLCRICIRELAHKGELPGVKKSSW 61

WP_103312538_1 ------------------MANTSKVVKAARGHKF------------AVQNY----------------SRCSRCGRARGFYRFFGMCRICIREMAHKGELPGVKKASW 61

WP_051964318_1 ------------------MANKGKTI-SHNSKKF------------AVQNY----------------SRCQRCGRARGYYRFFGLCRICLREMAHRGELPGVKKSSW 60

WP_017869803_1 ------------------MANKSKLAKQKQREKTVAKYAAKRAELKAAGDYYGLSQLPRDASPTRLHNRCEFTGRPRGYVRFFGVSRIVLREMAHRGELPGVKKASW 89

NP_295832_1 MPLRPETSAQKNLPRRNSMANKSKLAKQKQREKTVEKYAAKRAELKAAGDYYGLTQLPRDASPTRLHNRCEFTGRPRGYVRFFGVSRIVLREMAHRGELPGVKKASW 107

WP_015236799_1 ------------------MAKTSKIAKQKQREKMVAKYAEKRAALKAAGDYQGLAELPRDASPVRLRNRCEITGRPRGVSRFFGVSRIVMREKAHKGELPGVKKSSW 89

WP_013558060_1 ------------------MAKKAKIQRQKHREKMVAKYAAKRAELKAAGDYVGLAQLPRDASPTRLHNRCALTGRPRGYIRFFGVSRIVLREMAHAGELPGVKKASW 89

WP_013614186_1 ------------------MAKKSKIAKQKQREQKVAKYAAKRAEMKAAGDYYGLSQLPRDASPTRLHNRCEFTGRPRGYIRHFGVSRIVMREMAHRGELPGVRKASW 89

**paen9.00611, DNA-binding transcriptional regulator, FrmR family**

WP_081758827_1 -----------------------------------------------------MP------V-----ST--NE-K-----EH--AG----HS-------CG-------D------DCH-TSAPG----V-RKSHHSAEFKSGLTTRLNRIEGQIRGVKGMIERDTYCDDVLNQLAAVQAALNSVGKLLLEGHMKSCIIERIEAGEHEVIDELLVTVNKLMK- 117

WP_089523234_1 ---------MEIQRG------VESQPA----------------------------------A---------AQ-L-----EH--AA----EG-------CS-----N-E---------------------RRSHHSEKTKTNLISRLNRIEGQVRGIKGLIERDTYCDDVLNQISSIQSALNGVGKLLLEQHLKSCVVERLQEGDSTIIDELMVTMNKLIR- 117

WP_082655008_1 MSADNVEIES---------------------------------------------------A---------NE-R--S--AS--DP----EC-------CS-H-EGT-A---------------------RKSHHSDKVKNSLINRLNRIEGQIRGIKGLIEKDTYCDDVLNQIASVQSALNGVGKLLLENHMKSCVVERIQEGDMDVLNELMITMNKLIK- 119

WP_010273254_1 ---------MTHELGGYNTANTENNLTEAA-------------------------------V---------QQ-T-----ES--RE----EC-------C--S---T-E---------------------RKSHHSEKAKKSLIHRLNRIEGQIRGVKGLIEKDTYCDDVLNQISSIQSALNGVGKLLLEHHMKSCVIERIQEGDEEVIDELLTTVNKLMK- 126

WP_082867725_1 ---------MDIRREGVIR------------------------------------------M---------EE-QIHE--ST--SH----ES-------CG-T---D-G---------------------RKSHHSDKTKSNIISRLNRIEGQIRGVKGLIEKDTYCDDVLNQISSVQSALNGVGKLLLEHHMKSCVMDRIQEGDDEVIDELLKTINKLMK- 119

WP_082034113_1 ---------------------MELETPA---------------------------------A---------SP-E-QT--NQ--DE----QN-------HD-G-R-D-H---------------------QHSHHSEKTKTNLTNRLNRIEGQVRGIKGLIERDAYCDDVLNQIASVQAALNGVGKLLLEEHMKSCVVEKIQEGDLGVINELLTTINKLMK- 116

WP_082111048_1 -------------------------------------------------------------M-DK--ET--KS-A-----CL--HQ----HM--D----AG-------G------AGD-GQSME------RLSHHSDEMKANLIRRLNKVEGQIRGVKAMIEKDTYCDDVLTQIAAAQSALNGVGKLLLEGHMKSCIVDRIQAGEHEVIDELLITVKKLLK- 117

WP_099518604_1 -------------------------------------------------------------M-DK--ET--KS-A-----CL--HQ----HV--D----SG-------G------AGD-DQSME------RLSHHSDDMKANLIRRLNKVEGQIRGVKAMIEKDTYCDDVLTQIAAAQSALNGVGKLLLEGHMKSCIVDRIQAGEHEVIDELLITVKKLMK- 117

WP_005550465_1 -----------------------------MSNERIDEQVDDV-------------------L---------DA-A-----FE--SD----DG-------CS-------S-IEDGGACH-STEGK------RKSHHSDEMKKKLIHRLNRIEGQVRGVKSMIERDTYCDDVLNQIASIQSALHSVGRLLLEGHMKSCVVERLQDGEPEVIDELLTTIHKLLK- 130

WP_045673308_1 ------------------------------------------------------------------------------------------MA-------DT-K-H-H-H---------------------QRSHHSEKTKANLSNRLNRIEGQIRGIKGLIEKDAYCDDVLNQIASVQSALNGVGKLLLEEHLRSCVVEKIQEGDLGVINELLTTVNKLMK- 99

WP_076169039_1 MS---VELET---------------------------------------------------V---------ES-T--L--AE--NA----GC-------CS-S-EEP-G---------------------RKSHHSEKEKNNMISRLNRIEGQIRGIKGLIEKDTYCDDVLNQIASVQSALNGVGKLLLEHHMKSCVIDRIQEGDTEVLDELLKTMNKLMK- 116

WP_047843212_1 -------------------------------------------------------------M---------DD-T-----IL--EA----AS-------CD----------------H-TSSNE------RKSHHSESTKRKLISRLNRIEGQVRGVKGMIEKDTYCDDVLHQIASIQSALNGVGKQLLEHHMKSCVIERISEGDHKVLDELMITVNKLIK- 109

WP_062322003_1 -------------------------------------------------------------M---------DN-T-----IL--EA----AS-------CD----------------H-TSSNK------RKSHHSESTKRKLISRLNRIEGQVRGVKGMIEKDTYCDDVLHQIASIQSALNGVGKQLLEDHMKSCVVERISEGDNEVLDELMITINKLIK- 109

WP_006207111_1 -------------------------------------------------------------M---------TT-E-----NH--VH----DA-------CAVT---S-E---------------------RKSHHSDKTKSNLISRLNRIEGQIRGVKGLIEKDTYCDDVLNQISSIQSALNGVSKLLLEHHMKSCVVERLQEGDDEVITELLTTMNKLMK- 107

WP_007128955_1 -------------------------------------------------------------M---------TT-E-----SH--TH----EA-------CAVT---S-E---------------------RKSHHSEKTKNNLISRLNRIEGQIRGVKGLIEKDTYCDDVLNQISSIQSALNGVGRLLLEHHMKSCVVERLQEGDDDVITELLTTMNKLMK- 107

WP_009592707_1 -------------------------------------------------------------M---------TT-E-----NH--VH----DA-------CAVT---G-E---------------------RKSHHSDKTKSNLISRLNRIEGQIRGVKGLIEKDTYCDDVLNQISSIQSALNGVSKLLLEHHMKSCVVERLQEGDDEVITELLTTMNKLMK- 107

WP_068617707_1 MS---VEVET---------------------------------------------------N---------QE-S--V--TL--DP----GC-------CS-S-DET-E---------------------RKSHHSDKVKSNMISRLNRIEGQIRGIKGLIEKDTYCDDVLNQIASVQSALNGVGKLLLEHHMKSCVIERIQDGDTEVLDELLTTMNKLMK- 116

WP_052020444_1 -------------------------------------------------------------M---------ED-IIVEPNTQ--TH----ES-------CD-T---D-D---------------------RKSHHSEKTKKNLISRLNRIEGQVRGLKGLIEKDTYCDDVLNQISSVQSALNGVGKLLLEHHMKSCVMERIQEGDNQVIDELLVTINKLMK- 111

WP_081735351_1 -----------------------MAHHE---------------------------------L---------DE-Q-VG--TI--EQ----AS-------CH-T-G-S-E---------------------RKSHHSDKTKSNMVSRLNRIEGQIRGVKGMIEKDTYCDDVLNQIAAIQSALNSVGKLLLENHLNSCVIERIQEGDNDVIRELMVTMNKLIK- 114

WP_040711684_1 --------------------------------------------------------------------------------MT--CH----DE-------CA-S-QGG-E---------------------RKSHHSDKTKANLIARLNRVEGQIRGIKGLIEKDTYCDDVLNQISAAQSALNSVGKLLLEHHLKSCVTERLQAGDEQVIAELMITMNKLIR- 104

WP_045673594_1 ---------M---------------------------------------------------A---------NQ-T--E--EK--DV----GC-------CS-QPEDA-E---------------------RKSHHSHKVKSSLISRLNRIEGQVRGIKGLIEKDTYCDDVLNQIASVQAALNGVGKLLLEHHMKSCIIERIQEGDSEVLTELMTTINKLTK- 111

WP_036681383_1 -------------------------------------------------------------M---------DN-L-----TH--EH----ES-------CS-T---D-E---------------------RKSHHSDKTKKNLISRLNRIEGQVRGLKGLIEKDTYCDDVLHQISSVQSALNGVGKLLLEHHMKSCVVERIQDGENEVLDELLVTINKLIR- 106

WP_042131123_1 -------------------------------------------------------------M---------ED-H-----TH--EH----ES-------CS-T---G-E---------------------RKSHHSEKTKKNLVSRLNRIEGQVRGLKGLIEKDTYCDDVLHQISSVQSALNGVGKLLLEHHMKSCVVERIQDGENEVLDELLITINKLIK- 106

WP_013921027_1 ---------------------MDIETKE---------------------------------E---------QS-T-SA--AH--AD----DC-------CG-A-G-A-E---------------------RRSHHSAKVKDNLVSRLNRIEGQIRGVKGMIEKDTYCDDVLNQIAAIQSALNSVGKILLEGHMRSCVVERIEQGDTEVIDELMKTMGKLIK- 116

WP_044480825_1 -------------------------------------------------------------MVNEQELSKLSG-H-----GA--AG----HQ--D----QA-------A--KPEACCS-HEAGE-HAHA-RHSHHSDAVKSNLISRLNRVEGQIRGIKGMIEKDTYCDDVLNQIAAVQSALNSVGKLLLEGHMKSCVIERIQAGETEVVDELLLTVQKLMK- 130

WP_087071295_1 -------------------------------------------------------------------MR--EE-Y-----PQ--NT----AL--A----DH-------T------ECS-ST---------RRSHHSDEMKTNMVRRLNRVEGQIRGIKGMIEKDTYCDDVLNQIAAAQSALNSVGRLLLEGHMKSCVVERIEAGENEVIDELLITINKLLK- 111

WP_062322781_1 -------------------------------------------------------------M---------SE-V-----SQ--TD----DL--Q----AT-------S-----CHTA-GSDGK-H--V-RKSHHSQQMKGNLISRLNRVEGQIRGIKGLIEKDTYCDDVLTQIAAAQSALNSVGKLLLEGHMKSCIVERIQAGEHEVVDELLVTVRKLMK- 116

WP_074096703_1 -------------------------------------------------------------M---------SD-S-----TE--AS----ATVQE----AV-------S-----CHAE-GSDGK-H--V-RKSHHSQEMKSNLVSRLNRVEGQIRGIKGLIEKDTYCDDVLTQIAAAQSALNSVGKLLLEGHMKSCIVERIQAGEHEVVDELLVTMRKLMK- 118

WP_076172948_1 -------------------------------------------------------------M-TG---------D-----AL--LS----AV--G----PH-------S------SCE-GGCSE------RHSHHSEQTKNNLITRLNRIEGQIRGVKGLIEKDTYCDDVLNQIAAIQSALNGVGKLLLEGHMKSCVVERIEAGDHEVIDELLVTVKKLMK- 113

WP_036579776_1 -----------------------------------------MA------------------L-----ET--EH-I-----PA--AG-L--SE-------TD-------E------CCA-PGEGT----D-RKSHHSDKFKAGIGSRLNRIEGQIRGIRGMIERDTYCDDVLNQIAAVQSALNGVGKLLLEGHMKSCVVDRIEAGDHDVLDELLTTINKLVK- 118

WP_052020493_1 -------------------------------------------------------------M-SAA-NP--DK-D-----AF--NE----TT--DLLPEQD-------E------TCG-PGCSE------RHSHHSAQTKNNLITRLNRIEGQIRGVKGLIEKDTYCDDVLNQIAAVQSALNSVGKLLLEGHMKSCVVERIESGDHAVIDELLVTVKKLMK- 122

WP_068613751_1 -------------------------------------------------------------------MP--EQ-E-----NP------------V----VE-------D------HCC-AAGNH----T-RQSHHSEKTKSNLITRLNRVEGQIRGVKGMIEKDVYCDDILNQIAAIQSALNSVGRLLLEGHLKSCVIERIQAGETEVMDELLVTVNKLMK- 111

WP_027084721_1 -------------------------------------------------------------M---------NE-R-----IS--DN----DC-HA----KE-------S-----AHCA-SDTG-----A-RKSHHSDQAKASLISRLNRIEGQIRGIKGLIEKDTYCDDVLNQIAAAQSALGSVGKLLLEGHMKSCIVERIQAGEHEVIDELLVTVNKLMK- 115

WP_087431970_1 -------------------------------------------------------------M---------DE-N-----NQ--EL----DC-------CAVS---N-D---------------------RKSHHSEKTKKNLITRLNRIEGQIRGVKGLIEKDTYCDDVLNQISSIQAALNGVGKLLLEHHMKSCVIERIQEGDHEVIDEILTTMNKLMK- 107

WP_037285218_1 -------------------------------------------------------------------MT--IQ-------KK--VE----PV--S----EG-------E------HCH-TPGEDGK--LPRKSHHSEATKSNLVSRLNRVEGQIRGIKGMIEKDTYCDDVLNQIAAAQSALNSVGRLLLEGHMKSCVVERIEAGETEVIDELLITVNKLLK- 117

WP_042229263_1 -----------------------------------------MVLHSEKDNGRTEGETGSAPL---------AS-A-----DA--GT----SC--H----TH-------T--------R-DED-T----E-RRSHHSDKVKNNLVSRLNRIEGQIRGVKGMIEKDTYCDDVLNQIAAVQSALNSVGKLLLEGHMKSCVIERIQEGDTDVIDELLVTVNKLMK- 131

WP_082865773_1 -------------------------------------------------------------M-VDHNLD--NS-A-----KV--MD----QS--D-SDGVT-------T------HCH-SDTSI------RKSHHSAKTKSQLITRLNRIEGQIRGVKGMIEKDTYCDDVLNQIAAIQSAMNGVGKLLLEGHMKSCVVERIEAGEHEVIDELLITVNKLMK- 122

WP_076169706_1 -----------------MR------------------------------------------V---------EE-TMEE--SV--KH----ES-------CG-T---G-E---------------------RKSHHSPKTKNNLISRLNRIEGQIRGIKGLIEKDTYCDDVLNQISSVQSALNGVSKLLLEHHMKSCLIERIQDGENEVIDELLITINKLMK- 111

WP_041854490_1 ----------------------------------------------------------------------------MY--DA--EA----HV-------HS-H-D-E-H---------------------RRSHHSEKLKTDLITRLNRIEGQIRGIKGMIEKDTYCDHVLNQISAVQAALNSVGKLLLAGHMRSCVVERIQEGDLEVIDELLTTVNKLLK- 105

WP_054819715_1 -------------------------------------------------------------M---------DH-L-NG--AT--ED----IS-------CC-S-N-TEE---------------------RRSHHSDKTKSNLISRLNRIEGQIRGVKGMIERDTYCDDVLNQIAAIQSALNGVGKLLLENHMKSCVIERIQSGDHEVIDEILVTMNKLMK- 110

WP_068604123_1 -------------------------------------------------------------M-----EP--NDMK-----DH--CG-T----------------------------------------E-RKSHHSDKTKANLVSRLNRIEGQIRGVKGMIEKDTYCDDVLNQLASIQSALNGVGKLLLENHMKTCVTERLHAGEDEVIDELLTTIQKLMK- 104

WP_040951458_1 -------------------------------------------------------------------MA--DV-E-----KP--AE----EH--GTHGELE-------D------ACC-TAGSE------RRSHHSDKMKSNLISRLNRIEGQIRGVKGMLERDTYCDDVLNQIAAIQSALNGVGKLLLEGHMKSCVVERIQAGEHEVIDELLVTVNKLLK- 118

WP_083484831_1 -------------------------------------------------------------------MA--ET-R-----EN--GH----HC--E----DD-------A------CSA-ESLAS-T--E-RRSHHSDEMKNNLTRRLNRIEGQIRGIKGMIDKDTYCDDVLNQIAAVQSALNSVGRLLLEGHMKSCVIERIQAGEPEVVDELLITVNKLMK- 116

WP_082063348_1 -------------------------------------------------------------M-GN--EG--NT-S-----EQ--PK----TP--EI---EA-------D------HCH-TDATE------RHSHHSDQTKSNLITRLNRIEGQIRGIKGMIEKDTYCDDVLNQIAASQAALNGVGKLLLEGHMKSCVVERIEAGDHDVIDELLKTVNKLMK- 118

WP_091186211_1 -----------------------------------------------------------------------------M--KL--EE----TC-------CH-T-D-S-N---------------------RKSHHSEKTKSNLTSRLNRIEGQIRGIKGLIEKDTYCDDVLNQIAAVQSALNSVGKILLEHHMNSCVIERIQEGDNEVIKELMVTMNKLIK- 104

WP_108464135_1 -------------------------------------------------------------M---------KE-E-----NQ--AG----EK--Q----AS-------Q------CCD-SHHA-----D-RQSHHSEAFKGKLITRLNRIEGQIRGVKGLIEKDTYCDDVLNQIAAIQSALNGVGKLLLEGHMKSCLIERIQAGEMEVVDELLVTVNKLMK- 113

WP_082655297_1 -------------------------------------------------------------------MA--NQ-E-----EL--LP----D-------EAD-------R------SCC-SPESE------RKSHHSDKMKNNLISRLNRIEGQIRGVKGMIEKDTYCDDVLNQIAAIQSALNSVGKLLLEGHMKSCVVERIQAGEHEVIDELLITVNKLMK- 113

WP_016313703_1 -------------------------------------------------------------------MAKVEG-Q-----EHVASA----HC--E----EH-------G--GHQHVSE-TENGK-P--V-RRSHHSEAMKSNLTSRLNRIEGQIRGIKGLIERDTYCDDVLNQIAAVQSALNSVGRLLLEGHMKSCVIERIQAGETEVLDELLVTVNKLMK- 124

WP_036626513_1 -------------------------------------------------------------------MAD-NV-Q-----EH--TH----HC--E----DH-------GVQNEGKAEG-KTAGK-T--V-RRSHHSEALKSNLTSRLNRIEGQIRGIKGLIERDTYCDDVLNQIAAVQSALNSVGRLLLEGHMRSCVIERIQAGETEVVDELLVTVNKLMK- 123

WP_015846873_1 -------------------------------------------------------------M---------IE-Q-----EK--TE----HC--H----SN-------E--------S-EQQGA----E-RKSHHSDKMKSNLVSRLNRIEGQIRGVKAMIEKDTYCDDVLNQIAAVQSALNGVGKLLLEGHLKSCVIERIQAGESEVIDELLVTVNKLMK- 112

WP_091184108_1 -------------------------------------------------------------M---------IE-Q-----EK--SE----HC--H----SD-------Q--------P-DQQGA----E-RKSHHSDKMKSNLVSRLNRIEGQIRGVKAMIEKDTYCDDVLNQIAAVQSALNGVGKLLLEGHLKSCVIERIQAGESEVIDELLVTVNKLMK- 112

WP_007428460_1 -------------------------------------------------------------M---------AE-E-----QP--VS----HH--E----VH-------E-----NHCG-TDGEK-T--V-RKSHHSAEFKSSLTSRLNRIEGQVRGIKGLIEKDTYCDDVLNQIAAVQSALNGVGKLLLEGHMKSCVIERMQAGEPEVIDELLVTVKKLIR- 116

WP_013308456_1 -------------------------------------------------------------M---------AE-E-----QP--VS----HT--E----VH-------E-----NHCG-TDGEK-T--V-RKSHHSAEFKNSLVSRLNRIEGQVRGIKGLIEKDTYCDDVLNQIAAVQSALNGVGKLLLEGHMKSCVIERMQAGEQEVIDELLVTVKKLIR- 116

WP_013369073_1 -------------------------------------------------------------M---------AE-E-----QP--VS----HT--E----VH-------G-----AHCG-TDGEK-T--V-RKSHHSAEFKNSLVSRLNRIEGQVRGIKGLIEKDTYCDDVLNQIAAVQSALNGVGKLLLEGHMKSCVIERMQAGEQEVIDELLVTVKKLIR- 116

WP_014279443_1 -------------------------------------------------------------M---------AK-E-----QP--VS----HT--E----VH-------E-----A-CGRTDGDK-I--V-RKSHHSAEFKNSLTSRLNRIEGQIRGIKGLIEKDTYCDDVLNQIAAVQSALNGVGKLLLEGHMKSCVIERMQAGEPEVIDELLVTVKKLIR- 116

WP_016818842_1 -------------------------------------------------------------M---------AE-E-----QP--VS----HT--E----VH-------G-----THCG-TDGEK-T--V-RKSHHSAEFKNSLVSRLNRIEGQVRGIKGLIEKDTYCDDVLNQIAAVQSALNGVGKLLLEGHMKSCVIERMQAGEQEVIDELLVTVKKLIR- 116

WP_091186252_1 ------------------------------------------------------------------------------------------------MNEIN-------D------VCC-SSDNE------RKSHHSDKMKGSLISRLNRIEGQIRGVKAMIERDTYCDDVLNQIAAIQSALNGVGKLLLEGHMKSCVVERIQAGENEVIDELLITVNKLMK- 105

WP_077996729_1 -------------------------------------------------------------M-----EH--AN-------ES--PK-L----------------------------------------E-RKSHHSDKTKNSLVTRLNRIEGQIRGVKGMIEKDTYCDDVLNQLAAIQSALNGVGKLLLEHHMKSCVINRIQNGEPEVIDELLITVNKLIK- 102

WP_025332844_1 -------------------------------------------------------------M-----TA--HE-K-----RE--ET-LDQGS-------CD-------G------DCH---SSE----V-RKSHHTQEFKNGLTARLNRIEGQIRGIKGMIDRDTYCDDVLTQLAAVQSALGSVGKLLLEGHMKSCIVERIEAGEHEVIDELLITVGRLMK- 116

WP_038693187_1 -------------------------------------------------------------M-----AT--HH-K-----RE--DD-SETSV-------CA-------G------DCH---PSG----E-RKSHHAPEFKSGLTARLNRIEGQIRGIKGMIERDTYCDDVLTQLAAVQSALGSVGKLLLEGHMKSCIVERIEAGEHEVIDELLITVGRLMKS 117

WP_025694977_1 -------------------------------------------------------------M-----AA--HE-K-----HD--EGVVPHDC-------CG-------G------DCH---SSE----E-RKSHHTQEFKNGLTTRLNRIEGQIRGIKGMIERDTYCDDVLTQLAAVQSALGSVGKLLLEGHMKSCIIDRIEAGEHEVVDELLVTIGRLMK- 117

WP_042204830_1 -------------------------------------------------------------M-----AA--HE-K-----HD--EGVVPHDC-------CG-------S------DCH---SSE----E-RKSHHTQEFKNGLNTRLNRIEGQIRGIKGMIERDTYCDDVLTQLAAVQSALGSVGKLLLEGHMKSCIIDRIEAGEHEVVDELLVTIGRLMK- 117

WP_083180585_1 ------------------------------------------------------------------------------------------------------------------------------------------MKGKLVARLNRIEGQIRGVKGMIEKDTYCDDVLNQIAAIQSALNGVGKLLLEGHMKSCIVERIQDGETEVLDELIVTINKLMK- 83

WP_042184298_1 -------------------------------------------------------------M-----ST--SK-Q-----EQ--TE----KS-------CE-------A------DCH-SSASG----E-RMSHHSTDFKNGLNTRLNRIEGQIRGIKGMIERDTYCDDVLNQLAAVQAALNGVGKMLLEGHMKSCIIERIEAGEHEVIDELLITVNKLMK- 115

WP_042123574_1 -------------------------------------------------------------M-----ST--SK-Q-----EQ--TE----KS-------CE-------A------GCH-S--SG----E-RMSHHSADFKNGLNTRLNRIEGQIRGIKGMIERDTYCDDVLNQLAAVQAALNGVGKMLLEGHMKSCIIERIEAGEHEVIDELLITVNKLMK- 113

WP_038585124_1 -------------------------------------------------------------M-----AP--KE-E-----KL--SP----ES-------CD-------S------SCH---TPG----E-RKSHHSPEFKNGLTTRLNRIEGQIRGIKGMIERDTYCDDVLNQLAAVQAALNGVGKLLLEGHMKSCIIERIEAGEHEVIDELLITVNKLMK- 113

WP_039869277_1 -------------------------------------------------------------M-----AP--KE-E-----KQ--SS----AA-------CD-------S------SCH---APG----E-RKSHHSEEFKNGLATRLNRIEGQIRGIKGMIERDTYCDDVLNQLAAVQAALGGVGKLLLEGHMRSCIVERIEAGEHEVIDELLITVNKLMK- 113

WP_042172316_1 -------------------------------------------------------------M-----AP--KE-E-----KQ--TP----AA-------CD-------S------SCH---APG----V-RKSHHSEEFKNGLATRLNRIEGQIRGIKGMIERDTYCDDVLNQLAAVQAALGGVGKLLLEGHMRSCIVERIEAGEHEVIDELLITVNKLMK- 113

WP_051491259_1 -------------------------------------------------------------M-----ST--AK-K-----EQ--AA----ES-------CD-------I------NCH-SSSSG----E-RKSHHSAEFKNNLNTRLNRIEGQIRGVKGMIERDTYCDDVLNQLAAVQAALNGVGKLLLEGHMKSCIVERIEAGEHEVIDELLITVNKLMK- 115

WP_087919272_1 -------------------------------------------------------------M-----PA--KK-K-----EP--AA----HT-------CG-------E------DCQ-SADPG----E-RKSHHSTEFKNGLTTRLNRIEGQIRGVKGMIERDTYCDDVLNQLAAVQAALNSVGKLLLEGHMKSCIVERIEAGEHEVIDELLITVNKLMR- 115

WP_036650864_1 -------------------------------------------------------------M-----AT--HE-E-----EH--AG----QP-------NG-------D------DCH---TSK----V-RKSHHSDEFKNGLGNRLNRIEGQIRGIKGMIERDTYCDDVLNQLAAVQSALNSVGKVLLEGHMKSCIIERIEAGEHEVIDELLVTVNKLMK- 113

WP_042133035_1 -----------------------------------------MGGILYCYLRRLIP------V-----ST--NE-K-----EH--AG----HD-------CG-------E------DCH-SSASG----V-RKSHHSPEFKSGLTTRLNRIEGQIRGVKGMIERDTYCDDVLNQLAAVQAALNSVGKLLLEGHMKSCIIERIEAGEHEVIDELLITVNKLMK- 129

WP_039786745_1 ------------------------------------------------MLRRLIP------V-----ST--NE-K-----EH--AG----HS-------CG-------D------DCH-TSAPG----V-RKSHHSAEFKSGLTTRLNRIEGQIRGVKGMIERDTYCDDVLNQLAAVQAALNSVGKLLLEGHMKSCIIERIEAGEHEVIDELLVTVNKLMK- 122

WP_042241378_1 ----------------------------------------------------MIP------V-----ST--NE-K-----EH--AG----HD-------CG-------E------DCH-SSASG----V-RKSHHSPEFKSGLTTRLNRIEGQIRGVKGMIERDTYCDDVLNQLAAVQAALNSVGKLLLEGHMKSCIIERIEAGEHEVIDELLITVNKLMK- 118

WP_099052435_1 -------------------------------------------------------------M-----ST--NE-K-----EH--AG----HD-------CG-------E------DCH-SSASG----V-RKSHHSPEFKSGLTNRLNRIEGQIRGVKGMIERDTYCDDVLNQLAAVQAALNSVGKLLLEGHMKSCIIERIEAGEHEVIDELLITVNKLMK- 115

**paen9.00802, YycC-like protein**

WP_046233313_1 MSQPLQISADTAIKLSKQLGIPIEHLMHMPKHILLQKLGELA--KA---EA---E-A--NT-----------AAS---SP-SE-DE----A-SGDKPL--------------------------------- 66

WP_099519089_1 MSQPLQISADTAIKLSKQLGIPIEHLMHMPKHILLQKLGELA--KA---EA---E-A--NT-----------AAA---TP-SK-DE----A-SGDKPV--------------------------------- 66

WP_087917986_1 M-KPLQISAETAVALSKQLGVPLEHLMHMPQHILLQKIAELS--RK---PD---T-A--PD------VNA--NDSS--PA-SD-KE------PQ------------------------------------- 64

WP_036648016_1 M-RPLQISPDTAIKLSEKLGVPLEQLMHMPQHILLQKIAELS--KE---EP---A-L--DQ---KN------ADT---DA-KD-PS--------------------------------------------- 60

WP_014368682_1 M-RPLQISPDTAQKLAKELGVPLEHLMHMPQHILLQKLGELA--RK---EA---S----------P------AGE---AA-KE-PA-AGDGAKE------------------------------------- 63

WP_108466829_1 M-RPLQISAETAVKLADKLGVPLEQLMHMPQHILLQKLGELA--KE---DA---R-Q--EP-----------PKE---S---------------------------------------------------- 53

WP_015253552_1 MPGPRPISPDTAIKLSKYLGVPLEHLMHMPRHILLQKLAEMA--AK---EQ---A-Q--NQ-----------END---RE-QA-SD----R-TE-DPKSGPPAGRNSEASDKPGGDESR------------ 86

WP_037291248_1 M-RPLQISPDTAIKLSKQLNVPIEHLMHMPQNILMQRMAELA--KK---EA---A-E--AA-----------NAK---PE-DD-TE----G-NK------------------------------------- 61

WP_006037383_1 MSQPLQISAETAIKLSEQLKVPIEHLMHMPRHILLQKMAELA--------------A--TS-----------SAK---PT-NE-AE----P-QS------------------------------------- 57

WP_036586416_1 MTGPLQISPDTAIKLSKQLNVPIEHLMHMPRHILLQKLGELA--AA---EA---K-NAHGE-----------PSA---SD-KP-PE----D-PR---T--------------------------------- 65

WP_042164412_1 MSKPLQISPDTAIKLSKQLGVPIEHLMHMPRHILLQKLAELA--KA---EA--------GN-----------AAE---QT-DK-KE----D-SS---L--------------------------------- 61

WP_015842834_1 MSQPLQISPETAIKLSKQLGVPLEHLMHMPKHILLQKLTELA--KA---EA---E-A--GK-----------PSE---SAPGT-EE----S-SS------------------------------------- 63

WP_091186514_1 MSQPLQISPETAIKLSKQLGVPLEHLMHMPKHILLQKLTELA--KA---EA---E-A--GT-----------PSK---SA-DS-EE----S-SS------------------------------------- 62

WP_062406427_1 M-RPLPISPETAQRLARELNVPIEHLMHMPQHILMQKLAELA--KK---KP---D-E--SA------AKN--DAP---ES-GR-PT------SE------------------------------------- 63

WP_038596193_1 M-KPLQISPETAITLSKQLGVPLEHLMHMPQHILLQKIAELS--KQ---QS---A-E--TD------GKA--AET---PP-AE-KE------EQ------------------------------------- 63

WP_069202074_1 M-RPMPISADTALKLSKFMNVPVEHLMHMPQHVLLQKLAEMA--MA---EG---G-K--DK------SDN--GGK---TE--------------------------------------------------- 57

WP_016310919_1 M-KPLQISADTAVKLAAKLKVPVEHLMHMPQHILMQKIAELA--KE---EA---S-Q--PS------APSEPGID---SQ-AGHESERS------------------------------------------ 67

WP_077995948_1 M-KPLQISAETAQKLSKQLGIPLEHLMHIPQHILLQKLSELA--KS-I-PE---N-E--NG-----------SKD---PN-HN-LD----S-KE---------GKNS------------------------ 66

WP_042180479_1 M-KPLQISPETAITLSKQLGVPLEHLMHMPQHILLQKIAELS--KK---QS---T-D------------A--GTE---AP-PE-KE------NQ------------------------------------- 59

WP_036647552_1 M-RPLQISPETAIALSKQLGVPLEHLMHMPQHILLQKIAELS--RKEATPT---T-G--ED------LSS--GTETDEPS-AQ-KD------ET------------------------------------- 69

WP_089524378_1 MSRPLQISAETAVMLAKELKVPLEHLMHMPRHILLQKMAELARVQS---ETMEPE-S--TD-----------STD---SD-SK-DK----P-DE------QL----------------------------- 69

WP_042217338_1 M-KPLQISPETAITLSKQLGVPLEHLMHMPQHILLQKIAELS--KK---AS---T-E--PA------GGS--ASE---PS-PE-QD------KQ------------------------------------- 63

WP_020430169_1 M-KPLQISPETAVTLAKQLGVPLEHLMHMPQHILLQKIAELS--KK---QS---Q-D--TT------DSS--SAA---EA-PE-KG------EQ------------------------------------- 63

WP_039876914_1 M-KPLQISPETAVTLSKQLGVPLEHLMHMPQHILLQKVAELA--KK---QT---T-D------------A--GTE---AP-PE-KE------QE------------------------------------- 59

WP_082063186_1 M-KPLQISAETAITLSKKLGVPIEHLMHMPQHILLQKITELS--KQ-E-TS---K-----------------------PD-DE-KD------PS------------------------------------- 55

WP_099478440_1 M-RPLQISPETAVTLSKQLGVPLEQLMHMPQHILMQKIAELA--KN---DK---AEP--QS------EQE--SEQ------GR-SE------NS------------------------------------- 62

WP_087435891_1 M-RPLQISADTALKLSKALGVPVEQLMHMPQHILLKQLAELA--QA---DA---D-S--SA-AK--------NHT---SA-EN-SK--------------------------------------------- 60

WP_038573204_1 M-KPLQISPETAITLSKQLGVPLEHLMHMPQHILLQKIAELS--KK---QN---P-P--QT------EGE--NEQ---TP-SG-KD------SQ------------------------------------- 63

WP_042130920_1 M-KPLQISPETAITLSKQLGVPLEHLMHMPQHILLQKIAELS--KK---QN---S-P--QA------EDG--NEE---SP-SG-KD------SQ------------------------------------- 63

WP_026021234_1 M-KPLQISAETAIKLSEKLNVPIENLMHMPQHILMQKLAELA--QQ---EK---E-A--GH------PSS--SME-Q-PK-DS-GE------NR---------G--------------------------- 65

WP_062495204_1 MSKPLPISAETAVKLAEKLNVPLEHLMHMPRHILLQKLAELA------------N-E--PD-----------RAK---PE-DR-GG----G-SS------PL----------------------------- 60

WP_025336332_1 M-RPLQISPETALMLSKQLGVPLEHLMHMPQHILLQKIAELS--KK---NT---E-E--GN------AEA--GGE-------G-KD------KA------------------------------------- 60

WP_025695894_1 M-RPLQISPETALALSKQLGVPLEHLMHMPQHILLQKIAELS--KK---NT---D-G--GE------PKD--GVE-------G-KD------EA------------------------------------- 60

WP_036594730_1 M-RPLQISPETALALSKQLGVPLEHLMHMPQHILLQKIAELS--KK---NT---D-G--GE------PKD--GAE-------G-KD------EA------------------------------------- 60

WP_074096390_1 M-KPLQVSADTAVKLAESLGVPLEHLMHMPQHILMQKIAELA--KQ---ET---S-K--PS------APE--GEQ---E---------------------------------------------------- 56

WP_082083590_1 MSKPLQISPETALMLSKRLNVPLEHLMHMPKHILLQKMAELA--RS---E----N-A--GS-----------DQS---TQ-GA-GL----P-QE------PAAGNGSSATGDSDSQTAETDDDTPEGNRGT 92

WP_082704167_1 M-KPLQVSADTAVKLAESLGVPLEHLMHMPQHILMQKIAELA--KE---EA---A-K--AA------PAK--GEQ---E---------------------------------------------------- 56

WP_084402780_1 M-RPLQISAETAITLSKKLGVPIEHLMHMPQHILLQKIAELS--KE-DTSS---E-----------------------PT-IE-KD------PS------------------------------------- 56

WP_015737435_1 M-RPLQISPETAVTLSKQLGVPLEQLMHMPQHILMQKIAELA--KN---EE---A-K--AT------PKD--DES------HS-SE------NS------------------------------------- 61

WP_036619824_1 M-KPLQISPDTAVKLAAKLKVPLEHLMHMPQHILLQKIAELA--KE---EAG--S-K--PAEAGAEPSASEPAGD---SK-PGDPKQSL------------------------------------------ 74

WP_025707485_1 M-KPLQISPETAVTLAKQLGVPLEHLMHMPQHILLQKIAELS--KK---QS---E-G--TT------GSE--PES---QA-PE-KG------EQ------------------------------------- 63

WP_005551157_1 M-RPLPISADTAVKLSKALHVPLEQLMHMPQHILLQKLAELN--KA---QQ---N-P--SD-T---------DKS---PE-NT-TE--------------------------------------------- 59

WP_074048569_1 M-KPLQVSPETAVKLAEKLKVPLEHLMHMPQHILMQKIAELA--KE---EA---N-K--PA------SPDDSGAS---S---------------------------------------------------- 58

WP_081757132_1 M-KPLQISPDTAIKLAKALHVPIEQLMHMPPHILMQKLAELS--KQ---EA---E-N--GQ-----------KTE---AD-QE------------------------------------------------ 56

WP_014277894_1 M-RPLQISPETAVKLSEKLGIPLEHLMHTPQHILMQKLAELA--KE---ET---A-D--PN-----------GGE---SD-KS------------------------------------------------ 56

WP_006212637_1 M-RPLQISPETAVTLSKQLGVPLEQLMHMPQHILMQKIAELA--KN---DE---G-P--SS------KKD--DEP------HS-SE------NS------------------------------------- 61

WP_007431992_1 M-RPLQISPETAVKLSEKLGIPLEHLMHTPQHILMQKLAELA--KE---ET---T-D--PN-----------GGE---SD-KS------------------------------------------------ 56

WP_013372937_1 M-RPLQISPETAVKLSEKLGIPLEHLMHTPQHILMQKLAELA--KE---DT---A-G--SN-----------GGE---SD-KS------------------------------------------------ 56

WP_016819983_1 M-RPLQISPETAVKLSEKLGIPLEHLMHTPQHILMQKLAELA--KE---EA---A-G--SN-----------GGE---SD-KS------------------------------------------------ 56

WP_080683342_1 M-RPLQISPETAVKLSEKLGIPLEHLMHTPQHILMQKLAELA--KE---ET---A-G--SN-----------GGE---SD-KS------------------------------------------------ 56

WP_071193212_1 M-RPLQISPETAITLSKKLGVPIEHLMHMPQHILLQKIAELS--KE-D-AP---K-----------------------ST-DE-KD------PS------------------------------------- 55

WP_084706668_1 M-KPLNISPDTAVKLAKHLGVPLEHLMHMPQHILLQKLAELA--SS---KP---D-K--DP-----------APD---SE-SE---------TK------------------------------------- 58

WP_076248154_1 M-KPLQISPETAITLSKQLGVPLEHLMHMPQHILLQKIAELS--KK---AS---T-E---------------------------QD------KE------------------------------------- 51

WP_081956685_1 M-KPLQISPETAITLSKQLGVPLEHLMHMPQHILLQKIAELS--KK---AS---S-E---------------------------QD------KQ------------------------------------- 51

WP_071784237_1 M-KPLQISPETAIKLAAELKVPIEHLMHMPQHILLQKLADLA--KK---E-------------------S--ATP---SP-EK-PD------SSS------------------------------------ 57

WP_068620139_1 M-RPLQISAETAVKLAEKLGVPLENLMHMPQHILLQKISELA--KE---EA---G-S--ED-----------KPK---S---------------------------------------------------- 53

WP_038698560_1 M-RPLQISPETALALSKQLGVPLEHLMHMPQHILMQKIAELS--KK---NA---S-E--GG------SPE--G-E-------G-KN------EA------------------------------------- 59

WP_064505603_1 M-RPLQISADTAVKLAEKLNVPLEQLMHMPQHILMQKLAELA--KD---EQ---K-D--GS-----------E---------------------------------------------------------- 50

WP_076175142_1 M-RPLQISPETAVKLSEKLGVPLEHLMHMPQHILLQKLAELA--KE---EA---G-K--QD-----------GDE---GT-KS------------------------------------------------ 56

WP_068603279_1 M-KPLNISADTAVKLAEHLKVPIEQLMHMPQHILLQKLAELA--SA----T---E-K--DT-----------GKS-------------------------------------------------------- 51

WP_068697579_1 M-RPLQISADTAVKLAEKLHVPIEQLMHMPQHILMQKLAELA--KE---DA---A-K--DK-----------PDE---Q---------------------------------------------------- 53

**paen9.00805, Uncharacterized spore protein YtfJ**

WP_014281971_1 -MSEHPLQSFVQTAKDHLKDMMDVNTTIGEPVTVSDGT-IIIPICKVSYGFAAGGSDWER--H--I-QH---------------SA----------RV-----TPSFGGGMGGGVAVTPLAILIIGKMG--VQTIYLDNSPTVYDKILDMAPHLLDKLNNLI-KQYQ------T-T-DDN-SQ----------------- 134

WP_094155205_1 -MSEHPLQSFVKTAKDHLKDMVNVNTAIGEPVALSDGT-MIIPVCKVSYGFAAGGSDLDR--H--N-QE---------------SA----------WV-----MPSFGGGMGGGVSVTPLAILIIGKMG--VQTIYLDNSPTVYDKILDMAPHLLDKLHNLM-KQYQ------T-G-DDS-SQ----------------- 134

WP_038596748_1 -MSEH-IHQLVETALQNLKGMNDAETVIGKPIQTPDGT-VVIPICRTNFGLVTGGTEF---------SS---------------AV----------AA-----KLPFGGGIGVGMSIIPVAFLIIGPSG--VQMVPLESPKDAYSRLIDLSPQLLDKLKSLL-ER----------------------------------- 120

WP_025698007_1 -MSEQ-IHQLLENVLPNLKGIIDAESVIGEPIQTPDGT-VVIPICRTNFGLVAGGTEF---------RG---------------SG----------SS-----VLPFGGGIGAGVSVTPVAFLIIGPSG--VQTVSLDSPKDIYSRILDLSPQLLDKLKSLL-DRQP--------------------------------- 122

WP_042205911_1 -MSEH-IHRLLETALPNLKGVIDAESVIGEPIQIPDGT-VVIPICRTHFGFVTGGTEF---------SS---------------SA----------SS-----SLPFGGGIGAGASVTPVAFLIIGPSG--VQTVLLDSPKDIYSRILDLSPQLLDKLKSLL-DR----------------------------------- 120

WP_025335629_1 -MSDHPIQGLMQTAMENIKGMVDVNTIVGEPVETKDGS-VILPISKVAFGFAAGGSDFSV--E----DD------DKPVA----GQ-----N----GV-K---ILPFGGGSGGGVSIRPIAFLVVGREG--VHIVPLDNQTHIFEKIIDSTPGLIDKIQSMF-QNQG-----------GS-SS--G-----QQQQ----- 142

WP_081793606_1 -MMEHPIQGLMETAMESIKEMVDVNTIVGDPVQTPDGS-VIMPISKVGFGFVAGGSDIRF--D----GS-GG---TSATI----EE-----HN---AS-V---SLPFGGGSGGGVSITPIAFLVVGSHG--VKIVSLDNSTHLWERMIDSAPKVVDKLQSML---------------KGG-AN--A-------------- 138

WP_046231990_1 -MQEHPIQGLMQTAMENIKEMVDVNTIVGDPVQTPDGS-IIMPISKVGFGFVAGGSDMRF--S----GD-SSS--TSNSAD---DS-----HN---AA-V---AAPFGGGSGGGVSITPIAFLVVGTQG--VKIVPLDNNTHLLERIIESTPYVFDKVQSMM---------------RST-SS--G-T---GQFT----G 146

WP_099519904_1 -MQEHPIQGLMQTAMENIKEMVDVNTIVGDPVQTPDGS-IIMPISKVGFGFVAGGSDMRF--S----GD-SSD--SSSSSD---DS-----HK---AT-V---ATPFGGGSGGGVSITPIAFLVVGTQG--VKIVPLDNNTHLLERIIDSTPYVFDKVQSMI---------------RSS-SS--G-A---SQLT----G 146

WP_005546833_1 -MAEHPIEGLMQAVFNNIKAMVDTNAIVGDPVQTPDGS-VILPISRVALGFAAGGSDIRV--E----ER---H--DRLTT----DH-HEP-HA-T-SV-Y-G-SKPFGGGSGGGVYITPIAFLVVGKQG--IHVVSLDNQTHLLEKIIDAVPNVCDRINSMF-KK----------Q-ERS-TT--A-----EDII--L-- 150

WP_062492715_1 -MAEHPIQGLMQTAMENIKEMVDVNTIVGDPVQTPDGS-VIMPISKVGFGFVAGGSDIRF--DDRNDGT-ASA--GVSNGT---GH-----HN---AS-V---ALPFGGGSGGGVSITPIAFLVVGANGTGVRVVPLDNQTHLMERVIDAAPQVFEKIQRMI---------------RNR-ET--E-------------- 146

WP_082084126_1 -MAEHPIQGLMQTAMDNIKQMVDVNTIVGDPVQTPDGS-VIMPISKVGFGFVAGGSDIQM--N----GS-QAK--AGS------DA-----AH---AA-V---ALPFGGGSGGGVSITPIAFLVVGVQG--VKVVPLDNQTHLMERVIDAAPQVVDKIQTMF---------------RSN-AT--G-------------- 137

WP_087433119_1 -MAEHPIEGLMHAAFENIKAMVDVNAIVGDPVQTPDGS-VILPISRVALGFAAGGSDFRI--E----EK-ELQ--RRESG----AQ-HHP-PA----------AKPFGGGSGGGVCITPIAFLVVGPQG--INVVSLDNQTHLLEKIIDVTPKVLDRIEAMI-RRSP------S-Q-SKP-VP--G-----EDII--L-- 150

WP_042162615_1 MAAEHPIQGLMQTAMENIKEMVDVNTIVGDPVQTPDGS-IIMPISKVGFGFVAGGSDIRM--D----GS------EGASQS---DG-----HN-HNAS-V---SLPFGGGSGGGVSITPIAFLVVGTQG--VRVVPLDNQTHLLERIIDSAPQVFDKVQSMM---------------KSP-SR--S-------------- 140

WP_015254778_1 -MLEHPIQGLMETAMESIREMVDVNTIVGDPVQTPDGS-VILPISKVGFGFVAGGSDIKL--D----GN-SEP--THASV----DQ-----HN---AT-V---SLPFGGGSGAGVSITPIAFLVVGSQG--VKIVSLDNQTHLWERVIDSAPKMLDKLSAMM---------------KSG-SH--K-------------- 139

WP_023482608_1 -MSDHPIQGLMKTAMENIKEMVDVNTIVGDPVETPDGS-VIMPISKVGFGFAAGGSQFVT-DN----AA---R--EVRKT----DG-SQT-HT---SE-V---AMPFGGGSGGGISITPIAFLVVGKPG--VKVVPLDNQTHIVERLIDSAPQFVEKLQNMM-------------K-SKN-AT--G-----TTQP----- 146

WP_027085005_1 MTQQHPINGLMQTAMENIKDMVDVNTIVGEPVQTPDGS-VILPISKVGFGFAAGGSEFQG--A----DE-ANK--GNSKS----NN-----NE---GH-NASVQLPFGGGSGGGVSITPIAFLVVGTQG--VKVVPLDSQTHILERLIESAPTWVDKIKNAF-------------Q-SNV-----G-------------- 142

WP_006039315_1 -MADHPIQGLMQTAMENIKEMVDVNTIVGDPVQTPDGS-VIMPISKVGFGFVAGGSDIRF--D----GS-SQP--SDQHAAGGADA-----HN---AV-V---QGPFGGGSGGGVSITPIAFLVVGTHG--VKVVPLDNNTHLIERVIDSAPHVFDRIQGML---------------KSK-----E-------------- 141

WP_015843958_1 -MPEHPIQGLMQTAMENIKEMVDVNTIVGDPVQTPDGS-IIMPISKVGFGFVAGGSDIRF--D----GS-ASH--DHSSSS---DG-----HNQHNAG-A---AGPFGGGSGGGVSITPIAFLVVGTHG--VKIVPLDNQTHLMERIIDSAPVVFDKVQHMF---------------RTS-AG--K-------------- 143

WP_091188969_1 -MPEHPIQGLMQTAMENIKEMVDVNTIVGDPVQTPDGS-IIMPISKVGFGFVAGGSDIRY--D----GN-ASH--EHATPS---DG-----HN-HNAS-V---AGPFGGGSGGGVSITPIAFLVVGTHG--VKIVPLDNQTHLMERIIDSAPVVFDKVQHMF---------------KTS-AA--G-------------- 142

WP_044876390_1 -MEEHPIQGLMKTAMENIKNMVDVNTIVGDPVQTPDGS-VILPVSKVTFGFAAGGSDFNI--E----EK-NKS--GGT-A----NG-----NE---EK-K---RHPFGGGAGGGISISPISFLVVGKDG--VYMLPLDTQTHIYEKMIDTVPYVVDKIQSMC-------------Q-KVN-----T-------------- 137

WP_082034320_1 -MFDHPINDLMQTAMENIKEMVDVNTIVGEPVQTPDGS-VIMPISRVNFGFAAGGSDYNT--S----NE-TSI--GGTRM----EG-----AT---AS-V---ALPFGGGSGGGISITPIAFLVVGSQG--VNVVPLDNQTHILERLIDAAPHWVDKIKSMF---------------QGA-AP--A-------------- 139

WP_068657744_1 -MEDHPIKGLMQTAMENLKSMVDVNTIVGDPVQTPDGS-VILPISKVAFGFAAGGSDFNV--D----RK-AKS--SGSSS----NE-----HQ---DS-K---RLPFGGGTGGGVSIRPIAFLVVGKDG--VYILPLDSQTHIYEKLIDTVPYVMEKIQTIC-------------E-KSG-----N-------------- 138

WP_010273939_1 -MADHPIQGLMKTAMENIRNMVDVNTIVGDPVETPDGS-VIMPISKVGFGFAAGGSEFTT--E----PS---R--NTGA-----AP---M-DH---AE-V---SLPFGGGSGGGISITPIAFLVVGTQG--VKIVPLDNQTHLIERLIDSAPQVVEKIQTMV-------------R------------------------ 132

WP_068609309_1 -MTEHPIQGLMQTAMENIKDMVDVNTIVGDPVETVDGT-VILPISKVGFGFAAGGSEFAA--E----EG------HGHGL----HD-----HA---KV-----AMPFGGGSGGGISITPIAFLVVGPTG--IKVVSLDNQTHILERLIDSAPSVVEKIQGMM---------------KGN-----T-------------- 133

WP_089523778_1 -MAEHPIQGLMQTAMENIKEMVDVNTIVGDPVQTPDGS-VIMPISKVGFGFVAGGSDIRM--D----GS-AHS--NSTHA----DA-----HN---AQ-V---ASPFGGGSGGGVSITPIAFLVVGTHG--VRVVPLDNGTHLVERVIDSVPGAFEKIQGMF---------------RNS-GT--D-------------- 139

WP_013916453_1 -MTEHPIQGLMKTAMENIKEMVDVNTIVGDPVETPDGS-VIMPISKVGFGFAAGGSEFVTTEE----AN---P--PGLTR----HE---S-HN---AQ-V---ALPFGGGSGGGVSITPIAFLVVGKQG--VKIVPLDNQTHLLERLIDSAPQVVDRIQSMM-------------K-SGGGKS--Q-----GSEQ----- 146

WP_054819900_1 -MSEHPIQGLMKTSMENIKAMVDVNTIVGDPVETPDGS-VIMPISRVAFGFAAGGSDYNV--E----DG---S--SVSRN----DA-----HN---AS-V---ALPFGGGSGGGVSITPIAFLVVGKNG--VKILPLDNQTHLVERLIDSAPHVVDRLQSMI-------------K-ANS-------------------- 135

WP_074095666_1 -MSDHPIQGLMETAMENIKAMVDVNTIVGDAVETPDGT-VILPISKVGFGFAAGGSDFHV--N----GE-SSG--KSGAT----GTSAEHASS---AK-V---ASPFGGGSGGGVSIRPIAFLVVGKQG--VHIVPLDNSTHLFEKLIDSTPYVLDRIQGMF--------Q------RN--------------------- 141

WP_016311757_1 -MADHPIKGLMQTAMENIKEMVDVNTIVGEPVETPDGS-VILPISRVGFGFAAGGSDFIV--D----DE------HNKST----TGS----SE-N-SN-----VRPFGGGSGGGVSINPIAFLVVGKPG--VHIVPLDNQTHLVEKIIDTVPGIIDRIQSMF---------------PNQ-----N-------------- 135

WP_062324412_1 -MSDHPIQGLMETAMENIKAMVDVNTIVGDAVETPDGT-VILPISKVGFGFAAGGSDFRV--N----EG-GSS--KASA-----GSPTEHASS---AS-V---ASPFGGGSGGGVSIRPIAFLVVGKQG--VHIVPLDNSTHLFEKLIDSTPYVMDRIQDML--------RN-----RN--------------------- 141

WP_044479062_1 -MAEHPIQGLMKTAMENIKEMVDVNTIVGEAVQTPDGSSVILPISRVGFGFAAGGSDFHL--D----EN------QTSNN----GE----------AK-----PRPFGGGSGGGVSINPIAFLVVGQEG--VHIVPLDNQTHLFEKLIDSVPYVMDKIESMI---------------PGG-----N-------------- 132

WP_042228524_1 -MTDHPIQGLMKTAMENIKQMVDVNTIVGDPVETPDGS-VIMPISKVGFGFAAGGSEFTT--E----TL---P--EVGSR----GG---L-EK---AA-V---EMPFGGGSGGGVSITPIAFLVVGKHG--VRIVPLDNQTHLIERLIDSAPQIVDRVQSMI-------------K-QGG-------------------- 136

WP_108465979_1 -MNDHPIQGLMQTAMENIKDMVDVNTIVGEPVETAGGN-TILPISRVGFGFAAGGSDFNV--D----AE------PITGV----D-----------GK-----YRPFGGGSGGGVSINPIAFLVVGQQG--VNIVPLDNQTHIFEKLIDAVPTMVDKVQSMF---------------PGQ-----Q-------------- 130

WP_068649015_1 -MSDHPIQGLMQTAMENIKGMVDVNTIVGDPVQTPDGS-VILPISKVTFGFAAGGSDFRV--E----SS-TKN--SG--S----ND-----QQ---DK-K---TLPFGGGTGGGVSIRPIAFLVVGKDG--VYILPLDCQTHIYEKMIDTVPYVMEKIQEMC-------------Q-KNF-----S-------------- 136

WP_051985382_1 -MAEHPIQGLMQTAMENIKEMVDVNTIVGEPVQTADGG-VILPISRVGFGFAAGGSDFNV--D----EK------NGASH----EG----------GR-----LHPFGGGSGGGVSINPIAFLVVGKAG--VHIVPLDNQTHLVERIIDSVPGLIDKIQSMF---------------PG------N-------------- 130

WP_082926868_1 -MAEHPIQGLMHTALENIKEMVDVNTIVGEPVETPDGS-VILPISRVGFGFAAGGSDFNI--E----EH------DKTAG----E-----------SK-----ARPFGGGSGGGVSINPIAFLVVGKQG--VHIVPLDNQTHVVEKIIDSVPNVIDKIQSLF---------------PNS-----G-------------- 130

WP_068696002_1 -MAEHPIQGLMRTAMENIKAMVDVNTIVGAPVETKDGS-VILPISRVGFGFAAGGSDFRI--S----GE------HHEHH----AE-----NN---EV-----ARPFGGGSGGGVSINPIAFLVVGPQG--VNVVPLDNQTHVMEKIIDTVPGVINQLQNMF---------------N-N-----S-------------- 132

WP_036650001_1 -MSEHPIQGLMQTAMENIKVMVDVNTIVGDPVQTPDGS-VILPISKVGFGFAAGGSDFHV--E----DN-HKN--ATSTN----GQ-----SS---NEGK---PMAFGGGSGGGVSITPIAFLVVGKEG--VHIVPLDNQTHLLEKIIDSAPSLIDKVQSMF---------------PSK-DK--D-----KDSY----- 144

WP_055107729_1 -MSDHPIQGLMQTAMENIKDMVDVNTIVGEPVETPDGS-VILPISRVGFGFAAGGSDYNV--D----ET------GGASS----ES----------DK-----NRPFGGGSGGGVSINPIAFLVVGQQG--VHIVPLDNQTHLFEKMIDTVPYVMDKIQSML---------------PNN-----G-------------- 131

WP_099476817_1 -MAEHPIQGLMQTAMENIKGMVDVNTIVGDPVNTPDGS-VILPISKVAFGFAAGGSDYNI--E----AG-GTR--H-SAD----GM-----HG---HS-S---THPFGGGSGGGVSIRPIAFLVVGKEG--VHIVPLDNQTHLFERIIDATPYLLEQIQAMF-------------Q-SGS-NANAN-----NGGN----- 145

WP_013371720_1 -MSDHPIQGLMQTAMENIKAMVDVNTIVGDAVETPDGS-VILPISKVGFGFAAGGSDFNV--D----EE-ALH--TSSAS----GN-----HS---GK-E---VHPFGGGSGGGVSIHPIAFLVVGKQG--AQIVPLDNQTHLFEKIIDSTPYLIDKVQSIF--------QN-----SGS-NM----------------- 140

WP_014282132_1 -MSDHPIQGLMQTAMENIKAMVDVNTIVGDAVETPDGS-VILPISKVGFGFAAGGSDFNV--D----EE-ALH--TSSAS----GA-----HS---GK-E---GHPFGGGSGGGVSIHPIAFLVVGKQG--AQIVPLDNQTHLIEKLIDSTPYLIDKVQSIF--------RN-----ANV-DL----------------- 140

WP_023989202_1 -MSDHPIQGLMQTAMENIKAMVDVNTIVGDAVETPDGS-VILPISKVGFGFAAGGSDFNV--D----EE-ALH--TSSAS----GN-----HS---GK-E---GHPFGGGSGGGVSIHPIAFLVVGKQG--AQIVPLDNQTHLIEKLIDSTPYLIDKVQSIF--------QN-----AGG-NL----------------- 140

WP_071641433_1 -MSDHPIQGLMQTAMENIKAMVDVNTIVGDAVETPDGS-VILPISKVGFGFAAGGSDFNV--D----EE-ALH--TSSAS----GN-----HS---GK-E---GHPFGGGSGGGVSIHPIAFLVVGKQG--AQIVPLDNQTHLIEKLIDSTPYLIDKVQSIF--------QN-----AGG-NL----------------- 140

WP_094154700_1 -MSDHPIQGLMQTAMENIKAMVDVNTIVGEAVETPDGS-VILPISKVGFGFAAGGSDFNV--D----EE-EHH--TSSAA----GN-----HS---GK-E---GHPFGGGSGGGVSIQPIAFLVVGKQG--AQIVPLDNQTHLIEKLIDSTPYLIDKVQSIF--------QN-----VGG-GM----------------- 140

WP_016822238_1 -MSDHPIQGLMQTAMENIKAMVDVNTIVGDAVETPDGS-VILPISKVGFGFAAGGSDFNV--D----EE-SLH--TSS-S----GN-----HS---GK-E---VHPFGGGSGGGVSIHPIAFLVVGKQG--AQIVPLDNQTHLFEKIIDSTPYLIDKVQSIF--------QN-----SGS-NM----------------- 139

WP_076170059_1 -MAEHPIQGLMQTAMENIKGMVDVNTIVGDPVETPDGS-VILPISKVGFGFAAGGSDFNA--D----DI-SKS--VSAAP----GH-----ES---K------SLPFGGGSGGGVSIRPIAFLVVGKAG--VNIVPLDNQTHLFEKLIDSAPSMVDKITSMF-------------Q-TNT-QT--G-----AVNPAQTTT 147

WP_036676154_1 -MSDHPIQGLMQTAMENIKGMVDVNTIVGDPVQTPDGS-IILPISKVAFGFAAGGSDYRV--E----DS------ISGSS----SQ-----G----GV-K---MLPFGGGSGGGVSIRPIAFLVVGKDG--VNIVPLDNQTHLFEKIIDATPGLIDKIQTMF-QTSN-A----------------A-----AQTP----- 139

WP_039875042_1 -MSDHPIQGLMQTAMENIKGMVDVNTIVGDPVETPDGS-VILPISKVAFGFAAGGSDFRV--E----DD------APGIN----GS-----GS---GV-K---MLPFGGGSGGGVSIRPIAFLVVGREG--VHIVPLDNQTHLFEKIIDAAPNLIDKIQNMI-------------Q-SGT-PV--G-----AEIV----- 141

WP_042178329_1 -MSDHPIQGLMQTAMENIKGMVDVNTIVGDPVETPDGS-VILPISKVAFGFAAGGSDFRV--E----DD------GPGVN----GS-----GS---GV-K---MLPFGGGSGGGVSIRPIAFLVVGREG--VHIVPLDNQTHLFEKIIDAAPNLIDKIQNMI-------------Q-SGT-PV--G-----AEIV----- 141

WP_084146924_1 -MNEHPIQGLMQTAMENIKGMVDVNTIVGDPVETPDGS-IILPISKVAFGFAAGGSDFRI--E----EDEKTSGTAAGST----TG-----AA---SA-K---MLPFGGGSGGGVSIRPIAFLVVGKDG--VHIVPLDNQTHIFEKIIDATPGLIDKIQSMF---------------PGS-GQ--G-----AMSQ----- 146

WP_009595256_1 -MAEHPIQGLMQTAMENIKGMVDVNTIVGDPVNTPDGS-VILPISKVAFGFAAGGSDYNV--E----AG-GTH--HNTAN----GT-----HT---DH-N---ALPFGGGSGGGVSIRPIAFLVVGKEG--VHIVPLDNQTHLFERIIDATPYLLDQIQSMF-------------Q-SGS-NA-AG-----STTP----- 145

WP_039301696_1 -MSDHPIQGLMQTAMENIKGMVDVNTIVGDPVETPDGS-IILPISKVAFGFAAGGSDFRV--E----DD------APGVN----GS-----GT---GV-K---MLPFGGGSGGGVSIRPIAFLVVGRDG--VHIVPLDNQTHLFEKIIDATPNLIDKIQNMF-------------QPNGM-PL--G-----ADAA----- 142

WP_042215611_1 -MSDHPIQGLMQTAMENIKGMVDVNTIVGDPVETPDGS-IILPISKVAFGFAAGGSDFRV--E----DD------APGVN----GS-----GT---GV-K---MLPFGGGSGGGVSIRPIAFLVVGRDG--VHIVPLDNQTHLFEKIIDATPNLIDKIQNMF-------------QPGGI-PV--G-----NDAH----- 142

WP_042237694_1 -MSDHPIQGLMQTAMENIKGMVDVNTIVGDPVETPDGS-IILPISKVAFGFAAGGSDFRV--E----DD------APGVN----GS-----GT---GV-K---MLPFGGGSGGGVSIRPIAFLVVGRDG--VHIVPLDNQTHLFEKIIDATPNLIDKIQNMF-------------QPNGM-PL--G-----ADAP----- 142

WP_006209426_1 -MAEHPIQGLMQTAMENIKGMVDVNTIVGDPVNTPDGS-VILPISKVAFGFAAGGSDYNV--E----AG-GTH--HHSTD----GT-----HS---DH-I---SHPFGGGSGGGVSIRPIAFLVVGKEG--VHIVPLDNQTHLFERIIDSTPYLLEQIQSMF-------------Q-TGN-ST--------GTTP----- 143

WP_081951173_1 -MSDHPIQGLMQTAMENIKGMVDVNTIVGDPVQTPDGS-IILPISKVAFGFAAGGSDYRV--E----DS------ISGSS----SQ-----G----GV-K---MLPFGGGSGGGVSIRPIAFLVVGKDG--VNIVPLDNQTHLFEKIIDATPGLIDKIQTMF-QSSS-T-SG-----QAG-ST--A-----TQAP----- 146

WP_042129645_1 -MSDHPIQGLMQTAMENIKGMVDVNTIVGDPVQTPDGS-IILPISKVAFGFAAGGSDYRV--E----DS------ISGSS----SQ-----G----GV-K---MLPFGGGSGGGVSIRPIAFLVVGKDG--VNIVPLDNQTHLFEKIIDATPGLIDKIQTMF-QSSS-SSSG-----QAG-ST--A-----TQAP----- 147

WP_038594603_1 -MSDHPIQGLMQTAMENIKGMVDVNTIVGDPVETPDGS-VILPISKVAFGFAAGGSDFRV--E----DD------APGVN----GS-----GS---SV-K---MLPFGGGSGGGVSIRPIAFLVVGREG--VHIVPLDNQTHLFEKIIDATPALIDKIQNMF-------------Q-NGA-PV--G-----AEVV----- 141

WP_020427525_1 -MSEHPIQGLMQTAMENIKGMVDVNTIVGDPVETPDGS-VILPISKVAFGFAAGGSDYRV--E----DD------APGIN----GA-----GS---GV-K---MLPFGGGSGGGVSIRPIAFLVVGREG--VHIVPLDNQTHLFEKIIDATPGLIDKIQTMFPSSGS-SQNDASPQ-NGT-PV--G-----SQVV----- 153

WP_025706964_1 -MSDHPIQGLMQTAMENIKGMVDVNTIVGDAVETPDGS-VILPISKVAFGFAAGGSDFRV--E----DD------APGIN----GT-----GS---GV-K---MLPFGGGSGGGVSIRPIAFLVVGKEG--VHIVPLDNQTHLFEKIIDATPGLIDKIQTMFPSNAS-SQNGESSQ-NGT-PV--G-----SQVV----- 153

WP_038697129_1 -MSEHPIQGLMQTAMENIKAMVDVNTIVGEPVETKDGS-IILPISKVAFGFAAGGSDFHV--E----ED------DKHAQ----GQ-----NG---GV-K---MLPFGGGSGGGVSIRPIAFLVVGREG--VHIVPLDNQTHIFEKIIDATPGLIDKIQTMF-QTNGIN--------TGA-VP--G-----AAPY----- 146

WP_087916963_1 -MSDHPIQGLMQTAMENIKGMVDVNTIVGDPVETPDGT-VILPISKVAFGFAAGGSDFRV--E----DD------TPTAA----G-----------GV-K---MLPFGGGSGGGVSIRPIAFLVVGRDG--VHIVPLDNQTHLFEKIIDATPGIIDKIQNMF-------------Q-TQT-QT--P-----DPQK----- 138

WP_025696984_1 -MSDHPIQGLMQTAMENIKGMVDVNTIVGEPVQTSDGS-TILPISKVAFGFAAGGSDFSV--E----DD------NKQAA----GQ-----N----GV-K---LLPFGGGSGGGVSIRPIAFLVVSQQG--VHIVPLDNQTHIFEKIIDATPGLIDKIQSMF-PAQG-G--------QGG-QG--GQGGNATAQQ----- 149

WP_042207356_1 -MSDHPIQGLMQTAMENIKGMVDVNTIVGEPVQTSDGS-TILPISKVAFGFAAGGSDFNV--E----DE------DKPAN----GQ-----S----GV-K---LLPFGGGSGGGVSIRPIAFLVVGQQG--VHIVPLDNQTHIFEKIIDATPGLIDKIQSMF-PAQG-G--------QGG-QG--G---NATAQQ----- 146

WP_014281971_1 -------------------------------------------------------------------------------------------------- 134

WP_094155205_1 -------------------------------------------------------------------------------------------------- 134

WP_038596748_1 -------------------------------------------------------------------------------------------------- 120

WP_025698007_1 -------------------------------------------------------------------------------------------------- 122

WP_042205911_1 -------------------------------------------------------------------------------------------------- 120

WP_025335629_1 ----NT-Q---------AP---P--P-VPSE-AV-TFTPST----PV--------------------------------------------------- 163

WP_081793606_1 ----GT-S-------------TM--F-QTSG-TA-GLNGGT----------------------GMAGATGAAGMSGAGTTGAGATGNGTGSSGSGNFI 191

WP_046231990_1 TNM-NS-G-------------YM--S---------TTDPTI----------------------I---------------------------------- 162

WP_099519904_1 TNM-NS-G-------------YM--S---------TTDSTI----------------------I---------------------------------- 162

WP_005546833_1 -------S------------------------------------------------------------------------------------------ 151

WP_062492715_1 -------T-------------DV--L---------ESATIV----------------------D---------------------------------- 157

WP_082084126_1 ----TM-N-------------TT--ITTPIT-TSGINNDQL----------------------V---------------------------------- 158

WP_087433119_1 -------P------------------------------------------------------------------------------------------ 151

WP_042162615_1 ----H--D-------------YI--S---------DGSDTL----------------------I---------------------------------- 152

WP_015254778_1 ----HV-S-------------DA--Q-IETE-AT-TANTPV----------------------V---------------------------------- 158

WP_023482608_1 ----QD-Q---------GTE-VN--A-TIKT-EP-TAPNIQ-------------I------------------------------------------- 168

WP_027085005_1 ----SQ-Q---------DSA-ET--S-NT---LT-TQSSHS--------D-KL-M------------------------------------------- 165

WP_006039315_1 ----HS-N-------------HE--L---------SSENPI----------------------I---------------------------------- 154

WP_015843958_1 ----HEHD-------------YI--T---------DGNDTL----------------------I---------------------------------- 157

WP_091188969_1 ----SS-D-------------YI--T---------DGNDTL----------------------I---------------------------------- 155

WP_044876390_1 ----GS-N---------NAA-SK--S-ENTV-LY-GDDSPE--------Y-PI-L------------------------------------------- 162

WP_082034320_1 ----MN-A-------------GMTGM-TPGM-NQ-GMNQGI----------------------N-QGMNQGTQTQTQNTTTAGTNGN---PNSSNNVM 190

WP_068657744_1 ----KS-N---------SNN-KN--A-NNSNTGT-PGSAPI--------G-NM-SELTN----L---------------------------------- 169

WP_010273939_1 ---------------------GN--K-QNEP-HL-DTHQDE-------------LQPLA--------------------------------------- 152

WP_068609309_1 ----GN---------------------NSDT----TDEGNT------------------------TNIIV---------------------------- 150

WP_089523778_1 ----RV-E-------------ST--L---------INTDPI----------------------I---------------------------------- 152

WP_013916453_1 ----SS-S---------ANA-GA--T-TVTN-NI-ENQNFI-------------V------------------------------------------- 168

WP_054819900_1 -------K---------KDA-GE--T-STVT-SV-ENENYI-------------V------------------------------------------- 155

WP_074095666_1 ----TT-P---------TNA-TG--V-PVTP-DP-STYS----------------------------------------------------------- 160

WP_016311757_1 ----AT---------------------ANAA----MATAQA-----------------------------------AQMQESAVRTG------INPFA 163

WP_062324412_1 ----TA-Q---------PAE-SD--I-PVTP-NP-TTFS----------------------------------------------------------- 160

WP_044479062_1 ----HV---------------------VVTN----QRAAES--------------------------------------EAPLL-------------- 149

WP_042228524_1 ----SA-S---------GLG-GS--G-TSNI-NI-EADTIK-------------M------------------------------------------- 158

WP_108465979_1 ----GS---------------------NNGS----NSDSQN-------------------------------AQSSTSSHSHSSSSG-------QPFA 161

WP_068649015_1 ----KP-N---------NSN------------GS-STTAPA--------G-GI-IS------------------------------------------ 155

WP_051985382_1 ----GT---------------------ANAG----MMMGQT------------------------FQAAQAPQTAQAGMNEPNVNPAPA----VNPFA 171

WP_082926868_1 ----TN---------------------RTLG----EQRGGT------------------------GNAYPASQMNVTVNEVPHTQQG-------QTLL 168

WP_068696002_1 ----GS---------------------STMV----VSETPT------------------------FQSRV---------------------------- 149

WP_036650001_1 ----KG-K---------VSD--P--V-HQSI-HD-TSNPSY-S------------------------------------------------------- 165

WP_055107729_1 ----SG-----------------------TA----QSASIQ--------------------------------------GIPLANQN-N----GNTYS 155

WP_099476817_1 ----VS-A-Y---------------------------------------------------------------------------------------- 149

WP_013371720_1 ----QT-P---------PPQ-VP--A-NANT-DQ-SQSGPA----------YL--------------------------------------------- 163

WP_014282132_1 ----HT-P---------PPQ-VP--V-DVNT-GQ-NHTGPA----------SL--------------------------------------------- 163

WP_023989202_1 ----QT-P---------PPQ-VP--V-DVAT-DH-SNSGPA----------YL--------------------------------------------- 163

WP_071641433_1 ----QT-P---------PPQ-VP--V-DVAT-DH-SHSGPA----------YL--------------------------------------------- 163

WP_094154700_1 ----QT-P---------PPQ-VP--V-NVTT-DP-SQSGPA----------YL--------------------------------------------- 163

WP_016822238_1 ----QT-P---------PPQ-VP--A-NANT-DQ-SQSGPA----------YL--------------------------------------------- 162

WP_076170059_1 SEINPS-L-F---------------------------------------------------------------------------------------- 155

WP_036676154_1 ----PT-P-------ATPAA-AP--V-KTDP-ST-SQSSTH--------------------------------------------------------- 162

WP_039875042_1 ----GA-P---------GTKTTT--V-KTES-VH-SNPSAH--------------------------------------------------------- 163

WP_042178329_1 ----GA-P---------GSKTTT--V-KTET-VQ-SNSSAH--------------------------------------------------------- 163

WP_084146924_1 ----QM-P---QSQ---MPQ-SQ--M-PPTP-PS-STPPTQPLNQTVVKGETITINEAPDNKKH---------------------------------- 193

WP_009595256_1 ----PP-A-Y---------------------------------------------------------------------------------------- 149

WP_039301696_1 ----GT-T---------VTK-TV--V-KTEP--D-THSSAH--------------------------------------------------------- 162

WP_042215611_1 ----VA-E---------VTK-TV--V-KTEP--T-THSSAH--------------------------------------------------------- 162

WP_042237694_1 ----GT-T---------VTK-TV--V-KTEP--D-THSAAH--------------------------------------------------------- 162

WP_006209426_1 ----PP-A-Y---------------------------------------------------------------------------------------- 147

WP_081951173_1 ----QA-PQAPQAPQAPASQ-AP--A-KPDP-ST-SQSSTH--------------------------------------------------------- 176

WP_042129645_1 ----QA-Q---------ASQ-AP--A-KPDP-ST-SQSSTH--------------------------------------------------------- 168

WP_038594603_1 ----GA-P---------AAK-QA--V-KTDP-AA-SNSSTH--------------------------------------------------------- 162

WP_020427525_1 ----AS-G---------TEK--A--V-KTPV--P-SDSSTH--------------------------------------------------------- 172

WP_025706964_1 ----AS-V---------TEK--V--V-KKPV--T-SDSSTH--------------------------------------------------------- 172

WP_038697129_1 ----NP-T---------IPA-TP--V-TEAG-DV-HYTSSR----P---------------------------------------------------- 168

WP_087916963_1 ----KD-A---------APV-VV--V-TSDH------------------------------------------------------------------- 151

WP_025696984_1 ----NT-Q---------QPA-QP--V-TVTQ------------------------------------------------------------------- 162

WP_042207356_1 ----NT-Q---------PPA-QP--V-TVTQ------------------------------------------------------------------- 159

**paen9.00958, DNA segregation ATPase FtsK**

WP_035119316_1 -------------------------------------MLNREVAALA---KR-KKK--RAP--FGASLKYEVYGILLITLSVIAISGEA-AVGRSLSKLFGFLLGIHYYMLALAGVWVGLYVMIKRSWPRGWTSRRSGFIIVVLGFTLWSAMAAIDNS-L---GPVD-A-VTPSNIVSQTTDQLKEQLW--A-VPPE-E- 143

WP_060536749_1 ----------------------------------------------------------------MSLLKYEIYGIILITISVIALSRQA-AVGHFLYYITSFILGKMYFVLPLAGAYIGLMTMLRQRWAQGWNSRWSGLVML-VCTLTLWSV----------------------------------LIY--V-PADQ--- 94

WP_084777845_1 MCYYGKSVSKPGGIEVAQKKSTGTRTGSSKARSSGSRT-------TS-T-KN-KKK--KPGS-LASALKYEIYGIVLITLSVIGLSGQA-FIGQLLSAGFGLVLGKAYAVIPVLGIYTGLYLMIRRGLPYGWSARKSGLLLVSLAGVLMLSIGEAQSH-I---ELEG-Q-MSAGGILSAAQGDLHRSLI--A-PNAE--- 174

WP_085981382_1 --------------------------------------M------LA---RK-KKK--KQS--FSKSLKYEIYGILLITCSVIALAGGA-AFGRSLSKISGLLLGKWYFFIPLVFIYIGLSVMIKRQWPSGWNSRKSGIMVIILALTMISTVHAVEQR-I---LPTADE-VSAGYILNDIHKALNLELV--Q-ATDT-G- 137

WP_042233096_1 ---------------------------------------------MA---KR-RKK--SKT--VKTNLMFELYGILILIFSVIALAREG-HVGRSFHYVCRFFIGTWDFLIPLICIYIALHVMVKRSWPSRWTTRKTGVLLIVAGLLVMNHINLFQAL-F----PKG-N-YEATTILSVTWDALKQGLQ--P-A--N--- 131

WP_013919428_1 ---------------------------------------------MS---KK-RKK--GKG--IKTNVKFELYGIAILTLSIIALSREG-SVARSLTYLFRFLIGTWDFVIPLVFIYAGLYAMMKREWPVWRTPRKYGIALILLSLLLMSHISVFAQL-Y----PKA-D-FTGSEIWSQTWYSLKQGLK--P-TEQG--- 133

WP_087432898_1 ---------------------------------------------MA---RR-KKK--KQS--FSKSLKYEIYGILLITCSVIALSGGA-AFGRSLSKISGLLLGKWYFIIPLTFIYIGLAVMIKRNWPKGWNPRKTGMVCIILALTMISTVHAVEQR-I---LPVADE-VGAGFIFQDIHKTLKAELL--Q-AQET-G- 136

WP_062408022_1 ---------------------------------------------MS---KK-RKR--GKG--LKTNLKFELYGILILTLSIIALSREG-SVARALTYLFRFLIGTWDFVIPLVFIYAALYAMMKRSWPVWRTSRKTGLLLILLSLLLMSHISLFAQL-Y----EKA-E-FTAGQIWSQTWIGMIDGLE--P-TMQG--- 133

WP_099520634_1 ---------------------------------------------MA---KK-RRG--KKS--LAANLKYEVYGILLITVSIIALSGEA-TVGRSLSKLFGLFLGKFYFVIALIGIYVGLVVMVKRMWPKGWSNRKTGMLVLVLAFTLWSSIAEIDRK-L---GDTT-L-LSGKVILNQLDSDLRGELL--T-SNPQ-D- 135

WP_082110759_1 --------------------------------------------------------------------------------------------------------------------------MVKRMWPKGWSNRKTGMLVLVLAFTLWSSIAEIDRK-L---GDTT-L-LSGKVILNQLDSDLRGELL--T-SNPQ-D- 67

WP_010269406_1 ---------------------------------------------MA---KR-RKK--KGD--GKLAIKFELYGIAIIILSVIALSRQG-SVARVLTYMSRFLIGVLDFVIPLICIYVAVYIMINRAWPKKWTSKKTGILLAIVGLILLNHMSMMTIV-E----TQG-NPVNSSLIITETWERMIAGIG--A-E-DA--- 133

WP_079940749_1 ---------------------------------------------MA---KR-RRR-RKKS--VRTGLIYELYGILILIFAVIALAREG-HVGRSFNYLCRFFVGTWDFIIPLICIYIALHVMIKRDWPRTWTTKKTGIVLIILGLLVMNHIRLFANL-F----PDG-E-FTAADIFRYSWQLMVQGIQ--P-E--H--- 132

WP_089523128_1 ---------------------------------------------MP---KKRRRK--RRT--FGENLKYEVYGILLITVSVIALSGEA-TVGRSLTKLFALVLGKFHFVLAVIGIAVGLYVMILRLWPTGWTYRRTGFLLLIMAMTLMSSIGEIDRK-L---APAD-I-MSGSAILAQLGQDIREGLL--T-SGTP-D- 136

WP_041854363_1 ---------------------------------------------MA---KR-KRK--KNA--IGSALKYEVYGILLITLSVIAMSGQGWPVDQALANLFGLFFGDFYFVVPLAGIYVGLMTMIKRKWPSRWSSRMTGIALILLFWLLTYTAGDVEAK-T---KPLG-E-PVSVNAIMMHARDIRELAT--P-GEFY-A- 136

WP_042189402_1 ---------------------------------------------MA-K-RK-KKK--KKAL-LGSVLKYEIYGILLITISVIALSGEA-AVGRSLSSMAGYLLGRFYFVLPLIGIFYGLMVMIHRRWPSSWNSRHTGVLLLLLSMCLMSTISAMEQK-L---GPLS-L-LHPGNVMTQIHNDLSGSLS--P-GVNN-S- 137

WP_068650580_1 ---------------------------------------------MA-K-RR-KKK--KAI--LSSILKYEIYGIILITLSVIALSGEA-TVGWSLSKIFGLLLGSFYFVVPLIGVYYGLMVMINRKWPNRWNTRKSGLLLVVLALTLMSSISSIERK-L---APIS-D-LSSSRIFAQAQSDLHGGLL--T-STDQ-I- 136

WP_042138096_1 ---------------------------------------------MA-KKRK-KKK--KKAL-LGSVLKYEIYGILLITISVIALSGEA-AVGRSLSSMAGYLLGRFYFVLPLAGIFYGLMVMIHRKWPSTWNSRYTGGLLLLLSMCLMSTISAMQQK-L---GPIG-M-LHPGNVLAQIHNDLSGSLS--P-GAGE-S- 138

WP_068611148_1 ---------------------------------------------MA---KK-KKK--KSN--ITSTLKFEVYGILIITLSVISLSGEA-TVGRALMKLFRLVLGGSYFVLPLIFIYIGLFVMWNRSWPKGWSLRRTGLFVILLAFTIHTHVAMIDLK-F---NPAG-V-LTAGSILETTQRELFNGLV--S-VPAG-T- 135

WP_040948739_1 ---------------------------------------------MA-RGKR-KKK--KTT--VRAEFKFELYGILMLIFSAIALFRAG-SVGRSLTILFRFTAGVLDWVIPLIAIYIAFFIMINRKWPRKWTPKKSGVLLLLIGFILIMEISFINTLAA----SRS-S-IGPSFIWHTTWDRLTQGLSLYP-A--A--- 136

WP_016312388_1 ---------------------------------------------MP-R-KR-KKR--KAA--IGSILKYEIYGIILITLSIIALSGEA-AVGRTLSKMAALILGKFYFVIPLIGIYIGLAVMINRKWPNRWNARRSGVLLAVLALTLMSTISAMQHK-L---APAV-P-LSTGNVLGQIHRDLQAALF--Q-PAVS-D- 136

WP_062326102_1 ---------------------------------------------MA-R-RK-KRK--KKAAAFSGVLKYEIYGIVLITLAVIALSGEA-TVGRSLSKMFGLMLGKFYFAIPLIGIYYGLMVMIHRKWPSGWTTRKTGLVLLVFALTMMSTVSAMHQK-L---IPVG-A-LEPGAVITQIHNDMQTELL--T-PAAP-GE 139

WP_038571908_1 ---------------------------------------------MA-K-RK-KKK--KKAL-LGSVLKYEIYGILLITISVIALSGEA-AVGRSLSSMAGYLLGRFYFVLPLVGIFYGLMVMIHRRWPSSWNSRHTGVLLLLLSMCLMSTISAMEQK-L---GPLS-L-LHPGNVMTQIHNDLSGSLS--P-GSND-S- 137

WP_042128826_1 ---------------------------------------------MA-K-RK-KKK--KKAL-LGSVLKYEIYGILLITISVIALSGEA-AVGRSLSSMAGYLLGRFYFVLPLVGIFYGLMVMIHRRWPSSWNSRHTGVLLLLLSMCLMSTISAMEQK-L---GPLS-L-LHPGNVMTQIHNDLSGSLS--P-GVNN-S- 137

WP_038593363_1 ---------------------------------------------MA-K-RK-KKK--KKAL-LGSVLKYEIYGIMLITISVIALSGEA-AVGRSLSSMAGYLLGRFYFVLPLAGIFYGLMVMIHRRWPSSWNSRHSGVLLLVLSLCLMSSISAMEQK-L---GPLS-M-LHPGNVMAQIHNDLSGALS--P-GSSN-S- 137

WP_055108403_1 ---------------------------------------------MA-R-KK-KKK--KAA--LGTMLKYEIYGIILITLSIIALSGEA-AVGRTLSKMSALVLGKFYFVIPLIGIFIGLSVMISRKWPSRWNTRRSGALLAVLSLVLMSTIFAMEKK-L---APSV-S-LSAGNIMSQIHHDLQTALF--G-PALE-D- 136

WP_042214780_1 ---------------------------------------------MA-KKRK-KKK--KKAL-LGSVLKYEIYGILLITISVIALSGEA-AVGRSLSSMAGYLLGRFYFVLPLAGIFYGLMVMIHRKWPSTWNSRYTGGLLLLLSMCLMSTISAMQQK-L---GPIG-M-LHPGNVLAQIHNDLSGSLS--P-GAGE-S- 138

WP_042237084_1 ---------------------------------------------MA-KKRK-KKK--KKAL-LGSVLKYEIYGILLITISVIALSGEA-AVGRSLSSMAGYLLGRFYFVLPLAGIFYGLMVMIHRKWPSTWNSRYTGGLLLLLSMCLMSTISAMQQK-L---GPIG-M-LHPGNVLAQIHNDLSGSLS--P-GAGE-S- 138

WP_076168009_1 ---------------------------------------------MA-K-RR-KKK--KAV--LTNVLKYEIYGIILITLSVIALSGEA-TVGWSLSKMFGLLLGRFYFVIPLVGIYLGLSVMIQRKWPKRWNTRRSGILLLILALVLMSSISSLEKK-L---GPVG-A-LSTGNILSQVHTDLQGALL--S-PSAR-G- 136

WP_044877535_1 ---------------------------------------------MA-K-RR-KKK--KAL--LSSVLKYEIYGIILITLSVIALSGEA-TVGWSLSKIFALFLGRFYFVFPLIGIYFGLMVMINRKWPTRWNSRKSGLVLLVLALTLMSSLSGLEKK-L---QPI--E-LNSGSIFKQGQMDLHEALL--S-PSVE-T- 135

WP_006036648_1 ---------------------------------------------MA---KRKRKK--RKS--IGESLKYEIYGILLITISVIALSGEA-AVGRSLSKLFGLLLGKFFFAIALVGIYVGLAVMIKRAWPKGWSNRKTGLLVLVLALTLSSSIAEIGRK-FGPVSSSV-E-VTSSDILHQLGSDLQGELL--A-PDSP-S- 139

WP_044478415_1 ---------------------------------------------MA-R-KR-KKK--KTA--IASMLKYEIYGIILITLSIIALSGEA-AVGRTLSKMSALMLGKFYFVIPLVGIYIGLAVMISRKWPSKWTARRSGLLLATLAFALMSTVSAMGQK-L---APVG-S-LSFSNIMGQIHRDLQGALF--S-PVSS-D- 136

WP_091181726_1 ---------------------------------------------MA---KK-RRK--KKS--IATQLKYEVYGILLITVSVIALSGEA-TVGRSLSKLFGLFLGKFYFVLALVGIYVGLVVMVKRAWPRGWTNRRTGLLVFVLALTLSSSIAEIDRK-L---LPTA-D-LTGRAILHQLTTDMSDELL--T-FNGT-D- 135

WP_036656364_1 ---------------------------------------------MA-K-RK-KKK--KKVL-LGSVLKYEIYGILLITISVIALSGEA-AVGRSLSSMAGYLLGRFYFILPLIGIFYGLMVMIHRKWPSSWNSRQSGVVLLVLSMCLMSSISSMEQK-L---GPLS-M-LHPNNVLSQIHNDLNGALS--P-TSNS-S- 137

WP_087916202_1 ---------------------------------------------MA-K-RR-KKK--KKAL-LGSVLKYEIYGIMLITISVIALSGEA-AVGRSLSSMAGYLLGRFYFVLPLAGIIYGLMVMIHRKWPSAWTSRHTGGLLLVCSLCLMSTISAMEQK-L---GPLS-L-LHPSNVMAQIHNDLSGALK--P-GADG-S- 137

WP_014281139_1 ---------------------------------------------MS-R-RR-KRK--KKAA-FGGVLKFEIYGIVLITLAVIALSGEA-AVGRSLSKMFGLVLGKFYFVIPLIGIYYGLMVMIHRKWPNQWNSRKTGLLLLVFAFTLMSSISSMEQR-L---GPIN-A-LQPGGVMSQIHIDMREQLL--S-PDKE-G- 137

WP_039277135_1 ---------------------------------------------MS-R-RR-KRK--KKAI-FGGVLKYEIYGIVLITLAVIALSGEA-AVGRSLSKMFGLVLGKFYFVIPLIGIYYGLMVMIHRKWPNQWNSRKTGLLLLVFAFTLMSSISSMEQR-L---GPIN-A-LQPGGVMSQIHIDMREQLL--S-PDQQ-G- 137

WP_013370740_1 ---------------------------------------------MS-R-RR-KRK--KKAI-FGGVLKYEIYGIVLITLAVIALSGEA-AVGRSLSKMFGLVLGKFYFVIPLIGIYYGLMVMIHRKWPNQWNSRKTGLLLLVFAFTLMSSISSMEQR-L---GPIN-A-LQPGGVMSQIHIDMREQLL--S-PDQQ-G- 137

WP_023988230_1 ---------------------------------------------MS-R-RR-KRK--KKAA-FGGVLKFEIYGIVLITLAVIALSGEA-AVGRSLSKMFGLVLGKFYFVIPLIGIYYGLMVMIHRKWPNQWNSRKTGLLLLVFAFTLMSSISSMEQR-L---GPIN-A-LQPGGVMTQIHIDMREQLL--S-PDQQ-G- 137

WP_058710517_1 ---------------------------------------------MS-R-RR-KRK--KKAI-FGGVLKYEIYGIVLITLAVIALSGEA-AVGRSLSKMFGLVLGKFYFVIPLIGIYYGLMVMIHRKWPNQWNSRKTGLLLLVFAFTLMSSISSMEQR-L---GPIN-A-LQPGGVMTQIHIDMREQLL--S-PDQQ-G- 137

WP_071640541_1 ---------------------------------------------MS-R-RR-KRK--KKAA-FGGVLKFEIYGIVLITLAVIALSGEA-AVGRSLSKMFGLVLGKFYFVIPLIGIYYGLMVMIHRKWPNQWNSRKTGLLLLVFAFTLMSSISSMEQR-L---GPIN-A-LQPGGVMTQIHIDMREQLL--S-PDQQ-G- 137

WP_053325270_1 ---------------------------------------------MS-R-RR-KRK--KKAA-FGGVLKFEIYGIVLITLAVIALSGEA-AVGRSLSKMFGLVLGKFYFVIPLIGIYYGLMVMIHRKWPNQWNSRKTGLLLLVFAFTLMSSISSMEQR-L---GPIN-A-LQPGGVMTQIHIDMREQLL--S-PDQQ-G- 137

WP_094155431_1 ---------------------------------------------MS-R-RR-KKK--KKAV-FGGVLKFEIYGIVLITLAVIALSGEA-AVGRSLSKMFGLVLGKFYFAIPLIGIYYGLMVMIHRKWPNQWNSRKTGLLLLVFAFTLMSSISSMEQR-L---GPIN-A-LQPGGVMSQIHIDMREQLL--S-PDKV-G- 137

WP_068655629_1 ---------------------------------------------MA-K-RR-KKK--KAI--LSSILKYEIYGIILITLSVIALSGEA-TVGWSLSKMFGLILGKFYFVVPLVGIYYGLMAMINRKWPNRWNSRKSGVLLLVLAFTLMASISSLEQK-L---APIS-N-LSSGTIFSQAKLDLHGELL--T-PSID-N- 136

WP_015736290_1 ---------------------------------------------MA-K-RK-RKK--KAA--FAAVLKYEIYGIVLMTLSVIALSGEA-PVGRSLSKMSGYLLGKYYFIIPLIGIYYGLMVMIHRKWPKRWNSRRTGVLLLVLSLTLMSSIAGLESK-L---APIG-Q-LNASSAMSQIHSDLQGELL--H-AGADDG- 137

WP_036642107_1 ---------------------------------------------MA-K-RK-RKK--KAA--FAAVLKYEIYGIVLMTLSVIALSGEA-PVGRSLSKMSGYLLGKYYFIIPLIGIYYGLMVMIHRKWPKRWNSRRTGVLLLILSLTLMSSIAGLESK-L---APIG-Q-LNASNAMSQIHSDLQGELL--D-GGVDDG- 137

WP_108465295_1 ---------------------------------------------MAGK-KR-KKK--RAT--LAGALKYEIYGIILITLSIIALSGEA-AVGRSLSKMAALLLGKFYFVIPLVGIYIGLYVMIKRKWPNGWNQRLSGTLLLVLTLALNSTISAMGQK-L---LPLV-G-LTPGHIFTQVRLDLQHALS--A-PDYG-G- 137

WP_045672049_1 ---------------------------------------------MG---KRKRKK--RRS--FGDNLKYEVYGILLITISVIALSGEA-TVGRSLSKLFGLVLGKFYFVLALVGVYAGLSVMVKRQWPSGWSNRKSGLLLLVLAFTLMSSMSEVSRK-L---EPSV-E-LTASAIMEQLGSDIRGALL--T-SDAS-G- 136

WP_036651575_1 ---------------------------------------------MA-K-RR-RKK--KAS--LTNVLKYEIYGIILITLSVIALSGEA-KVGWSLSKMFGLLLGRFYFIIPLIGIYLGLAVMIQRKWPSRWSTRKTGILLVVLALTLMSSISSLGTK-I---PP-N-I-ISPSSIITQVHNDLQGSLL--T-SAVN-G- 135

WP_074094588_1 ---------------------------------------------MA-R-RK-KRK--KKGAGFSGVLKYEIYGIVLITLAVIALSGEA-TVGRSLSKMFGLMLGKFYFAIPLVGIYYGLMVMIHRKWPSGWTTRKTGLVLLVFALTLMSTVSAMHQK-L---IPVG-A-LEPGAVITQVHNDMQTELL--T-PATP-GE 139

WP_020428017_1 ---------------------------------------------MA-K-RR-KKK--KKAL-LGSVLKYEIYGIMLITISVIALSGEA-AVGRSLSNMSGYLLGRFYFVLPLVGIFYGLMVMIHRRWPSKWTSRHSGALLLVLSMCLMSTISAMEQK-L---GPLS-M-LHPGNVLAQIHNDLSGSLS--S-GADG-S- 137

WP_025704675_1 ---------------------------------------------MA-K-RR-KKK--KKAL-LGSVLKYEIYGIMLITISVIALSGEA-AVGRSLSNISGYLFGRFYFVLPLVGIFYGLMVMIHRRWPSKWTSRHSGALLLVLSMCLMSTISAMEQK-L---GPLS-M-LHPGNVLAQIHNDLSGSLS--S-GAGG-S- 137

WP_047171161_1 --------------------------------------MLPGVAGVA-K-RR-KRKSKKKAT-IGSVLKYEIYGIMLITISVIALSGEA-AVGRSLSSMAGYLLGRFYFVLPLVGIIYGLMVMINRKWPSGWNSRYTGAVLLVLSLCLMSSISAMQQK-L---GPVN-M-LHPGNVLAQIHNDLSGALK--P-GASD-S- 146

WP_042177473_1 --------------------------------------MLPGVAGVA-K-RR-KRKSKKKAT-IGSVLKYEIYGIMLITISVIALSGEA-AVGRSLSSMAGYLLGRFYFVLPLVGIIYGLMVMIHRKWPSGWNSRYTGAVLLVVSLCLMSSISAMQQK-L---GPVN-M-LHPGNVMAQIHNDLSGALK--P-GTND-S- 146

WP_099479291_1 ---------------------------------------------MA-K-RR-KKK--KAA--FAAVLKYEIYGIVLMTLSVIALSGEA-PVGRSLAKMSGYLLGKYYFIIPLIGIYYGLMVMIHRKWPRRWNSRRTGVLLLVLALTLMSSIAGLGSK-L---APIG-Q-LTASNVMSQIHSDLQGELL--H-GEVRSD- 137

WP_038696628_1 ---------------------------------------------MA-K-RK-KRK-KKKAL-LGSVLKYEIYGILLITFSVIALSGEA-AVGRSLSNLAGYLLGRFYFVLPLVGIYYGLSVMIFRKWPTNWSSRYTGVFLLVLSLCLMSTISTMEQK-L---GPVS-M-LHPGNVISQTQKDLAAALK--P-GAES-D- 138

WP_015844967_1 ---------------------------------------------MA---KK-RRK--KKS--ITTQLKYEVYGILLITVSVIALSGEA-TVGRSLSKLFGLFLGKFYFVLALVGIYVGLVVMVKRAWPRGWTNRRTGLLVFVLALALSSSIAEIDRK-L---LPSS-D-LTGGAILHQLGQDMKDELL--A-FDGA-D- 135

WP_025335307_1 ---------------------------------------------MA-K-RK-RRK-KKKAL-LGSVLKYEIYGILLITFSVIALSGEA-AVGRSLSSLAGYLLGRFYFVLPLVGIYYGLMVMIYRKWPSNWNSRHSGVLLLVLSMCLMSSISAMEQK-L---GPLS-M-LDPGNVLSQTRKDLAGALL--P-SANG-T- 138

WP_036622367_1 ---------------------------------------------MA-R-KR-KKK--KAA--LGSMLKYEIYGIILITLSVIALSGEA-AVGRTLSKMSALILGKFYFVIPLIGIYIGLAVMINRKWPSRWNSRRSGLLLAVLAFSLMSTISAVQHK-L---VPAV-S-LSAGNIFGQIQRDLQGALF--Q-TAAG-D- 136

WP_042160596_1 ---------------------------------------------MA---KK-RRK--KKS--IASNLKYEVYGILLITVSVIALSGGA-TVGRSLSKLFGLFLGKFYFVLALVGIYVGLSVMVKRAWPKGWSHRRTGILIFVLALALWSSVAMIDKS-L---APSS-E-LSGGLIFEQLSSSIRLELL--T-FDGA-D- 135

WP_062492619_1 ---------------------------------------------MA---KR-RRK--RSS--LGTSLKYEVYGILLITISVIALSGEA-TVGRSLSKLFGLVLGKFYFVLALVGIYIGLAVMVKRRWPTGWSQRKSGLVLLVLALTLFSSIAEIDAK-L---AGAE-G-VTWTTILNQLGDDIRGALL--SGAGNP-D- 136

WP_068694663_1 ---------------------------------------------MAGK-KR-KRK--KTA--LTRILKYEIYGIILITLSIIALSGEA-AVGRSLSKMAALMLGKFYFVIPLIGIYAGLTVMIQRHWPNKWTSRKSGLVLVVLSFALMSTISAMELK-L---TPVG-A-LTPNHIFNQTRIDLQQALS--M-PGYG-G- 137

WP_025694771_1 ---------------------------------------------MA-K-RR-KRK-KKKAL-LGSVLKYEIYGILLITFSVIALSGEA-AVGRSLSSLAAYLLGRFYFVLPLIGMYYGLMVMIYRKWPSNWSSRHSGVLLLVCSMCLMSSISAMEQK-L---GPVS-M-LHPGNVLSQTQKDLAGALT--P-AVND-G- 138

WP_042207076_1 ---------------------------------------------MA-K-RR-KRK-KKKAL-LGSVLKYEIYGILLITFSVIALSGEA-AVGRSLSSLAAYLLGRFYFVLPLVGMYYGLMVMIYRKWPSNWSSRHSGVLLLVFSLCLMSSISAMEQK-L---GPVS-M-LHPGNVMSQTQKDLAGALT--A-SANG-G- 138

WP_068619226_1 ---------------------------------------------MA-R-KR-KKK--KAT--LGSVLKYEIYGIILITLSIIGLAGEA-AVGHTLFKMSAMVLGKFYFVIPLIGIYVGLTVMIHRKWPSKWSSRRTGGVLLVLAFTLMSTISAMQTK-L---APVL-P-LTPTNILRQIHHDLQFALT--Q-VGTG-V- 136

WP_041063710_1 ---------------------------------------------MA---KR-KKK--RAS--FGANLKYEIYGILLITLSMIAISGEA-AVGRSLSKLFAFLLGTHYYFLALAGIWAGLYVMIRRAWPRGWTSKRSGFVLVTLILTLWSAMGDIDKS-L---GAMD-G-ITASGIVAQTSAQLKEQLW--Q-VPPS-Q- 135

WP_035119316_1 D-VSITK--KDIGGGMAGALQYSVLYWLFGYYGAKFVLIIEFAIALLLITNRSYVEMGRAIRL-FLMRVMPL-----LAARL----S-------SG----------SK---LLRA------KAV-PVKGAGKP-----AAI--------------PA-I-------PLE------P-D--DDA-M---DEQ-PLMP---- 258

WP_060536749_1 L-ISLPA--RQAAGGYIGAVQMWLFMMLFGVIGTKLIAGVMLVISLMLITQLSVVDLLERIRM-YGAKAGTA-----VQSQW--N-A-------SR----------EA---LAEQ--RR--ERE-LAAEEAEL-----DVY--------------DE---------TEE------E-Y--EED----------------- 203

WP_084777845_1 M-SQITPFLRPIGGGYFGALLLSLLYLMVGMTGAKLASGVLLIIGVMLITQMSYVDLMNMIRR-KAGEAARS-----AGERL--K-E-------ERQVAQQRKIEAEK---KAEI--RA--KEK-AEAEEKRL-----AAF--------------SK---------SEG------E-E--AEE----------------- 295

WP_085981382_1 TAGTMLQ--KDISGGYIGAILYCTLFVLFGALGTKLLTIVMLFIGFMLATQLSYVDLVKLIRV-QVKKFAAI-----IKKKR----K-------AR----------LN---ERKR--ME--SKN-RKSAKKD------SDD--------------DY-MLD-----DEL------D-D--EEE---------NDS----- 251

WP_042233096_1 --GKILL--NDVGGGMIGAVIYSLLYFLFDTLGARLVEFALFAIGLILVTNLSYVEAMQKLRARGKRFFT------LLEKKF--Q-T-------MQ----------RG---KGKS--SS--ASK-TKGKAAVS-----ATK---SE---------RT---------YKEPDFPYDD-E--FDE-E-------VYRP-D-- 253

WP_013919428_1 --QAILR--DQVGGGMIGASLYSVLFMLFSNLGARLIQYMLFAAGFMMVTGLSVADIVDKVR--GRKAFKDTLFGRYLQRKL--Q-E-------RQ----------KQ---AAKA--VA--AAE-KKRKAAEA-----AAA---AD---------EP---------EDEDELP--D-E--LDE-E-------TVKP-Q-- 257

WP_087432898_1 TAGTMLQ--KDISGGYVGAVLYCALFMLFGSLGSKLLTIVLLFIGFMLATQLSYVELVRLLRS-QIKKLAAV-----MKKKR----K-------AR----------LN---ERKL--QD--GKQ-QRAAAKH------PVK--------------EY-MLP-----DSL------D-D--EED---------EDGPPRR- 254

WP_062408022_1 --QEILT--AGVGGGMIGALIYSVLYYLFDNLGARLIQYTLFIAGFLLVTGISLGDVLNRVR--GRKPFADTLLGQYIKRKL--L-E-------RH----------QR---R------Q--AVN-KVKKRKPA-----VAY---IP---------EP---------DE---------E--YDE-E-------TYKP-E-- 247

WP_099520634_1 L-RPLKD--KAISGGYVGALQYSVLFTLFGKIGAQLLMLVMIAISIMLITGKSYVELFKTLRT-RFVRMLKL-----LAAKW----S-------DY----------SA---ERAA--AQ--SSR-NASVVSSA-----STV--------------LT-S-------DNT------I-D--DDE-----EDF-APRP---- 251

WP_082110759_1 L-RPLKD--KAISGGYVGALQYSVLFTLFGKIGAQLLMLVMIAISIMLITGKSYVELFKTLRT-RFARMLKL-----LAAKW----S-------DY----------AA---ERAA--TQ--SSR-SASTVSAA-----STV--------------LT-S-------DNT------I-D--DDE-----EDF-ALRP---- 183

WP_010269406_1 --SLLLT--SPVGGGMIGAFLYAALHMLFGVWGSRLIVYAIFAAAFILVTGISLVDVFKK----GSAWLTS--VRDKLIKRF--S-K---PSG-SK----------KK---KLNT--RQ--IPS-SKLKKPVE-----VIV---AD---------EE---------DEE------E-A--IES-P-------IIEP-D-- 252

WP_079940749_1 --GNILT--DKVGGGMIGAAEYSVLYFLFDTLGAKLVMYALFAIGFILITGLSYVDAGKKVRI-GSTAFFEK-----LTARF--R-T-------SR----------KN---RLVR--KA--AER-SERKVKAG-----RRA---VE---------AD---------EPEAQFY--E-D--YEE-E-------L--P-S-- 250

WP_089523128_1 NPGSPVQ--QSVSGGYLGAMQYALLYTLFGYFGSKLLLIVMFAISAMLITGKSYVDIGRAIRT-RTTNLAKL-----LHAKA----S-------AR----------PR---KPRA--PKLPSKK-GEITAATA-----SAG--------------SD-HLSYGLNGDGH------D-D--DYG-----STIVTPAP---- 263

WP_041854363_1 --MPIIE--KNLSGGYIGALAYALLFFLFGNFGAKFVTVVLLLIGIMLATGKSYVELALMLRR-QIGRFVQL-----IRLKL----A-------DS----------RR---RRPA--AA--QAV-PVQAEDEL-----PADKPDADNPGSPLILIDD---------EDL------M-D--DDD---------SFVP-PK- 263

WP_042189402_1 N-VYMLG--KDISGGYIGGLEYAALLWLFGSLGAKLLMIVMLAISFMLITNLSYVEIITLLRV-RTVKFVEG-----IRLHA----A-------NR----------PK---AVPV--AA--RPS-KAAAPAKT--R--PVK--------------QP-VYD-----EDD------E-E-EEDD---------RYLP---- 254

WP_068650580_1 E-HSMMD--KDIGGGYVGAIQYTALFWLFGNTGAKLLMIVMFIISFMLVTNLSYIDLMRIVRS-RVVKVVSF-----VHKQF----S-------MR----------QTVGKKTSS--PA--RGL-AERNRRL---L--PGD--------------ED-E-------DDF------D-E--VVL---------PK-V---- 251

WP_042138096_1 S-VYMLG--KDISGGYIGALEYAALLWLFGNLGAKLLMIVLLAISFMLITNLSYVELFTLLRV-RAVKLVEG-----IKLRS----A-------NR----------PA---AVPV--VP--RAA-RNSTPAAP--Q--PAD--------------DD-DYY-----EDD------E-E--ENN---------NQLP---- 254

WP_068611148_1 G-ASPVG--KDYGGGYTGGLGYALLFFLFGYTGTKLMIYTMFIIGFMLITGVSYVELAQAARN-KGKNAVGR-----MDKKI-IH-V-------LK----------QW---QANR--SL--NVA-SRNTDRSEN-M--PSI--------------HE---D-----DEF------D-E--ENY---------TPLK---- 252

WP_040948739_1 --GDILT--RGVGGGIIGAVLYSALDQLFSYEGTVLVVIALFAIGIMLVTGISFVEIGKRIRA-SLKAFN---VG--MKNRL--L-DWFGAGGTKR----------RS---TARG--TK--GRT-ALKEINTE-----PMH---SQ---------QD---------EED------E-D--DED-D-------VM------ 256

WP_016312388_1 AGISLLG--LDISGGYVGALEFALLYSLFGMAGAKLIMIVLLAISFMLVTGLSYVDLFRMLNS-RLAVLAEN-----FGKKM--R-M-------LR----------PI---PVRN--KA--KRA-VKKAEPVV-----TTY--------------DD-P-E-----DEE------S-P--LDE---------EAVPAA-- 254

WP_062326102_1 R-DSMLN--KDISGGYIGAAQFALFLWLFGSLGARLIMIVMFIISFMLVTNLSYVDLIRIFRT-KVWDAGSA-----MYKKLESRPA-------AR----------TV---PSSA--GK--KNG-NTRKVVPVP-V--DDY--------------EE-D-E-----EDM------P-D--QHL------------P---- 256

WP_038571908_1 N-VYMLG--KDISGGYIGGLEYAALLWLFGSLGAKLLMIVMLAISFMLITNLSYVEIFTLLRV-RSVKFVEG-----IRLHA----A-------NR----------PK---AVPV--AA--RPS-RAAAPAKA--R--QVK--------------QP-VYD-----DED------E-E-DEDD---------RYLP---- 254

WP_042128826_1 N-VYMLG--KDISGGYIGGLEYAALLWLFGSLGAKLLMIVMLAISFMLITNLSYVEIITLLRV-RTVKFVEG-----IRLHA----A-------NR----------PK---AVPV--AA--RPS-KAAAPAKT--R--AVK--------------QP-VYE-----EDD------E-E-EEDD---------RYLP---- 254

WP_038593363_1 S-VYMLG--KDISGGYIGALEFAALLWLFGTLGAKLLMIVMMAISFMLVTNLSYVELFSLLRV-RAGKLLEG-----IRLKA----A-------NR----------PA---AVPV--AA--RST-RTGRTPAP--Q--PVQ--------------EL-D-------EDE------E-D--EDE---------QALP---- 251

WP_055108403_1 TGFSLLG--LDISGGYIGGLEFALLYSLFGIAGAKLIMIVMMAISFMLITGLSYVDLFRLIRK-NLRGAGTS-----FSRKL--K-L-------LR----------PV---PVNS--RP--KRT-PAES-VQA-----NRY--------------E----E-----EEA------E-E--EEE---------LPVPRG-- 251

WP_042214780_1 S-VYMLG--KDISGGYIGALEYAALLWLFGNLGAKLLMIVLLAISFMLITNLSYVELFTLLRV-RAVKLVEG-----IKLRS----A-------NR----------PA---AVPV--VP--RAT-RNSTPPAP--Q--PAD--------------DD-DYY-----EDD------E-E--ENN---------QQLP---- 254

WP_042237084_1 S-VYMLG--KDISGGYIGALEYAALLWLFGNLGAKLLMIVLLAISFMLITNLSYVELFTLLRV-RAVKLVEG-----IKLRS----A-------NR----------PA---AVPV--VP--RAA-RNSTPAAP--Q--PAD--------------DD-DYY-----EDD------E-E--ENN---------NQLP---- 254

WP_076168009_1 D-HSMLN--KDISGGYIGAIEFAALYWLFGTLGSKLMMIVMFIISFMLITNLSYVDLMRIFRS-RLVKAGEN-----VQKRM----D-------NR----------RQ---SAKV--AS--KRA-ASSRQKKPQPL--PAD--------------DD-E-------DEL------DED--FMP---------PK-P---- 252

WP_044877535_1 E-RSMMD--KDIGGGYVGTVQYTVLFWLFGTIGAKLMMIVMFVISFMLITNLSYIDLIRIVRT-KVWKTGSS-----LTKQF----V-------TH----------RQSVNQTKS--NA--KSA-TVHKSRL---L--PED--------------ED-E-------ED-------E-D--FSL---------PK-T---- 249

WP_006036648_1 T--PVMS--RDIAGGYVGAVQYTILYALFGKYGSKFLMIVMYAIAIMLITGRSYVDIADGVRT-RLGRLITL-----LRAKY----A-------GR----------RP---RTVA--VA--ARA-NAANTGQS-----PSL--------------SR-RTIDDFDDDAE------D-E--DDD-----AQD-FADA---- 261

WP_044478415_1 SGFSLLQ--LNISGGYIGGLEFALLYSLFGLAGARLIMIVMLAISFMLVTGLSYVELGKILRG-KVGGLGQQ-----FGKKL--R-M-------LR----------PV---PVKG---G--DRP-VRKAAAKK-----PVI--------------EE-T-P-----PLE------P-I--EAE---------EAAPSVRQ 255

WP_091181726_1 D-RPISQ--RAISGGYIGAIEYSMLFSLFGYFGSKLIAGVMYAISIMLLTGKSYVELARIGRD-KTIRLYKL-----LSAKW----S-------KG----------AS---LRSAAKAK--SSK-AVQ----P-----PVF--------------VQ-S-------DAI------D-D--DDD-----DDYAAALP---- 250

WP_036656364_1 E-VFMLG--KDISGGYLGALEYAALLWLFGNLGAKLLMIVMLAISFMLITNLSYVELFGLFRQ-RTVKLIES-----IRLRA----A-------NR----------PQ---AVPV--TS----T-RAGKGKKE--P--VIQ--------------DD-DLD-----DDE------D-Y-NEGG---------QPLP---- 252

WP_087916202_1 T-VYMLG--KDISGGYIGALEYSALLFLFGSLGAKLLMIVMLAISFMLVTNLSYVELFTLLRV-RGVKLVEG-----IRKKA----A-------NR----------PK---AVPV--AA--KPS-R------S--R--AAA--------------PP-VLP-----DDD------E-DYVEED---------PLLP---- 249

WP_014281139_1 H-TSMLN--KDISGGYIGALQLTVLLWLFGLTGAKLIMIVMFVICFMLLTQLSYVDLVRIVRT-KMLAAGGS-----VRKKW----I-------GK----------AT---PLSA--SG--RGT-GNKKIKSES-I--PAY--------------EE-DMD-----DDF------D-E--MQP------------P---- 251

WP_039277135_1 H-TSMLN--KDISGGYVGALQLTVLLWLFGLTGAKLIMIVMFVICFMLMTQLSYVDLVRIVKT-KLLTAGGS-----VRKKW----T-------GK----------AT---PLSA--SS--KGS-GNRKPKSEP-I--PAY--------------EE-DMD-----DDF------D-E--MQP------------P---- 251

WP_013370740_1 H-TSMLN--KDISGGYVGALQLTVLLWLFGLTGAKLIMIVMFVICFMLMTQLSYVDLVRIVKT-KLLTAGGS-----VRKKW----T-------GK----------AT---PLSA--SS--KGS-GNRKPKSEP-I--PAY--------------EE-DMD-----DDF------D-E--MQP------------P---- 251

WP_023988230_1 H-TSMLN--KDISGGYVGALQLTVLLWLFGLTGAKLIMIVMFVICFMLMTQLSYVDLVRIVKT-KLLTVGGS-----VRKKW----T-------GK----------AT---PLSA--SS--KGS-GNRKIKSEP-I--PAY--------------EE-DMD-----DDF------D-E--MQP------------P---- 251

WP_058710517_1 H-TSMLN--KDISGGYVGALQLTVLLWLFGLTGAKLIMIVMFVICFMLMTQLSYVDLVRIVKT-KLLTAGGS-----VRKKW----T-------GK----------AT---PLSA--SS--KGS-GNRKPKSEP-I--PAY--------------EE-DMD-----DDF------D-E--MQP------------P---- 251

WP_071640541_1 H-TSMLN--KDISGGYVGALQLTVLLWLFGLTGAKLIMIVMFVICFMLMTQLSYVDLVRIVKT-KLLTVGGS-----VRKKW----T-------GK----------AT---PLSA--SS--KGS-GNRKIKSEP-I--PAY--------------EE-DMD-----DDF------D-E--MQP------------P---- 251

WP_053325270_1 H-TSMLN--KDISGGYVGALQLTVLLWLFGLTGAKLIMIVMFVICFMLMTQLSYVDLVRIVKT-KLLTVGGS-----VRKKW----T-------GK----------AT---PLSA--SS--KGS-GNRKIKSEP-I--PAY--------------EE-DMD-----DDF------D-E--MQP------------P---- 251

WP_094155431_1 H-TSMLN--KDISGGYVGALQLTVLLWLFGITGAKLIMIVMFVICFMLLTQLSYVDLVRIVRT-KLLTAGGS-----VRKKW----I-------GK----------AT---PLSA--SD--KG---NRKVKSEP-I--PAY--------------EE-DMD-----DDF------E-E--MQP------------P---- 249

WP_068655629_1 K-NSMMD--KDIGGGYVGAIQYSALFWLFGNMGAKLLRIVMFIISFMLITNLSYVELVRIVRT-RVMKVGSY-----VHKKL----S-------MR----------QHPAGNGST--KV--KSI-PERNRQL---L--PGD--------------ED-D-------DDF------D-V--DIL---------PK-V---- 251

WP_015736290_1 R-HPVMG--RDISGGYVGALQFALLLMLFGSIGAKLIVIVMLFISFMLITNLSYVDLMRMFRV-RVVKAGER-----IQKKM----N-------SQ----------QT---KSKA--AA--KAS-AKEQRIQ---L--PED--------------LDED-------DDL------D-D--MEG---------MRLP---- 251

WP_036642107_1 H-HPVMG--RDISGGYVGALQFAFLLMLFGSIGARLIMIVMLFISFMLITNLSYVDLMRMFRV-RVVKAGER-----IQKKV----N-------SQ----------QT---KSRA--AA--KSN-AKEQRMQ---L--PED--------------LD-D-------DDL------D-D--MES---------MRLP---- 250

WP_108465295_1 A-VAIFN--LDISGGYVGAAEFALLSMLFGLAGSKLIMIVMYAVSFMLITSLSFVDLAQMARS-GIGNLAKL-----AGKKM--R-M-------MK----------PV---PVSS--KA--SKA-SKASKAKPV-IREPEY---------------V-D-D-----DDE------D-Y--EEE---------VAQHQK-- 257

WP_045672049_1 G-GLATQ--QSISGGYAGAVQYALLYSLFGYFGAKFILIVMFAIAIMLITGRSYVDLARVVQR-RLHNLIKL-----IHAKA----A-------SR----------PV---KRTA--PK----T-SGLTALQP-----DDM--------------VD-HY------DDA------D-D--DFG-----GTL-QPAR---- 251

WP_036651575_1 D-RSMMN--KDISGGYIGALQFAVLFWLFGSLGSKLMLVVMFIISFMLITNLSYVDLLRIFRT-RVMKVGGE-----MHKRI----G-------SN----------RQ---QTPL--TK--SGK-STASTRAPL-L--PTE--------------DE-E-------DEF------D-D--FVV---------PK-S---- 249

WP_074094588_1 R-DSMLN--KDISGGYLGAGQFVLFLWLFGSLGARLIMIVMFVISFMLITNLSYVDLIRIFRT-KVWDAGST-----MYKKLESRTA-------TR----------SA---SVAD--DR--KKA-NARKVLPVP-V--EDD--------------ED-EYE-----DDL------E-E--QHL------------P---- 257

WP_020428017_1 E-VYMLG--KDISGGYVGALEYAALLWLFGSLGAKLLMIVMLAISFMLITNLSYVELFTLLRV-RVMKLAEG-----IRQRT----A-------NR----------PA---AVPV--TS--RPA-RTGRTAAP--Q--PVQ--------------D----------DDD------E-D--EDD---------QQLP---- 249

WP_025704675_1 E-VYMLG--KDISGGYVGALEYAALLWLFGSLGAKLLMIVMLAISFMLITNLSYVELFTLLRV-RVVKLAEG-----IRQKA----A-------NR----------PA---AVPV--NT--RPA-RTGRTAAP--Q--PVQ--------------D----------DGD------E-D--EDE---------QQLP---- 249

WP_047171161_1 S-VYLLG--KDISGGYLGALVFAGLLWLFGTLGAKLIMIVMLAISFMLVTNLSYVELFSLLRV-RSVKLLES-----LRQRA----A-------NR----------PA---AVPV--AP--RQS-RAGRAAAA--A--PPD--------------IE---------EDE------D-E--EDE---------PSLP---- 259

WP_042177473_1 S-VYLLG--KDISGGYVGALVFSALLWLFGTLGAKLIMIVMLAISFMLVTNLSYVELFSLLRL-RSVKLIEN-----LRQRA----A-------NR----------PA---AVPV--TP--RQT-RADRAVAA--A--PPG--------------IE---------EEE------E-E-EEDE---------SSLP---- 260

WP_099479291_1 Q-HPVMG--RDISGGYVGALQFALLIMLFGSLGARLIVIVMLFISFMLITNLSYVDLMRMFRV-RVVKAGER-----LQKKM----N-------SR----------QT---KSKA--AA--KTA-AKEERTP---L--PDD--------------LD---------DDL------D-D--MED---------LRLP---- 249

WP_038696628_1 S-VYMLG--KDIGGGYAGGLTFALLLMLFGTLGAKLIMIVMLAISFMLVTNLSYIELFGLLRV-RLVKLAKE-----LKKRA----A-------NR----------PK---AVPI--AS--RTSGGRGKAQ-------HVE--------------AE-E-D-----EED------E-E--EQE---------TSLP---- 251

WP_015844967_1 D-RPISQ--RAISGGYIGAMEYSMLFTLFGYFGSKLIAGVMYAIAIMLLTGKSYVELARIGRD-KSIRMFKL-----LAAKW----S-------KG----------AA---LRSA--SK--SNK-AVK----P-----PVF--------------VQ-S-------DAI------D-D--DDD----EDDYAPALP---- 249

WP_025335307_1 S-VYMLG--KDISGGYVGGLEFAFLLWLFGTLGAKLLMIVMLAISFMLVTNLSYIELFGLLRN-RAVKLAEG-----IRLRA----A-------SR----------PQ---AMPV--S-------NRSGDRAK--L--RSQ--------------SS-QDD-----DDE------D-D--EEE---------TSLP---- 250

WP_036622367_1 AGFSLLG--MDISGGYIGALEFVLLYSLFGMAGAKLIMIVMLAISFMLVTGLSYVDLFRLLRS-KLGGLSES-----FGRKM--R-L-------LR----------PV---PVRG--KG--RRP-EPKI-PAV-----TEY--------------DA-AYD-----AGE------S-E--EEE---------LPEPSAK- 255

WP_042160596_1 D-RPMNE--KDISGGYIGAIEYSMLFMLFGYFGAKLIMWVMFAISLMLMTGKSYVEMGRLVKG-KLIKLFKL-----FAAKW----N-------QR----------AA---LRAA---T--AAA-AAR----A-----PRT--------------AD-S-------QGY------A-Q--DDDAIDEDEDY-PLTP---- 251

WP_062492619_1 E--PMMQ--KAISGGYVGAIQYSILFGLFGYFGAKFVMIVMFAISVMLITGRSYVEMAKLGKE-KLQRLYTL-----LRAKY----A-------AS----------RK---TRRP--VA--PAA-VNVKAASP-----VHT--------------VH-SLPDDEEDDEE------N-E--GED-----EQY-APEA---- 258

WP_068694663_1 A-VSIFK--LDISGGYIGAAEFALLSMLFGLTGSKLILIVMFAISFMLITNLSFVELGRLVRT-RVFNLGEV-----ISRKL--R-T-------LR----------AV---PAGQ--KR--KAK-PAAVKASAA----PAF--------------DP-D-D-----DDE------D-YDPESE---------IPSGPA-- 257

WP_025694771_1 S-IYMLG--KDISGGYLGGLEFAVLLWLFGTLGAKLLMIVMLAISFMLVTNLSYIELFGILRI-RAVKLAEG-----IRLRA----A-------NR----------PQ---VVPV--S-------GRGRPK-------KVQ--------------EE-PEE-----EEE------E-E--ELE---------AGLP---- 247

WP_042207076_1 G-VYMLG--KDISGGYLGGLEFAVLLWLFGTLGAKLLMVVMLAISFMLVTNLSYIELFGILRN-RAVKLAEG-----IRLRA----A-------NR----------PQ---AVPV--S-------GRGRPK-------KVQ--------------EE-P-E-----EEE------E-E--ELE---------AGLP---- 246

WP_068619226_1 A--SMFN--LDISGGYVGALEFILLYVLFGITGAKLMMLVMFAISFMLITNLSYVELFSIIKD-RTSRTAAL-----LSKKY--R-L-------LR----------PT---PVAQ--GT--KSK-ERPVKIT------PAV--------------DE-M-E-----EDE------D-H--FPE---------QEQEGV-- 251

WP_041063710_1 D-VPVMQ--KDIGGGIVGAVQYSIFYWMFGYYGAKFILIIEIAIALMLITNRSYVEMARTIRL-FVLRVSPL-----IAARF----S-------SG----------RK---LLSA------KAV-PVKGPKRA-----VVQ--------------TP-M-------DSL------P-E--DDG-L---DEA-SLMP---- 250

WP_035119316_1 R-Q------RKTPLFFQLFH-----GK----------------PER-ESG-----SMPED------------Q-W-DED---EPVNLNIRP--SKKTDAAP-D--VKNMPSDVHTMH-GD--DDR-E-----------PD--G-----ELPQS-----------SV-TPPQ--E-QP----------------------- 342

WP_060536749_1 E--------GHSAKRKGLFT--W--GR---------KP-----TPE-SEP-----VETDE----SVYGNV--P---DHL---KET---IKA--Y----TTA----ERRDK------T-----ATA-S-PI--------KE--A---R-QATSV-QD-----EE-DLWDNVQ--T-EP--------EL------------- 288

WP_084777845_1 A--------NRNL--TGVLR-----GR---------KK-----KLA-AES-----EEQED---------L--P---QRE---GFK---ITG-VY----RVP----SELKE------R-----QTN-E-AL--------KT--S---V-EPSEI-QP-----DS-TA-APSE--A-LP--------DR------------- 372

WP_085981382_1 ---------SRTPVFMQIFG--KL-SE-------A-GK-----KRF-LAS-----KEEDE------------E-Y-NER-H-TAH---SSK-------PHT----ARLND--------------D-H-NI-HPEADT-AS--M---N-TVSNA-SKLNAL-LA-RI-EQDD--D-QS----------------------- 336

WP_042233096_1 P-K------RKKPRFLQLLK-----------------------QPD-PQQ-----DDDQD------------E---DDL---AVS---GTA-GGYS--EEK----VVYKV----------------------------DS----G---RDS----A--------AL-DMND----EP----------------------- 314

WP_013919428_1 R-KVRQKT-GKRSVFMELIK-----GS-RSDEE-K-RK-----KAK-TPE-----WDDEE------------E---EGV---SVY---SAE----T--EGR----GAFTT----------------------------PA----E---DEDDI-PA--------PL-TRTH----DP----------------------- 333

WP_087432898_1 I--------PKMPIFMQLF-----------------SR-----KRA-AEN-----ADEDE------------E-Y-GES---AIF---KGR-------AKA----ARLSD------------ADD-E-DD-GPIINA-AV--T---A-TAAKA-SS-ELQ-NG-HG-DLDA--A-VS----------------------- 334

WP_062408022_1 K-KNRGSG-KKRALFFELLNPSKG-GS-KSADK-V-SG-----GPD-SDH-----SSDSE------------E---EMV---TVY---TRA----E--DER----SLLSG----------------------------NE---------------P--------PV-AEFN----DP----------------------- 320

WP_099520634_1 V-K------NKKSLFFSWRQ-----SD-------------K--A----KA-----AASHD------------E-W-ELE---DDP---LHGESGRGEEGAA-V--PHWAED--WD---QD--WDS-E-----------QD--EFAAQTAPSTA-----------AS-STAA--A-VP---LPETSAS-LWPSEQEDLAAK 352

WP_082110759_1 A-K------NKKSLFFSWRQ-----SD-------------K--T----KA-----TVSHD------------E-W-ELE---DEP---LHGEAGRGEEGAA-V--PHWAEE--WD---QD--WDN-E-----------QD--GFAAQTAPSTV-----------SS-STAA--A-VP---LPETSAS-LWPSEQEDLTAK 284

WP_010269406_1 E--------KKPSVFVQLLS-------------------------K-KEK-----PAERD------------A---SPV---NLA---EQM--G-------------------------------------------------------------------------IKQE----EE----------------------- 290

WP_079940749_1 K-K------RKKPVFLELFQ----------------PK-----QAA-QDH-----IEEED------------T---DWI---EAH---EEA-------EEI----TVYQA----------------------------GQ-----------------------------NE----IP----------------------- 300

WP_089523128_1 A--------KGRSLFFSWGS-----KA-------A-QE-DK--EEQ-EDN-----NADDS------------D-W-AME---ESA---LRQ-------GAS-K--SRKAPV-----------VSL-R-----------GR--N-----TAEPM-----------GQ-SQ------PA-----------PWEDEEGEDWED 344

WP_041854363_1 Q-T----A-RRRTLFFPWRS----------------DA-----GRK-EED-----ASPAE----------------PEH---GNP---EAA-------DAA----VAMPG---------E--LNP-------------LE--------PVQGI-----------QP-ESEA--S-EE----------------------- 327

WP_042189402_1 N--------RKQPVLLKKIS-----GW-------F-TG-----STR-SEA-------EAD------------S-D-DDH---GPI---VTS-------ASH-G--PIISG------------LSV-D-----------SR--V---T-PLEDI--------QH-DF-DDADMDM-EP----------------------- 326

WP_068650580_1 K--------RNPPIFFQLFG-----GK-------S-AK-----SEN-SYAEE-DDDSDDI------------S---EII---EMV---EPV-------QRK-V--KRGRV------------EKP------LPADD--SS--------APTDT--------FV-GE-DDLS--Q-HK----------------------- 328

WP_042138096_1 R--------RAQPNLLGRVA-----GW-------F-SG-----SAR-NQQ-Q-P-GTEEY------------D-DGELP---DVI---LTE-------PRN-G--PIISG------------LAA-G-----------RG--M---P-PEDFG--------ED-EF-PEED--L-EP----------------------- 329

WP_068611148_1 P--------KKRPLFFQLLQ-----KE-------P-KT-----GQA-DDA-----EDDDD------------G---DEL-I-RRP---LPG-------NIS-A--KSKRA------------VSS-R----TFED---DD--L---E-EESET--HIPVP-AV-TL-QTAE--A-QP----------------------- 333

WP_040948739_1 ---------AKKPAASSFFN------------R-K-KQ-----RGP-IVD-----VAPEP------------L---PPL---EVY---AAV-------DTT----SAI-----------------------------------------------P--------LV-TAPE----GD----------------------- 307

WP_016312388_1 S--------SKKPVFFQLLN--FK-GR---------SD-----EPV-EAA-----PIKDE------------E---PAV---AES---AEQ-------SVDAG--INWNE------------VNY------IG---R-DE--------DESRK--------SD-GF-AGSD----------------------------- 324

WP_062326102_1 K--------RKAPIFFQLFGKWGSDRE-------Q-AK-----AER-ELD-----QENDA------------L---HSE---QVV---YRA-------DQD-KLADSWHD--------------E-------------PI--------VASDA--------TA-PA-AIPV--Q-SR----------------------- 329

WP_038571908_1 N--------RKQPVLLKKIS-----GW-------F-SG-----STR-SQV-------EAD------------PED-DDQ---GPI---VTS-------ASS-G--PIISG------------LTP-D-----------SR--V---I-PLEDI--------DH-DF-DDADLDI-EP----------------------- 327

WP_042128826_1 N--------RKQPVLLKKIS-----GW-------F-SR-----STH-PEV-------DVD------------P-E-DDQ---GPI---VTS-------ASQ-G--PIISG------------LSA-N-----------SR--V---T-PLEDI--------HH-DF-DDADMDM-EP----------------------- 326

WP_038593363_1 R--------RSQPLFFRRIG-----GW-------L-SG-----TTR-TEG-Q---GEEER------------E-E-DLS---TDL---LNG-------SPN-S--PIISG------------LDA-G-----------RG--R---A-SLDNL--------ED-DF-AGED--L-EP----------------------- 324

WP_055108403_1 P--------QRKPVFFQLFN--FK-PR-------P-KQ-----DDI-AEE-----SREEL------------H---GEF---ADQ---ESL-------HTS-E--VNWNQ------------LEY------AG---S-TP--------EAARFDPYTGEPLVQ-TD-EQKD----------------------------- 329

WP_042214780_1 R--------RAQPNLLGRVA-----GW-------F-SG-----SAR-NEQ-Q-S-GTEEH------------G-DGELP---DVI---LTE-------PRN-G--PIISG------------LAA-G-----------RG--M---P-PEDLG--------ED-DF-PEED--V-EP----------------------- 329

WP_042237084_1 R--------RAQPNLLGRVA-----GW-------F-SG-----SAR-NQQ-Q-P-GTEEY------------D-DGEMP---DVI---LTE-------PRN-G--PIISG------------LAA-G-----------RG--M---P-PEDFC--------ED-EF-PEED--L-EP----------------------- 329

WP_076168009_1 K--------RSAPVFFQLFG-----SR-------F-AK-----NEA-QPP-----LADDE------------L---DGS---DIL---EPV-------RD-----TRPAA------------GGR------FSEA---VE--------PEEND--------AF-DL-HEEP--L-WA----------------------- 322

WP_044877535_1 K--------RSTPLFFQLFG-----SK-------K-KS-----TRA-DTEGQ---EGSPV------------I---QDQ---SLI---DED-------DLE-------------------------------------AE--------EHKHP--------WI-RA-AETK--S-PE----------------------- 310

WP_006036648_1 N--------KRKSMFFSWMR-----DQQHDSNEHA-SE-SR--EWT-MDH-----DDDEEQQRKTPLVRIRSN-R-TAD---QPE---ARK-------PEG-D--TLLSRM-----------LRR-A-----------GK--P-----SADER-----------ET-DSDN--EARA-----------PFASLNDEGVGR 365

WP_044478415_1 P--------RNKPVFFQLLG--FK-GD-------RGKE-----ETV-GVE-----QPANE------------A---PPE---EEA---AAV-------S---G--INWNE------------LDY------QG----------------GSTV--------TD-PA-TASD----------------------------- 320

WP_091181726_1 P-R------RKKSLFFSWRN-----QE-------------Q--SSE-QEP-----QSAQD------------D-W-SMN---EEH---YHG----GHDNSV-K--PHWANTPDWD---DE--WDR-N-----------GE---------VGSV-----------AK-QQGD--E-LP-----------PWE-QQPERENM 337

WP_036656364_1 N--------RRQPLFFKRLA-----QW-------T-SG-----SLN-TRS-E-----TDH------------L-E-DDE---SEQ---ELS-------GQT-G--PIITG------------LTG-G-----------AAPTI---G-PLDN---------EN-EL-IDDD--L-EP----------------------- 324

WP_087916202_1 D--------RSQPTIIQRLT-----GW-------F-KG-----STP-ERA-G-A-AEDMD------------E-D-DDF---G---------------LAD-G--PLISG------------LSSIP-----------TS--S---A-PYAGM--------E-----EADEAEL-EP----------------------- 318

WP_014281139_1 A--------RKQPIFFQLFGL----KK-------S-AG-----ENK-DDR-----SVHAD------------E---DDQ---EPA---YEA-------EEA-Q--PLWRN------------AKQ-------------AE--------ELSDT--------AE-HI-EEEA--S-VP----------------------- 320

WP_039277135_1 A--------RKQPIFFQLFGL----KN-------T-DK-----ADK-EEQ-----TAHAD------------E---DEH---DLL---YDA-------GDV-Q--PQWRN------------GNQ-------------GE--------ELSDV--------IE-HA-EQEA--S-IP----------------------- 320

WP_013370740_1 A--------RKQSIFFQLFGL----KN-------T-DK-----ADK-EEQ-----PAHAD------------E---DEH---DLL---YEA-------GDV-Q--PQWRN------------GNQ-------------GE--------ELSDV--------IE-HA-EQEA--S-IP----------------------- 320

WP_023988230_1 A--------RKQPIFFQLFGL----KN-------S-DK-----ADK-EKH-----SVDTD------------E---DDH---DLP---YEA-------EDA-Q--AQWRN------------DNQ-------------SE--------ELSDV--------VE-HA-EEEA--A-TP----------------------- 320

WP_058710517_1 A--------RKQPIFFQLFGL----KN-------T-DK-----ADK-EEQ-----PAHAD------------E---DDH---DLL---YEA-------GDV-Q--PQWRN------------GNQ-------------GE--------ELSDV--------IE-HA-EQEA--S-IP----------------------- 320

WP_071640541_1 A--------RKQPIFFQLFGL----KN-------S-DK-----ADK-GEH-----SVYTD------------E---DDH---DLP---YEA-------EDA-Q--AQWRN------------GNQ-------------SE--------ELSDV--------VE-HA-EQEA--A-IP----------------------- 320

WP_053325270_1 A--------RKQPIFFQLFGL----KN-------S-DK-----ADK-EKH-----SVDTD------------E---DDH---DLP---YEA-------EDA-Q--AQWIN------------DNQ-------------SE--------ELSDV--------VE-HA-EQEA--A-IP----------------------- 320

WP_094155431_1 A--------RKQPIFFQLFGL----KK-------S-DK-----SDK-EEH-----SDHVD------------E---DNY---DLP---YEP-------EAA-E--SQWRN------------GTQ-------------EE--------ELSDV--------IE-HA-GEEA--S-TP----------------------- 318

WP_068655629_1 K--------RNAPVFFQLFG-----GK-------T-EK-----KED-SNIEV---DTDDM------------L---DDS---GVI---EFD-------KRK-N--RARKV------------VDR------AQQDNKFSK--------DSDRA--------LL-DD-ELLQ--S-EH----------------------- 328

WP_015736290_1 K--------RKAPQFFQLFG-----SK-------G-NK-----RNE-AAE-----EAEDV------------M---DES---APL---FTV-------GNG-H--GEHDE------------GEV------VLFS---RA--------DQSEE--------GT-DA-AFSA--P-DP----------------------- 323

WP_036642107_1 R--------RKAPQFFQLFS-----SK-------G-NK-----RNE-VPE-----EAVEA------------V---DDS---APV---FTV-------GNG-H--GTNEK------------GEV------VLFN---SS--------DQPEG--------VM-DA-ATPP--V-DS----------------------- 322

WP_108465295_1 A--------KAKPAFFQLLG--LK-DR-------H-RD----VSDD-EAH-----ERYQE------------D---SIS---------ENE-------QAR-V--INWND------------VSY------D--HEN-HE--------DAEPI---------M-KP-GRED----------------------------- 325

WP_045672049_1 Q--------NKKSLFFSWRS-----KE-------A---------EQ-TAQ-----PAGDD------------D-W-MME---EPF---SSG-------GAS-P--V--SPE-----------VTI-S-----------SR--Q-----SDDDV---------------D------VP-----------PWQTRADDQPDK 322

WP_036651575_1 K--------RSTPVFFQMFG-----SR-------F-AK-----QEK-DYQ------EDEE------------L---DGR---DIL---EGI-------KDY-P--SKPAK------------ASKGPYDHLLRQS---LD--------NPNKQ--------GL-DV-EREE--V-FQ----------------------- 326

WP_074094588_1 K--------RNSPIFFQLFGKWGAKRE-------Q-AT-----SGR-EMD------EVDS------------A---ETE---QIV---YRA-------EQD-HNLEAWQD--------------T-------------TE--------DVTNK--------LA-SS-RVPV--Q-AK----------------------- 329

WP_020428017_1 S--------RSQPQFLRRIG-----GW-------F-SG-----ASR-TGQ-P-A-GEAHP------------A-D-EEA---DVV---LAG-------TPS-E--PIISG------------FSA-G-----------DQ--S---Q-LDD----------DE-DY-LGDD--M-EP----------------------- 321

WP_025704675_1 S--------RNQPQFLRRIG-----GW-------F-SG-----GAR-TGQ-P-A-GEGHS------------A-D-EDA---DAV---FTG-------TPS-N--PIISG------------LPG-G-----------DL--S---Q-PDD----------DE-DY-IGDD--A-EP----------------------- 321

WP_047171161_1 R--------RSQPLFFRRIG-----GW-------L-SG-----ASN-APD-HTE-GSEQS------------L-Q-ADG---GAV---SSA-------SPV-L--PVISG------------LEA-D-----------IT--R---A-VSDDP--------SAYEP-SEEE--V-EP----------------------- 335

WP_042177473_1 Q--------RSKPLFISRIG-----NW-------I-SG-----ASK-SPP-D-E-GPDQG------------L-Q-ATG---APP---GLT-------SQA-L--PVISG------------LEA-D-----------NA--R---G-ASEHP--------SS-EN-ADED--Q-GP----------------------- 334

WP_099479291_1 R--------RKAPQFFQLFG-----GK-------G-QK-----TAQPTPD-----FSDDH------------D---EDQ---APV---FTA-------GNG-----VHDQ------------VEK------VLYR---ND--------DAAH----------L-SP-EEAE--H-SG----------------------- 318

WP_038696628_1 S--------RKAPQFFERLM-----QR-------K-PA-----RER-VES-N----PELE------------E-E-------PAL---PAE---------A-M--PIMSG------------FTA-S-----------SN--D---N-FDDFE--------DF-HP-EEED--L-EE----------------------- 318

WP_015844967_1 P-R------RKKSLFFSWRN-----QE-------------Q--NSQ-QEQ-----QNAHD------------D-W-TMN---EEH---YDG----GHDNGA-K--PHWANDQAWD---DDEQWDR-N-----------GE--G-----DASTS-----------HK-QQED--E-LP-----------PWEEQQPVRGNP 341

WP_025335307_1 N--------RKKPQFFDKLI-----KR-------K-SP-----GSG-SEA-S----MATD------------E-EHGKK---PDW---D-----------N-V--PIISG------------VNS-R-----------SD--W---SDSGEDN--------DF-DF-DSEE--L-EP----------------------- 320

WP_036622367_1 P--------RKKPVFFQLLG--YK-GR-------S-KE-----PAA-EVP-----EPFGP------------D---DEA---DDY---NES-------PVA-S--INWNE------------LDY------TG---R-GA--------DGGSV----GVPHGS-GE-TAAD----------------------------- 329

WP_042160596_1 R-K------KKRSLFFSWLQ-----QD-------------K--S----EQ-----TVKQD------------D-W-SLD---DEE---DEE----HHLNGM-S--PHWAKNGNGEVTAGE--WDR-N-----------DD--P-----AEQRP-----------IL-EQDQ--E-HTQETLHNPPDQVPWE-TVPTIPDP 351

WP_062492619_1 EVR----RPKKRAMFFQWFL-----ND-------G-GK-ER--NRS-GDE-----EEPAE------------S-G-TEE---HPL---YSD-------SGG-R--SAWMKE-----------EER-E-----------NA--L-----HADEH-----------DR-DDED--E-EP-----------PWDEEDEKG-RP 345

WP_068694663_1 A--------PKKPVFFQLLG--FK-GN-------R-QERQPLMDEA-DLD-----APFED------------N---ASPAIITGP---ASD-------TAD-S--VDWNE------------VKF------SGGFDR-TE--------PAAEL---------N-PR-QEEE----------------------------- 337

WP_025694771_1 S--------RKAPQFFDKLL-----RR-------K-TP-----RSE-GEA-L----PESG------------E-D--------DW---DEE-------HRN-I--PIISG------------IPS-Q-----------SD--W---S--GPDE--------DY-DF-ENEE--L-ES----------------------- 314

WP_042207076_1 S--------RKAPQFFDKLL-----RR-------K-TP-----RSE-GEA-L----PESE------------E-E--------DW---DAE-------HKN-V--PIISG------------IPS-Q-----------SD--W---S--GADD--------DY-DF-DNEE--Q-EP----------------------- 313

WP_068619226_1 ---------RKKPVFFQLLG--FK-EK-------N-KQ-E---ETP-AAE-----NGTSG------------D---APE-----------------------D--VDWDG------------V-Y------TS---E-NA--------GRSPL------ETET-AH-QNQE----------------------------- 313

WP_041063710_1 K-Q------RKAPLFFQLFH-----GK----------------SER-EMN-----LPPED------------N-W-EEE---DPV---YQP--GRRQ-------------------Q-GP--REH-H-----------ND--G-----AAEDA-----------SV-RNNE--Q-QS----------------------- 315

WP_035119316_1 ------------------Q--P------RFTDFAQ------QVYPTPT--WEAEAD-R--E------------E--E-P---------------------------------------------------E-MP-A-------------HLVQS-E-L-ELG-----------------------------MDDD-GHA- 390

WP_060536749_1 ------------ES----AS-PELEKPLIIRDFFE------QIRSERS--GDQPED----H-GT-ESLSSSS-E--F-N-PD-YSL------DHS------------A-ALPDS-A-------A------L-SA-DMGAASFP------NSRVA-----SGT----------DL-----------------NDQG-TAE- 376

WP_084777845_1 ------------DR----QG-P------IIRDFFE------HIRSEKS--RTAESD----Q-GS--TLTVD---------------------DDA------------D-DFVES-I-------A------L-SS-SAA-----------NPMVA-----DGV-----------I-----------------NPIE-AAS- 438

WP_085981382_1 ------------HT-------P------LFRDFTA------T--------DDSGDE------KS---------H----H-AE----------EVGGFTKPATWEEGAD-DIEDD---W------------N-ND---PQAETQ-GVQQ-GASPL---R-HQN------------------------DVVE-SEGD----- 411

WP_042233096_1 ------------QA-DR-EFST------VFRDFQE------QVQKEEK--VVPSSP------------------------------------AAE--------------GTVGP--------------A-VVAG---------------SPKAA-----ASL-----------------------------HAEK-IPA- 370

WP_013919428_1 ------------AI-------P------VIRDFQE------QVKQETA--VKPDES------------------------------------VPP--------------AASAA------------------AA---------------GPGAA-----SAA-----------------------------AEEG-AGG- 381

WP_087432898_1 ------------EA-------P------RFRDFTA------QAAESGR--ADQEDQ------LP---------E----H-VP----------AAG----QSPWKH--D-DTEDD---W------------E-EA---VPGAL---AHQ-GGADS---A-HAG-----------------------------LDPE----- 403

WP_062408022_1 ------------AI-------P------VIRDFQE------QVQQETV--LATGAK-----------------------------------------------------GEGGA------------------SS---------------GSKAA-----AQE-----------------------------SAEH-EGG- 365

WP_099520634_1 AAQPYAHSDIT-DE-QE-WS-E------PWRPQET-QEQQELVHIEGN--EAEDSE-Y--D------------A--A-EQAN-EAFGEQAVQQGD------------D-EYDEP-L--------------L-DS-E-------------RAADG-H-L-DAA-----------------------------AVPG-SNA- 443

WP_082110759_1 AAQTYAHNDLT-DE-QE-WS-E------PWRPQET-QEQQELVHIEEN--EEENTE-Y--D------------A--I-EQTN-EEF-EQALQQND------------D-EYEGP-V--------------L-DG-V-------------RAAGS-Q-L-EAA-----------------------------AVSD-SNA- 374

WP_010269406_1 ------------AI-------P------VIRDFRD------HLQQEEA--IPPK------------------------------------------------------------------------------PL---------------ETKAK-----PAE-----------------------------TTEG-AAD- 328

WP_079940749_1 ------------TE-------P------VITDFLD------HPDINTK--TASGQS------------------------------------AQS--------------AQSAQ------------------SQ---------------PISHE-----HQA-----------------------------MREE-EAG- 348

WP_089523128_1 A-----------------SG-P------IASSGSKLSSGSGPTISESS--QMDDRE-M--E------------T--F-D-------------AGN------------A-DDQEE-F---P----------V-AP-V-------------RIHPA-E-Q-SEA-----------------------------LAGP-D--- 409

WP_041854363_1 ------------------AG-T------AGREPWQ---------PEPL--RQEDPA----D------------P--V-E-PE----------ADA------------G-DGTDA-A--------------E-EA---------------PGAAA---P-ASP-----------V-----------------VRIG----- 380

WP_042189402_1 ------------VT-------P------IIRDFFE------HIRSEGL--NEEDRE----E-WS---------E--F-S-PA----------ARG------------V-TVKAP-V--------------A-GA---------------NPVSQ-Q-P-SDE-----------------------------PDEN----- 384

WP_068650580_1 ------------SP----ATVP------IIRDFFE------HVKAEGL--ADEDDL----E-EA------------F-T-G-----------REV------------A-DLKES-I--------------M-KN----------HGEI-SGEPS---L-AFG-----------------------------QASE-GNV- 393

WP_042138096_1 ------------VT-------P------IIRDFFE------HIRSEGL--NAEDRE----E-WS---------E--F-S-PA----------ARG------------G-GVTGA-G--------------D-EA---------------LPAAAGH-P-ALA-----------------------------DEAA-EDG- 391

WP_068611148_1 ------------FV-------P------IIRNFED------QLPIAEL--DDEETA--------------------F-I-SN----------ASS------------A-VTLQA-H--------------V-NS-------------------------SDN-----------------------------GQSD-GDE- 383

WP_040948739_1 ------------AI-------P------VVRDFQD------HRDAFEE--AVPFPA------------------------------------SGN--------------GMNGN------------------GH---------------SVGSG-----AKT-----------------------------KSSA-VGT- 355

WP_016312388_1 ------------------TG-P------IIRDFFD------HIQQESF--HQDEAP----D-ED--------------E-PG----------LHS------------S-LSPSV-P--------------F-EE---------------TAQDA---L-DPV------GGD----ISGDNDESGL------GNPA-AEM- 395

WP_062326102_1 ------------PN----SA-P------IIRDFFE------HVRAEDA--SKEDDL----D-DAYPFPDDLTDN--M-H-GD----------EPP------------I-KVSDE-L--------------V-DTNEWSGAG---HTGT-DVMDA---I-EGA-----------------------------MDEE-STL- 411

WP_038571908_1 ------------VT-------P------IIRDFFE------HIRSEGL--NEEDRE----E-WS---------E--F-S-PA----------ARG------------E-AAKPA-V--------------A-GA---------------NAVKQ-Q-P-TDE-----------------------------SEEN----- 385

WP_042128826_1 ------------VT-------P------IIRDFFE------HIRSEGL--NEEDRE----E-WS---------E--F-S-PA----------ARG------------V-TGKAP-V--------------T-GV---------------NAVNQ-P-P-SDE-----------------------------PEEN----- 384

WP_038593363_1 ------------VT-------P------IIRDFFE------HIRSEGL--NEEDRE----D-WS---------E--F-S-PA----------ARS------------GNSLRGS-V--------------L-EG---------------VPADT---P-AID-----------------------------GDAP-EDP- 385

WP_055108403_1 ------------------VG-P------IIRDFFD------NIQFEG---RSGAYE----N-ES--------------E-AE----------EQP------------T-GGQAG-W--------------P-IE---------------AVPEE---A-HQTLEPDGFELE----LPAGD-ADN-------DQAA-PSA- 403

WP_042214780_1 ------------VT-------P------IIRDFFE------HIRSEGL--NAEDRE----E-WS---------E--F-S-PA----------ARG------------G-AVIGA-G--------------D-EA---------------LSSAAGY-P-AAE-----------------------------GESA-EAG- 391

WP_042237084_1 ------------VT-------P------IIRDFFE------HIRSEGL--NAEDRE----E-WS---------E--F-S-PA----------ARG------------G-AVTGV-G--------------D-EA---------------LPAAAGH-P-AAA-----------------------------DKAA-EDG- 391

WP_076168009_1 ------------EPKRNESS-P------IIRDFFE------HVKSEGN--VDPEEL----D-EA------------Y-A-G-----------AAG------------A-AFPPE-G--------------I-SA----------EFHE-DPAGT---P-DGG-----------------------------VRVT-AAD- 390

WP_044877535_1 ------------IV----AT-P------IIRNFFE------QVKSEGM--IEEDDL----E-DV------------F-T-G-----------QEV------------E-DLKNV-I--------------M-NN----------PDVA-AVGHT---R-DQD-----------------------------QGDM-NKL- 374

WP_006036648_1 T-----------------DR-P------TWPNHPS-EFNEDAAFEPGA--TAGDDW-D--N------------K--Y-P-------------EQQ------------A-AAPSA-R--------------V-SP-S-------------SLSPA-F-E-DGE-----------------------------IDGG-STGF 431

WP_044478415_1 ------------------TG-P------IIRDFFE------HIRQESW--RPEEDL----DGED--------------L-PG----------PEE------------T-VGTEG-T--------------E-GT---------------EVVQE---L-QDR------EG-----INNHEAVEHLSPMEASGEDG-GLQ- 397

WP_091181726_1 A-----------PV-YP-AY-N------ELEEDEE-PAVITRIVPRAD--TYEDEA-Q--D------------D--Y------VTL------PAD------------P-ARYGE-E--------------Y-DE-I-------------DPEPA-M-A-VDL-----------------------------NSED-FTE- 408

WP_036656364_1 ------------VT-------P------IIRDFFE------HIRSEGL--SQEDRE----E-WS---------E--F-S-PT----------ARS------------G-AVAHE-P--------------E-IS---------------PEELA-A-L-NHL-----------------------------EDFG-DKN- 385

WP_087916202_1 ------------VT-------P------IIRDFFE------HIRSEGL--NEEDRE----E-WS---------E--F-S-PA----------ARS------------G-SMPAA-Q--------------P-SA---------------GQVQA-D-P-NQA-----------------------------PAADGEEGV 381

WP_014281139_1 ------------VK----RS-P------IIRDFFE------QVKHEER--RPEDES----E-TS------------L-A-GA----------ERT------------D-SAYDE-S--------------L-DD-NFMDAAV--NTDT-DGTAA---TGHNT-----------------------------PDSA-DGR- 393

WP_039277135_1 ------------VK----RS-P------IIRDFFE------QVKNEEK--RSEAEL----E-DS------------L-T-GG----------VRN------------D-SI-DE-P--------------L-DS-NFMDADANTNINI-NDSAT---I-HGT-----------------------------SEEA-QEL- 393

WP_013370740_1 ------------VK----RS-P------IIRDFFE------QVKNEEK--RSEAEL----E-DS------------L-T-GG----------VRN------------D-SIIDE-H--------------L-DS-NFMDADA--DMNR-NDSAT---I-HGT-----------------------------SEEA-QEL- 392

WP_023988230_1 ------------VK----RS-P------IIRDFFE------QVKHEER--RPEAEF----G-ES------------L-T-GA----------EQT------------T-SIIDE-S--------------L-DD-DFVDAHA--NTDM-DDSTT---I-QGT-----------------------------LEGA-QGQ- 392

WP_058710517_1 ------------VK----RS-P------IIRDFFE------QVKNEEK--RSEAEL----E-DS------------L-T-GG----------VRN------------D-SIIDE-H--------------L-DS-NFMDADA--DMNR-NDSAT---I-HGT-----------------------------SEEA-QEL- 392

WP_071640541_1 ------------VK----RS-P------IIRDFFE------QVKHEER--RPEAEF----G-ES------------L-T-GA----------EQT------------T-SIIDE-S--------------L-DD-DFVDANA--NTDM-DDSTT---I-QGT-----------------------------LEGA-QGQ- 392

WP_053325270_1 ------------VK----RS-P------IIRDFFE------QVKHEER--RPEAEF----G-ES------------L-T-GA----------EHT------------T-SIIDE-S--------------L-DD-DFVDANA--N-----DSTT---I-QGT-----------------------------LEGA-QGQ- 388

WP_094155431_1 ------------VK----RS-P------IIRDFFE------QVKHEER--RSEAE-------ES------------L-A-EV----------EHS------------D-SVYDE-P--------------L-DD-NFTDAVV--NTDM-DNASA---S-RST-----------------------------TDGT-EGQ- 388

WP_068655629_1 ------------SV----TTTP------IIRDFFE------HVKSEGI--SDDDDL----E-EA------------F-T-G-----------QEV------------A-DLKDA-I--------------M-KS----------HEQV-AVDQA---S-EQG-----------------------------QGIG-GKV- 393

WP_015736290_1 ------------VP----SS-P------IIRDFFE------QVKQEGR--LDDDQD----D-HE------------A-V-M-----------PPA------------H-EDPSG-N--------------A-AS----------SSNAMVDPET---L-NGG-----------------------------TN---SAV- 386

WP_036642107_1 ------------VP----SS-P------IIRDFFE------QVKQEER--LDDEYD----D-QE------------A-V-M-----------PPL------------A-QDEPG-G--------------K-AA----------GPNS-IDANE---L-PGG-----------------------------GDLD-ATA- 386

WP_108465295_1 ------------------SG-L------IIRDFFD------HIQNEDR--SLDDDKEAWPE-EE------------H-L-AD----------AAD------------D-VHRL-----------------E-DS---------------ELSPA---P-PDSYDPAV-EGA--------QVAEP-------GEPP-PQP- 399

WP_045672049_1 R-----------------AG-Q------ASSEGSK----AGAAVAEYE--RPATAE-N--D------------E--L-E-------------DDN------------D-DWTED-L---AE---------V-HP-E-------------GIDDG-E-L-DEL-----------------------------PAGS-ESA- 386

WP_036651575_1 ------------AP-----T-P------IIRDFFE------QVKSEG---VGEEDW----D-DP------------H-V-N-----------DSV------------G-D-ESV-Q--------------L-NA----------EADD-ELGDV---L-NDG-----------------------------SSVS-DTN- 387

WP_074094588_1 ------------PN----SA-P------IIRDFFE------HVRTEEA--SIEDDL----D-DAYPFPDNLADPNVV-Q-QG----------EHA------------I-KITDE-L--------------V-ET-EWS-AS---DAGT-NGMNA---V-DEI-----------------------------QSGE-EVP- 411

WP_020428017_1 ------------VT-------P------IIRDFFE------HIRAEGL--NAEDRE----E-WS---------E--F-S-PA----------ARS------------G-AVREA-V--------------S-GT---------------PPVNGGA-T-AEE-----------------------------AVPE-EDL- 383

WP_025704675_1 ------------VT-------P------IIRDFFE------HIRAEGL--NAEDRE----E-WS---------E--F-S-PA----------ARN------------G-AVREA-V--------------S-AI---------------PPVHGGV-P-AEE------------------------------VPE-EDL- 382

WP_047171161_1 ------------VT-------P------IIRDFFE------HIRSEGL--SEEERE----E-WG---------G--F-S-PA----------ARS------------A-GTRSP-A--------------G-SS---------------GQAAV-E-P-SGT-----------------------------SDIP-DEE- 396

WP_042177473_1 ------------VT-------P------IIRDFFE------HIRSEGL--SEEERE----E-WG---------G--F-S-PA----------ARS------------A-GTRGP-A--------------A-PD---------------SPAAA-R-P-AGA-----------------------------ADVP-DED- 395

WP_099479291_1 ------------AP----SS-P------IIRDFFE------QVKQEGS--LHEEAD----E-PD------------G-P-IG----------EPG------------M-DISPD-H--------------S-EA----------AVNA-VEAEH---S-GDG-----------------------------AAAE-ANG- 383

WP_038696628_1 ------------VT-------P------IIRDFFQ------HIRSEGL--SEEERE----E-WG---------E--F-S-PA----------ARS------------S-MRTGDLE--------------L-DT---------------GQTPNDFGD-PEL-----------------------------SQAE-PDA- 382

WP_015844967_1 S-----------SA-SP-VH-N------EYEEDEE-PAVITRIVPRAD--TYDDEE-Q--E------------D--Y-PAPAPAAF------AAD------------S-ARYTE-E--------------Y-DQ-N-------------ETESS-V-V-NEV-----------------------------HGSQ-DIQ- 417

WP_025335307_1 ------------AT-------P------IIRDFFE------HIRSEGL--SDEDRE----E-WG---------E--F-S-PA----------ARS------------S-MKPAP-------------------------------------------A-SGI-----------------------------TSET-EDG- 371

WP_036622367_1 ------------------TG-P------IIRDFFD------HIQREGLGDREDEDE----N-ES-----------PLYT-AA----------DDA------------D-AAYGG-R--------------G-ES---------------GVADD---L-RGA--ADG-EPD----MPPGSGASG-------EEGA-DGA- 407

WP_042160596_1 AVAQHQEAALDAPS-YP-PA-P------PYAEEEK-DEEWTGTWPQQS--TEDEQQ-M--E------------E------PS-VDF------PED------------E-----------------------------------------QLPAA-V-A-CEA-----------------------------ADTA-DTA- 424

WP_062492619_1 T-----------------TA-P------DWER-------EWPPVESAT--ESAERF-A--D---------------M-P-------------EGA------------G-KSPS--------------------P-A-------------ELSEA-L-V-DAD-----------------------------GGGG-TEG- 399

WP_068694663_1 ------------------RG-L------IIRDFFD------HIRKENG--DYEDDH----E-ET------------F-Y-SS----------VAE------------H-GNRVG-QDYGAGDESGYEGPQA-NS---------------PVNQE---Q-EEVFGQHG-EGASGMDLEEEDTLNP-------GSAA-IKE- 431

WP_025694771_1 ------------AA-------P------IIRDFFE------HIRAEGL--SDEDRE----E-WS---------E--F-S-PA----------ARG------------S-AKPAD-------------------------------------------A-LSL-----------------------------AEAA-EGS- 365

WP_042207076_1 ------------VT-------P------IIRDFFE------HIREEGL--SDEDRE----E-WS---------G--F-S-PA----------ARS------------S-TKPAD-------------------------------------------A-LSL-----------------------------TEAA-EGA- 364

WP_068619226_1 ------------------PS-L------IIRDFFD------QIKREDV--REEDKD------DQ--------------T-AD----------MDD------------R-EDSHS-Y-------TPFD---P-DA---------------GGSPS---A-HAE------EGP-----------------E--NGDL-PGA- 378

WP_041063710_1 ------------------AG-P------RFTDFTS------QSYAEPP--WEADAH-E--E------------P--I-A---------------------------------------------------S-DP-A-------------AVHNE--------------------------------------SDK-SEG- 358

WP_035119316_1 -----------EVA-----------DQPQAD-DVADIAAT----------PE--PPK--PARKPP-KPYRLPGLNLLDKPTATGKSG-NAAD-YMSTARKLEATLESFGVRAKVLDVARGPAVTRYELQPDVGVKVSRIVNLTDDIALALAAKDIRMEAPIPGKSAIGIEVPNNEVSVVTLREVLETQPFQESQAKLTIA 550

WP_060536749_1 -----------GLM-A---------EAKEQT-ENGT--VENRDSEAQ---EV--IPV--PAPPPP-KPYKLPPFQLLAMPKNIASAS-DQND-YMVIARKLETTLESFGVRAKVLEVVRGPAVTRYEIQPDVGVKVSRIVGLTDDIALALAAKDIRMEAPIPGKSAIGIEVPNSEVSMVTMREVMETQIFKESEAKLSIA 542

WP_084777845_1 -----------SVG-A---------EAVLPE-GDPS--IP---ATAG---EL--AEA--PIKPPP-KPYKLPPLKLLALPKEDENASANQAD-YMQTAHKLETTLESFGVRARVLEVVRGPAVTRYEIQPDVGVKVSRVVGLTDDIALALAAKDIRMEAPIPGKSAIGIEVPNNEVSMVTMREVMDTSVFADAPSKLTVA 602

WP_085981382_1 V----------QLE-T---------DGLLSD-GEGT--------------NE--AVV--IQKPPP-KPYKMPPFNLLAKQQGSGKGG-DQSD-FMQTARKLEATLESFGVRAKVLEVVRGPAVTRYEIQPDIGVKVSRIVSLSDDIALALAAKDIRMEAPIPGKSAIGIEVPNNEVSVVTMREVMETPTFIDAPSRLSIT 569

WP_042233096_1 -----------DEQ-----------LETLDD----------LQLVDT------------AVPPVL-PPYDLPSLTLLSKP-AAGKGS-EMAD-YKANARKLEATLESFGVRAKVLEVVRGPAVTRYEIQPDVGVKVSRIVSLTDDIALALAAKDIRMEAPIPGKSAIGIEVPNSEVSVVTMREVMETSAFQEASSRLSVT 522

WP_013919428_1 -----------GEE-----------FDIKNK-------------------------------PLA-IPYELPPLNLLSKP-ASGKGA-ESLD-YKAVARKLEATLESFGVRAKVLEVVRGPAVTRYEIQPDVGVKVSRIVSLTDDIALALAAKDIRMEAPIPGKSAIGIEVPNSEVSVVTMREVMESGAFQDAASKLSIT 524

WP_087432898_1 V----------VQA-P---------DG--AE-GIAA--------------EP--ALI--KPPPPPVKPYKMPSFNLLSKQPNSAKGG-DQAD-FMQTARKLEATLESFGVRAKVLEVVRGPAVTRYEIQPDIGVKVSRIVSLSDDIALALAAKDIRMEAPIPGKSAIGIEVPNNEVSTVTMREVMETPTFLEAASRLSIA 560

WP_062408022_1 -----------TED-----------FDMKNK-------------------------------PLE-IPYELPSLQLLSKP-SSGKGG-DALD-YKAVARKLEATLESFGVRAKVLEVVRGPAVTRYEIQPDVGVKVSRIVSLTDDIALALAAKDIRMEAPIPGKSAIGIEVPNTEVSVVTMREVMESPTFQDAASKLSIT 508

WP_099520634_1 -----------ASN-----------EANQQA-AESK--------------PA--LAA--SNVPVV-KPYVLPPFTLLSKPSHMVRGG-VGSS-SMEAKLKLERTLESFGVKAKVLDPVIGPAVTRFEVEPASGVKVSKIVSLTDDIALALAAKDIRMEAPIPGRSAIGIEVPNMEISIVTMREVMETKEFYDSPSKLSIA 599

WP_082110759_1 -----------GSN-----------EANSQA-AESK--------------PA--ISA--SSVPVV-KPYVLPPFTLLSKPSHMVRGG-IGSS-SMEAKLKLERTLESFGVKAKVLDPVIGPAVTRFEVEPASGVKVSKIVSLTDDIALALAAKDIRMEAPIPGRSAIGIEVPNMEVSIVTMREVMETKEFYDSPSKLSIA 530

WP_010269406_1 -----------QEE-----------LAFSNA----------------------------SEEARE-KPYQLPDFGLLSKPIGVGRGS-NHLD-NKANAKKLEMTLESFGVRAKVLDVVIGPAVTRYEIQPDTGVKVSRIVGLTDDIALALAAKDIRMEAPIPGKSAIGIEVPNTEVSMVTLREVMESTAFYDSDAKLSIS 475

WP_079940749_1 -----------ALE-----------ADIPA--------------------------------SQV-KPYVLPPFSLLSQP-ASNKNG-EGTD-HKANARKLEATMESFGVRAKVLAVVRGPAVTRYEIQPDIGVKVSRVVGLTDDIALALAAKDIRMEAPIPGKSAIGIEVPNSEVSVVTMREVMETSAFQSSASKLSIT 490

WP_089523128_1 --------------------------------------GD----------SE--ASH--PPVKSA-KPYRLPSLSLLAKPVVSSRSG-DLIDINNDSRRKLEATLESFGVRAKVLDVVPGPAVTRYEVQPATGVKVSRIVSLTDDIALALAAKDIRMEAPIPGKSAIGIEVPNAEVAIVTMREVMETPTFASASSKLSIA 555

WP_041854363_1 V----------KRE-N---------SEETGD-TEDA------------------DAL--PPKPS--RPYLLPPLSLLSRPQPGKNSA-ADHG---EARRKLEATLESFGVKATVLDVVRGPAVTRYEVQPAAGVKVSRIVSLTDDIALALAAKDIRMEAPIPGKSAIGIEVPNTEVATVTLREVMESPVFQNAASKLSVA 533

WP_042189402_1 V----------TID-L---------DGLLSA-TPDG--------------QII-PAP--PAPPPP-KPYKLPSFRLLAKPNNSGKAG-DQND-YMQTARKLEATLESFGVRAKVLEVVRGPAVTRYEIQPDIGVKVSRIVNLTDDIALALAAKDIRMEAPIPGKSAIGIEVPNSEVSIVTMREVMETQIFQEAESRLSIA 543

WP_068650580_1 I----------GQA-N---------AEGMNE-NGEE--------------VS--LEII-APVPPP-KPYVLPPFRLLGKPSNAGKAG-DQND-YMQTARKLEATLESFGVRAKVIEVVRGPAVTRYEIQPDIGVKVSRIVNLTDDIALALAAKDIRMEAPIPGKSAIGIEVPNNEVSIVTMREVMETALFQEAESKLSVA 552

WP_042138096_1 L----------PVE-L---------EGLLGT-AENG--------------EVV-PAV--PPPPPP-KPYKLPSFRLLAKPNNGAKAG-DQND-YMQTARKLEATLESFGVRAKVLEVVRGPAVTRYEIQPDIGVKVSRIVNLTDDIALALAAKDIRMEAPIPGKSAIGIEVPNSEVSIVTMREVMETQIFQEAESRLSIA 550

WP_068611148_1 I----------SDE-M---------NGMFV-----------------------------PEPPVK-KPYKLPPFHLLAKGSAFGKGT-DQMD-YMSTARKLEATLESFGVRAKVLEVVKGPTVTRYEIQPDTGVKVSRIVSLTDDIALALAAKDIRMEAPIPGKAAIGIEVPNSEVSIVTMREVMESAAFQDASSKMSIT 531

WP_040948739_1 -----------DPS-----------GDNAPD----------VELSLN------------PEKVPP-KPYIIPSFQLLNRPVSPGKGG-GADD-YKANARKLEATLESFGVRAKVLEVVRGPAVTRYEIQPDTGVKVSRIVSLTDDIALALAAKDIRMEAPIPGKAAIGIEVPNSEVSVVTLREVMESTPFQESQAKLTIV 508

WP_016312388_1 L----------EGA-D-EAEG----DGQAAA-----------------------PPP--APKPAP-KPYKLPSFRLLSKPAGAGKSG-DQAD-YMQTARKLEATLESFGVRARVLEVVRGPAVTRYEIQPDIGVKVSRIVNLTDDIALALAAKDIRMEAPIPGKSAIGIEVPNNEVSLVTMREVMETPTFMEAESKLSIA 551

WP_062326102_1 S----------NAAKE---------AGVPDA-EGQE--------------IQ--PVK--PPPPPP-KPYKLPSFRLLSKPNNGGKGG-DQKD-YMQTARKLEATLESFGVRAKVLEVVRGPAVTRYEIQPDIGVKVSRIVSLTDDIALALAAKDIRMEAPIPGKSAIGIEVPNGEVSVVTMREVMETATFQDAESKVTIA 570

WP_038571908_1 V----------SID-L---------EGLLGA-SPDG--------------EII-PAP--PAPPPP-KPYKLPSFRLLAKPNNGGKAG-DQND-YMQTARKLEATLESFGVRAKVLEVVRGPAVTRYEIQPDIGVKVSRIVNLTDDIALALAAKDIRMEAPIPGKSAIGIEVPNSEVSIVTMREVMETQIFQEAESRLSIA 544

WP_042128826_1 V----------NID-L---------DGLLTA-TPEG--------------EII-PAP--PAPPPP-KPYKLPSFRLLAKPNNSGKAG-DQND-YMQTARKLEATLESFGVRAKVLEVVRGPAVTRYEIQPDIGVKVSRIVNLTDDIALALAAKDIRMEAPIPGKSAIGIEVPNSEVSVVTMREVMETQIFQEAESRLSIA 543

WP_038593363_1 V----------VVE-L---------DGLLGA-GEDG--------------VPV-PAA--PLPPPP-KPYKLPSFRLLAKPNNGGKAG-DQND-YMQTARKLEATLESFGVRAKVLEVVRGPAVTRYEIQPDIGVKVSRIVNLTDDIALALAAKDIRMEAPIPGKSAIGIEVPNSEVSVVTMREVMETQVFQDAESRLSIA 544

WP_055108403_1 G----------EGI-A-E-------KNAPQE-----------------------EPP--PPKPAP-KPYKLPPFRLLSKPAGSGKSG-DQAD-YKQTARKLEATLESFGVRARVLEVVRGPAVTRYEIQPDIGVKVSRIVNLTDDIALALAAKDIRMEAPIPGKSAIGIEVPNNEVSLVTMREVMETPVFQDAESKLSIA 556

WP_042214780_1 L----------PVE-L---------DGLLET-AENG--------------EVI-PAI--PPPPPP-KPYKLPSFRLLAKPNNGGKAG-DQND-YMQTARKLEATLESFGVRAKVLEVVRGPAVTRYEIQPDIGVKVSRIVNLTDDIALALAAKDIRMEAPIPGKSAIGIEVPNSEVSIVTMREVMETQIFQEAESRLSIA 550

WP_042237084_1 L----------PVE-L---------EGLLGT-AENG--------------EVV-PAI--PPPPPP-KPYKLPSFRLLAKPNNGAKAG-DQND-YMQTARKLEATLESFGVRAKVLEVVRGPAVTRYEIQPDIGVKVSRIVNLTDDIALALAAKDIRMEAPIPGKSAIGIEVPNSEVSIVTMREVMETQIFQEAESRLSIA 550

WP_076168009_1 L----------QGE-S---------AGSPAE-PAEA--------------LE--LAMT-PPAPPP-KPYKLPPFRLLSKPQNGGKAG-DQND-YKQTARKLEATMESFGVRAKVLEVVRGPAVTRYEIQPDIGVKVSRIVGLTDDIALALAAKDIRMEAPIPGKSAIGIEVPNNEVSLVTMREVMETPVFQDASSKLSIA 549

WP_044877535_1 D----------DVS-T---------SEVLTS-DVDI--------------MP--EEPI-VQAPPP-KPYILPPFRLLAKQQGNGNSG-DQQD-YMQTARKLEATLESFGVRAKVLEVVRGPAVTRYEIQPDIGVKVSRIVSLTDDIALALAAKDIRMEAPIPGKSAIGIEVPNNEVSIVTMREVMETSIFQDAESKLSVA 533

WP_006036648_1 DEYDASADERYPEG-----------NAEYDA-DEPLEGGALVETSPNEHPAD--AVM--AAAPKP-RIYRLPSLNLLLKPTGGVKGS-DGVD-KLDSQRTLEATLESFGVRAKVLDVVQGPAVTRYEVQPATGVKVSRIVGLQDDIALALAAKDIRMEAPIPGKSAIGIEVPNSEVSVVTMREVMESSAFQNSNSKLSIA 612

WP_044478415_1 L----------EGI-E-GNPE----NVGVEA-----------------------PPA--PPKPAP-KPYKLPSFRLLSRPSGAGKSG-DQAD-YMQTARKLEATLESFGVRARVLEVVRGPAVTRYEIQPDIGVKVSRIVNLTDDIALALAAKDIRMEAPIPGKSAIGIEVPNSEVSVVTMREVMETSTFTEAESKLSIA 553

WP_091181726_1 -----------ENT-----------KEKAEA-ATTP--------------VH--QPA--SDVPVK-KPYLLPPFSLLAKPSLMARGG-DGAD-AMDSKRKLEATLESFGVKAKVLDVVRGPAVTRYEVQPASGVKVSRIVSLTDDIALALAAKDIRMEAPIPGKSAIGIEVPNMEVSMVTMREVMETTTFHNAPSKLSIA 564

WP_036656364_1 -------------V-I---------DPLGSS-AVEE--------------ELV-ISP--PLPPPP-KPYKLPPFRLLTKPSNAGKAG-DQND-YMQTARKLEATLESFGVRAKVLEVVRGPAVTRYEIQPDIGVKVSRIVNLTDDIALALAAKDIRMEAPIPGKSAIGIEVPNPEVSIVTMREVMETQTFQEAESKLTIA 541

WP_087916202_1 A----------PLE-L---------AGLLDE-LGEG--------------QVV-VPP--P--PPP-KPYKLPSFRLLAKPNNGSKAG-DQND-YMQTARKLEATLESFGVRAKVLEVVRGPAVTRYEIQPDIGVKVSRIVNLTDDIALALAAKDIRMEAPIPGKSAIGIEVPNSEVSVVTMREVMETQIFQEAESKLTIA 538

WP_014281139_1 A----------QAN-A---------TGTSGE-ETEG--------------TT--VAI--PAPPPP-KPYKLPSFRLLAKPQNVGKGL-GQKD-YMQTARKLEATLESFGVRAKVLEVVRGPAVTRYEIQPDIGVKVSRIVSLTDDIALALAAKDIRMEAPIPGKSAIGIEVPNNEVSIVTMREVMETTVFQESVSNLSIA 551

WP_039277135_1 A----------QTI-A---------NGLTGE-EAEG--------------NN--VTI--PAPPPP-KPYKLPSFRLLAKPQNAGKGM-GQKD-YMQTARKLEATLESFGVRAKVLEVVRGPAVTRYEIQPDIGVKVSRIVSLTDDIALALAAKDIRMEAPIPGKSAIGIEVPNNEVSIVTMREVMETTVFQESVSNLSIA 551

WP_013370740_1 A----------QMI-A---------NELTGE-EAEG--------------TN--VAI--PAPPPP-KPYKLPSFHLLAKPQNAGKGM-GQKD-YMQTARKLEATLESFGVRAKVLEVVRGPAVTRYEIQPDIGVKVSRIVSLTDDIALALAAKDIRMEAPIPGKSAIGIEVPNNEVSIVTMREVMETTVFQESVSNLSIA 550

WP_023988230_1 A----------QTI-A---------TGVAGE-EAEG--------------TH--TAI--PAPPPP-KPYKLPSFRLLAKPQNVGKGM-GQKD-YMQTARKLEATLESFGVRAKVLEVVRGPAVTRYEIQPDIGVKVSRIVSLTDDIALALAAKDIRMEAPIPGKSAIGIEVPNNEVSIVTMREVMETTVFQESTSNLSIA 550

WP_058710517_1 A----------QTI-A---------NGVTGE-EAEG--------------TN--VAI--PAPPPP-KPYKLPSFRLLAKPQNAGKGM-GQKD-YMQTARKLEATLESFGVRAKVLEVVRGPAVTRYEIQPDIGVKVSRIVSLTDDIALALAAKDIRMEAPIPGKSAIGIEVPNNEVSIVTMREVMETTVFQESVSNLSIA 550

WP_071640541_1 A----------QTI-A---------TGVAGE-EAEG--------------TH--TAI--PVPPPP-KPYKLPSFRLLAKPQNTGKGM-GQKD-YMQTARKLEATLESFGVRAKVLEVVRGPAVTRYEIQPDIGVKVSRIVSLTDDIALALAAKDIRMEAPIPGKSAIGIEVPNNEVSIVTMREVMETTVFQESTSNLSIA 550

WP_053325270_1 A----------QTI-A---------TGVAGE-EAEG--------------TH--TAI--PAPPPP-KPYKLPSFRLLAKPQNVGKGM-GQKD-YMQTARKLEATLESFGVRAKVLEVVRGPAVTRYEIQPDIGVKVSRIVSLTDDIALALAAKDIRMEAPIPGKSAIGIEVPNNEVSIVTMREVMETTVFQESTSNLSIA 546

WP_094155431_1 A----------QTT-A---------TGFTGV-GTEG--------------AD--AAM--PAPPPP-KPYKLPSFRLLAKPQNAGKGM-GQKD-YMQTARKLEATLESFGVRAKVLEVVRGPAVTRYEIQPDIGVKVSRIVSLTDDIALALAAKDIRMEAPIPGKSAIGIEVPNNEVSIVTMREVMETTVFQESASNLSIA 546

WP_068655629_1 P----------VQS-G---------DDATSE---ED--------------VE--LTLV-NIAPPP-KPYILPPFRLLGKPNNGGKAG-DQND-YMQTARKLEATLESFGVRAKVLEVVRGPAVTRYEIQPDIGVKVSRIVSLTDDIALALAAKDIRMEAPIPGKSAIGIEVPNNEVSIVTMREVMETTIFQEAESKLSVA 550

WP_015736290_1 M----------ADT-G---------GQEGSA-PAA------------------------PPAPPP-KPYKLPPFRLLSKPNNGGKGG-DQND-YMQTARKLEATLESFGVRAKVLEVVRGPSVTRYEIQPDIGVKVSRIVNLTDDIALALAAKDIRMEAPIPGKSAIGIEVPNNEVSLVTMREVMETPTFQDAESKLSIA 538

WP_036642107_1 V----------AGD-G---------VQAGKA-PSA------------------------PPAPPP-KPYKLPPFRLLSKPNNGGKGG-DQND-YMQTARKLEATLESFGVRAKVLEVVRGPSVTRYEIQPDIGVKVSRIVNLTDDIALALAAKDIRMEAPIPGKSAIGIEVPNNEVSLVTMREVMETPTFQEAESKLSIA 538

WP_108465295_1 A----------VSE-G-------------EQ-----------------------PPV--PPKPAP-KPYKLPPFRLLSKPTNGGKAG-DQAD-YMSTARKLEATLESFGVRARVLEVVRGPAVTRYEIQPDIGVKVSRIVNLTDDIALALAAKDIRMEAPIPGKSAIGIEVPNNEVSVVTMREVMETPTFQEAEAKLTIA 547

WP_045672049_1 -----------AFD-----------SPALEG-AAPAAGSG----------SA--AAA--PQIPEA-KPYRLPPLSLLAKPS-GSRGG-DSGD-SSESRRKLEATLESFGVRAKVLDVVRGPAVTRYEVQPATGVKVSRIVGLTDDIALALAAKDIRMEAPIPGKSAIGIEVPNSEVSVVTMREVMETPTFTSAPSKLSIA 545

WP_036651575_1 T----------SES-A---------QGESLD-GGEK--------------PS--I-----PVPPP-KPYKIPPFRLLAKPQTGGKAG-DQQD-YMQTARKLEATMESFGVRAKVLEVVRGPAVTRYEIQPDIGVKVSRIVGLTDDIALALAAKDIRMEAPIPGKSAIGIEVPNNEVSLVTMREVMETPIFQDAQSKLSIA 542

WP_074094588_1 N----------DVMQG---------TDTPTP-EGQD--------------TQ--PVK--PPPPPP-KPYKLPSFRLLAKPNNGGKAG-DQKD-YMQTARKLEATLESFGVRAKVLEVVRGPAVTRYEIQPDIGVKVSRIVSLTDDIALALAAKDIRMEAPIPGKSAIGIEVPNGEVSVVTMREVMETATFQDAESKVTIA 570

WP_020428017_1 T----------RLE-L---------DGLLDS-AEGG--------------EPV-P-V--PLPPPP-KPYKLPPFRLLAKPNSGGKSG-DQND-YMQTARKLEATLESFGVRAKVLEVVRGPAVTRYEIQPDIGVKVSRIVNLTDDIALALAAKDIRMEAPIPGKSAIGIEVPNPEVSVVTMREVMETQIFQDAESRLSIA 541

WP_025704675_1 A----------PLE-L---------NGLLDS-AEGG--------------EPV-P-V--PLPPPP-KPYKLPPFRLLAKPNNGAKAG-DQND-YMQTARKLEATLESFGVRAKVLEVVRGPAVTRYEIQPDIGVKVSRIVNLTDDIALALAAKDIRMEAPIPGKSAIGIEVPNPEVSVVTMREVMETQIFQDAESRLSIA 540

WP_047171161_1 V----------TVE-L---------DGLLGT-AEGG--------------EPV-PAP--PLLPPP-KPYKLPPFRLLSKPNGGGKAG-DQND-YMQTARKLEATLESFGVRAKVLEVVRGPAVTRYEIQPDIGVKVSRIVNLTDDIALALAAKDIRMEAPIPGKSAIGIEVPNPEVSVVTMREVMETQIFQDAESRLSIA 555

WP_042177473_1 V----------TVE-L---------DGLLGT-AEGG--------------EPA-PAP--PLPPPP-KPYKLPPFRLLSKPNGSGKAG-DQND-YMQTARKLEATLESFGVRAKVLEVVRGPAVTRYEIQPDIGVKVSRIVNLTDDIALALAAKDIRMEAPIPGKSAIGIEVPNPEVSVVTMREVMETQIFQDAESRLSIA 554

WP_099479291_1 Q----------DGT-G---------NGIGQSIPAA------------------------PPAPPP-KPYKLPPFRLLSKPKNGGKGG-DQSD-YMQTARKLEATLESFGVRAKVLEVVRGPSVTRYEIQPDIGVKVSRIVNLTDDIALALAAKDIRMEAPIPGKSAIGIEVPNNEVSIVTMREVMETPIFQEAESKLSIA 536

WP_038696628_1 L----------AAE-V-GSVTDAVSTGSAEP-ISGA------------------EEP--KPVPKP-KPYKLPHFRLLSKPNNGGKGS-DQND-YMQTARKLEATLESFGVRAKVLEVVRGPAVTRYEIQPDIGVKVSRIVNLTDDIALALAAKDIRMEAPIPGKSAIGIEVPNPEVSLVTMREVMETPIFQEAESKLTIA 546

WP_015844967_1 -----------EVT-----------EETDKS-ATTG--------------IH--QPA--SDAPVK-KPYLLPPFSLLAKPSLMARGG-DSAD-AMDSKRKLEATLESFGVKAKVLDVVRGPAVTRYEVQPASGVKVSRIVSLTDDIALALAAKDIRMEAPIPGKSAIGIEVPNMEVSMVTMREVMETATFQNAPSKLSIA 573

WP_025335307_1 V----------PAE-NTGIAPTEAASGGLNE-TGEE--------------LPVVEAP--KPVPPP-KPYKLPPFRLLSKPNNGGKAG-DQND-YMQTARKLEATLESFGVRAKVLEVVRGPAVTRYEIQPDIGVKVSRIVNLTDDIALALAAKDIRMEAPIPGKSAIGIEVPNPEVSLVTMREVMETQIFQEADARLTIA 540

WP_036622367_1 A----------AGV-P-NDGS----EAAAEV-----------------------PAA--PPKPAP-KPYKLPPFRLLAKPAGAGKSG-DQAD-YMQTARKLEATLESFGVRARVLEVVRGPAVTRYEIQPDIGVKVSRIVNLTDDIALALAAKDIRMEAPIPGKSAIGIEVPNNEVSLVTMREVMETPTFMEAESKLSIA 563

WP_042160596_1 -----------AAA-----------AVLVKE-AEPA--------------SS--SSV--PAVPQP-KPYMLPPFSLLSKPSLLARGG-DGAD-SIDAKRKLEATLESFGVRAKVLDVVRGPAVTRYEVQPATGVKVSRIVSLTDDIALALAAKDIRMEAPIPGKSAIGIEVPNMEVSVVTMREVMETPAFHNAPSKLSIA 580

WP_062492619_1 --------------------------------------------------------A--PAAPVPAKPYRLPPISLLNRPTGSGRGN-DAAD-TAESRRKLEATLESFGVRAKVLDVVRGPAVTRYEVQPATGVKVSRIVGLTDDIALALAAKDIRMEAPIPGKSAIGIEVPNTEVSIVTMREVMEMPAFLNAPSRLSIA 539

WP_068694663_1 E----------GTA-G-GAGS----GEHAEQ-----------------------PAQAEPPKPKP-KPYKLPPFRLLERPSDGNKGG-DQAD-YMNTARKLEATLESFGVRARVLEVVRGPAVTRYEIQPDIGVKVSRIVNLTDDIALALAAKDIRMEAPIPGKSAIGIEVPNSEVSIVTMREVMETATFQKAESKLSIA 589

WP_025694771_1 V----------SGE-D-GVIV-EASTGKEGE-AEGD--------------IP--PAP--KPAPPP-KPYKLPPFRLLSKPSNGGKAG-DQND-YMQTARKLEATLESFGVRAKVLEVVRGPAVTRYEIQPDIGVKVSRIVNLTDDIALALAAKDIRMEAPIPGKSAIGIEVPNPEVSLVTMREVMETQIFQEAEARLTIA 530

WP_042207076_1 V----------SGE-A-GIIP-EASTGAEGE-AGGD--------------IP--PAP--KPAPPP-KPYKLPPFRLLSKPNNGGKAG-DQND-YMQTARKLEATLESFGVRAKVLEVVRGPAVTRYEIQPDIGVKVSRIVNLTDDIALALAAKDIRMEAPIPGKSAIGIEVPNPEVSLVTMREVMETQIFQEAEAKLTIA 529

WP_068619226_1 V----------QGL-T-------------PI-----------------------PTP--PPKPVP-KPYKLPSFKLLSKGVSGAKAG-DQAD-YMQTARKLEATLESFGVRAKVLEVVRGPAVTRYEIQPDIGVKVSRIVNLTDDIALALAAKDIRMEAPIPGKSAIGIEVPNSEVSVVTMREVMETPTFQDAESKMSIA 526

WP_041063710_1 -----------AVT-----------ETDPAA-N-----TQ----------PA--PAK--PARKPQ-KPYRLPSLNLLDKPAAAGKSG-NAND-YMNTARKLEATLESFGVRAKVLDVARGPAVTRYELQPDVGVKVSRIVSLTDDIALALAAKDIRMEAPIPGKSAIGIEVPNNEVSVVTLREVLETQPFKESPAKLTIA 513

WP_035119316_1 FGRDISGQPIVGNLAKMPHLLVAGATGSGKSVCINGIITSLLYKAKPDEVKFLMIDPKMVELNVYNGIPHLLAPVVTDPRRASLALKKIVVEMEKRYEKFSKSGTRNIEGYNALMLSGDNP----D-GVLPYIVVIVDELADLMMVAAGDVEDAITRLAQMARAAGIHLIIATQRPSVDVITGVIKANIPSRIAFGVSSQ 745

WP_060536749_1 FGRDIAGQTIVGNLAKMPHLLVAGATGSGKSVCINGIITSILYKAKPDEVKFLMVDPKMVELNIYNGIPHLLAPVVTDPRRASLALKKIVVEMEKRYDLFSKSGTRNMEGYNNLM--KDNP----D-AILPYIVVIVDELADLMMVAAGDVEDAIARLAQMARAAGIHLIIATQRPSVDVITGVIKANIPSRIAFGVSSQ 735

WP_084777845_1 FGRDISGQTIVGNLARMPHLLVAGATGSGKSVCINGIIASILYKAKPDEVKFLMVDPKMVELNIYNGIPHLLAPVVTDPKRASLALKKIVVEMEKRYELFSKSSTRNIEGYNTLM--KDNP----S-AVLPYIVVIVDELADLMMVAAGDVEDAIARLAQMARAAGIHLIIATQRPSVDVITGVIKANIPSRIAFGVSSQ 795

WP_085981382_1 LGRDIAGTPIVGNLARMPHLLVAGATGSGKSVCINGIITSILYKAKPDEVKFLMVDPKMVELNVYNGIPHLLAPVVTDPRRASLALKKIVVEMEKRYELFSKSGTRNIEGYNKLV--ADEP----D-KVLPYIVIIVDELADLMMVAANDVEDSICRLAQMARAAGIHLIIATQRPSVDVITGVIKANIPSRIAFGVSSQ 762

WP_042233096_1 LGRDISGQPIVGNLAKMPHLLVAGATGSGKSVCINGIITSILYKAKPNEVKFLMVDPKMVELNVYNGIPHLMAPVVTDPRRASLALKKVVVEMEKRYELFSKSGTRNIEGYNAML--IENGT---E-APLPYIVVIVDELADLMMVAANDVEDAICRLAQMARAAGIHLIIATQRPSVDVITGVIKANIPSRIAFGVSSQ 716

WP_013919428_1 FGRDISGQPIVGNLARMPHMLVAGATGSGKSVCINGIITSVLYKAKPDEVKFLMIDPKMVELNVYNGIPHLLAPVVTDPKRASLALKKVVVEMEKRYELFSKSGTRNIEGYNTML--LETQT---G-APLPYIVVIVDELADLMMVAANDVEDAICRLAQMARAAGIHLIIATQRPSVDVITGVIKANIPSRIAFGVSSQ 718

WP_087432898_1 LGRDISGTPIVGNLARMPHLLVAGATGSGKSVCINGIITSILYKAKPDEVKFLMVDPKMVELNVYNGIPHLLAPVVTDPRRASLALKKIVVEMEKRYELFSKSGTRNIEGYNKLV--AEEP----E-KVLPYIVIIVDELADLMMVAANDVEDAICRLAQMARAAGIHLIIATQRPSVDVITGVIKANIPSRIAFGVSSQ 753

WP_062408022_1 LGRDISGQPIVGNLARMPHLLVAGATGSGKSVCINGIITSILYKAKPDEVKFLMIDPKMVELNVYNGIPHLLAPVVTDPKRASLALKKVVVEMEKRYELFSKSGTRNIEGYNAML--TETQT---G-APLPYIVVIVDELADLMMVAANDVEDSICRLAQMARAAGIHLIIATQRPSVDVITGVIKANIPSRIAFGVSSQ 702

WP_099520634_1 FGRDIAGKPIVGNLAKMPHLLVAGATGSGKSVCINGIITSILYKATPDEVKFLMVDPKMVELNVYNGIPHLLAPVVTDPRRASLALKKIVVEMEKRYELFSKSGTRNIEGYNTLM--ADNP----K-AVLPYIVVIVDELADLMIVAANDVEDAIARLAQMARAAGIHLIIATQRPSVDVITGVIKANIPSRIAFGVSSQ 792

WP_082110759_1 FGRDIAGKPIVGNLAKMPHLLVAGATGSGKSVCINGIITSILYKATPDEVKFLMVDPKMVELNVYNGIPHLLAPVVTDPRRASLALKKIVVEMEKRYELFSKSGTRNIEGYNTLM--ADNP----K-AVLPYIVVIVDELADLMIVAANDVEDAIARLAQMARAAGIHLIIATQRPSVDVITGVIKANIPSRIAFGVSSQ 723

WP_010269406_1 LGRDITGQPIVANLAKMPHLLVAGATGSGKSVCINGIITSILYKAKPSEVKFLMIDPKMVELNVYNGIPHLLAPVVTDPRRASLALKKVVVEMEKRYELFSKSGTRNIEGYNSLI--ADEASDTNG-EPLPLIVVIVDELADLMMVAANDVEDAIIRLAQMARAAGIHLIIATQRPSVDVITGVIKANIPSRIAFGVSSQ 672

WP_079940749_1 LGRDISGQPIVGNLAKMPHLLVAGATGSGKSVCINGIITSILYKAKPNEVKFMMIDPKMVELNVYNGIPHLLAPVVTDPRRASLALKKIVSEMERRYELFSKSGTRNIEGYNAML--NENGT---E-APLPYYVVIVDELADLMMVAANDVEDAICRLAQMARAAGIHLIIATQRPSVDVITGVIKANIPSRIAFGVSSQ 684

WP_089523128_1 FGRDIAGQSIVGNLAKMPHLLVAGATGSGKSVCINGIITSILYKAAPDEVKFLMIDPKMVELNMYNGIPHLLAPVVTDPRRASLALKKIVVEMEKRYEQFSKSGTRNIEGYNSLM--KDNP----A-AVLPYIVVIVDELADLMMVAAKDVEDSITRLAQMARAAGIHLIIATQRPSVDVITGVIKANIPSRIAFGVSSQ 748

WP_041854363_1 LGRDISGQPIVADLARMPHLLVAGATGSGKSVCINGIITSILYKAAPDEVKFLMVDPKMVELNVYNGIPHLLAPVVTDPRRASLALKKIVVEMEKRYEAFSKTGTRNIEGYNAQT--KDNP----E-ARLPYIVVIVDELADLMMVAASDVEDAITRLAQMARAAGIHLIIATQRPSVDVITGVIKANIPSRIAFGVSSQ 726

WP_042189402_1 FGRDISGQTIIGNLAKMPHLLVAGATGSGKSVCINGIITSILYKAKPDEVKFLMVDPKMVELNVYNGIPHLLAPVVTDPKRASLALKKIVVEMEKRYELFSKSGTRNMEGYNKLM--AENP----A-AILPYIVVIVDELADLMMVAANDVEDAICRLAQMARAAGIHLIIATQRPSVDVITGLIKANIPSRIAFGVSSN 736

WP_068650580_1 FGRDISGQTIIGNLAKMPHLLVAGATGSGKSVCINGIITSILYKAKPDEVKFLMVDPKMVELNVYNGIPHLLAPVVTDPKRASLALKKIVVEMEKRYELFSKSGTRNIEGYNKLM--QENL----P-AVLPYIVVIVDELADLMMVAANDVEEAITRLAQMARAAGIHLIIATQRPSVDVITGVIKANIPSRIAFGVSSN 745

WP_042138096_1 FGRDISGQTIIGNLAKMPHLLVAGATGSGKSVCINGIITSILYKAKPNEVKFLMVDPKMVELNVYNGIPHLLAPVVTDPKRASLALKKIVVEMEKRYELFSKSGTRNMEGYNTLM--KDNP----A-AVLPYIVVIVDELADLMMVAANDVEDAICRLAQMARAAGIHLIIATQRPSVDVITGLIKANIPSRIAFGVSSN 743

WP_068611148_1 LGRDISGQPIVGNLARMPHLLVAGATGSGKSVCINGIITSILYKAKPDEVKFLMVDPKMVELNVYNGIPHLLAPVVTDPRRASLALKKIVLEMEKRYELFSKSGTRNIEGYNTML--SNKG----NIDLLPYIVVIVDELADLMMVAASDVEDAICRLAQMARAAGIHLIIATQRPSVDVITGVIKANIPSRIAFGVSSN 725

WP_040948739_1 LGRDISGQPIVGNLARMPHLLVAGATGSGKSVCINGIITSILFKAKPDEVKFLMIDPKMVELNVYNGIPHLLAPVVTDPRRASLALKKIVVEMEKRYELFSKSGTRNIEGYNAML--TDANNPNPG-TPLPLIVVIVDELADLMMVAAGDVEDSICRLAQMARAAGIHLIIATQRPSVDVITGVIKANIPSRIAFGVSSQ 705

WP_016312388_1 FGRDISGQTIVGNLAKMPHLLVAGATGSGKSVCINGIITSILFKAKPDEVKFMMVDPKMVELNVYNGIPHLLTPVVTDPRRASLALKKIVVEMEKRYELFSKSGARNIEGYNQMM--ADQP----E-AVLPYIVVIVDELADLMMVAASDVEDAIARLAQMARAAGIHLIIATQRPSVDVITGVIKANIPSRIAFGVSSQ 744

WP_062326102_1 FGRDISGQTIIGNLARMPHLLVAGATGSGKSVCINGIITSILYKAKPDEVKFLMVDPKMVELNVYNGIPHLMAPVVTDPKRASLALKKIVVEMEKRYELFSKSGTRNVEGYNNLM--KDNP----A-AVLPYIVVIVDELADLMMVAAGDVEDAIARLAQMARAAGIHLIIATQRPSVDVITGVIKANIPSRIAFGVSSQ 763

WP_038571908_1 FGRDISGQTIIGNLAKMPHLLVAGATGSGKSVCINGIITSILYKAKPDEVKFLMVDPKMVELNVYNGIPHLLAPVVTDPKRASLALKKIVVEMEKRYELFSKSGTRNMEGYNKLM--AENP----A-AILPYIVVIVDELADLMMVAANDVEDAICRLAQMARAAGIHLIIATQRPSVDVITGLIKANIPSRIAFGVSSN 737

WP_042128826_1 FGRDISGQTIIGNLAKMPHLLVAGATGSGKSVCINGIITSILYKAKPNEVKFLMVDPKMVELNVYNGIPHLLAPVVTDPKRASLALKKIVVEMEKRYELFSKSGTRNMEGYNKLM--AENP----A-AILPYIVVIVDELADLMMVAANDVEDAICRLAQMARAAGIHLIIATQRPSVDVITGLIKANIPSRIAFGVSSN 736

WP_038593363_1 FGRDISGQTIVGNLAKMPHLLVAGATGSGKSVCINGIITSILYKAKPNEVKFLMVDPKMVELNVYNGIPHLLAPVVTDPKRASLALKKIVVEMEKRYELFSKSGTRNVEGYNTLM--KDNP----A-AVLPYIVVIVDELADLMMVAANDVEDAICRLAQMARAAGIHLIIATQRPSVDVITGLIKANIPSRIAFGVSSN 737

WP_055108403_1 FGRDISGQTIVGNLAKMPHLLVAGATGSGKSVCINGIITSILYKAKPDEVKFMMVDPKMVELNVYNGIPHLLAPVVTDPKRASLALKKIVVEMEKRYELFSKSGARNVEGYNLMM--KDNP----E-AILPYIVVIVDELADLMMVAANDVEDAIARLAQMARAAGIHLIIATQRPSVDVITGVIKANIPSRIAFGVSSQ 749

WP_042214780_1 FGRDISGQTIIGNLAKMPHLLVAGATGSGKSVCINGIITSILYKAKPNEVKFLMVDPKMVELNVYNGIPHLLAPVVTDPKRASLALKKIVVEMEKRYELFSKSGTRNMEGYNTLM--KDNP----A-AVLPYIVVIVDELADLMMVAANDVEDAICRLAQMARAAGIHLIIATQRPSVDVITGLIKANIPSRIAFGVSSN 743

WP_042237084_1 FGRDISGQTIIGNLAKMPHLLVAGATGSGKSVCINGIITSILYKAKPNEVKFLMVDPKMVELNVYNGIPHLLAPVVTDPKRASLALKKIVVEMEKRYELFSKSGTRNMEGYNTLM--KDNP----A-AILPYIVVIVDELADLMMVAANDVEDAICRLAQMARAAGIHLIIATQRPSVDVITGLIKANIPSRIAFGVSSN 743

WP_076168009_1 FGRDISGQTIVGNLAKMPHLLVAGATGSGKSVCINGIITSILYKAKPDEVKFLMVDPKMVELNVYNGIPHLMAPVVTDPKRASLALKKIVVEMEKRYELFSKSGTRNIEGYNNLM--KDNP----A-AVLPYIVVIVDELADLMMVAAHDVEEAITRLAQMARAAGIHLIIATQRPSVDVITGVIKANIPSRIAFGVSSQ 742

WP_044877535_1 FGRDISGQTIIGNLAKMPHLLVAGATGSGKSVCINGIIASILYKAKPDEVKFLMVDPKMVELNVYNGIPHLLAPVVTDPKRASLALKKIVVEMEKRYEQFSKSGTRNIEGYNKLM--KDNL----P-AILPYIVVIVDELADLMMVAANDVEDAITRLAQMARAAGIHLIIATQRPSVDVITGVIKANIPSRIAFGVSSQ 726

WP_006036648_1 FGRDISGQSIVGNLAKMPHLLVAGATGSGKSVCINGIITSILYKAKPDEVKFMMIDPKMVELNMYNGIPHLLAPVVTDPRRASLALKKIVVEMEKRYELFSKSGTRNIEGYNTLM--ESNP----A-AVLPYIVVIVDELADLMMVAANDVEDAITRLAQMARAAGIHLIIATQRPSVDVITGVIKANIPSRIAFGVSSQ 805

WP_044478415_1 FGRDISGQTIVGNLAKMPHLLVAGATGSGKSVCINGIITSILYKAKPDEVKFMMVDPKMVELNVYNGIPHLLAPVVTDPRRASLALKKIVVEMEKRYELFSKSGARNIEGYNQMM--KENP----E-AVLPYIVVIVDELADLMMVAAHDVEDAIARLAQMARAAGIHLIIATQRPSVDVITGVIKANIPSRIAFGVSSQ 746

WP_091181726_1 FGRDISGQPIIGNLARMPHLLVAGATGSGKSVCINGIITSILYKAAPDEVKFLMVDPKMVELNVYNGIPHLLAPVVTDPRRAALALKKIVVEMEKRYELFSKSATRNIEGYNALM--AENP----K-AVLPYIVVIVDELADLMMVASNDVEDSIARLAQMARAAGIHLIIATQRPSVDVITGVIKANIPSRIAFGVSSQ 757

WP_036656364_1 FGRDISGQTIIGNLAKMPHLLVAGATGSGKSVCINGIIASILYKAKPNEVKFLMVDPKMVELNVYNGIPHLLAPVVTDPKRASLALKKIVVEMEKRYELFSKSGTRNVEGYNTLM--KDNP----D-AVLPYIVVIVDELADLMMVAANDVEDAICRLAQMARAAGIHLIIATQRPSVDVITGVIKANIPSRIAFGVSSM 734

WP_087916202_1 FGRDISGQTIIGNLAKMPHLLVAGATGSGKSVCINGIITSILYKAKPNEVKFLMVDPKMVELNIYNGIPHLLAPVVTDPKRASLALKKIVVEMEKRYDLFSKSGTRNLEGYNNLM--KDNP----A-AVLPYIVVVVDELADLMMVAASDVEDAICRLAQMARAAGIHLIIATQRPSVDVITGLIKANIPSRIAFGVSSN 731

WP_014281139_1 FGRDIAGQTIVGNLAKMPHLLVAGATGSGKSVCINGIITSILYKAKPDEVKFLMVDPKMVELNVYNGIPHLLAPVVTDPKRASLALKKIVVEMEKRYELFSKSGTRNIEGYNNLM--KDNP----A-AFLPYIVVIVDELADLMMVAAGDVEDAIARLAQMARAAGIHLIIATQRPSVDVITGVIKANIPSRIAFGVSSQ 744

WP_039277135_1 FGRDIAGQTIVGNLAKMPHLLVAGATGSGKSVCINGIITSILYKAKPDEVKFLMVDPKMVELNVYNGIPHLLAPVVTDPKRASLALKKIVVEMEKRYELFSKSGTRNIEGYNNLM--KDNP----D-AFLPYIVVIVDELADLMMVAAGDVEDAIARLAQMARAAGIHLIIATQRPSVDVITGVIKANIPSRIAFGVSSQ 744

WP_013370740_1 FGRDIAGQTIVGNLAKMPHLLVAGATGSGKSVCINGIITSILYKAKPDEVKFLMVDPKMVELNVYNGIPHLLAPVVTDPKRASLALKKIVVEMEKRYELFSKSGTRNIEGYNNLM--KDNP----D-AFLPYIVVIVDELADLMMVAAGDVEDAIARLAQMARAAGIHLIIATQRPSVDVITGVIKANIPSRIAFGVSSQ 743

WP_023988230_1 FGRDIAGQTIVGNLAKMPHLLVAGATGSGKSVCINGIITSILYKAKPDEVKFLMVDPKMVELNVYNGIPHLLAPVVTDPKRASLALKKIVVEMEKRYELFSKSGTRNIEGYNNLM--KDNP----D-AFLPYIVVIVDELADLMMVAAGDVEDAIARLAQMARAAGIHLIIATQRPSVDVITGVIKANIPSRIAFGVSSQ 743

WP_058710517_1 FGRDIAGQTIVGNLAKMPHLLVAGATGSGKSVCINGIITSILYKAKPDEVKFLMVDPKMVELNVYNGIPHLLAPVVTDPKRASLALKKIVVEMEKRYELFSKSGTRNIEGYNNLM--KDNP----D-AFLPYIVVIVDELADLMMVAAGDVEDAIARLAQMARAAGIHLIIATQRPSVDVITGVIKANIPSRIAFGVSSQ 743

WP_071640541_1 FGRDIAGQTIVGNLAKMPHLLVAGATGSGKSVCINGIITSILYKAKPDEVKFLMVDPKMVELNVYNGIPHLLAPVVTDPKRASLALKKIVVEMEKRYELFSKSGTRNIEGYNNLM--KDNP----D-AFLPYIVVIVDELADLMMVAAGDVEDAIARLAQMARAAGIHLIIATQRPSVDVITGVIKANIPSRIAFGVSSQ 743

WP_053325270_1 FGRDIAGQTIVGNLAKMPHLLVAGATGSGKSVCINGIITSILYKAKPDEVKFLMVDPKMVELNVYNGIPHLLAPVVTDPKRASLALKKIVVEMEKRYELFSKSGTRNIEGYNNLM--KDNP----D-AFLPYIVVIVDELADLMMVAAGDVEDAIARLAQMARAAGIHLIIATQRPSVDVITGVIKANIPSRIAFGVSSQ 739

WP_094155431_1 FGRDIAGQTIVGNLAKMPHLLVAGATGSGKSVCINGIITSILYKAKPDEVKFLMVDPKMVELNVYNGIPHLLAPVVTDPKRASLALKKIVVEMEKRYELFSKSGTRNVEGYNNLM--KDNP----A-AFLPYIVVIVDELADLMMVAAGDVEDAIARLAQMARAAGIHLIIATQRPSVDVITGVIKANIPSRIAFGVSSQ 739

WP_068655629_1 FGRDISGQTIVGNLAKMPHLLVAGATGSGKSVCINGIITSILYKAKPDEVKFLMVDPKMVELNVYNGIPHLLAPVVTNPKRASLALKKIVVEMEKRYELFSKSGTRNIEGYNKLM--QSNL----P-AVLPYIVVIVDELADLMMVAANDVEEAITRLAQMARAAGIHLIIATQRPSVDVITGVIKANIPSRIAFGVSSN 743

WP_015736290_1 FGRDISGQTIVGNLARMPHLLVAGATGSGKSVCINGIITSILYKAKPDEVKFLMVDPKMVELNVYNGIPHLMAPVVTDPKRASLALKKIVVEMEKRYELFSKSGTRNIEGYNNLM--KDNL----P-AVLPYIVVIVDELADLMMVAANDVEDAITRLAQMARAAGIHLIIATQRPSVDVITGVIKANIPSRIAFGVSSQ 731

WP_036642107_1 FGRDISGQTIVGNLARMPHLLVAGATGSGKSVCINGIITSILYKAKPDEVKFLMVDPKMVELNVYNGIPHLMAPVVTDPKRASLALKKIVVEMEKRYELFSKSGTRNIEGYNNLM--KDNL----P-AVLPYIVVIVDELADLMMVAANDVEDAITRLAQMARAAGIHLIIATQRPSVDVITGVIKANIPSRIAFGVSSQ 731

WP_108465295_1 FGRDISGQTIVGNLAKMPHLLVAGATGSGKSVCINGIITSILYKAKPDEVKFMMVDPKMVELNVYNGIPHLLAPVVTDPKRASLALKKIVVEMEKRYELFSKSGTRNVEGYNNLM--KDNP----S-AILPYIVVIVDELADLMMVAAGDVEDAIARLAQMARAAGIHLIIATQRPSVDVITGVIKANIPSRIAFGVSSQ 740

WP_045672049_1 FGRDISGQTIVGNLARMPHLLVAGATGSGKSVCINGIITSILYKAAPDEVKFMMIDPKMVELNVYNGIPHLLAPVVTDPRRASLALKKIVVEMEKRYELFSKSATRNIEGYNGLM--KDNP----A-AVLPYIVVIVDELADLMMVAANDVEEAITRLAQMARAAGIHLIIATQRPSVDVITGVIKANIPSRIAFGVSSQ 738

WP_036651575_1 FGRDISGQTIVGNLAKMPHLLVAGATGSGKSVCINGIITSILYKAKPDEVKFLMVDPKMVELNVYNGIPHLLAPVVTDPKRASLALKKIVVEMEKRYELFSKSGTRNIEGYNNLM--KDNL----P-AVLPYIVVIVDELADLMMVAANDVEEAITRLAQMARAAGIHLIIATQRPSVDVITGVIKANIPSRIAFGVSSQ 735

WP_074094588_1 FGRDISGQTIIGNLARMPHLLVAGATGSGKSVCINGIITSILYKAKPDEVKFLMVDPKMVELNVYNGIPHLLAPVVTDPKRASLALKKIVVEMEKRYELFSKSGTRNVEGYNNLM--KDNP----A-AVLPYIVVIVDELADLMMVAAGDVEDAIARLAQMARAAGIHLIIATQRPSVDVITGVIKANIPSRIAFGVSSQ 763

WP_020428017_1 FGRDISGQTIVGNLAKMPHLLVAGATGSGKSVCINGIITSILYKAKPNEVKFLMVDPKMVELNVYNGIPHLLAPVVTDPKRASLALKKIVVEMEKRYELFSKSGTRNVEGYNNLM--KDNP----A-AVLPYIVVIVDELADLMMVAANDVEDAICRLAQMARAAGIHLIIATQRPSVDVITGLIKANIPSRIAFGVSSN 734

WP_025704675_1 FGRDISGQTIVGNLAKMPHLLVAGATGSGKSVCINGIITSILYKAKPNEVKFLMVDPKMVELNVYNGIPHLLAPVVTDPKRASLALKKIVVEMEKRYELFSKSGTRNVEGYNNLM--KDNP----A-AVLPYIVVIVDELADLMMVAASDVEDAICRLAQMARAAGIHLIIATQRPSVDVITGLIKANIPSRIAFGVSSN 733

WP_047171161_1 FGRDISGQTIIGNLAKMPHLLVAGATGSGKSVCINGIITSILYKAKPDEVKFLMVDPKMVELNVYNGIPHLLAPVVTDPKRASLALKKIVVEMEKRYELFSKSGTRNVEGYNNLM--KDNP----A-AILPYIVVIVDELADLMMVAANDVEDAICRLAQMARAAGIHLIIATQRPSVDVITGLIKANIPSRIAFGVSSN 748

WP_042177473_1 FGRDISGQTIIGNLAKMPHLLVAGATGSGKSVCINGIITSILYKAKPDEVKFLMVDPKMVELNVYNGIPHLLAPVVTDPKRASLALKKIVVEMEKRYELFSKSGTRNVEGYNNLM--KDNP----A-AILPYIVVIVDELADLMMVAANDVEDAICRLAQMARAAGIHLIIATQRPSVDVITGLIKANIPSRIAFGVSSN 747

WP_099479291_1 FGRDISGQTIVGNLARMPHLLVAGATGSGKSVCINGIITSILYKAKPDEVKFLMVDPKMVELNVYNGIPHLMAPVVTDPKRASLALKKIVVEMEKRYDLFSKSGTRNIEGYNNLM--KDNP----A-AVLPYIVVIVDELADLMMVAANDVEDAITRLAQMARAAGIHLIIATQRPSVDVITGVIKANIPSRIAFGVSSQ 729

WP_038696628_1 FGRDISGQTIIGNLARMPHLLVAGATGSGKSVCINGIITSILYKAKPEEVKFLMVDPKMVELNVYNGIPHLMAPVVTDPKRASLALKKIVVEMEKRYELFSKSGTRNVEGYNQLM--KDNP----A-AVLPYIVVIVDELADLMMVAAGDVEDAICRLAQMARAAGIHLIIATQRPSVDVITGVIKANIPSRIAFGVSSQ 739

WP_015844967_1 FGRDISGQPIIGNLARMPHLLVAGATGSGKSVCINGIITSILYKAAPDEVKFLMVDPKMVELNVYNGIPHLLAPVVTDPRRAALALKKIVVEMEKRYELFSKSSTRNIEGYNALM--AENP----K-AVLPYIVVIVDELADLMMVASNDVEDSIARLAQMARAAGIHLIIATQRPSVDVITGVIKANIPSRIAFGVSSQ 766

WP_025335307_1 FGRDISGQTIIGNLAKMPHLLVAGATGSGKSVCINGIITSILYKAKPDEVKFLMVDPKMVELNVYNGIPHLMAPVVTDPKRASLALKKIVVEMEKRYELFSKSGTRNVEGYNQLM--KDNP----A-AVLPYIVVIVDELADLMMVAAGDVEDAICRLAQMARAAGIHLIIATQRPSVDVITGVIKANIPSRIAFGVSSQ 733

WP_036622367_1 FGRDISGQTIVGNLAKMPHLLVAGATGSGKSVCINGIITSILFKAKPDEVKFMMVDPKMVELNVYNGIPHLLTPVVTDPRRASLALKKIVVEMEKRYELFSKSGARNVEGYNQMM--KDNP----E-AILPYIVVIVDELADLMMVAASDVEDAIARLAQMARAAGIHLIIATQRPSVDVITGVIKANIPSRIAFGVSSQ 756

WP_042160596_1 FGRDISGQPIIGNLARMPHLLVAGATGSGKSVCINGIITSILYKAKPDEVKFLMVDPKMVELNVYNGIPHLLAPVVTDPRRASLALKKIVVEMEKRYELFSKSATRNIEGYNNLM--ADNP----K-AVLPYIVVIVDELADLMMVAAGDVEDSIARLAQMARAAGIHLIIATQRPSVDVITGVIKANIPSRIAFGVSSQ 773

WP_062492619_1 FGRDISGQPIVGNLAKMPHLLVAGATGSGKSVCINGIIASILYKAKPDEVKFMMIDPKMVELNVYNGIPHLLAPVVTDPRRASLALKKIVVEMEKRYELFSKSGTRNIEGYNTLM--ADNP----A-AVLPYIVVIVDELADLMMVAANDVEDSICRLAQMARAAGIHLIIATQRPSVDVITGVIKANIPSRIAFGVSSQ 732

WP_068694663_1 FGRDISGQTIVGNLAKMPHLLVAGATGSGKSVCINGIITSILFKAKPDEVKFMMVDPKMVELNVYNGIPHLLAPVVTDPKRASLALKKIVVEMEKRYEKFSKSGTRNIEGYNNLM--KDNP----D-AILPYIVVIVDELADLMMVAANDVEDAIARLAQMARAAGIHLIIATQRPSVDVITGVIKANIPSRIAFGVSSQ 782

WP_025694771_1 FGRDISGQTIIGNLAKMPHLLVAGATGSGKSVCINGIITSILYKAKPDEVKFLMVDPKMVELNVYNGIPHLMAPVVTDPKRASLALKKIVVEMEKRYELFSKSGTRNVEGYNNLM--KDNP----E-AVLPYIVVIVDELADLMMVAANDVEDAICRLAQMARAAGIHLIIATQRPSVDVITGVIKANIPSRIAFGVSSQ 723

WP_042207076_1 FGRDISGQTIIGNLAKMPHLLVAGATGSGKSVCINGIITSILYKAKPDEVKFLMVDPKMVELNVYNGIPHLMAPVVTDPKRASLALKKIVVEMEKRYELFSKSGTRNVEGYNHLM--KDNP----E-AVLPYIVVIVDELADLMMVAANDVEDAICRLAQMARAAGIHLIIATQRPSVDVITGVIKANIPSRIAFGVSSQ 722

WP_068619226_1 FGRDISGQTIVGNLAKMPHLLVAGATGSGKSVCINGIITSILYKAKPDEVKFLMVDPKMVELNMYNGIPHLLAPVVTDPRRASLALKKIVVEMEKRYEMFSKSGARNVEGYNNMM--KDNP----A-AVLPYIVVIVDELADLMMVAANDVEDAIARLAQMARAAGIHLIIATQRPSVDVITGVIKANIPSRIAFGVSSQ 719

WP_041063710_1 FGRDISGQPIVGNLARMPHMLVAGATGSGKSVCINGIITSLLYKAKPDEVKFLMIDPKMVELNVYNGIPHLLAPVVTDPRRASLALKKIVVEMEKRYESFSKSGTRNIEAYNALMLSGDNP----D-GVLPYIVVIVDELADLMMVAAGDVEDSITRLAQMARAAGIHLIIATQRPSVDVITGVIKANIPSRIAFGVSSQ 708

WP_035119316_1 VDSRTILDMVGAEKLLGRGDMLFLPMGASKPIRVQGAFLSDNEVEQVVGYARGQAEAEYSEDLVPEVDDTESDTPDEMLDELFDQAVQIIIEAKQASVSLLQRRMRVGYTRAARLIDTMEAKGIVGPYEGSKPREVLMTMEQY-Q--QRISS-- 894

WP_060536749_1 IDSRTILDMGGAEKLLGRGDMLFMPVGSSKPVRVQGAFMSDQEVETIVEYCRSQGEAQYNDDIVPEIDDTAA-AMDEQKDELYDQAMQIVIEAKQASVSLLQRRMRVGYTRAARLIDSLEAHGVVGPYEGSKPREVLISPEQY-HQ-INTGS-- 884

WP_084777845_1 VDSRTILDMGGAEKLLGRGDMLFMPMGASKPVRVQGAFLSDNEVEEIVAYCSGQAEAEYDTDLVPEIDDNAA-EPEEVLDDLYDQAVQIVVEAKQASVSLLQRRMRVGYTRAARLVDSMEARGIVGPYEGSKPREVLISIEQL-QA-SRAAANS 946

WP_085981382_1 VDSRTILDMAGAEKLLGRGDMLFLPMGSSKPIRVQGAFLSDQEVEAVVGFVSGQAQAEYNEELVPEIDESGH-ASDEPVDELYDQAIQIVLEAQQASASLLQRRMRIGYNRASRLIDHMHMQGLIGAHEGSRPREVLMTLEQY-QQ-SKISS-- 911

WP_042233096_1 VDSRTILDMVGAEKLLGRGDMLYLPVGASKPVRVQGAFLSDQEVETVVNFVRTQQEANYREDMVPQVEEQQE-QQDEFGDELYDQAVQIVIEAKQASASLLQRRMRIGYARAGRLIDSMESNGIVGPHEGSKPREVLISLEQF-QH-NRMSS-- 865

WP_013919428_1 VDSRTILDSAGAEKLLGRGDMLYLPMGASKPVRVQGAFLSDQEVEAVVNYVRTQGQAEYVEDMVPMVEEKQD-DGDQYEDELYDQAVQIILEAKQASVSLLQRRMRVGYTRAARLIDTMEARGVVGPYEGSKPREVLMSLEQY-QV-MRMSS-- 867

WP_087432898_1 VDSRTILDMAGAEKLLGRGDMLFLPMGSSKPIRVQGAFLSDQEVESVVSFVSGQGQAEYNDDLVPEIDENGL-AADEPIDELYEQAIQMVLEAQQASASLLQRRMRIGYNRASRLIDHMHMQGIIGPHEGSRPREVLLTLEQY-QQ-NKISS-- 902

WP_062408022_1 VDSRTILDSVGAEKLLGRGDMLYLPMGASKPVRVQGAFLSDAEVEAVVSFVRTQGKAEYVEDMVPQVEERQE-EQNEFEDELYDQAVQIILEAKQASVSLLQRRMRVGYTRAARLIDTMEARGIVGPYEGSKPREVLVSLEQY-QI-NRMSS-- 851

WP_099520634_1 VDSRTILDMVGAEKLLGRGDMLYLPMGTSKPTRVQGAFLSDQEVEALVGYARGQAEAEYKEDLVPEIEEETA-SSDEVMDELYDQAVQIVVEAKQASVSLLQRRMRIGYTRAARLIDEMEARHIVGPYEGSKPREVLLTIDQL-EA-GRISS-- 941

WP_082110759_1 VDSRTILDMVGAEKLLGRGDMLYLPMGTSKPTRVQGAFLSDQEVEALVGYARGQAEAEYKEDLVPEIEEETA-SSEEVMDELYDQAVQIVVEAKQASVSLLQRRMRIGYTRAARLIDEMEARHIVGPYEGSKPREVLLTVDQL-EA-GRISS-- 872

WP_010269406_1 VDSRTILDMGGAEKLLGRGDMLFMPVGASKPIRVQGAFLSDKEVEAVVKFCKDQESANYQEEMVPEIEEVAD-QGEEFEDELFEQAVQIVLEAKQASVSLLQRRMRVGYTRAARLIDSMEAKGIVGPYEGSKPREVLVTMEQF-QQ-SRMSS-- 821

WP_079940749_1 VDSRTILDMVGAEKLLGRGDMLYLPVGASKPIRVQGAFLSDQEVEAVVRYCRDQQQANYQEEMVPEVEEQSD-THEEFEDELYDQAVQIVLEGGQASVSLLQRRMRIGYTRAARLIDAMEAKGIIGPYEGSKPREVLISLEQY-QQ-NRISS-- 833

WP_089523128_1 VDSRTILDMVGAEKLLGRGDMLFLPVGMSKPIRVQGAFLSDNEVEAIVEYARSQGEAEYKEDLVPELDENGGSNQGDDVDELFDQAMQIVVEARQASVSLLQRRMRVGYTRAARLIDQLEARGIVGPYEGSKPREVLMTLDQF-QA-NQKHA-- 898

WP_041854363_1 VDSRTILDMAGADKLLGRGDMLFLPVGMSKPIRVQGAFLSDQEVEAVVQFARDQAQAEYKEDLVPEVDDEPQ-EADDDLDELFDQAVQVVLEAKQASVSLLQRRMRIGYARAARLIDQMEARGIVGPYEGSKPREVLISEGQY-PP-GQISS-- 875

WP_042189402_1 VDSRTILDMPGAEKLLGRGDMLFLPMGASKPIRVQGAFMSDQEVETIVQYVSSQGEAEYDESIVPEVDDTIA-EDQEPQDELYEQAVTIVLEAKQASVSLLQRRMRVGYTRAARLIDSMEARGVIGPYEGSKPREVLVSLEQY-QH-NKISS-- 885

WP_068650580_1 VDSRTILDMAGAEKLLGRGDMLFLPMGSSKPIRVQGAYLTDQEVENIVDYVRDQGQAVYDESLVPEIEESSS-DPEEKLDELYDQAVQIILEAKQASVSLLQRRMRVGYTRAARLIDSMEARGIIGPYEGSKPREVLLSMEQY-QQ-NRMSS-- 894

WP_042138096_1 VDSRTILDMPGAEKLLGRGDMLFLPMGASKPIRVQGAFMSDQEVETIVQYVSSQGEANYDESLVPEVDDTLS-EDQDPQDELYEQAVQIVLEAKQASVSLLQRRMRVGYTRAARLIDAMEARSVIGPYEGSKPREVLMSLEQY-QH-NRISS-- 892

WP_068611148_1 VDSRTILDMAGAEKLLGRGDMLYLPMGASKPIRVQGAFLSDPEVEAVVNFCRDQEQAEYKEDLVPEVEDVPE-NGDQPLDELFDQAVQIILEAKQASVSLLQRRMRIGYTRAARLIDSMEAKGVVGPYEGSKPREVLISLEQY-QH-NRIS--- 873

WP_040948739_1 VDSRTILDMAGAEKLLGRGDMLFHPVGASKPTRVQGAFLSDQEVEAVVGYVSNQGKAGYQEDFVPQVEESTE-GEDIFEDELYDQAVQIVVEAKQASVSLLQRRMRVGYTRAARLIDSMEAKGVVGPYEGSKPREVLISPEQY-QM-SRMSS-- 854

WP_016312388_1 VDSRTILDMAGAEKLLGRGDMLYMPMGASKPIRVQGAFMSDQEVEAIVNYVRGQGQAEYDESLVPEVDEEVQ-ETEEVQDELFDQAVQIVLEAKQASVSLLQRRMRVGYTRAARLIDSMEARGIVGPYEGSKPREVLMSIDQY-KM-GRISS-- 893

WP_062326102_1 VDSRTILDMGGAEKLLGRGDMLFMPMGASKPVRVQGAFMSDEEVENIVNYVRGQGEAQYDESIVPEVDDSIQ-AADEVQDELYEQAVQIILEAKQASVSLLQRRMRVGYTRAARLIDSMEARGVIGPYEGSKPREVLVSLEQY-QQ-NKISS-- 912

WP_038571908_1 VDSRTILDMPGAEKLLGRGDMLFLPMGASKPIRVQGAFMSDQEVETIVQYVSSQGEAEYDESIVPEVDDTIT-EDQEPQDELYEQAVQIVLEAKQASVSLLQRRMRVGYTRAARLIDSMEARGVIGPYEGSKPREVLMSLEQY-QH-NKISS-- 886

WP_042128826_1 VDSRTILDMPGAEKLLGRGDMLFLPMGASKPIRVQGAFMSDQEVETIVQYVSSQGEAEYDESIVPEVDDTIA-EDQEPQDELYEQAVTIVLEAKQASVSLLQRRMRVGYTRAARLIDSMEARGVIGPYEGSKPREVLVSLEQY-QH-NKISS-- 885

WP_038593363_1 VDSRTILDMPGAEKLLGRGDMLFLPMGASKPIRVQGAFMSDQEVETIVQYVSSQGEANYDETLVPEVDDMVT-EDQEPQDELYEQAVQIVLEAKQASVSLLQRRMRVGYTRAARLIDAMEARAVIGPYEGSKPREVLMSLEQY-QH-NKISS-- 886

WP_055108403_1 VDSRTILDMAGAEKLLGRGDMLFMPMGASKPIRVQGAFMSDQEVEAIVGYVRGQGQAEYDDSLVPEVEEEVQ-GTEEIVDELFDQAVQIILEAKQASVSLLQRRMRIGYTRAARLIDSMEARGIVGPYEGSKPREVLISLEQF-KI-GKISS-- 898

WP_042214780_1 VDSRTILDMPGAEKLLGRGDMLFLPMGASKPIRVQGAFMSDQEVETIVQYVSSQGEANYDESLVPEVDDSIS-EDQEPQDELYEQAVQIVLEAKQASVSLLQRRMRVGYTRAARLIDAMEARSVIGPYEGSKPREVLMSLEQY-QH-SRISS-- 892

WP_042237084_1 VDSRTILDMPGAEKLLGRGDMLFLPMGASKPIRVQGAFMSDQEVETIVHYVSSQGEANYDESLVPEVDDTLS-EDQDPQDELYEQAVQIVLEAKQASVSLLQRRMRVGYTRAARLIDAMEARSVIGPYEGSKPREVLMSLEQY-QH-NRISS-- 892

WP_076168009_1 VDSRTILDMAGAEKLLGRGDMLFMPMGASKPIRVQGAFMTDQEVEIIVNYVRDQGEAKYDESLVPEVEETSQ-DADDQLDELYDQAVQIVLESKQASVSLLQRRMRVGYTRAARLIDSMEARGIIGPYEGSKPREVLVSLEQYQQQ-NRISS-- 892

WP_044877535_1 IDSRTILDMGGAEKLLGRGDMLFLPMGSSKPIRVQGAFINDQEVENIVDYVRDQGQAKYDEFLVPEVEEASA-NVDEMLDELYDQAVQIILEAKQASVSLLQRRMRVGYTRAARLIDSMESRGIIGPYEGSKPREVLLSIEQYQQQ-NKISS-- 876

WP_006036648_1 VDSRTILDMAGAEKLLGRGDMLFLPVGMSKPIRVQGAFLSDPEVEAVVAHARSQGEAEYKPELVPEIDESSN-DPDEIVDELFDQAVQIVVEAKQASVSLLQRRMRVGYTRAARLIDQMEARGVVGPYEGSKPREVLISADYP-GG-RVGGQ-- 954

WP_044478415_1 VDSRTILDMAGAEKLLGRGDMLFMPMGASKPIRVQGAFMSDQEVETIVNFVRDQGQAEYDESLVPEVEEEAG-GQEEVLDELYDQAVQIVLEAKQASVSLLQRRMRVGYTRAARLIDSMEARGVVGPYEGSKPREVLMTMDQY-RM-NKMTS-- 895

WP_091181726_1 VDSRTILDMVGAEKLLGRGDMLYLPVGMSKPIRVQGAFLSDQEVEALVEYARGQAEAEYKEDLVPEVEEESA-DPEEIVDELYDQAVQIVLEAKQASVSLLQRRMRVGYTRAARLVDQMEARGIVGPYEGSKPREVLMSMEQY-QA-SRIPS-- 906

WP_036656364_1 VDSRTILDMGGAEKLLGRGDMLFLPMGSSKPIRVQGAFLSDQEVETIVQYVSGQAEANYDDSIVPEVDDSNT-VDQEPQDELYEQAVQIVLEAKQASVSLLQRRMRVGYTRAARLIDSMEARGVIGSYEGSKPREVLISLEQY-QH-NRISS-- 883

WP_087916202_1 VDSRTILDMPGAEKLLGRGDMLFLPMGASKPVRVQGAFMSDQEVETIVQYVSSQGEATYDESIVPEVDDTVE-EDQEPQDALYEQAVQIILEAKQASVSLLQRRMRVGYTRAARLIDSMEARGVIGPYEGSKPREVLVSLEQY-QI-GRISS-- 880

WP_014281139_1 VDSRTILDMGGAEKLLGRGDMLFMPMGASKPVRVQGAFMSDQEVENIVNYVREQGEAQYDETLVPEVEELSA-DADEMLDELYDQAVNIILEAKQASVSLLQRRMRIGYTRAARLIDSMEARGVIGPYEGSKPREVLISMEQY-QQ-NKVSS-- 893

WP_039277135_1 VDSRTILDMGGAEKLLGRGDMLFMPMGASKPVRVQGAFMSDQEVENIVNYVREQGEAQYDETLVPEVEEVST-DADEMLDELYDQAVNIILEAKQASVSLLQRRMRIGYTRAARLIDSMEARGVIGPYEGSKPREVLISMEQY-QQ-NKVSS-- 893

WP_013370740_1 VDSRTILDMGGAEKLLGRGDMLFMPMGASKPVRVQGAFMSDQEVENIVNYVREQGEAQYDETLVPEVEEVST-DADEMLDELYDQAVNIILEAKQASVSLLQRRMRIGYTRAARLIDSMEARGVIGPYEGSKPREVLISMEQY-QQ-NKVSS-- 892

WP_023988230_1 VDSRTILDMGGAEKLLGRGDMLFMPMGASKPVRVQGAFMSDQEVENIVNYVREQGEAQYDETLVPEVEEVSA-DADEMLDELYDQAVNIILEAKQASVSLLQRRMRIGYTRAARLIDSMEARGVIGPYEGSKPREVLISMEQY-QQ-NKVSS-- 892

WP_058710517_1 VDSRTILDMGGAEKLLGRGDMLFMPMGASKPVRVQGAFMSDQEVENIVNYVREQGEAQYDETLVPEVEEVST-DADEMLDELYDQAVNIILEAKQASVSLLQRRMRIGYTRAARLIDSMEARGVIGPYEGSKPREVLISMEQY-QQ-NKVSS-- 892

WP_071640541_1 VDSRTILDMGGAEKLLGRGDMLFMPMGASKPVRVQGAFMSDQEVENIVNYVREQGEAQYDETLVPEVEEVSG-DADEMLDELYDQAVNIILEAKQASVSLLQRRMRIGYTRAARLIDSMEARGVIGPYEGSKPREVLISMEQY-QQ-NKVSS-- 892

WP_053325270_1 VDSRTILDMGGAEKLLGRGDMLFMPMGASKPVRVQGAFMSDQEVENIVNYVREQGEAQYDETLVPEVEEVSA-DADEMLDELYDQAVNIILEAKQASVSLLQRRMRIGYTRAARLIDSMEARGVIGPYEGSKPREVLISMEQY-QQ-NKVSS-- 888

WP_094155431_1 VDSRTILDMGGAEKLLGRGDMLFMPMGASKPVRVQGAFMSDQEVENIVNYVREQGEAQYDETLVPEVEEVSA-DADEMLDELYDQAVNIILEAKQASVSLLQRRMRIGYTRAARLIDSMEARGVIGPYEGSKPREVLISIEQY-QQ-NKVSS-- 888

WP_068655629_1 VDSRTILDMAGAEKLLGRGDMLFMPMGSSKPIRVQGAYLTDQEVENIVDFVRDQGQAEYDESLVPEIDESAT-NDGEVLDELYDQAVQVILEAKQASVSLLQRRMRVGYTRAARLIDSMEARGVIGPYEGSKPREVLVSMEQY-QQ-NRISS-- 892

WP_015736290_1 VDSRTILDMAGAEKLLGRGDMLFMPMGSSKPIRVQGAFMSDHEVENIVDFVRDQGQAEYDESLVPEIEESAG-ADEEELDELYEQAVTIVLEAKQASVSLLQRRMRVGYTRAARLIDSMEARGVIGPYEGSKPREVLMSIEQYQQQ-QNRIS-- 881

WP_036642107_1 VDSRTILDMAGAEKLLGRGDMLFMPMGSSKPIRVQGAFMTDQEVENIVDFVRDQAQAEYDESLVPEIDESSN-SDDEELDELYEQAVTIVLEAKQASVSLLQRRMRVGYTRAARLIDSMEARGVIGPYEGSKPREVLMSMEQYQQQ-QNRIS-- 881

WP_108465295_1 VDSRTILDMAGAEKLLGRGDMLFMPMGASKPVRVQGAFMSDQEVENIVNFVRDQGQAEYDESLVPEVDETIQ-EPEEQLDELYDQAVQIVVEAKQASVSLLQRRMRVGYTRAARLIDSMEARGVVGPYEGSKPREVLISVEQY-RQ-SKISS-- 889

WP_045672049_1 VDSRTILDMVGAEKLLGRGDMLFLPVGMSKPIRVQGAFLSDQEVEAVVEFARGQAEAEYKEDLVPEIEEPSG-DDNELLDELFDQAVQIVLEAKQASVSLLQRRMRVGYTRAARLVDQMEAKGIVGPYEGSKPREVLLSLDQY-QA-NQKHA-- 887

WP_036651575_1 VDSRTILDMAGAEKLLGRGDMLFMPMGSSKPIRVQGAFMTDQEVEIIVDYVREQGEAKYDESLVPEIEETTT-DGNEVLDELYDQAVQIVLEAKQASVSLLQRRMRVGYTRAARLIDSMEARGIIGPYEGSKPREVLMSMEQYQQN-NRISS-- 885

WP_074094588_1 VDSRTILDMGGAEKLLGRGDMLFMPMGASKPVRVQGAFMSDEEVENIVNYVRGQGEAQYDESLVPEVDDSIQ-AEDEVQDELYEKAVQIILEAKQASVSLLQRRMRVGYTRAARLIDSMESRGVIGPYEGSKPREVLVSLEEY-QQ-NKISS-- 912

WP_020428017_1 VDSRTILDMPGAEKLLGRGDMLFLPMGASKPIRVQGAFMSDQEVETIVQYVSSQGEANYDESIVPEVDDTVT-EDDEPQDELYEQAVQIVLEAKQASVSLLQRRMRVGYTRAARLIDAMEARSVIGPYEGSKPREVLMSLEQY-QH-NRISS-- 883

WP_025704675_1 VDSRTILDMPGAEKLLGRGDMLFLPMGASKPIRVQGAFMSDQEVETIVQYVSSQGEANYDESIVPEVDDTVT-EDNEPQDELYEQAVQIVLEAKQASVSLLQRRMRVGYTRAARLIDAMEARSVIGPYEGSKPREVLMSLEQY-QH-NRISS-- 882

WP_047171161_1 VDSRTILDMPGAEKLLGRGDMLFLPMGASKPVRVQGAFMSDQEVETIVQFVSSQGEAEYDETLVPEVDDMVS-EDQEPQDELYEQAVQIVLEAKQASVSLLQRRMRVGYTRAARLIDAMEARSVIGPYEGSKPREVLMSLEQY-QQ-NRISS-- 897

WP_042177473_1 VDSRTILDMPGAEKLLGRGDMLFLPMGASKPVRVQGAFMSDQEVETIVQFVSSQGEAEYDETLVPEVDDMVN-EDQEPQDELYEQAVQIVLEAKQASVSLLQRRMRVGYTRAARLIDAMEARSVIGPYEGSKPREVLMSLEQY-QQ-NRISS-- 896

WP_099479291_1 VDSRTILDMAGAEKLLGRGDMLFMPMGSSKPIRVQGAFMSDQEVENIVSFVRDQGQAEYDESLVPEIEESAQ-SGDEELDELYDQAVTIVLEAKQASVSLLQRRMRVGYTRAARLIDSMEARGVIGPYEGSKPREVLMSLEQYQQQ-QNRIS-- 879

WP_038696628_1 VDSRTILDMAGAEKLLGRGDMLFMPMGSSKPIRVQGAFMSDAEVEAIVHYVSSQGEAEYDESIVPEIDEMSS-EAEEPQDELYDQAVQIVLEAKQASVSLLQRRMRVGYTRAARLIDSMEARGVIGPYEGSKPREVLISLEQY-QQ-NRISS-- 888

WP_015844967_1 VDSRTILDMVGAEKLLGRGDMLYLPVGMSKPIRVQGAFLSDQEVEALVDYARGQAEAEYKEDLVPEVEEESA-DPEEVLDELYDQAVQIVLEAKQASVSLLQRRMRVGYTRAARLVDQMEARGIVGPYEGSKPREVLMSMEQY-QS-SRIPS-- 915

WP_025335307_1 VDSRTILDMAGAEKLLGRGDMLFMPMGSSKPIRVQGAFMSDAEVEAIVHYVSSQGEAEYDESIVPEVDDTAA-EAEEPQDELYEQAVQIVLEAKQASVSLLQRRMRVGYTRAARLIDSMEARGVIGPYEGSKPREVLISLEQY-QQ-NRISS-- 882

WP_036622367_1 VDSRTILDMAGAEKLLGRGDMLYMPMGASKPTRVQGAFMSDQEVETIVNYVRAQGQAEYDESLVPEVDDEVQ-EPEEELDELYDQAVQIVLEAKQASVSLLQRRMRVGYTRAARLIDSMEARGIVGPYEGSKPREVLMSIEQY-KM-GRISS-- 905

WP_042160596_1 VDSRTILDMVGAEKLLGRGDMLFLPVGMSKPLRVQGAFLSDQEVEALVDYSRGQAEAEYKEDLVPEVEEESN-DPNDIVDELYDQAVQIVLEAKQASVSLLQRRMRIGYTRAARLIDQMEARSIVGPYEGSKPREVMLTMEQY-QA-GRISS-- 922

WP_062492619_1 VDSRTILDMAGAEKLLGRGDMLFLPVGMSKPIRVQGAFLSDQEVEAIVSYVRSQGEAEYNNDLVPEADEIAA-DEDEMLDELYDQAVQIVLEAKQASVSLLQRRMRIGYTRAARLIDQMEAKGVVGPYEGSKPRDVLISSEQY-HG-RISS--- 880

WP_068694663_1 VDSRTILDMAGAEKLLGRGDMLFMPMGASKPVRVQGAFMSDQEVETIVQYVSSQGQAEYDESLVPEVDDTQQ-EMEEVLDELYDQAVQIILEAKQASVSLLQRRMRIGYTRAARLIDSMEARGIVGPYEGSKPREVLVSAEQY-NQ-NRISS-- 931

WP_025694771_1 VDSRTILDMAGAEKLLGRGDMLFMPMGSSKPVRVQGAFMSDAEVEAIVHYVSSQGEAEYDESIVPEVDDAAP-ETEEPQDELYEQAVQIVLEAKQASVSLLQRRMRVGYTRAARLIDSMEARGVIGPYEGSKPREVLISLEQY-QQ-SRISS-- 872

WP_042207076_1 VDSRTILDMAGAEKLLGRGDMLFMPMGSSKPVRVQGAFMSDAEVEAIVHYVSSQGEAEYDESIVPEVDDATA-ETGEPQDELYEQAVQIVLEAKQASVSLLQRRMRVGYTRAARLIDSMEARGVIGPYEGSKPREVLISLEQY-QQ-SRISS-- 871

WP_068619226_1 VDSRTILDMAGAEKLLGRGDMLFMPMGASKPIRVQGAFMSDQEVEAIVDYVRGQAEAEYDETLIPEVDDSVQ-EPEEQLDELYDQAVQIVLEAKQASVSLLQRRMRVGYTRAARLIDSMEARGVVGPYEGSKPREVLTSIEQY-KQQNRVSS-- 869

WP_041063710_1 VDSRTILDMVGAEKLLGRGDMLFLPMGASKPIRVQGAFLSDSEVESVVAYARGQAEAEYNEDLVPEIDETDGQAPDEIMDELFDQAVQIVLDAKQASVSLLQRRMRIGYTRAARLIDSMEAKGIVGPYEGSKPREVLMTMEQY-Q--QRIPS—- 857

**paen9.01226, DNA mismatch repair ATPase MutL**

WP_045672515_1 --MGIIRVLDEQLANQIAAGEVVERPASVVKELVENAVDAGATTIDIAVEEGGLQLIRVTDNGSGIQHEDMEIAFSRHATSKIQSDRDLFRIASLGFRGEALPSIAAVARVSCVSSAEDSGLGRLIRMEGGKLTADEHAGASQGTDMSVSDLFFNTPARLKYMKSIQTELGHISDYVYRIALAHPGIAFSLTHNGGSLLR 198

WP_016822099_1 --MSKIRVLDEHIANQIAAGEVVERPASVVKELVENAIDADGTRVDVWVEEGGLQSIRVTDNGSGIEPEDVETAFYRHATSKIGHGRDLFQITSLGFRGEALPSIAAVSKVELLTAAGDDGRARKLVIEGGKLVLHEDAAGRQGTDFTVRDLFYNTPARLKYMKTIQTELGHISDVLYRMALSHPEVAFTLRHNGNTLLQ 198

WP_102163696_1 --MSKIRVLDEHIANQIAAGEVVERPASVVKELVENAIDAGGTRVDVWVEEGGLQSIRVTDNGSGIEPEDVETAFYRHATSKIGHGRDLFQITSLGFRGEALPSIAAVSKVELLTAAGDDGRARKLVIEGGKLMLHEDAAGRQGTDFTVRDLFYNTPARLKYMKTIQTELGHISDVLYRMALSHPEVAFTLRHNGNTLLQ 198

WP_013371549_1 --MSKIRVLDEHIANQIAAGEVVERPASVVKELVENAIDAGGTRVDVWVEEGGLQSIRVTDNGSGIEPEDVETAFYRHATSKIGHGRDLFQITSLGFRGEALPSIAAVSKVELLTAAGDDGRARKLVIEGGKLMLHENAAGRQGTDFTVRDLFYNTPARLKYMKTIQTELGHISDVLYRMALSHPEVAFTLRHNGNTLLQ 198

WP_074095505_1 --MAKIHVLDEHIANQIAAGEVVERPASVVKELLENSVDAGATKIEVTVEEGGLLSIRVKDNGTGIEPEDMETAFYRHATSKIAHGRDLFQITSLGFRGEALASIAAVSKVEVLSASGNDGRGRRIAIEGGNLVSHEDATSPQGTDFAVRELFYNTPARLKYMKTIQTELGHISDVLYRMAMSHPNISFRLRHNENVLLQ 198

WP_087435490_1 --MAVIKILDEHIANQIAAGEVVERPSSVVKELVENAIDAGSTRIDVTVEEGGLQLIRVKDNGSGIGDDDVENAFQRHATSKIVTGKDLFAIRSLGFRGEALPSIAAVAKVDVVTATDDSGLGRRLVIEGGTVKTLEPAQSMQGTEFTVRELFYNTPARLKYMKTIQTELGHISDLIYRLALSYPNIAFTLNHNDNTLLQ 198

WP_023989046_1 --MSKIRVLDEHIANQIAAGEVVERPASVVKELVENAIDAGGTRVDVWVEEGGLQSIRVTDNGSGIEPEDVETAFYRHATSKIGHGRDLFQITSLGFRGEALPSIAAVSKVELLTAAGDDGRARKLVIEGGKLLLHEDAASRQGTDFTVRELFYNTPARLKYMKTIQTELGHISDVLYRMALSHPEVAFTLRHNGNTLLQ 198

WP_053325620_1 --MSKIRVLDEHIANQIAAGEVVERPASVVKELVENAIDAGGTRVDVWVEEGGLQSIRVTDNGSGIEPEDVETAFYRHATSKIGHGRDLFQITSLGFRGEALPSIAAVSKVELLTAAGDDGRARKLVIEGGKLLLHEDAASRQGTDFTVRELFYNTPARLKYMKTIQTELGHISDVLYRMALSHPEVAFTLRHNGNTLLQ 198

WP_071641229_1 --MSKIRVLDEHIANQIAAGEVVERPASVVKELVENAIDAGGTRVDVWVEEGGLQSIRVTDNGSGIEPEDVETAFYRHATSKIGHGRDLFQITSLGFRGEALPSIAAVSKVELLTAAGDDGRARRLVIEGGKLLLHEDAASRQGTDFTVRELFYNTPARLKYMKTIQTELGHISDVLYRMALSHPEVAFTLRHNGNTLLQ 198

WP_005549848_1 --MAIIKVLDEHIANQIAAGEVVERPSSVVKELVENSIDAGATRIDVSVEEGGLQLIRVKDNGSGIGDDDAENAFLRHATSKIQTGKDLFSIRSLGFRGEALPSIAAVAKVELVTSTDSSGLGRRLVIEGGTIKSFEPAQSMQGTEITVRDLFYNTPARLKYMKTIQTELGHISDLIYRLAMSYPNIAFTLKHNEHTLLQ 198

WP_068617384_1 --MGQIQILDEHIANQIAAGEVVERPSSVVKELVENAIDAGAKRIDVTVEEGGLSLIRIIDNGSGIEPEDCETAFYRHATSKLSLGRDLFQIRTLGFRGEALPSIAAVSKVELVSSTSELSLGKRIVIEGGSLKMNEDTPAPQGTDISVRELFYNTPARLKYMKTIQTELGHISDVMYRQALAHAEIAFTLRHNGNVLLQ 198

WP_094154817_1 --MSKIRVLDEHIANQIAAGEVVERPSSVVKELVENAIDAGGTRVDVSVEEGGLQSIRVTDNGSGIEPEDVETAFYRHATSKIGHGRDLFQITSLGFRGEALPSIAAVSKVELLTAAGDDGRARKLVIEGGKLMLHEDAAARQGTDFTVRELFYNTPARLKYMKTIQTELGHISDVLYRMALSHPEVAFTLRHNGNTLLQ 198

WP_042177336_1 --MAKIHVLDEHIANQIAAGEVVERPASVVKELVENAIDAGSTRIEVTVEEGGLQSIRVKDNGSGIEPDDCETAFYRHATSKIATGRDLFQITSLGFRGEALPSIAAVSKLTLLTASGDDGKGRQIDIEGGKLIRNEDQPSGKGSDMAVRELFYNTPARLKYMKSIQTELGHISDAMYRMALAHPDISFTLQHNGNQLLR 198

WP_037288812_1 --MAKIKVLDEHIANQIAAGEVVERPASVVKELVENAIDAGATKVDVWTEEGGLSSIRVTDNGSGIDPEDCETAFYRHATSKISGGRDLFQITSLGFRGEALPSIASVSKVEVVTACGEDGAGRRLVIEGGTLKSNEDAPASRGTDFRVKELFYNTPARLKYMKTVQTELGHISDYMYRMALSHPEVAFTLRHNGNSLLQ 198

WP_014281438_1 --MSKIRVLDEHIANQIAAGEVVERPASVVKELVENAIDAGGTRVDVWVEEGGLQSIRVTDNGSGIEPEDVETAFYRHATSKIGHGRDLFQITSLGFRGEALPSIAAVSKVELLTAAGDDGRARKLVIEGGKLVLHEDAAARQGTDFTVRELFYNTPARLKYMKTIQTELGHISDVLYRMALSHPEVAFTLRHNGNTLLQ 198

WP_068700502_1 --MGKIQVLDEHIANQIAAGEVVERPASVIKELIENSIDAGATKIEVSVEEGGLDSIRITDNGSGIDPEDCETAFYRHATSKLSSSRDLYQIRTLGFRGEALPSIAAVAKVKLLTANSDSGLGREVVIEGGNLKSSEDAPAPQGTDITVKELFYNTPARLKYMKTIQTELGHISDVVYRQALAHPEIGFVLRHNGNLLLQ 198

WP_010273601_1 --MGRIRLLDDHIANQIAAGEVVERPSSVVKELVENSIDAGSTRIEVFLEEGGIQLIRVKDNGSGMEKSDVQLAFHRHATSKISASKDLFRIRSLGFRGEALPSIAAVAKVECVTSDNTSGLGTRIVVAGGQVLAAEEAAASRGTEITVRELFYNTPARLKYMKTIQTELGHVSDYMYRLALAHPGIAFSLKHNHNSLLH 198

WP_076171200_1 --MAKIRVLDEHIANQIAAGEVVERPASVVKELVENAIDAGSSRIEVTVEEGGLESIRVTDDGSGIEPEDVSTAFYRHATSKIENSRDLFNITSLGFRGEALPSIAAVAKVELVTASADNGLGRRIVIEGGRLVTEEDTAAPKGTDLTVRELFYNTPARLKYMKTIQTELGHISDYMYRIALSHPEIAITLRHNGNVLLQ 198

WP_062328181_1 --MAKIHVLDEHIANQIAAGEVVERPASVVKELLENSVDAGASKIDVTVEEGGLLRIRVKDNGSGIEPEDMEKAFYRHATSKIAHGRDLFQITSLGFRGEALASIAAVSKVKVLSASGNDGRGRRIVIEGGKLLSHEDGTSPQGTDFEVKELFFNTPARLKYMKTIQTELGHISDVLYRMAISHPNISFTLRHNENTLLQ 198

WP_042228138_1 --MGKIRLLDEHIANQIAAGEVVERPASVVKELVENSIDAGSSRIDVVAEEGGLQLIRVTDNGSGIESEDCELAFYRHATSKIQSGKDLFSIRTLGFRGEALPSIAAVSKTEFVTSTDGSGLGRKLAVEGGDVVTQEETSARKGTDISVKELFYNTPARLKYMKTIQTELGHISDYMYRLALAHPEIAFTLKHNGNSLLQ 198

WP_042137912_1 --MAKIHVLDEHIANQIAAGEVVERPASVVKELVENAIDAGSTRIEVSVEEGGLQSIRVKDNGSGIEPEDCETAFYRHATSKIANGRDLFLITSLGFRGEALPSIAAVSKLTLLTASGDDGKGRLLEIEGGKLILSEDAPSGRGSDLTVRELFYNTPARLKYMKSIQTELGHISDAMYRMALAHPDISFTLQHNSNQLLH 198

WP_042236923_1 --MAKIHVLDEHIANQIAAGEVVERPASVVKELVENAIDAGSTRIEVSVEEGGLQSIRVKDNGSGIEPEDCETAFYRHATSKIANGRDLFLITSLGFRGEALPSIAAVSKLTLLTASGDDGKGRLLEIEGGKLILSEDAPSGRGSDLTVRELFYNTPARLKYMKSIQTELGHISDAMYRMALAHPDISFTLQHNGNQLLH 198

WP_042214549_1 --MAKIHVLDEHIANQIAAGEVVERPASVVKELVENAIDAGSKRIEVSVEEGGLQSIRVKDNGSGIEPEDCETAFYRHATSKIANGRDLFLITSLGFRGEALPSIAAVSKLTLLTASGDDGKGRLLEIEGGKLIRNEDAPSGQGSDLTVRELFYNTPARLKYMKSIQTELGHISDAMYRMALAHPDISFTLHHNGNQLLH 198

WP_038696521_1 --MARIHVLDEHIANQIAAGEVVERPASVVKELVENAIDAGSTRIEVSIEEGGLQSIKVKDNGFGIEPEDCETAFYRHATSKIESGRDLFQITSLGFRGEALPSIAAVAKVSLLTASSDDGKGKLLEIEGGKLMVNEPAPSGRGTEMTVRELFYNTPARLKYMKSIQTELGHISDAMYRMALAHPQISFTLRHNGNQLLQ 198

WP_042128372_1 MKLAKIHILDEHIANQIAAGEVVERPASVVKELVENAIDAGSTRIEVSVEEGGLQSIRVKDNGSGIEPEDCETAFYRHATSKIANGRDLFQITSLGFRGEALPSIAAVSKVSLLTATADDGKGRLIDIEGGNLIRNEDSPSGRGSDLAVRELFFNTPARLKYMKSIQTELGHISDAMYRMALAHPGISFTLHHNGNQLLH 200

WP_038571591_1 --MAKIHILDEHIANQIAAGEVVERPASVVKELVENAIDAGSTRIEVSVEEGGLQSIRVKDNGSGIDPEDCETAFYRHATSKILNGRDLFQITSLGFRGEALPSIAAVSKVSLLTATADDGKGRLVDIEGGNLIRNEDAPSGRGSDLAVRELFFNTPARLKYMKSIQTELGHISDAMYRMALAHPGISFTLHHNGNQLLH 198

WP_039873825_1 --MAKIHVLDEHIANQIAAGEVVERPASVVKELVENAIDAGSTRIEVTVEEGGLQSIRVKDNGSGIEPDDCETAFYRHATSKIASGRDLFQITSLGFRGEALPSIAAVSKLTLLTASGDDGKGRQIDIEGGKLIRNEDQPSAKGSDMAVRELFYNTPARLKYMKSIQTELGHISDAMYRMALAHPDISFTLQHNGNQLLR 198

WP_079940587_1 --MGRIQLLDEQISNQIAAGEVVERPASVVKELVENSIDASSTRVDVSIEEGGLQSIRVRDNGSGIEAEDCELAFFRHATSKIARGKDLFSIRTLGFRGEALPSIAAVSKVTCTTATDNNGLGRQIVIEGGNICSVQETAASKGTDIIVKDLFYNTPARLKYMKTIQTELGHISDYMYRLALAHPDRSFTLRHNGNTLLQ 198

WP_046504127_1 --MAKIHVLDEHIANQIAAGEVVERPASVVKELVENAIDAGSTRIEVSVEEGGLQSIRVKDNGSGIDPEDCETAFYRHATSKIVNGRDLFQITSLGFRGEALPSIASVSKLSLLTSSNEDGKGRALDIEGGKLIRNEDEPAGKGSDLAVRELFYNTPARLKYMKSVQTELGHISDTMYRVALAHPSISFTLHHNGNQLLH 198

WP_042266908_1 --MAKIHVLDEHIANQIAAGEVVERPASVVKELVENAIDAGSTRIEVSVEEGGLQSIRVKDNGSGIDPEDCETAFYRHATSKILNGRDLFQITSLGFRGEALPSIASVSKLSLLTSSNEDGKGRALDIEGGKLIRNEDEPSGKGSDLAVRELFYNTPARLKYMKSVQTELGHISDTMYRVALAHPSISFTLHHNGNQLLH 198

WP_025335246_1 --MVKIHVLDEHIANQIAAGEVVERPASVVKELVENAIDAGSTRIEVAVEEGGLQSIRVKDNGSGIEPEDCETAFYRHATSKIENGRDLFQITSLGFRGEALPSIAAVAKVSLTTASSDDGRGRRLEIEGGKLTASEDTAHGRGTEMTVRELFYNTPARLKYMKSIQTELGHISDAMYRMALAHPGISFTLQHNGNQLLH 198

WP_108465825_1 --MAKIQILDEHIANQIAAGEVVERPSSVVKELVENAIDAGSTKVDVMVEEGGLMSIRVTDNGSGIEPEDCETAFYRHATSKLSSGRDLFQIRSLGFRGEALPSIAAVAKVQLISASSDSGLGYKVSIEGGKLKESEAAPAPKGTDLVIRELFYNTPARLKYMKTIQTELGHISDVMYRQALAHPEIGFSLRHNGNALLQ 198

WP_038593031_1 --MAKIHVLDEHIANQIAAGEVVERPASVVKELVENAIDAGSTRIEVSVEEGGLQSIRVKDNGSGIEPEDCETAFYRHATSKIANGRDLFLITSLGFRGEALPSIAAVSKVSLLTASSDDGKGRLIDIEGGKLIRNEDTPSGKGSDLAVRELFFNTPARLKYMKSIQTELGHISDAMYRMALAHPSISFTLHHNGNQLLH 198

WP_081951145_1 --MAKIHILDEHIANQIAAGEVVERPASVVKELVENAIDAGSTRIEVSVEEGGLQSIRVKDNGSGIEPEDCETAFYRHATSKIANGRDLFQITSLGFRGEALPSIAAVSKVSLLTATADDGKGRLIDIEGGNLIRNEDSPSGRGSDLAVRELFFNTPARLKYMKSIQTELGHISDAMYRMALAHPGISFTLHHNGNQLLH 198

WP_068648544_1 --MAKIHILDEHIANQIAAGEVVERPASVVKELVENAIDAGSTKIEVTIEEGGLQSIRVTDNGSGIEPEDCESAFYRHATSKITNGRDLFHITSLGFRGEALPSIASVSKIALTTSSQEDGLGRKLIIEGGNLLSNDDSAAPRGTDFVVKELFYNTPARLKYMKTIQTELGHISDYMYRIALSHPGIAITLRHNDNTLLQ 198

WP_036656101_1 --MAKIHILDEHIANQIAAGEVVERPASVVKELVENAIDAKSTVIEVSVEEGGLQSIRVKDNGSGIEADDCETAFYRHATSKIANGRDLFLITSLGFRGEALPSIAAVSKVSLLTASADDGKGRLIKIEGGKLICNEDAPSGQGTDFEVRELFFNTPARLKYMKSVQTELGHISDAMYRMALAHPDISFRFQHNGNQLLH 198

WP_015734656_1 --MGIIRILDEHIANQIAAGEVVERPASVVKELVENAIDAGSTKIDVSVEEGGLDSIRVTDNGAGIDPEDCETAFYRHATSKIAEGRDLFQITSLGFRGEALPSIAAVSKVRLVTSNAQDGRGRKIEIEGGHLRVNEETAAPRGTDFLVKELFYNTPARLKYMKTIQTELGHISDYMYRLALSRPDIAFTLRHNGNSLLQ 198

WP_016312025_1 --MAKIAILDEHIANQIAAGEVVERPASVVKELVENAIDAGATKIEVAVEEGGLQLIRVTDNGSGIEPEDCETAFYRHATSKITSGRDLFQIRSLGFRGEALPSIAAVAKVRLVSSSDDSGLGRVIEIEGGSLKLNEDIAAPQGTDIVVKELFYNTPARLKYMKTIQTELGHISDTMYRQALAHPEIAFTLRHNGNTLLQ 198

WP_087920285_1 --MAKIHVLDEHIANQIAAGEVVERPASVVKELVENAIDAGSTRIEVSVEEGGLQSIRVKDNGSGIEPEDCETAFYRHATSKIANGRDLFQITSLGFRGEALPSIAAVSKVSMLTATADDGKGRLIDIEGGKLLRNEDSPAGRGSDLAVKELFYNTPARLKYMKSIQTELGHISDAMYRMALAHPDISFTLHHNSNQLLH 198

WP_025694959_1 --MAKIHVLDEHIANQIAAGEVVERPASVVKELVENAIDAGSTRIEVAVEEGGLQSIRVKDNGSGIEAEDCETAFYRHATSKIANGRDLFQITSLGFRGEALPSIAAVAKVSLLTASSDDGRGRLLEIEGGKLTVNEDAARSRGTEMNVRELFYNTPARLKYMKSIQTELGHISDAMYRMALAHPGISFTLHHNGNQLLH 198

WP_042207003_1 --MAKIHVLDEHIANQIAAGEVVERPASVVKELVENAIDAGSTRIEVAVEEGGLQSIRVKDNGSGIEAEDCETAFYRHATSKIANGRDLFQITSLGFRGEALPSIAAVAKVSLLTASSDDGRGRLLEIEGGKLTVNENAARSRGTEMNVRELFYNTPARLKYMKSIQTELGHISDAMYRMALAHPAISFTLHHNGNQLLH 198

WP_027084813_1 --MGIIRPLDEHLANQIAAGEVVERPASVLKELIENAVDAGASRIDVSAEEGGLTLLRVADDGTGIEADDMLVAFQRHATSKIATGKDLFLIATLGFRGEALPSIAAVAKVKCVSATDNSGLGRCVEIEGGALVTDKEINAPKGTEMVVRDLFFNTPARLKYMKTVQTELGHLSDVVYRQALARPDIAFTFTHNGNLLLR 198

WP_044876703_1 --MAKIHILDEHIANQIAAGEVVERPASVVKELVENAIDAGSTKIVVTVEEGGLQSIRITDNGSGIDPEDCETAFYRHATSKIMFGRDLFHITSLGFRGEALASIAAVSKVELITSSRDDGLGSRLVIEGGKLIVKEDTASSRGTDFVVRELFYNTPARLKYMKTIQTELGHISDYMYRIALSHPGIGITFRHNDNTLLQ 198

WP_099476734_1 --MGKIRVLDEHIANQIAAGEVVERPASVVKELVENAIDAGSTRIDVAVEEGGLDSIRVTDNGSGIDPDDCETAFYRHATSKIAEGRDLFQIISLGFRGEALPSIAAVSKVRVVTSNEQDGRGRKIEIEGGNLRVNEETASPKGTDFLVRELFYNTPARLKYMKTIQTELGHISDYMYRLALSRPDIAFTLRHNGNTLLQ 198

WP_036637535_1 --MGIIRILDEHIANQIAAGEVVERPASVVKELVENAIDAGSTKIDVTVEEGGLDSIRVTDNGAGIDPEDCETAFYRHATSKITEGRDLFQITSLGFRGEALPSIAAVSKVRLVTSNVQDGRGRKIEIEGGHLRVNEETAAPRGTDFLVKELFFNTPARLKYMKTIQTELGHISDYMYRLALSRPDIAFTLRHNGNSLLQ 198

WP_016362589_1 --MGKITILDEHIANQIAAGEVVERPSSVVKELVENAVDAGSTRVDVSIEEGGLQLIRVTDNGGGIEEDDVEAAFFRHATSKIATSQDLFSIRTLGFRGEALPSIAAVSKVECLTSAGSTGLGTRLRIEGGTVRLKEDAAAQRGTDLSVRELFYNTPARLKYMKTVQTELGHVTDYMYRLALAYPNIAFTLKHNGHVLLQ 198

WP_068658264_1 --MAKIHVLDEHIANQIAAGEVVERPASVVKELVENAIDAGSTKVEVVIEEGGLQSIRVSDNGSGIEPDDCEAAFYRHATSKIQHGRDLFNITSLGFRGEALASIAAVSKVTLTTSSNDDGLGRKLIIEGGKLITNEDTAASRGTSFIVRELFYNTPARLKYMKTIQTELGHISDYIYRIALSHPNIGIILRHNENTLLQ 198

WP_062408748_1 --MGKITILDEHIANQIAAGEVVERPSSVVKELVENAIDAGSSRIDVTIEEGGLQLIRVTDNGSGMDREDCETAFYRHATSKIASSHDLFQIRTLGFRGEALPSIAAVSKVELLTSPDSSGLGTRLVLEGGSVLERSDATAPQGTDITVRELFYNTPARLKYMKTIQTELGHVTDYIYRLALAHPNIAITLKHNGHVLAQ 198

WP_042161919_1 --MGKIKVLDEQLANQIAAGEVVERPASVIKELVENAVDAGSSQIDITVEEGGLTLIRVTDNGSGIEAGDIPTAFQRHATSKIATSSDLFRIASLGFRGEALPSIAAVSRLTAISSTETTGLAHRLVIEGGEIKTDEPANAPQGTDMTVRDLFFNTPARLKYMKAIQTELGHISDYIYRIALAHPGIAFTLKHNGNTLLR 198

WP_068609064_1 --MAIIQILDEHIANQIAAGEVVERPSSVVKELVENAIDAGATAVDVVIEEGGLSLIRVTDNGTGMDAEDLETAFFRHATSKIATGKDLFQISSLGFRGEALPSIAAVSKVECISSSGGDGLGRRIVIEGGTVKVVEDAAAMRGTVFSVKELFFNTPARLKYMKTIQTELGHISDFIYRLALSHPGIAFTLKHNGNSLIQ 198

WP_046232528_1 --MGKILVLDEQLANQIAAGEVVERPASVIKELVENAVDAGSSTIDITVEEGGLNLIRVTDNGSGIPAEEIETAFQRHATSKISTSSDLFRIASLGFRGEALPSIAAVSRLECLSSTGHTGLGHKLSIEGGTITASIPAATPQGTDMTVRDLFYNTPARLKYMKAVQTELGHISDYIYRIALAHPGIAFTLKHNGNTLLR 198

WP_099520158_1 --MGKILVLDEQLANQIAAGEVVERPASVIKELVENAVDAGSSTIDITVEEGGLNLIRVTDNGSGIPAEEIETAFQRHATSKIATSSDLFRIASLGFRGEALPSIAAVSRLECLSSTGQTGLGHKLSIEGGTITASIPAAMPQGTDMTVRDLFYNTPARLKYMKAVQTELGHISDYIYRIALAHPGIAFTLKHNGNTLLR 198

WP_015844205_1 --MGKIQVLDEQLANQIAAGEVVERPASVIKELVENAIDAGSSVIDITIEEGGLSFIRVTDNGAGIEPGDMETAFQRHATSKISSSSDLFKIASLGFRGEALPSIAAVSRLECVSSIDSTGLAQRLFIEGGTVTASEPANSPQGTDMSVRDLFYNTPARLKYMKAIQTELGHITDYVYRIALAHPGIAITLKHNGSVLLR 198

WP_091189189_1 --MGKIQVLDEQLANQIAAGEVVERPASVIKELVENAIDAGSSVIDITVEEGGLSFIRVTDNGSGIEPADMETAFQRHATSKISSSSDLFKIASLGFRGEALPSIAAVSRLECVSSMDSTGLAQRLIIEGGTVTANEPVNAPQGTDMSVRDLFYNTPARLKYMKAIQTELGHITDYVYRIALAHPGIAITLKHNGSVLLR 198

WP_006036759_1 --MGNIRVLDEQLANQIAAGEVVERPASVVKELVENAVDAGATTIDLTIEEGGLNLIRVIDNGAGIEPDEIETAFQRHATSKLLSSADLFRIASLGFRGEALPSIAAVSRVTCISSPTKDGLGKQLVIEGGTVQQSGPVSAPQGTEMSVRDLFYNTPARLKYMKSIQTELGHISDYVNRIALAHPGIAFSLKHNGNELLR 198

WP_062492889_1 --MGKIRILDEQLANQIAAGEVVERPASVVKELVENAVDAGSTAIDIAVEEGGISLIRVTDNGSGIDPDDLETAFQRHATSKIASGSDLFRIASLGFRGEALPSIAAVSRVECVSASDDSGLGRRLAMNGGTVVALEPVNAPRGTDITVRDLFYNTPARLKYMKSIQTELGHLSDYVNRIALAHPGIAFTLRHNGNLLLR 198

WP_015254643_1 --MGIIQVMDEQLANRIAAGEVVERPASVVKELAENAIDAGATEIDIAVEEGGLTFIRVRDNGSGIEADDLERAFQRHATSKIRTDRDLFRIATLGFRGEALPSIAAVSRVSCLSAADDSGLGRKIVLEGGHVRSIEQAASPKGTEIVVRDLFFNTPARLKYMKTIQTELGHISDCVNRLALSHPGIAFTLTHNGNPLLR 198

WP_089523565_1 --MGKIKVLDEHLANQIAAGEVVERPASVVKELVENAVDAGATSIDITIEEGGFSLIRVQDNGSGIEVEDMETAFQRHATSKLLTDRDLFRIASLGFRGEALPSIAAVARVDCVSASNDSGLGRRIVMEGGKLTTDEHAGAGQGTDMTVRDLFFNTPARLKYMKSIQTELGHISDYIYRIALAHPGIAFSLTHNGGSLLR 198

WP_045672515_1 TRGDGDRLQVIAAVYGTNTAKAMIPVQGDHPDYDLQGFISKPELTRANRNGITTIVNGRYIRSYAVQQALLQAYHTLLPINRFPLAVLEIGMHPGLVDVNVHPSKLEVRFSKEAELREFVEHVAGEALGKRRHIPGP-DA-GRAE-NSKPVFKQE-EIRFH---------------------RP---------------- 357

WP_016822099_1 TLGNGDLLQVIAAIYGTSAAKSMLMVEGESLDYRISGYVSRPEWTRSNRNAISTVVNGRFVRSYGLNQALLKAYHTLLPINRYPLAVIQLDMHPSLVDVNVHPAKLEVRFSKEAELFQLVEDSVKAVLGQQVLIPKA-VKREIGG-KDSGSFVQE-QFHFS---------------------KG--------NGAEVDAS 366

WP_102163696_1 TLGNGDLLQVIAAIYGTSAAKSMLMVEGESLDYRISGYVSRPEWTRSNRNAISTVVNGRFVRSYGLNQALLKAYHTLLPINRYPLAVIQLDMHPSLVDVNVHPAKLEVRFSKEAELFQLVEDSVKAVLGQQVLIPKA-VKREIGG-KDSSSFVQE-QFHFS---------------------KG--------NSAKVDTS 366

WP_013371549_1 TLGNGDLLQVIAAIYGTSAAKSMLMVEGESLDYRISGYVSRPEWTRSNRNAISTVVNGRFVRSYGLNQALLKAYHTLLPINRYPLAVIQLDMHPSLVDVNVHPAKLEVRFSKEAELFQLVEDSVKAVLGQQVLIPKA-VKREIGG-KDSGSFVQE-QFHFS---------------------KG--------NSAKVDTS 366

WP_074095505_1 TLGNGDLLQVVAAIYGTSAAKAMLPIQGESLDYRVSGLISLPEWTRANRNGMSTIVNGRFVRNYGLNQAILKAYHTLLPINRFPLVVVQLEMHPSLVDVNVHPAKLEVRFSKEPELYEFIETTLRGILRQEVLIPQV-KKQQIRR-GDDSSFIQE-QFLFP---------------------RG----------PLKDAS 364

WP_087435490_1 TIGNGDLLQVIAAVYGVHTAKGMMKVTAEHLDFELEGYIGKPEMTRSNRNAMSWFVNGRYVRSFPLNQAVLRAYHTLLPINRFPMVVLQTRMHPTLVDVNVHPAKLEVRFSKEAELCEFVESTLRELLLQQQLIPKA-APPK----AKVRTFVEQTELQWA---------------------SA------P-TVDANNEP 365

WP_023989046_1 TLGNGDLLQVIAAIYGTSAAKSMLLLEGESLDYRISGYVSRPEWTRSNRNAISTVVNGRFVRSYGLNQALLKAYHTLLPINRFPLAVIQLEMHPSLVDVNVHPAKLEVRFSKEAELFQLVEDSVKAVLGQQVLIPKA-VKREIGG-KDSGSFVQE-QFHFS---------------------KG--------NSVEGDAP 366

WP_053325620_1 TLGNGDLLQVIAAIYGTSAAKSMLLLEGESLDYRISGYVSRPEWTRSNRNAISTVVNGRFVRSYGLNQALLKAYHTLLPINRFPLAVIQLEMHPSLVDVNVHPAKLEVRFSKETELFQLVEDSVKAVLGQQVLIPKA-VKREIGG-KDSGSFVQE-QFHFS---------------------KG--------NSAEGDAS 366

WP_071641229_1 TLGNGDLLQVIAAIYGTSAAKSMLLLAGESLDYRISGYVSRPEWTRSNRNAISTVVNGRFVRSYGLNQALLKAYHTLLPINRFPLAVIQLEMHPSLVDVNVHPAKLEVRFSKEAELFQLVEDSVKAVLGQQVLIPKA-VKREIGG-KNSGSFVQE-QFHFS---------------------KG--------NGAEGDAP 366

WP_005549848_1 TIGNGDLLQVIASVYGVQTAKGMLHIKGEHLDFEVEGYIGKPELTRSNRNAMSWMINGRYVRSFPLNQAILRAYHTFLPINRYPMLVLHVNMHPSLVDVNVHPAKLEVRFSKEAELCAFIEETVKTLLHKQRLIPEA-APAK----AKVRTYVEQAELQWE---------------------AA------E-TIAANQEK 365

WP_068617384_1 SPGNGDLLQVIASIYGTQAAKAMLPIQAENPDYKVEGFIGRPDLTRSNRGGISTMINGRYIRNPGLQAAMMRAYHTLLPINRYPLAVLQLTMHPSLVDVNVHPSKLEVRFSKEQELYVFLEESIRAVLSQEVLIPHV-VKTTVNR-GETKSVIQE-QFNFQ--------------------------------------- 356

WP_094154817_1 TLGNGDLLQVIAAIYGTSAAKSMLPLEGESLDYRISGFVSRPEWTRSNRNAISTVVNGRFVRSYGLNQALLKAYHTLLPINRFPLAVIQLEMHPSLVDVNVHPAKLEVRFSKETELFQLVEDSIKAVLGQQVLIPKA-VKREIGG-KDSGSFVQE-QFHFS---------------------KGHDTGYDTGNRTEGAVS 374

WP_042177336_1 TLGNGDLLQVIAAVYGTSAAKAMLPVSAEDPDYKISGYISRPEWTRSNRNAVTTIVGGRYIRSNGLNAAIMRAYHTLLPINRYPLLVLELDMHPSLVDVNVHPAKLEVRFSKEPELYAFVEQELRKVLLGQNLIPRP-GRELVGG-KGSSSFIQE-QFAFT---------------------KG---------------- 358

WP_037288812_1 TLGGGDLLQVIAAVYGTTSAKAMIELQGESLDFAVSGFVSLPDFTRSNRSAITTVVNGRYIRSHVLNQAIMRAYHTLLPVGRHPLVVLRLVMHPSLVDVNVHPAKLEVRFSKEAELVAFVEDKIRAALSHEVLIPKA-VNQRIG---KNNSIVQE-QFHFP---------------------KR--------EGLLGDLA 364

WP_014281438_1 TLGNGDLLQVIAAVYGTSAAKSMLLLEGESLDYRISGYVSRPEWTRSNRNAISTVVNGRFVRSYGLNQALLKAYHTLLPINRFPLAVIQLEMHPSLVDVNVHPAKLEVRFSKEAELFQLVEDSIKAVLGQQVLIPKA-VKREIGG-KDSGSFVQE-QFHFS---------------------KG--------SGMEGNSS 366

WP_068700502_1 TQGNGDLLQVIASIYGNNAARSMLSISAEDPDYRITGYIGRPELTRSNRAGMSTIVNGRFIRNPALQQAILRAYHTLLPIGRYPLLVLELKMHPSLVDVNVHPAKLEVRFSKEAELTAFVEAAIKETLSQEVLIPQV-VKQKISR-GSSNSVIQE-QFHFS---------------------AP------S--------- 359

WP_010273601_1 TLGNGDLLQVIAAVYGTSAAKQMLAVSLETPDYTISGYISRPEWTRANRYGISTIINGRYIRNYGLVQALLKGYHTLLPINRYPLAVLHLEMSPELVDVNVHPAKLEVRFSKEPELLQSVEESVRAALAQEVLIPEG-RKPA----D-KKPVVRE-QIAWYGDEMFAAGQQGCEAGSAIAKESA---------------- 375

WP_076171200_1 TVGNGDLLQVVAAIYGRQAAKALLPIHAENLDFTIDGYVSRPDWTRSNRNGISIIVNGRYIRSYGLNQAIQRAYHTLLPINRYPLAVLEVKMHPSLVDVNVHPAKLEVRFSKEQELFPFVESSIHQVLRQEVLIPQP-VKQSIGD-KDKGSFIQE-QFHFS---------------------KS---------------- 358

WP_062328181_1 TLGNGDLLQVVAAIYGTSAAKAMLPIQGESLDYRVSGLISLPEWTRANRGGMSTIVNGRYIRNYGLNQAILKAYHTLLPINRFPLVVVQLEMHPSLVDVNVHPAKLEVRFSKEAELYEFIETTLRGILRKEVLIPQV-TKQQIRR-GDNSSFIQE-QFLFP---------------------RG----------PLKEET 364

WP_042228138_1 TLGGGDLLQVIAGVYGSAVGKQMIAVGGESLDYKISGYISRPELNRANRSAMSIMVNGRYVRSFAVQHAIMNGFHTLLPINRFPVVVLHIEMDPSLVDVNVHPSKLEVRFSKEPELVKMIEDMIKEALGRQVLIPKGVQASA----RIKESFVQE-TMTLY---------------------RP---------------- 356

WP_042137912_1 TLGNGDLLQVIAAVYGTSAAKAMLPVAAEDPDYKISGYISRPEWTRSNRNAVTTIVGGRYIRSNGLNAAIMRAYHTLLPINRYPLLVLQLDMHPSLVDVNVHPAKLEVRFSKETELYAFVEQQIRSLLMGQSLIPRP-TKEIIGG-KNSSSFIQE-QFAFS---------------------KG---------------- 358

WP_042236923_1 TLGNGDLLQVIAAVYGTSAAKAMLPVAAEDPDYKISGYISRPEWTRSNRNAVTTIVGGRYIRSNGLNASIMRAYHTLLPINRYPLLVLQLDMHPSLVDVNVHPAKLEVRFSKETELYAFVEQQIRSLLMGQSLIPRP-NKEIIGG-KNSSSFIQE-QFAFS---------------------KG---------------- 358

WP_042214549_1 TLGNGDLLQVIAAVYGTSAAKAMLPVAAEDPDYKISGYISRPEWTRSNRNAVTTIVGGRYIRSNGLNAAIMRAYHTLLPINRYPLLVLQLDMHPSLVDVNVHPAKLEVRFSKETELYAFVEQQIRSLLMGQSLIPRP-TKEIIGG-KNSSSFIQE-QFAFS---------------------KG---------------- 358

WP_038696521_1 TLGGGDLLQVIAAVYGTSAAKAMLPVEAEDPDYRISGYISRPEWTRANRNAITTIVGGRYIRSGGLNAAIMRAYHTLLPINRYPLLVLKLDMHPSLVDVNVHPAKLEVRFSKENELYQFVEQELRKVLLGENLIPRP-GRETIGP-KGSTSFIQE-QFSFT---------------------KP---------------- 358

WP_042128372_1 TLGNGDLLQVIAAVYGTSAAKAMLPITAEDLDYRISGYISRPEWTRSNRNAVTTIVGGRYIRSNGLNAAIMRAYHTLLPINRYPLLVLELDMHPSLVDVNVHPAKLEVRFSKENELYSFVEQEIRKVLLGQSLIPRP-GKETIGP-KGSSSFIQE-QFAFS---------------------KG---------------- 360

WP_038571591_1 TLGNGDLLQVIAAVYGTNAAKAMLPITAEDLDYRISGYISRPEWTRSNRNAITTIVGGRYIRSNGLNAAIMRAYHTLLPINRYPLLVLELDMHPSLVDVNVHPAKLEVRFSKENELYTFVEQEIRKILLGQSLIPRP-SKETIGP-KGSNSFIQE-QFAFS---------------------KG---------------- 358

WP_039873825_1 TLGNGDLLQVIAAVYGTSAAKAMLPVSAEDPDYKISGYISRPEWTRSNRNAVTTIVGGRYIRSNGLNAAIMRAYHTLLPINRYPLLVLKLDMHPSLVDVNVHPAKLEVRFSKEPELYAFVEQELRKVLLGQNLIPRP-GRETVGG-KGSSSFIQE-QFAFS---------------------KG---------------- 358

WP_079940587_1 TLGNGDLLQVIAAIYGTNLAKKMISLKGESLDYTVRGYISKPELTRANRSGISIFVNGRYVRSFPLNHAILSGYHTLLPINRFPLVVLEVGMDPGLVDVNVHPAKLEVRFSKEQDLTRWLEELIKETLGMQVLIPKA-DKPA----KIREQVVQE-ELHLA---------------------RA---------------- 355

WP_046504127_1 TLGNGDLLQVIAAVYGTSAAKAMLPLAAEDPDYRISGYVSRPEWTRSNRNAVTTIVGGRYIRSNGLNAAIMRAYHTLLPINRYPLLVLQLEMHPSLVDVNVHPAKLEVRFSKEAELYAFVEQEIRKVLMGQSLIPRP-SKEIIGA-KGSSSFIQE-QFAFS---------------------KG---------------- 358

WP_042266908_1 TLGNGDLLQVIAAVYGTSAAKAMLPLSAEDPDYRISGFVSRPEWTRSNRNAVTTIVGGRYIRSNGLNAAIMRAYHTLLPINRYPLLVLQLEMHPSLVDVNVHPAKLEVRFSKEAELYAFVEQEIRKVLMGQSLIPRP-GKEIIGA-KGSSSFIQE-QFAFP---------------------KG---------------- 358

WP_025335246_1 TLGNGDLLQVIAAVYGTSAAKAMLPVHAEDPDYRISGYVSRPDWTRSNRNAITTIVGGRNIRSNGLNAAIMRAYHTLLPINRYPLLVLKLDMHPSLVDVNVHPAKLEVRFSKEPELYQLVEQEIRKALLGENLIPRP-GRETIGP-KGSTSFIQE-QFAFP---------------------KA---------------- 358

WP_108465825_1 TPGNGDLLQVIAAIYGNNAARAMVPIEYEDPDYTLRGFIGRPDLTRSNRNAISTVVNGRYIRNTGLQQAILRGYHTLLPIGRYPLVVLQLTMHPSLVDVNVHPAKLEVRFSKEGELNTFVETAVKGALSQHVLIPQV-VKQQIGK-GDSRSVIQE-QFSFP---------------------SP------S--------- 359

WP_038593031_1 TLGNGDLLQVIAAVYGTSAAKAMLPISAEDPDYRITGFVSRPEWTRSNRNAVTTIVGGRYIRSNGLNAAIMRAYHTLLPINRYPLLVLQLDMHPSLVDVNVHPAKLEVRFSKEIELYAFVEQEIRKVLLGQSLIPRP-GKEIIGA-KGSSSFIQE-QFAFP---------------------KG---------------- 358

WP_081951145_1 TLGNGDLLQVIAAVYGTSAAKAMLPITAEDLDYRISGYISRPEWTRSNRNAVTTIVGGRYIRSNGLNAAIMRAYHTLLPINRYPLLVLELDMHPSLVDVNVHPAKLEVRFSKENELYTFVEQEIRKVLLGQSLIPRP-GKETIGP-KGSSSFIQE-QFAFS---------------------KG---------------- 358

WP_068648544_1 TVGNGDLLQVIAAIYGTQAAKAMLPIQAEDMDYSLQGYVSIPEWTRSNRNAVSTFVNGRYIRNNGLNHAILRAYHTLLPINRYPLAILSLTMHPSIVDVNVHPAKLEVRFSKENELYPFVEQAIHAVLRQQVLIPQP-VKHPIGN-TGSSSFIQE-QFRFN---------------------KD---------------- 358

WP_036656101_1 TLGKGDLLQVIAAVYGTSAAKAMLPITAEDPDYRISGYISRPDWTRSNRNAVTTIVGGRYIRSNGLNAAVMRAYHTLLPINRYPLLVMKLDMHPSLVDVNVHPAKLEVRFSKEVELYKFVEEETRKVLLGQTLIPRP-GRETIGP-KGSSSFIQE-QFAFQ---------------------KG---------------- 358

WP_015734656_1 TLGNGDALQVIAAVYGTQSAKAMLPFAAENMDYTLSGYISRPDYTRANRNGMSLIINGRYIRNYGLMQAILRGYHTLLPINRFPLVVIQLSMHPSLIDVNVHPSKLEVRFSKEQELFAFVEEEVRKVLQQEILIPRP-AKQNIG--KSNNAYIQE-QLSFP---------------------QA---------------- 357

WP_016312025_1 SPGGGDLLQVIAAIYGVNASKGMVPIEAEDLDYRVTGYIGRPDLARSSRSGMSTIVNGRYIRNQGLHQAILRAYHTLLPINRYPLVVLMLEMHPSLVDVNVHPSKLEVRFSKEPELNSFVENSVRAVLSGQVLIPQV-VRQTIGK-GPNRSVIQE-QFHFP---------------------AP------P--------- 359

WP_087920285_1 TLGNGDLLQVIAAVYGTSAAKAMLPVAAEDPDYRISGYISRPEWTRSNRNAITTIVGGRYIRSNGLNAAILRAYHTLLPINRFPLLVLDLDMHPSLVDVNVHPAKLEVRFSKEIELYAFVEDQLRKVLLGQSLIPRP-VKETLGP-KGSSSFIQE-QFAFS---------------------KG---------------- 358

WP_025694959_1 TLGNGDLLQVIAAVYGTSAAKAMLPVQAEDPDYRISGLVSRPEWTRSNRNAVTTIVGGRYIRSSGLNAAIMRAYHTLLPINRYPLLVLKLDMHPSLVDVNVHPAKLEVRFSKETELYQFVEQEIRKVLLGENLIPRP-GREPIGP-KGSTSFIQG-QFAFP---------------------KA---------------- 358

WP_042207003_1 TLGNGDLLQVIAAVYGTSAAKAMLPVQAEDPDYRISGLVSRPEWTRSNRNAVTTIVGGRYIRSNGLNAAIMRAYHTLLPINRYPLLVLKLDMHPSLVDVNVHPAKLEVRFSKETELYQFVEQEIRKVLLGENLIPRP-GRETIGP-KGSTSFIQE-QFAFP---------------------KA---------------- 358

WP_027084813_1 TPGNGDLRQVVAAIYGTASAKATIALSAEHPDYALTGVTALPSETRANRNAVTVLVNGRYVRSQAVMQPLLQAYHTLLPLHRYPLAVISLTMHPTLVDVNVHPAKLEVRFSKESELRGFVEQAVKEALGGKTHIPSG-AN--TRA-AKQPSWVQE-QIRFQ---------------------LP---PSEA--------- 360

WP_044876703_1 TLGNGDLLQVIAAIYGTQAAKAMLPIQTEDLDFNIQGFISRPEWTRSNRNAISIIVNGRYIRNYGLNQAILRAYHTLLPINRYPMGILALNMHPSLVDVNVHPAKLEVRFSKEAELYPFVEQAVHAVLRQEVLIPQP-VKTNIGS-S-DSSFIQE-QFRFS---------------------KA---------------- 357

WP_099476734_1 TLGNGDALQVIAAIYGTQSAKAMMKIEAENMDYTLHGYISRPDFTRANRNGMSLIVNGRYIRNYGLMQAVLKGYHTLLPINRYPLVVLQLSMHPSLIDVNVHPSKLEVRFSKEPELFSFVEEAVRNVLRQEILIPRP-AKQTIG--KSDNAFIQE-QLNFP---------------------AP---------------- 357

WP_036637535_1 TLGNGDALQVIAAIYGTQSAKAMLAFEAENMDYTLSGYISRPDYTRANRNGMSLIINGRYVRNYGLMQAVLRGYHTLLPINRFPLVVIQLTMHPSLIDVNVHPSKLEVRFSKEQELFAFVEEEIRKVLQQEILIPRP-AKQAIG--KSEHAFIQE-QLQFP---------------------NA---------------- 357

WP_016362589_1 TLGSGDLLQAVAAVYGSSLAKSMLKIEGETLDYKLEGLISKPELTRANRGGITTVINGRYIRSFPVNQALLQGYHTLLPIGRFPVAVLHLTMDPTLVDVNVHPAKLEVRFSKEPELIALIENAVRETLGRQRLIPQG-GAPAAPA-RVREQLVQE-QLELY---------------------RP---------------- 358

WP_068658264_1 TIGNGDLLQVVAAIYGTQAAKAMIPIQAEDLDFSMQGFVSLPEWTRSNRNGISTIVNGRYIRSNGLNHALLRAYHTLLPINRYPLVILSLTMHPSIVDVNVHPAKLEVRFSKESELYPFVEQAIHAVLRQEVLIPQP-VKHPIGN-N-TSSFIQE-QFRFT---------------------KD---------------- 357

WP_062408748_1 TPGGGDLLQTIASIYGTAVAKSMLPVYGETLDYRLEGFIAKPEVTRANRGGMTTVINGRYIRNYPMVQALLQGYHTLLPIGRFPVAVLHVQMDPTLIDVNVHPAKLEVRFSKEPELMQLIERETRETLGRQRLIPSA-GSPGGAGKRLREPLVQE-QLELY---------------------RP---------------- 359

WP_042161919_1 TLGTGDRLQVIAAVYGTNTAKSMLSVEGENPDYDLRGYISKPELTRANRNGMTVIVNGRYIRSHAVNQAILQAYHTLLPINRFPLVVLELGMHPSLLDVNVHPSKMEVRFSKEAELRTLIEQSVKAALGQYRYIPGP-EASLKQE-RSKPLFVQD-AISFH---------------------RP--LPEDA--------- 363

WP_068609064_1 TLGNGELLQVIAAVYGKSSAKTMIRVNAESLDYTLSGYISAPELTRSNRNGITTVVNGRYVRNYALVNAMLRAYHTLLPINRFPLAVLELRMHPTLVDVNVHPSKLEVRFSKEAELSQFLEATLAQALNRQVLIPAG-AYPATQA-AKPAAVVQE-QLQLY---------------------RP---------------- 358

WP_046232528_1 TLGTGDRLQVIAAIYGSNTAKAMMAIQGESPDYELLGYISKPELTRSNRNGITVIVNGRYIRSHAVNQALLQAYHTLLPINRFPLAIIEIGMHPSLLDVNVHPSKMEVRFSKEAELRTLVESAIQAALGKERHIPGP-DSIHNQK-RSGPVFVQG-AMSFH---------------------RP---------------- 358

WP_099520158_1 TLGTGDRLQVIAAIYGSNTAKAMMAIQGESPDYELLGYISKPELTRSNRNGITVIVNGRYIRSHAVNQAMLQAYHTLLPINRFPLAIIEIGMHPSLLDVNVHPSKMEVRFSKEAELRTLVETAIQAALGKERHIPGP-DSIHNQK-RSGPVFVQG-AMSFH---------------------RP---------------- 358

WP_015844205_1 TLGTGDRLQVIAAVYGTNTAKAMLKVEGETSDYDLRGYISKPELTRANRNGMTVIVNGRYIRSHAVNQAILQAYHTLLPINRFPIVVLEIGMHPSLLDVNVHPSKMEVRFSKENELRTLVEQAVKGALGQVRHIPGP-AE--KPE-REKPLFVQD-AISFH---------------------RP---------------- 356

WP_091189189_1 TLGTGDRLQVIAAVYGTNTAKAMLKVEGETSDYDLRGYISKPELTRANRNGMTVVVNGRYIRSHAVNQAILQAYHTLLPINRFPIVVLEIGMHPSLLDVNVHPSKMEVRFSKENELRTLVEEAVKEALGQVRHIPGP-AE--K---REKPLFVQD-AISFH---------------------RP---------------- 354

WP_006036759_1 TPGTGDRLQVIAAVYGTSTAKAMLPVEGETPDYVLTGYVSKPELTRANRNAITVIVNGRYIRSFLVNQALLQGYHTLLPINRFPLVTLEIAMHPSLLDVNVHPSKMEVRFSKETELRELIEQSVKKTLGRDRYIPEP-AASGDQE-RSKPVYVQE-RIAFH---------------------RP---------------- 358

WP_062492889_1 TTGGGDRLQVIAAIYGTNTAKAMLKVSAEHPDYDLNGYISKPELTRANRNGLTVIVNGRTIRSYIVNQAVIQAYHTLLPINRYPLAVLEIGMHPSLIDVNVHPSKMEVRFSKEAELREFVEHAVRDALSGIRHIPGP-DA-GARD-RSKPVYVQG-RIAFH---------------------RA---------------- 357

WP_015254643_1 TTGGGDRLQVIAAIYGAAAARQMLRAAADHPDYRLEGWLSKPELTRANRSGITTIVNGRYIRSFAIQQAILEAYHTLLPTGRFPLVVLEVGMHPGLLDVNVHPAKQEVRFSKEAELKELVESALRRALGRTAHIPSA-PA----A-GMKPAVIQE-RLSFP---------------------PA---------------- 354

WP_089523565_1 TSGSGDRLQVIAAVYGTGTAKAMLPLRGEHADYELNGYISKPELTRASRTAITTVVNGRYIRSFAVQQSLLTAFHTLLPINRFPVAVLEIGMHPGLVDVNVHPSKMEVRFSKEAELKAFIEETARAALGGRRHIPGP-DS-GRGT-RSGPVYRQE-QLRFH---------------------QP---------------- 357

WP_045672515_1 -----------------ES-------------A-FAP-DRAN-AGGT-----GR-------KPA---EP-----------------------------------------------------GW--A----------------P-----KGYRNDRR------DDAP---------------ASPP-------------I 399

WP_016822099_1 SDNGHNRTLPLSM-A--SP---------QVQ---EST---------------QS-NHL-T-SNE-----------------------E-KKKISQ----EREQHP-------SRERSHS-------VN------GQ----A-NT-LA--RQSISDDE----L--------------------DADF-------------V 440

WP_102163696_1 ADN---------I-S--SN---------ESN---ESN---------------ES-NE----SNE-----------------------E-SKKFSH----EHEQLP-------NRG-SHS-------VI------GQ----A-NT-LA--RQSISDDE----L--------------------DADF-------------V 428

WP_013371549_1 TDN---------I-S----------------------------------------------SNE-----------------------E-SKKFSY----EHEQLP-------NRG-SHS-------VI------GQ----A-NT-LA--RQSISDDE----L--------------------DADF-------------V 416

WP_074095505_1 DAEGYGQQGPLGKPT--AE---------PLK---LAT---------------ED-DDL-D-LDA-----------------------P-ADVTTG----ESVSEQ-------GQ--SVP-------LP------EA----P-PE-IT--HPPVQLES----W--------------------NGDILQKVASHDGGQTSV 450

WP_087435490_1 QLKPMGALI-----P--SP-----GS--NGE-S-SPP---------------AT-------NVP-----------------------E-AKAGAA----AAQEAS--G-Y----------------AV---DM-NI--Q-A-ET-KA--AAVAPRVE-R------------------------------------ESA-V 431

WP_023989046_1 SGKDQGGALPLSV-T--SP---------ESQ---VPR---------------QS-NYL-N-RNE-----------------------E-NNRGSQ----EREQRP-------SRD-SLS-------VI------GR----A-NN-LS--GKSISDDE----L--------------------DADF-------------V 439

WP_053325620_1 SGKDRGGALPLSV-T--SP---------ESQ---VPR---------------QS-NYL-N-RNE-----------------------E-NNRGSQ----EREQRP-------SRD-SLS-------AI------GR----A-NN-LS--GKSISDDE----L--------------------DADF-------------V 439

WP_071641229_1 SGKDRGGALPLSV-T--SP---------ESQ---VPR---------------QS-NYL-N-RNE-----------------------E-NNRGSQ----EREQRP-------SRD-SLS-------AI------GR----A-NN-LS--GKSISDDE----L--------------------DADF-------------I 439

WP_005549848_1 QLKPMGALV-----I--AS-----DS--SSD-S-SSAKEFFQNSANETRISNQS-------IVN-----------------------R-SQESAE----NREQNK--G-ND-SSG-IAS-NDSES-SI---DS-SS--Q-R-RA-VV--RELANRPE-RL-V-EGSE------AKILLQSEMSADA---TTNATTNAS-T 483

WP_068617384_1 ------------------------ST--------ATP---------------AA-------VSD-----------------------S-GHEAEKRP--EHAGYP--E----RPG-GSP-G-------------GA----H-EG-----AASPQNDR----V--------------------REAA-------------A 408

WP_094154817_1 SGEDRGLAEPLSV-A--SL---------KGQ---DPR---------------QS-NRL-N-GNE-----------------------E-NRRVSS----EREQRT-------RRE-GVT-------AI------DR----A-SA-LS--GTSVSDEE----L--------------------NADF-------------V 447

WP_042177336_1 --------------N--VP---------------APQ---------------E--SGA---NAP-----------------------A-AVPGAE----ASAALPGLNASAAAPG-ADASSSAAADTKLPGSVSGS----S-TQ-----QPMTPSGS----T-QSPP------GAAASGYTPPAVS-------------P 444

WP_037288812_1 SG------LPQDK-D--VP---------SAT---SES---------------QS-AAT---HSE-----------------------Q-ASQGNA----ASQQNA-------AAS-ADK---NK----------ES----S-DS-----PDSRPKGA-A--V--------------------RADK-------------V 429

WP_014281438_1 AGESRGEAQLLSG-S--YS---------EGR---DPR---------------QS-IRL-D-GNE-----------------------E-SSRLGT----ERKQRQ-------DRE-RLP-------DS------GR----G-NP-SS--GQRVSDEE----L--------------------EADF-------------V 439

WP_068700502_1 --------------A--AP---FGDS--------ASE---------------AG-------RTR-----------------------P-GQAG-----IDGAAEP--D----RPG-SGS-A-------------GEPFPGG-QG-TP--PESEAAPD----L--------------------TGEA-------------A 419

WP_010273601_1 --------------K--DD---LGTG--------EPG---------------QT-------ASS-----------------------G-EARAQA----GVPQAG-------VPQ-AGV---------------PA----A-AG-RP--DAARDQQR-------------------------TPRQ-------------- 428

WP_076171200_1 --------------P--APQAGPMDGLMDGGEAPLPQ---------------DP-------GPA-----------------------GHTANSVA----DGSLTP-------VSA-SGS-------LP-------V----K-SP-----LAAGPEGAGTGFS--------------GPRAQAPAQQ-------------G 434

WP_062328181_1 ESAGYGQQGPLGK-H--AE---------PGK---MAS---------------ED-DDL-D-LDA-----------------------P-ADLQSG----TDRNME-------GQ--TPP-------LP------EA----P-PE-IP--PSYDPFGM----L--------------------SGDL---------GSNVL 440

WP_042228138_1 --------------E--AD-PLLKQGI-------TPA---------------QW-------REQ-----------------------L-SVPEDG----QAPASA-------DNR-EGA---------------GE----G-RD-TA--QEADERRR-------------------------SLAS-------------- 412

WP_042137912_1 --------------A--VP---------------GAP---------------QQ-------GAE-----------------------P-ANPADA----SAGRGP-------GQG-MPA---DA--LQ------G-------GS-----SQPRPGGN----S---------------AAPFPGGAA-------------P 414

WP_042236923_1 --------------S--VP---------------GAP---------------QQ-------GAE-----------------------P-ANPAEA----SAGRGP-------IQG-MPA---DADALQ------G-------GS-----SQPRPGGN----S---------------A-------A-------------P 409

WP_042214549_1 --------------A--VP---------------GAP---------------QQ-------GTD-----------------------A-ANPADA----SAPRIP-------GSG-LSA---DA--LR------GA----G-GI-----SQPRPGGS----S---------------D---------------------- 407

WP_038696521_1 --------------A--SQ---------------TAE---------------TA-QDAVNKGAN-----------------------G-EGRLFG----PQGAQG--D----PAG-PAA---GY--LG--------------GSHQELPGNLGVNGS----P--------------------SGAS-------------F 420

WP_042128372_1 --------------T--VP---------------PPP---------------SV--DG---GKD-----------------------S-VPQGAG----SSFSRQ--Q----TSN-VEL-----------------------NA-----REFRQEGL----T-------------------------------------- 404

WP_038571591_1 --------------A--VP---------------PSS---------------ST--GD---VKD-----------------------T-APTGSA----NFLSGL--Q----SSS-LDR-----------------------NT-----RDFSKEGQ----A-------------------------------------- 402

WP_039873825_1 --------------T--AP---------------APQ---------------E--PGA---DSL-----------------------A-AVSGAN----ATAASD--TNAPAAPG-ANA----------PAAAAGG----S-NP-----QPLAPSGS----T-QSPP-------VPAGSYAPPGAA-------------P 431

WP_079940587_1 --------------K--EE---------------TAP---------------AF-------QGQ-----------------------T-KFKEDK----PLQSAP-------VDK-EIF---------------ST----SVRY-QA--EDMHEKSR-------------------------ISES-P-----------L 407

WP_046504127_1 --------------A--VP---------------PPP---------------QE-EAL---GRS-----------------------A-AEPGYP----QTGQAP-------APG-GAV------------PVSGA----G-GS-----GPLRSPGA----L-SGLT------------LPPLDGG-------------R 420

WP_042266908_1 --------------A--VP---------------PPP---------------QE-EAL---GRS-----------------------S-AEPGYP----RTGPAP-------APG-GA----------------GA----G-SS-----EPPRSPGP----L-SSLP------------QQSLDGV-------------R 416

WP_025335246_1 --------------S--PK---------------FNG---------------SI-DEV---KEQ-----------------------G-AGLKVS----QETGAGADE----SST-NAA---DV--TL--------------ST-----DLTEQPGA----W--------------------RGPN-------------P 414

WP_108465825_1 --------------Q--AP---V-DT--------ANR---------------QE-------FIH-----------------------S-GNSAPV------SGSP--S----REG-STA-G-------------GQ----G-QG--M--DVPSVSSS----M--------------------SQV--------------- 410

WP_038593031_1 --------------A--IP---------------QPA---------------DGLPGM---ARN-----------------------D-QDGPDP----RTA----------GPQ-SDM------------PS-GV----A-PQ-----SDLQQAGT----SDANRP------------VERPDAW-------------N 418

WP_081951145_1 --------------S--VP---------------APP---------------PS--EV---GKD-----------------------P-VPQGSG----SSFSRQ--Q----PSN-VEQ-----------------------SA-----REIRKEGQ----I-------------------------------------- 402

WP_068648544_1 --------------Q--QT----------------------------------------------------------------------ANTASS----NQSEKS-------LSD-SSK-------LVQS----DM----I-GS-----QLSPLTGS------------------------------------------- 396

WP_036656101_1 --------------S--KE---------------ALK---------------ES-PIE---QLE-----------------------R-AKYTDS----NLNKGL-------MTG-SPA-----------------------FS-----QQEKQSER----E-------------------------------------- 402

WP_015734656_1 --------------Q--GD---------------VGA---------------AR-------GGQ-----------------------D-RDLEIP----GSDERS-------AGG-NTP-------LP-T---------------PP--AAWPDKQT----L-------------------------------------- 401

WP_016312025_1 --------------L--GA---E-EG--------ASA---------------AG-------HAA-----------------------A-AAESPLRPPVTGGGAE--R----ATG-ARS-A-------------GA----D-RG-WA--GAPASGAP----M--------------------SGAP-------------S 419

WP_087920285_1 --------------S--PP---------------LPG---------------KE--QA---AAD-----------------------P-AAVQSL----GVQQGP-------LPA-GGF-T-------------GS----G-DN-----TAARPAGT------------------------------------------- 404

WP_025694959_1 --------------A--PN---------------SAA---------------AG-GVP---KTS-----------------------P-EQLGFT----AKSGAG--E----TPG-IRE---GQ--MV--------------PE-----SGQGQGGA----S--------------------RGQS-------------P 412

WP_042207003_1 --------------S--PN---------------TAA---------------AD-GIP---KTS-----------------------P-DNFGFT----AKAGAG--E----TLG-NRE---GR--MV--------------PE-----GGPGEAGA----R--------------------SGQN-------------P 412

WP_027084813_1 --------------T--EA-------------T-EAS-R-------------GS-------EAS--------------------------------------------------------------------------A---NA-----PDFAREAA------AAVE-----------------NS-------------L 391

WP_044876703_1 --------------P--SV----------------------------------------------------------------------IDHSDA----NRADS---------NH-TNK-------LE-Q----GI----A-NP-----LVVSKDGV------------------------------------------- 392

WP_099476734_1 --------------S--YE---------------APS---------------RG-------ERA-----------------------G-MPQESL----GGRSQA-------ETA-GSD-------FR-R-------------------ESSPKPDS----L-------------------------------------- 399

WP_036637535_1 --------------Q--GR---------------DAS---------------VG-------SRE-----------------------D-GEAPAA----RSGDQS-------DQG-MTS-------FP-I---------------PPG-SAWSEKRD----H-------------------------------------- 402

WP_016362589_1 --------------Q--AA---------------SAA---------------EP-------AAV-----------------------P-PAAVRS----EAPAAS-------PGG-SPF---------------GG----G-----P--RGAGAPAP-------------------------AAQP-Q-----------A 406

WP_068658264_1 --------------Q--PT----------------------------------------------------------------------PNDGLS----SQSNSQ-------RNW-NDN-------IP------------------------------------------------------------------------ 380

WP_062408748_1 --------------Q--AP---------------AAA---------------ER-------LT-----------------------------------------------------GTV---------------GQ----A-----P--R-------------------------------------------------- 377

WP_042161919_1 --------------AAAAP-------------S-ALP-T-------------GQ-------QAQLQSQPQSQPQLESKSQLELPSQSE-SQLQQS----GSQSVP-------HSQ-PQW-QPSR--SA------QE-SS---NG-----GGMGRNSY------TGYK-DQP-----------T-EY-------------V 451

WP_068609064_1 --------------D--GA---------------AGT---------------AG-------AGA-----------------------A-GTLPSW----DEVRLA-------PA--APF---------------GG----G-----F--A-------------------------------------------------- 392

WP_046232528_1 -----------------EP-------------K-TPP-V-------------PE-------KDS--------------------------------------------------------------------------K---GE-----RSIAQAPY------SPFG-QGA-----------SREF-------------V 393

WP_099520158_1 -----------------EP-------------K-TPP-V-------------PE-------KDN--------------------------------------------------------------------------I---GE-----RSIAQAPY------SPFG-QGA-----------SREF-------------V 393

WP_015844205_1 -----------------DP-------------L-SMP-W-------------GQ-------ERS-------------------PS--N-SNGSGA----QDSSSH-------LPG-GSV-NPGR--PA------GL-ST---SA-----ANTGSSFN------SSFP-QPP-----------KRDY-------------V 421

WP_091189189_1 -----------------DP-------------Q-TMP-W-------------GQ-------ERS-------------------SSAGN-SDAGAT----SGSASY-------SSG-SSF-TANR--PA------G--------T-----SNSGASYN------SAFP-QPP-----------KRDF-------------V 417

WP_006036759_1 -----------------EP-------------S-FPA-S-------------AR-------GGE-------------------TA--S-SFAGFG----AQSKAA-------SSG-SSS-SVGA--AP------R---T---GS-----GSYASAWP------TGVPGRNV-----------VRES-------------I 422

WP_062492889_1 -----------------ED-------------G-FGD---------------GP-------RTY---AP-----------------------------------------------------GA--A---------------GA-----AGSRPDSR------N-----------------------------------I 385

WP_015254643_1 -----------------PP-------------D-RPA-V----------------------------KP---------------------------------------------------------------------P---SA-----GGIA----------------------------------------------V 371

WP_089523565_1 -----------------EP-------------G-FGA-AGAGLPGGSAPAG-GE-------PPA---VP--------------PGGAA-TLPPAA----LAAAGE-------AAA-ASA-AAGF--AA------PQ-QG---GP-----RGYAGSGR------KGPP-PGSIAAGPWRPAAQGAPP-------------V 449

WP_045672515_1 PRDAAERL---Y----G-------RVQP------E-GALFRE--------AGADW---------TAGR--LP-AR----------------AADANA------------------------------E-TVA--A-E-----TA--AAS-A-AA-S-PL-QA-APYP---DADEKKNA--------AD------SS---A 475

WP_016822099_1 EENNGSDD---R----G-------SVSL-P----D-GMTTRI----------------------------ST-GD----------------HAETDV--------------------------S---S-SYS--E-G-I-----------Q-ET-A----AS-AAYR-S-SV--SPLA----------------DN---- 499

WP_102163696_1 EENNEGDV---R----G-------GVSL-P----D-GTTTRI----------------------------ST-GD----------------HAETDV--------------------------S---S-SYS--E-G-I-----------Q-ET-A----AS-ATYR-P-AV--SPIA----------------DN---- 487

WP_013371549_1 EENNEGDV---R----G-------GVSL-P----D-GTTTRI----------------------------ST-GD----------------HAETDV--------------------------S---S-SYS--E-G-I-----------Q-ET-A----AS-ATYR-P-AV--SPIA----------------DN---- 475

WP_074095505_1 NVQEGTKD---V----S-------TSS-------S-TSTSST----------------------------ET-VP----------------KSDLSS--------------------------Q---D-GGD--R-G-A-----------T-EK-PLAEGKP-ATYR-S-ESVNSPVR----------------EA---- 513

WP_087435490_1 PNNSAIPV---Q----P-------HEAV-E----S-RGVVPH----------------------------DA-GK-Q--QI----------AATRQS---E----------------------P---P-RLP--V-D-TGA-RA--SYV-H-EP-K-------PRYG-E----REERG--------FR------DR-V-- 501

WP_023989046_1 EENSGSEV---R----G-------DVPL-P----D-GATVQS----------------------------SE-GD----------------NAAIGT--------------------------S---S-LYS--G-G-V-----------Q-ET-A----AS-AAYP-S-SM--PPIA----------------GH---- 498

WP_053325620_1 EENSGSEV---R----G-------DVPL-P----D-GVTVQS----------------------------SE-GD----------------NAAIGT--------------------------S---S-LYS--G-G-I-----------Q-ET-A----AS-AAYP-S-SM--PPIV----------------GH---- 498

WP_071641229_1 EENNGSEV---R----G-------DVPL-P----D-GATVQS----------------------------SE-GD----------------NAAIGT--------------------------S---S-LYS--G-G-I-----------Q-ET-A----AS-AAYP-S-SM--PPIA----------------GH---- 498

WP_005549848_1 DANSDAAI---Q----PEASAIGNEDAV-D----R-PEVHQQ----------------------------AA-DH-G-VRF----------SVPDES---A----------------------S---S-QLS--A-N-RIE-SG--ESI-R-EP-S-------TSYA-G----RSAGA--------SD------SK-A-- 561

WP_068617384_1 TPSYSLPE---R----N-------GGSE------D-GLPAAG----------------------------PN-GD----------------TSTQQI--------------------------S---Y-AQT--P-G-NTD-QR--KIG-D-EA-R----PV-GNSP-A-----GTAG----------------GS-S-S 473

WP_094154817_1 DENNGS----------G-------GMSL-PGAHRQ-SSGAQE----------------------------VQ-GD----------------EAAVGT--------------------------S---S-VYS--E-A-A-----------Q-ET-A----AS-AAYR-S-SM--APSA----------------GN---- 507

WP_042177336_1 GGASSAPL---F----S-------S-NA------G-SALRPG----------------------------QT-Q-----------------PQARES--------------------------A---A-AYS--G-S-FSQ-AP--ARP-R-PE-V----GA-GSYR-P-----QPAA----------------MP---- 505

WP_037288812_1 PEALKASI----------------SYPA------I-GGAQDS----------------------------RP-QQ----------------VREPGH--------------------------G---L-GYT----G-------------R-SA-S-S--GG-SNPR-Q-----AAQP----------------AQ---- 482

WP_014281438_1 DVN--------------------------------------------------------------------D-GN----------------KAESGS--------------------------P---P-LYS--G-G-V-----------Q-EA-A----AS-VAYR-P-SA--SLDA----------------GN---- 478

WP_068700502_1 QAGLAPPGDKGY----A-------GNAQ----S-A-SLNEQV----------------------------RE-QA----------------AAAQAY--------------------------PYPDY-AQL--D-G-YGN-PD--SAY-R-SQ-Q----PA-GQSA-V-----RPAG----------------GT-VQN 492

WP_010273601_1 AESSASSA---P----G-------AGQA------PLTPPNAL----------------------------PP-EV-G-------VRRSG--PPAGSSSAPEPEASLPSDASGQGGGAGGRGDRP---A-AAG--R---APL-D---------TF-A-SR-GA-GPAR-EARGYSPRDSRRGMSA----------AS---- 531

WP_076171200_1 YSGQSSPN---A----G-------SVRNPG--E-A-AYAQQK----------------------------LP-YS---ARE----------TVRENT--------------------------V---P-GYR----L-NLS-QS--GR--V-SQ-S----SN-PALA--------------------------------- 494

WP_062328181_1 KQNYSPDE---N----S-------HSD-------P-ASPVSE----------------------------ST-GD----------------AADHAS--------------------------Q---D-AIP--S-A-E-----------D-RR-P----SA-SAYR-S-NSVDSPVR----------------ER---- 499

WP_042228138_1 LDSLAPPE---S----G-------AGSA------G-SAAEAL----------------------------APDGD-G-------RREQQ--PLAAGG-----AAGLSGRGAADDRRTGSASDAA---V-PDG--V---PAP-AA------QEAA-A-SR-LR-EPQS-RPAAAEPYGAPYGSGAYGSQPRSGAAAG---- 523

WP_042137912_1 LTGSSAPL---F----G-------GGAA------A-GAPQRY----------------------------PS-GG-------N-SLPPQ--SQTRET--------------------------A---A-AYG--G-G-TMP-PA--PYR-N-GT-A----AQ-PSSR-PRDSYRPQAA----------------AA---- 488

WP_042236923_1 LTGSSGPL---F----G-------GGAA------A-GAPQRY----------------------------PS-GG-------N-SLPPQ--SQTRET--------------------------A---A-AYG--G-G-IIP-PA--PYR-N-GT-A----AQ-PSSR-PRDSYRPQAA----------------AA---- 483

WP_042214549_1 -----APL---F----G-------GGAA------A-GAPQRY----------------------------PS-GG-------I-PLPQQ--PQARET--------------------------A---A-AYG--G-G-TPL-PA--PYR-S-EA-A----GQ-PSSR-PRDSYRPQAA----------------AA---- 476

WP_038696521_1 GSAPGRQA---A----G-------SPAAGG----P-SGLLES----------------------------AA-ASSGTAAFQT--------REGGSS--------------------------G---A-PYS--SSG-GNS-AS--RVR-D-AS-A----FG-AGYRAS----GPAAA----------------GR---- 496

WP_042128372_1 ---------------------------------------------------------------------------------------DH--PMTREA--------------------------A---P-SYG--S-G-YQP-SS--SQG-R-QN-N----VS-NNYT-N--SYHPQET----------------AS---- 447

WP_038571591_1 ---------------------------------------------------------------------------------------DH--QVAREV--------------------------A---P-AYG--S-G-YPS-SS--SQG-R-QT-S----AN-TSYS-N--SYRPQET----------------SM---- 445

WP_039873825_1 GGSSSAPL---F----S-------SGSA------G-AALRPG----------------------------QA-Q-----------------PQARES--------------------------A---A-AYS--G-S-FSQ-AP--ARP-R-TD-G----GA-GGYR-P-----PAAA----------------IP---- 493

WP_079940587_1 LESYADPN---Q----F-------RGWE------S-SPEVGN----------------------------SP-GA-G-------SR-SK--PSGYHS-----DYGF-------NHGSGYRSDYR---M-PHG--N---KQP-SV------Q-SV-K-SL-LP-EPKE-E-------------------------TG---- 483

WP_046504127_1 SGGNDAPL---Y----G-------AAAA------G-AAPYSS----------------------------SS-GL-------S--------SQSRER--------------------------A---A-AYS--G-S-APQ-AA--AQR-R-EG-A----GS-ESYR-Q-----FPSV----------------NP---- 484

WP_042266908_1 SGGNGAPL---P----G-------AAAG------E-AAPYSS----------------------------SS-GL-------N--------PQSRER--------------------------A---A-AYS--G-S-APQ-AA--AQR-R-EG-A----GS-ESYR-Q-----YPPA----------------NS---- 480

WP_025335246_1 RSVTSDAA---S----E-------AAASRA--YSP-SIPYDD----------------------------RS-SG-------K--------TRGGER--------------------------S---T-SYA--G-G-SFQ-P---QLR-D-GA-A----Y--GGYR-T----GSGTG----------------PR---- 481

WP_108465825_1 ---------------------------------------RSP----------------------------QV-RE----------------GAAAGY--------------------------A---A-GAG--A-G--------------------------GSYN-Q-----KGWQ----------------NE-V-- 442

WP_038593031_1 SGISSASP---F----G-------NGAA------A-GERYSG----------------------------NA-QQ-------RTGEPQSAMPVARES--------------------------A---A-AYS--G-S-TSS-LP------R-EG-A----GG--SYR-Q-----PAGA----------------MP---- 486

WP_081951145_1 ---------------------------------------------------------------------------------------DT--PMTRET--------------------------A---P-SYG--S-G-YQS-SS--SQG-R-QN-N----VS-NNYA-N--GYRPQET----------------SS---- 445

WP_068648544_1 ----------------------------------V-NYAMND----------------------------VS-SG---VNL----------PVRERN--------------------------D---Q-NYR----I-NGA-KG--NF--P-EE-G----KG-APFN--------------------------------- 439

WP_036656101_1 -------------------------------------------------------------------------------------------PFKSE---------------------------------PYK------PSQ-AA--SQI-R-EA-G----KN-NSYR-A-----PQST----------------YN---- 435

WP_015734656_1 ----------------------------------------------------------------------------------A--------TSPNCI--------------------------K---R-SYG--D-G-TAE-TAAASQM-R-ET-A----AS-SSYR-S----DYRNE----------------AR---- 443

WP_016312025_1 AAGAPVDG--------------------------G-ALPRSA----------------------------AY-PP----------------AAADGL--------------------------TLRER-GAG--A-GTYGA-AP--SSA-K-PQ-G----RL-GQPN-A-----ATYG----------------GG-E-- 481

WP_087920285_1 ------PL---P----G-------AYPA------A-GETRAA----------------------------AG-A-----------------GGTREA--------------------------A---G-SYSSYG-S-KSN-AA--PQG-S-GS-A----GN-GRYT-P------PAA----------------SF---- 461

WP_025694959_1 NSGLSAGA---A----D-------AASF------P-SAPYEA----------------------------DA-SS-------M--------TRGGAR--------------------------D---A-VYP--D-R-PAS-PS--QLR-D-GA-P----YG-GGYR-S----DSRTA----------------SR---- 477

WP_042207003_1 YSAPSAGA---A----A-------AASS------P-SAPHKA----------------------------DG-SS-------M--------TSRSGR--------------------------D---A-VYP--D-R-PAS-QS--QLR-D-GA-A----YA-GGYR-S----DSRTA----------------SR---- 477

WP_027084813_1 SRDSRVSE---Q----K-------PSYG------N-NSF-----------GGINYG--------SAG---SY-AK----------------PSSQAS------------------------------S-SYA--T-R-----PF-------------------------------------------------------- 436

WP_044876703_1 ----------------------------------A-NRPIGN----------------------------D--------------------SVREAS--------------------------A---Q-GYY----A-KGT-TN--GLSAP-SY-R----NS-QTVN--------------------------------- 431

WP_099476734_1 ----------------------------------------------------------------------------------R--------ASITGS--------------------------S---P-DYK--D-W-TAQ-QP---QM-R-ET-A----AA-AAYP-S----KPGYE----------------DRTV-- 440

WP_036637535_1 ----------------------------------------------------------------------------------L--------QPESGI--------------------------R---R-SYG--D-G-SGG-TPATAPM-R-ET-A----AA-SSYR-S----DYRYE----------------NK---- 444

WP_016362589_1 QAPWAPPA---R----A-------DGPA------A----------------------------------------------------SA--PRLREA--------------------------G---A-AYA--E---RPA-PA------R-GP-G-------DARE-T-------------------------AA---- 451

WP_068658264_1 -------------------------------------------------------------------------------------------PVRENR--------------------------E---Q-NYR----V-RDE-LG--YR--P-TD-S----KQ-TPFN--------------------------------- 409

WP_062408748_1 -APWAQPP---A----G-------DAPR------P----------------------------------------------------ER--PAFAAE--------------------------P---A-PRG--E---PPA-PV------R-AE-A-PR-ERLQPRE-A-------------------------AA---- 426

WP_042161919_1 PPDATERL---Y----K-------PAEP------Q-NSALRE--------RQAFW---------------EQ-AA----------------PTVQSV------------------------------PESNP--A-A-----DS--WME-E-AH---------------------------------------------- 502

WP_068609064_1 ----TPPA---R----A-------QAPA------M----------------------------------------------------SS--PG-RGA--------------------------A---A-PDG--G---AAR-EG------R-PA-Y-------GGRE-P-------------------------VP---- 432

WP_046232528_1 PKDAAERL---Y----K-------PAQA------G-NGLARE--------ASAAWGNFTSETVQPHGRAAAD-SS----------------PSQSQD------------------------------A-PLP--S-E-----PS--WIR-Q-DAAS-TA-SS-QTMA---NGSEPGNG--------LE------GT---G 481

WP_099520158_1 PKDAAERL---Y----K-------PAQT------G-NSLARE--------ASAAWGNFASEAVQSLGRAAAN-SS----------------PSQSED------------------------------A-PLP--A-E-----PA--WIR-Q-DA----A-SS-QTAA---NGSEPGNG--------LE------SA---G 478

WP_015844205_1 PSDATERL---Y----K-------PAEQ------R-ETSVRE--------AAAPW---------------EQ-AA----------------PALQAA------------------------------E------------------------------------------------------------------------ 458

WP_091189189_1 PKDATERL---Y----K-------PAEQ------R-EISVRE--------TAAPW---------------EQ-AA----------------PALQQT------------------------------D------------------------------------------------------------------------ 454

WP_006036759_1 PPDATERL---Y----A-------IPPA------A-DERVN-----------------------------AV-RE----------------PSMDDA------------------------------D-FYA--P-A-----PA-------------------------------------------------------- 460

WP_062492889_1 PRDATERL---Y--------------AP------A-AMPQRR-----------DD---------GGGA--VQ-DS----------------AAVREA------------------------------D-NAA--G-G-GLR-AE--TMA-A-GG-N-DD-GL-RTYA---PG-RTEPP--------AA------PS---W 457

WP_015254643_1 PRDAADRL---Y--R-P-------SARL------N-EAAIRE--------TAAGL---------AYG---AD-RS----------------TADTGG------------------------------F-SGA--A-G-----PA--AAP-A-AD---------------------------------------------- 425

WP_089523565_1 PPDAAQRL---YAPAAG-------AFGA------A-GLRERSAAPYAPAPAGSGA---------SAGG--FA-YP----------------PTTQQA------------------------------N-PPA--P-NDGDELSA--SLA-A-AG-S-PA-DL-QEPP---QGYRPAAP--------TE------LS---A 542

WP_045672515_1 ILSDDGAAIFSG----DSAAV--------P-SE---DSA-AVRSADSAAGT-----------Y----------AAET-GTATEG----A---------------------GDFPELSWVGQLHGTYLIAQNADGMYLIDQHAAHERINYEYYYRKFGTPLAASQQLIVPFTLEYTSSEAANLTQLLPLLTEAGVELEPFG 611

WP_016822099_1 ----------------TTK---FTDQK--T-HQ---ERL-------------------------KTEQL----AALS-GAAPEV--------------------------PTFPELNLIGQHHGTYLIAQNDQGLYLIDQHAAHERVNYEFYYEKFGNPESVSQELLLPITLEFTPSETEKLKTRLHWFEQAGVYLEHFG 618

WP_102163696_1 ----------------TTK---FTDQK--P-RQ---EHL-------------------------NTEQL----TALS-GAAPEV--------------------------PTFPELNLIGQHHGTYLIAQNDQGLYLIDQHAAHERVNYEFYYEKFGNPESVSQELLLPITLEFTPSETEKLKTRLHWFEQAGVYLEHFG 606

WP_013371549_1 ----------------TTK---FTDQK--P-RQ---EHL-------------------------NTEQL----TALS-GAAPEV--------------------------PTFPELNLIGQHHGTYLIAQNDQGLYLIDQHAAHERVNYEFYYEKFGNPESVSQELLLPITLEFTPSETEKLKTRLHWFEQAGVYLEHFG 594

WP_074095505_1 ----------------RST---YNPSSI-A-RG---ERTWK------------------TSGLPDATKL----AAAI-KSDASM--------------------------PEFPELSLIGQHHGTYLIAQNDQGLYLIDQHAAHERVNYEYYYEKFGNPAQASQELLLPITLEFTPSETEKLKTRLAWFEQAGVYLEHFG 640

WP_087435490_1 ----------PPP---AAGSM--------P------KKS-G-------------------DAW-KE--W-MD-SGDG-SAEAAL--------------------------PPFPALSLIGQLHGTYLIASNEEGLYLIDQHAAHERINYEYYYEQFGQPAQASQELLLPITLEFTPSEAARIRERMHLLEQIGMQMEPFG 622

WP_023989046_1 ----------------AMDSRHFADQK--P-RQ---QRL-------------------------DAEQF----AAVS-GEVPEL--------------------------PTFPELNLIGQHHGTYLIAQNDQGLYLIDQHAAHERVNYEFYYEKFGNPASVSQELLLPITLDFTPSETEKLKTRLHWFEQAGVYLEHFG 620

WP_053325620_1 ----------------ATDSRHFADQK--P-RQ---QRL-------------------------DAEQL----AAVS-GEVPEL--------------------------PTFPELNLIGQHHGTYLIAQNDQGLYLIDQHAAHERVNYEFYYEKFGNPASVSQELLLPITLDFTPSETEKLKTRLHWFEQAGVYLEHFG 620

WP_071641229_1 ----------------ATDSRHFADQK--P-RQ---QRL-------------------------DAEQL----AAVS-GEVPEL--------------------------PTFPELNLIGQHHGTYLIAQNDQGLYLIDQHAAHERVNYEFYYEKFGNPASVSQELLLPITLDFTPSETEKLKTRLHWFEQAGVYLEHFG 620

WP_005549848_1 --KSYQSYPNSKQ---QAGQS--------T------TKA-G------------------TEKW-KE--W-LT-EEGS-ADSAAL--------------------------PKFPALSYIGQLHGTYLIASNEEGLYLIDQHAAHERINYEYYFERFGKPVEASQELLLPITLEFTPADAERIRSRMHLLEQVGIVMEPFG 691

WP_068617384_1 RSSVGSRYDYERP---APPAA--------S-VSP--DKL-----------------------------Y----GVPG--SKPAM--------------------------PAFPKLSLIGQHHGTYLIAQNEEGLYLIDQHAAHERVNYEYYYEKFGNPADASQELLIPITLEFTSADSEKLKTRLQWFENAGVILEHFG 598

WP_094154817_1 ----------------AADSRYFADEK--P-RQ---PRL-------------------------NAEQL----AAMS-GEAPEL--------------------------PTFPELNLIGQHHGTYLIAQNDQGLYLIDQHAAHERVNYEFYYEKFGNPESVSQELLLPITLDFTPSETEKLKTRLHWFEQAGVYLEHFG 629

WP_042177336_1 ----------------ARGSL--------P-SA---EEL-----------------------------Y----APSG-SADPGL--------------------------PQFPELNYIGQHHGTYIIAQNDGGLYLIDQHAAHERINYEYYYEKFGRPEDASQELLLPITMEFTPSESRQLSERLHWFEKAGVYLEHFG 617

WP_037288812_1 ----------------NSAAL--------A-RA---AEL-----------------------------Y-SR------GEAPKR--------------------------PTFPEMTLIGQHHGTYLIAQNDQGLYLIDQHAAHERINYEYYYEKFGQPEEASQELLLPITLEFTSAESTKIRDRLGWFEQAGVYMEPFG 592

WP_014281438_1 ----------------AVDSRYFTDQK--P-RQ---SRL-------------------------NAEQL----AVVS-GEAPEL--------------------------PAFPELNLIGQHHGTYLIAQNDQGLYLIDQHAAHERVNYEFYYEKFGNPEAVSQELLLPITLEFTPSETEKLKTRLHWFEQAGVYLEHFG 600

WP_068700502_1 RSGGGAKPSASGSVN-RNPAV--------P-AAA--GRL-----------------------------Y----ESPA--EQAGL--------------------------PAFPELTYIGQHHGTYLIANNDTGLYLIDQHAAHERVNYEYFYEKFGAPADASQELLLPITIEFTPSESAKLKERLHWFEQVGVVLEHFG 619

WP_010273601_1 ----------------AELAA-GLQPQ--P-AT-------A------------------AEST---------------RGPGGL--------------------------PAFPALTPIGQLHGTYILAQNEQGLYLIDQHAAHERINYEYYYEKFGKPEEASQELLVPLTLEFTPAEAALLNERLDWFERVGVYMESFG 645

WP_076171200_1 -----------------------------S-------RL-----------------------------L----AGTE--APPEI--------------------------PPFPELTLIGQHHGTYLIAQNADGLFLIDQHAAHERINYEFYYEKFGRPADASQELLLPITLEFTPSESEQLTQKLHWFEQVGVYLEHFG 597

WP_062328181_1 ----------------RSS---YNPSAAGT-KG---ERSWK------------------APSLPDPARL----ASAI-KSDVSM--------------------------PAFPELSLIGQHHGTYLIAQNQDGLYLIDQHAAHERVNYEYYYEQFGNPAQASQELLLPITLEFTPSETEKLKARLAWFEQAGVYLEHFG 627

WP_042228138_1 ----------------AGGRT-GRAPA--P-DD-------R------------------PYSR---------------EAAGSLLAALP-----------TRSDKEALKRPDFPSLSPIGQLHGTYLLAQNEAGFYMIDQHAAHERINYEYYYTKFGEPQEASQELLVPIPLEFTSADFQALRDKLPLLEQVGVYLEAFG 652

WP_042137912_1 ----------------DRGQM--------P-TA---EEL-----------------------------YAPAVAPAG-SEDTGL--------------------------PQFPELNYIGQHHGTYIIAQNDSGLYLIDQHAAHERVNYEFYYEKFGKPEDASQELLLPITLEFTPSESRQLSERLHWFQQAGVFLEHFG 604

WP_042236923_1 ----------------DRGQM--------P-TA---EEL-----------------------------YAPAIAPAG-SEDTGL--------------------------PQFPELNYIGQHHGTYIIAQNDSGLYLIDQHAAHERVNYEFYYEKFGKPEDASQELLLPITLEFTPSESRQLSERLHWFQQAGVFLEHFG 599

WP_042214549_1 ----------------DRGQM--------P-TA---EEL-----------------------------YAPAIAPAG-SEDTGL--------------------------PQFPELNYIGQHHGTYIIAQNDSGLYLIDQHAAHERVNYEFYYEKFGKPEDASQELLLPITLEFTPSESKQLSERLHWFQQAGVFLEHFG 592

WP_038696521_1 ----------------NALPP--------Q-AG---REL-----------------------------Y----APAP-ESPSTL--------------------------PPFPELSYIGQHHGTYIIAQNDDGLYLIDQHAAHERVNYEYYYEKFGRPAEASQELLLPITLEFTPSESQKLSERLNWFQQAGVYLEHFG 608

WP_042128372_1 ----------------SQKQL--------P-SV---EKL-----------------------------W----APPQQQEEPGL--------------------------PAFPELNYIGQHHGTYIIAQNDGGLYLIDQHAAHERINYEFFYEKFGRPEDASQDLLLPITLEFTPSESRQLSERLHWFQQAGVYLEHFG 560

WP_038571591_1 ----------------SQKQL--------L-SV---EKL-----------------------------W----APPQ-QEEPGL--------------------------PAFPELNYIGQHHGTYIIAQNDGGLYLIDQHAAHERINYEYFYEKFGRPEDASQELLLPITLEFTPSESRQLSERLHWFQQAGVYLEHFG 557

WP_039873825_1 ----------------ARGSL--------P-SA---EEL-----------------------------Y----APGQ-GADPGL--------------------------PQFPELNYIGQHHGTYIIAQNDGGLYLIDQHAAHERINYEYYYEKFGRPEDASQELLLPITMEFTPSESRQLSERLHWFEKAGVYLEHFG 605

WP_079940587_1 ----------------QGGGV-A-----------------------------------------------------------------------------------------FPELTPVGQVHGTYLVAQNEEGLFLIDQHAAHERINYEYYYHKFGQPEEASQELLMPISLEFTPSEAAALKDRLHLLEQVGVHIEPFG 577

WP_046504127_1 ----------------AKGQM--------P-SA---EEL-----------------------------Y----APPG-TEDAGL--------------------------PQFPELNYIGQHHGTYIIAQNDSGLYLIDQHAAHERVNYEYYYEKFGRPEDASQELLLPITLEFTPSESKQLSERLHWFQQAGVYLEHFG 596

WP_042266908_1 ----------------ARGQL--------P-SA---EEL-----------------------------Y----APPG-NEEAGL--------------------------PQFPELNYIGQHHGTYIIAQNDSGLYLIDQHAAHERVNYEYYYEKFGRPEDASQELLLPITLEFTPSESKQLSERLHWFQQAGVYLEHFG 592

WP_025335246_1 ----------------KEQPV--------P-GT---EEL-----------------------------W----APAG-SETSGL--------------------------PPFPELNYIGQHHGTYIIAQNDGGLYLIDQHAAHERINYEYYFEKFGRPAEASQELLLPITLEFTPSESKLLGERLQWFEQAGVYLEHFG 593

WP_108465825_1 ----------------RTSAL--------P-KPEHIERL-----------------------------Y----ESPA--EAPQV--------------------------PQFPELTLIGQHHGTYLIANNDTGLYLIDQHAAHERVNYEYYYEKFGSPADASQELLLPITLEFTPSDSEKLKDRLHWFESAGVILEHFG 556

WP_038593031_1 ----------------PRGGM--------P-SA---EEL-----------------------------Y----APTG-SEDPAL--------------------------PQFPELNYIGQHHGTYLIAQNDSGLYLIDQHAAHERINYEYYYEKFGRPEDASQELLLPITLEFTPSESRQLSERLHWFEQAGVFLEHFG 598

WP_081951145_1 ----------------LQKQI--------P-SA---QAL-----------------------------W----APPV-DEEPGL--------------------------PAFPELNYIGQHHGTYIIAQNDGGLYLIDQHAAHERINYEFFYEKFGRPEDASQDLLLPITLEFTPSESRQLSERLHWFQQAGVYLEHFG 557

WP_068648544_1 -----------------------------P-------EV-----------------------------Y----AIPK-NGSPEI--------------------------PPFPELTLIGQHHGTYLIAQNEEGLYLIDQHAAHERINYEFYYEQFGRPSDASQELLLPITLEFTPSETEQLKQKINWFEQVGVYLEHFG 543

WP_036656101_1 ----------------AREVA--------A-GI---EEL-----------------------------L----APPA-GGGTGL--------------------------PVFPELNYIGQHHGTYIIAQNEEGLYLIDQHAAHERINYEFYYEQFGRPADASQELLLPITLEFTPSESRQLGERLHWFEQAGVYLEHFG 547

WP_015734656_1 ----------------GGAGI--------P-AV---TKE-----------------------------W--L-QAVS-GPAPEI--------------------------PPFPELTLIGQHHGTYLIAQNDTGLYLIDQHAAHERINYEYYYEQFGNPADASQELLLPITLEFTPSESEKVKDRLHWFEKVGVYMEFFG 556

WP_016312025_1 ----------------RLQEM--------P------EAL-----------------------------Y----GSPA--AAPEL--------------------------PAFPELSLIGQHHGTYLIAQNDQGLYIIDQHAAHERVNYEYYYEKFGRPADASQELLLPITLEFTPSDSAKLQERLHWFEQVGVVLEHFG 590

WP_087920285_1 ----------------PRAQL--------P-SA---EAV-----------------------------W----APAA-DGDTGL--------------------------PPFPELNYIGQHHGTYIVAQNDGALYLIDQHAAHERVNYEFYYEKFGRPEEASQELLLPITLEFTPSESRQLSERLHWFQQAGVYLEHFG 573

WP_025694959_1 ----------------PAQAL--------P-GT---EEL-----------------------------W----APAG-SGNTGL--------------------------PPFPELTYIGQHHGTYIIAQNDEGLYLIDQHAAHERINYEFYYEKFGRPAEASQELLLPITLEFTPAESKLLAERLQWFEQAGVYLEPFG 589

WP_042207003_1 ----------------PAQAL--------P-GT---EEL-----------------------------W----APAG-SENVGL--------------------------PPFPELTYIGQHHGTYIIAQNDEGLYLIDQHAAHERINYEYYYEKFGRPAEASQELLLPITLEFTPAESKLLAERLQWFEQAGVYLEPFG 589

WP_027084813_1 ----------------PTSQQ--------P-PV---QIP-D------------KVW-----------------EAAF-AAPPVK----P----------------ET---PAFPELSWIGQLHGTYIVAQNENGLYLIDQHAAHERIHYEWYYDKFGRPEEASQELLLPVTLEFAPDECLVLRGRLAAFESAGVYLEDFG 554

WP_044876703_1 -----------------------------PVMT---QEL-----------------------------F----AQAS-SGISEI--------------------------PPFPELTLIGQHHGTYLIAQNEKGLYLIDQHAAHERINYEFYYEQFGQPADASQELLIPITLEFTPSESEQLKQKLNWFEQVGVFLEHFG 539

WP_099476734_1 --------------ERRSANV--------P-AV---TKE-----------------------------W--M-TAAS-GPAPDI--------------------------PPFPELTYIGQHHGTYIIAQNETGLYLIDQHAAHERINYEYYYEQFGKPADASQELLLPITLEFTPSETEKVKERLHWFEKAGVYMEFFG 555

WP_036637535_1 ----------------GNAHI--------P-SV---TKE-----------------------------W--L-QAAS-GPAPEI--------------------------PPFPELTLIGQHHGTYLIAQNDSGLYLIDQHAAHERINYEYYYEQFGKPAAASQELLLPITLEFTPSESEKVKQRLHWFEKVGVYMEFFG 557

WP_016362589_1 ----------------ASGAL-LRALAQ-T-EA---------------------------------------------SAPPAL--------------------------PAFPKLTPIGQLHGTYIVAQNEEGLFLVDQHAAHERINYEHYFDLFGRPAEASQELLVPMTLEYTSVEALRLTEKLPQLEQAGVYLEPFG 561

WP_068658264_1 -----------------------------P-------EI-----------------------------Y----ARPE-GGSSEI--------------------------PPFPELTLIGQHHGTYLIAQNEDGLYLIDQHAAHERINYEFYYEQFGRPDDASQELLLPITIEFTPSESEKLKQKLNWFEQVGVYIEHFG 513

WP_062408748_1 ----------------ASGAL-LRTLA--P-EP---------------------------------------------ETPPAL--------------------------PGFPKLQPIGQLHGTYIVAQNEEGLFLIDQHAAHERINYERFFDMFGRPEEASQELLVPITLEFTAVEAERLADKLPQLEQAGVYLEPFG 535

WP_042161919_1 --------------I-ESPAE--------A-DP---ASN-T--------------------------------ATIA-GIASRE----A-------------TGAAS---PAFPELYWIGQLHGTYIVAQAEDGLYLIDQHAAHERINYEYYYMKFGNPAAASQQLLVPLTLEFTPNDAQQLRTMTDLFAEAGVEIEPFG 621

WP_068609064_1 ----------------AAG-L-LPALA--P-AA---------------------------------------------PAAAPL--------------------------PAFPELHPIGQMHGTYIVAQNPKGLYLIDQHAAHERIHYEYYYEQFGKPQPASQELLIPITLEFTPSEADTLQQRLPLFEQAGVYMEPFG 540

WP_046232528_1 AAIADLSSEVAAS-V-NTAAL--------P-DI---NLV-Q--------------------------------GGQA-PAAPEQ----P-------------SAANE---AGFPELHWIGQLHGTYIVAQSEEGLYLIDQHAAHERINYEYYYAKFGMPEVASQQLLVPLTLEFTPGEAEQLRGLLPLFNEAGVELESFG 613

WP_099520158_1 AATADLSSEVAAT-V-HTAAL--------P-DV---NFG-Q--------------------------------GGQV-PVAPEQ----P-------------SAANE---AGFPELHWIGQLHGTYIVAQSEEGLYLIDQHAAHERINYEYYYAKFGMPEAASQQLLVPLTLEFTPGEAEQLRGLLPLFNEAGVELEPFG 610

WP_015844205_1 ----------------ETSAP--------P-P----APD-S--------------------------------ERPV-GEASER----P-------------DG--T---PAFPDLYWIGQLHGTYIVAQAEEGLYLIDQHAAHERINYEYYFRKFGNPQAASQQLLVPLTLEFTSGEAEQLRGMIGLFQEAGVELEPFG 573

WP_091189189_1 ----------------DMPQQ--------P-QF---TEQ-P--------------------------------ASQP-GQPERT----Q-------------EG--T---PAFPELYWIGQLHGTYIVAQAEEGLYLIDQHAAHERINYEYYYQKFGNPQAASQQLLVPLTLEFTSGESEQLRGMLELFREAGVELESFG 570

WP_006036759_1 ----------------EQQAQ--------P-AS---EQR-A--------------------------------STGL-PPRIAV----E-------------RDPET---PPFPELSWIGQLHGTYLIAQSEDGLYMIDQHAAHERIHYEYYVEKFGRPQQASQQLLVPITLELTAGEAAQLHDKLELLAEVGLELESFG 578

WP_062492889_1 REAAPAAEAYAA----EASSG--------T-DA---AGA-S--GGAAESGL--------DAAE----------TAGA-GLPDGG----A-----------EPGGPRA---DAFPELSWIGQLHGTYIVAQNEEGLYLIDQHAAHERIHYEFYLGKFANPQPASQPLLVPMTLEYTPAEAALLRERLDTLAEAGIGLEPFG 601

WP_015254643_1 ----------AA----NPAAK--------P-GP---ADA-V------------QPHPA---------------AGQD-AVRPDG----E---------R---EDSER---RAFPEMSWIGQLHGTYILAQSEDGLYLIDQHAAHERINYEYYLRKFSEPPKLSQTLLVPMTLEFTSGEAEAIRSRLDAFAAAGVELEPFG 551

WP_089523565_1 AVDSDADPAFGA----DPSSA--------V-EQ---VSG-AYRGSGTDSASDADPAHATDPAF----------AADL-AHATDS----APYADPASASNPSSAGATD---SGFPELSWVGQLHGTYLIGQNEEGMYLIDQHAAHERINYEYYYRKFGEISRESQPLLVPFTLEYTSVEAMQLADRLPLLAEAGVELEPFG 707

WP_045672515_1 TNAFLVRSHPEWLPKGDEQSVIAEIVEWVLSEKKAVDIAKLREKSAILCSCKASIKANDRMNREEGEFLLARLAACSQPYTCPHGRPILVHISTYQLEKMFKRVM- 716

WP_016822099_1 GQTFRVSSYPYWLPKGEEADVIEEMAGWVLEEK-AIDLAKLREAASIMCSCRASIKANQKLTDRQAVVLLERLAACKQPYTCPHGRPIVVSFSTYDLEKLFKRVM- 722

WP_102163696_1 GQTFRVSSYPYWLPKGEEADVIEEMAGWVLEEK-AIDLAKLREAASIMCSCRASIKANQKLTDRQASVLLERLAACKQPYTCPHGRPIVVSFSTYDLEKLFKRVM- 710

WP_013371549_1 GQTFRVSSYPYWLPKGEEADVIEEMAGWVLEEK-AIDLAKLREAASIMCSCRASIKANQKLTDRQASVLLERLAACKQPYTCPHGRPIVVSFSTYDLEKLFKRVM- 698

WP_074095505_1 GQTFRVRSHPFWFPKGDEKDIIEEMSEWVLSER-SIDVAKMREAASIMCSCKASIKANQKLTDQEAEVLIQRLGSCRQPYTCPHGRPIVISFSIYDLEKLFKRVM- 744

WP_087435490_1 GQTFLVRSHPYWFPKGQETAIIQEMTDWVLGEK-APDIAKLREASAIMCSCKASIKANQRLSDAEAETLFARLATCRQPYTCPHGRPIVVRFTTYDLEKMFKRVM- 726

WP_023989046_1 GQTFRVSSYPYWLPQGEEADVIEEMAGWVLEER-AIDLAKLREAASIMCSCRASIKANQKLTDRQAIVLLERLAACKQPYTCPHGRPIVVSFSTYDLEKLFKRVM- 724

WP_053325620_1 GQTFRVSSYPYWLPQGEEADVIEEMAGWVLEER-AIDLAKLREAASIMCSCRASIKANQKLTDRQAIVLLERLAACKQPYTCPHGRPIVVSFSTYDLEKLFKRVM- 724

WP_071641229_1 GQTFRVSSYPYWLPQGEEADVIEEMAGWVLEER-AIDLAKLREAASIMCSCRASIKANQKLTDQQAIVLLERLAACKQPYTCPHGRPIVVSFSTYDLEKLFKRVM- 724

WP_005549848_1 GQTFLIRSHPYWFPKGEEANIIQEMTDWVLNEK-APDIAKLREASATMCSCKASIKANQRLTAAEAETLFVRLGTCRQPFTCPHGRPIVIRFTTYDLEKMFKRVM- 795

WP_068617384_1 GGTFRVISHPYWFPQGEEASIIEEMAEWVLSER-AIDLAKLRERSSILCSCKASIKANQRLTDQEATTLLDRLAACKQPYTCPHGRPIVVSFSTYDLEKMFKRVM- 702

WP_094154817_1 GQTFRVSSYPYWLPKGEEADVIEEMAGWVLEEK-AIDLAKLREAASIMCSCRASIKANQKLTDRQAVVLLERLAACKQPYTCPHGRPIVVSFSTYDLEKLFKRVM- 733

WP_042177336_1 GQTFLVRSLPYWFPEGEEKALVEEMAEWVLSER-SIDLAKLREKSSILCSCKASIKANQKLTEPEVDALLSRLAACKQPYTCPHGRPIVVSFSAYDLEKLFKRVM- 721

WP_037288812_1 GQTFRVTAYPHWFPAGDESSIIEEMAEWVLSER-AIDLAKLREKASTLCSCKASIKANQRLSDIQAQTLIERLGRCKQPYTCPHGRPIVVSFSSYDLEKLFKRVM- 696

WP_014281438_1 GQTFRVSSYPYWLPQGEEADVIEEMAGWVLDEK-AIDLAKLREAASIMCSCRASIKANQKLTDRQAVVLLERLAACRQPYTCPHGRPIVVSFSTYDLEKLFKRVM- 704

WP_068700502_1 GQTFRVVSLPYWFPQGEEAALVEEMAEWVLNER-TIDLAKLREKSSILVSCKASIKANQKLTEDEANTLLKRLAACRQPYTCPHGRPIVVSFTSYDLEKLFKRVM- 723

WP_010273601_1 GASFIVRAYPHWFPAGEEKELIEEMAEWMLTEKKQLDLAKLREKSSTLCSCKASIKANQALSIAEMETLIRRLAGCRIPYTCPHGRPIVVSFSTYELEKMFKRVM- 750

WP_076171200_1 GQTFRVVSHPYWFPKGEEKALIEEMAEWVLSER-SIDLAKLRETSSILCSCKASIKANQKLTEQEATTLLDRLAACRQPYTCPHGRPIVVSFSTYDLEKLFKRVM- 701

WP_062328181_1 GQTFRVRSHPFWFPKGDEKDIIEEMAEWVLSER-SIDVAKMREAASIMCSCKASIKANQKLTDQEAEVLIQRLGSCRQPYTCPHGRPIVVSFSTYDLEKLFKRVM- 731

WP_042228138_1 GNTYLVRAYPHWFPAGDEQAIVEEMCEWILTERKGVDLSKLREKSSTLCSCKASIKANQSLSTAEMEALLDRLGGCGNPYTCPHGRPIVISFSTYELEKMFKRVM- 757

WP_042137912_1 GQTFLVRSLPYWFPEGDEKAVIEEMAEWVLSER-SIDLAKLREKSSILCSCKASIKANQKLTELEVESLLSRLAACRQPYTCPHGRPIVVSFSSYDLEKLFKRVM- 708

WP_042236923_1 GQTFLVRSLPYWFPEGDEKAVIEEMAEWVLSER-SIDLAKLREKSSILCSCKASIKANQKLTELEVESLLSRLAACRQPYTCPHGRPIVVSFSSYDLEKLFKRVM- 703

WP_042214549_1 GQTFLVRSLPYWFPEGDEKAVIEEMAEWVLSER-SIDLAKLREKSSILCSCKASIKANQKLTELEVESLLSRLAACRQPYTCPHGRPIVVSFSSYDLEKLFKRVM- 696

WP_038696521_1 GQTFLVRSLPYWFPRGEEKEIVEDMAEWVLSEK-NIDLAKLREKSSILCSCKASIKANQKQTEQEAMSLLDRLAACRQPYTCPHGRPILISFSPYDLEKLFKRVM- 712

WP_042128372_1 GQTFLIRSLPYWFPEGEEKAIVEEMAEWVLSER-LIDLAKLREKSSILCSCKASIKANQKLTEQEVEALLSRLAACKQPYTCPHGRPIVISFSSYDLEKLFKRVM- 664

WP_038571591_1 GQTFLVRSLPYWFPEGDEKAVVEEMAEWVLSER-MIDLAKLREKSSILCSCKASIKANHKLTEQEVEALLSRLAECKQPYTCPHGRPIVISFSSYDLEKLFKRVM- 661

WP_039873825_1 GQTFLVRSLPYWFPEGEEKALVEEMAEWVLSER-SIDLAKLREKSSILCSCKASIKANQKLTEPEVDALLSRLAACKQPYTCPHGRPIVVSFSAYDLEKLFKRVM- 709

WP_079940587_1 GNTFLVRAYPHWFPSGDEKEIIDEMCEWILSEKQGVDLAKLREKSSTLCSCKASIKANQNLSRLEMEVLLDRLSGCKNPYTCPHGRPIVVSFSTYELEKMFKRVM- 682

WP_046504127_1 GQTFLVRSLPYWFPEGDEKAVIEEMAEWVLSER-SIDLAKLREKSSILCSCKASIKANQKLTEHEVESLLSRLAACRQPYTCPHGRPIVVSFSSYDLEKLFKRVM- 700

WP_042266908_1 GQTFLVRSLPYWFPEGDEKAVIEEMAEWVLSER-SIDLAKLREKSSILCSCKASIKANQKLTEHEVESLLSRLAACRQPYTCPHGRPIVVSFSAYDLEKLFKRVM- 696

WP_025335246_1 GQTFLVRSLPYWFPKGEEKEIIEEMAEWVLGER-NIDLAKLREKSSILCSCRASIKANQKLTEQEVESLLSRLSACRQPYTCPHGRPIVISFSPYDLEKLFKRVM- 697

WP_108465825_1 GQTFRVVSHPYWFPKGEEAAIIEEMADWVLNER-SIDLAKIREKSSILCSCKASIKANQKLTQEEAVTLLRRLAACRQPYTCPHGRPIVVSFSTYDLEKLFKRVM- 660

WP_038593031_1 GQTFLVRSLPYWFPEGDEKAVIEEMAEWVLSER-YIDLAKLREKSSILCSCKASIKANQKLTEQEVEALLSRLAACRQPYTCPHGRPIVVSFSSYDLEKLFKRVM- 702

WP_081951145_1 GQTFLIRSLPYWFPEGEEKAIVEEMAEWVLSER-LIDLAKLREKSSILCSCKASIKANQKLTEQEVEALLSRLAACRQPYTCPHGRPIVISFSSYDLEKLFKRVM- 661

WP_068648544_1 GQTFRVRAHPYWFPKGDEQSLIEEMAEWVLSER-MIDLAKLREKSSILCSCKASIKANQKLTNLESDTLLERLAACKQPYTCPHGRPIVISFSSYDLEKLFKRVM- 647

WP_036656101_1 GQTFLVRSLPYWFPQGEEKAIIEEMAEWVLSEK-TIDLAKLREKSSILCSCKASIKANHKLTEQEVDSLLTRLSACRQPYTCPHGRPILISFSAYDLEKLFKRVM- 651

WP_015734656_1 GHTFLVRSHPFWFPKGEEKAIIEEMAEWVLNER-NIDIAKLRETSSIMCSCKASIKANQKLTEQEAMTLIQRLGACRQPYTCPHGRPIVVSFSAYDLEKMFKRVM- 660

WP_016312025_1 GGTFRVVAHPYWFPQGEEASIIEEMAEWVLNER-AIDLAKLREASSTMVSCKASIKANQKLTPEEANTLLRRLAACKQPYTCPHGRPIVVSFSTYDLEKLFKRVM- 694

WP_087920285_1 GQTFLVRSLPYWFPDGDEKAVIEEMAEWVLSER-HIDLAKLREKSSILCSCKASIKANQKLTEHEVESLLVRLAACRQPYTCPHGRPIVISFSAYDLEKLFKRVM- 677

WP_025694959_1 GQTFLVRSHPYWFPKGDEKEIIEEMAEWVLGEK-SIDLAKLREASSILCSCKASIKANQKLTEQEVESLLSRLSACRQPYTCPHGRPIVISFSAYDLEKLFKRVM- 693

WP_042207003_1 GQTFLVRSHPYWFPKGDEKEIIEEMAEWVLGEK-SIDLAKLREASSILCSCKASIKANQKLTEQEVESLLSRLSACRQPYTCPHGRPIVISFSAYDLEKLFKRVM- 693

WP_027084813_1 GNTFMIRAVPHWFPDGDEAAVVREMAEWVLKE-KTIDLKALREKAAILCSCKASIKANQAQTREAGEKLLERLAACRQPYTCPHGRPIVVSFSAYELEKMFKRVT- 658

WP_044876703_1 GQTFLVRSHPYWFPKGDEQGIIEEMVEWVLGER-TIDLAKLREKSSVMCSCKASIKANQKLTNLESDALLERLAACKQPYTCPHGRPIVVSFTTYDLEKLFKRVM- 643

WP_099476734_1 GNTFLVRSHPFWFPKGDEKALIEEMAEWVLSER-HIDIAKLREASSIMCSCKASIKANQKLTEQEAMMLIRRLAECRQPYTCPHGRPIVVSFSPYDLEKMFKRVM- 659

WP_036637535_1 GQTFLVRSHPFWFPKGDEKSIIEEMAEWVLSER-NIDVAKLRETSSIMCSCKASIKANQKLTEQEAMTLIQRLGACRQPYTCPHGRPIVVSFSAYDLEKMFKRVM- 661

WP_016362589_1 GTSFLVRAYPHWLPKGEEQAIVEEMIDLLLQDKKGIDIAKFREKAAIMCSCKASIKANQSMGTLEIEVLLDRLAACRNPYTCPHGRPIVVSFSTYELEKMFKRVM- 666

WP_068658264_1 GQTFRVRSLPYWFPKGEEQEIIEEMAEWVLSER-MIDLAKLREKSSVLCSCKASIKANQRLTELESDTLIERLAACKQPYTCPHGRPIVISFSTYDLEKLFKRVM- 617

WP_062408748_1 GTSFLVRAYPHWLPKGDEQAIVEEMIEWLLSEKKQVDIGKLREKAAIMCSCKASIKANQSIGTLEIETLLDRLAACRNPYTCPHGRPIVVSFSTYELEKMFKRVQ- 640

WP_042161919_1 QQTFLVRSYPEWLPDGEEQSIIEEMADLLLSERKSIDVAKLREKAAIMCSCKASIKANDRKTREEGEALLARLGACNQPYTCPHGRPIIVHMSTYQLEKMFKRVMS 727

WP_068609064_1 GNAFLVRSYPYWFPEGEERSIIQEMADWILSEKKAVDIGKIREKSAILCSCKASIKANQSLTHVEIEKLLERLSVCSNPYTCPHGRPIVVSFSTYELEKMFKRVM- 645

WP_046232528_1 AQTFLVRAYPQWLPDGEEQDIIEEMADLLLTERKSINIAKLREKAAIMCSCKASIKANDRLTREEGEALLSRLGACHQPYTCPHGRPIVVHMSTYQLEKMFKRVMS 719

WP_099520158_1 SQTFLVRAYPQWLPDGEEKDIIEEMADLLLAERKSINIAKLREKAAIMCSCKASIKANDRLTREEGAALLSRLGACHQPYTCPHGRPIVVHMSTYQLEKMFKRVMS 716

WP_015844205_1 AQTFLVRAYPEWFPQGEEQAIIEEMAELLLSERKSVNIAKLREKAAIMCSCKASIKANDRKTREEGEALLARLAACNQPYTCPHGRPIVVHLSTYQLEKMFKRVMS 679

WP_091189189_1 PQTFLVRAYPEWFPQGEEQSIIEEMAELLLSERKSINIAKLREKAAIMCSCKASIKANDRKTREEGEVLLARLAACNQPYTCPHGRPIVVHISTYQLEKMFKRVMS 676

WP_006036759_1 ANTFLVRAHPEWLPAGDEQSIIEEMIEWVLQEKRNVDIQKLREKTAIMCSCKASIKANQRLTREEGEALLDRLGACGQPYTCPHGRPIVVHISSYQLEKMFKRVMS 684

WP_062492889_1 PHGFLVRYHPEWLPKGEEQALVEEMIEWVLSEKRHIDIGKLREKAAIMCSCKASIKANQRLTREEGETLIRRLAACRQPYTCPHGRPIVVHMSTYQLEKMFKRVMS 707

WP_015254643_1 GSAFLVRAYPEWLPQGSEREIIEEMAEWILAEKKSIDIGKLREKAAIMTACKASIKANDRMTREEGEALIARLSACSQPYTCPHGRPIVVHISTYQLEKMFKRVI- 656

WP_089523565_1 SQSFLVRSHPHWFPKGEEQQLVEEMVEWVLREKRAIDIAKLREEAAIMCSCKASIKANDRLSREEGEALLSRLAACQQPYTCPHGRPILVHISVYQLEKMFKRVMS 813

**paen9.01699, Regulator of replication initiation timing YabA**

WP_055105375_1 ----------MENK-DIFTHMQALDAQMGTVHGELSKLMLEVKKLVEENQQLRLENEQLRKILKRE--S---AEG-RLPS-SSS-EQIE-SALPE-GKEAQEAI---D-I-----------------------------VGEGYDNLARLYHEGFHICNVYYGHLRTEGDCLFCLSFLNK-- 126

WP_006038200_1 ----------MDNKNDLFVEVDELEGRIGGIHAELGLLKRHIKSLLEENKRLSIENNQLHKLLRQG------TSF-------LE-PQEE-PAAAD----ESTIQ-----DH--LHESGLETVKMADTVKEPMPT-----VSGGHDNLARLYHEGFHICNVYYGHLRTEGDCLFCLSFLNK-- 139

WP_054819630_1 ----------MNKR-DIFVQIEQLEEQTGVVHSELGSLKRQIIELLEENKRLSIENQQLRKLLKKE------TEP-ITPA-LLA-VGKV-ADASTDPAAASVLV---K-EP----------------------------TGEGYDNLARIYHEGFHICNVNYGHLRTEGDCLFCMSFLNK-- 127

WP_037286062_1 ----------MEKK-NVFAQIHEMETQVESFKDELGTMKQAVKELMEDNHRLITENERLREILKRE--V---Q---VESV-SGA-RSEP-FNVLSEPKRKDEES---S-DT----------------------------VGEGYDNLARLYHEGFHICNVYYGHLRTEGDCLFCLSFLNK-- 126

WP_013921259_1 MLLRLEVMESVDKR-DIFVQIEQMEEQMGTLYVELGQIKQQIIELIEENKRLSIENGQLRKLLKKD--A---E------------PLHP-VLQPV----GTTITLPGK-EP----------------------------TGEGYDNLARIYHEGFHICNVYYGHLRTEGDCLFCMSFLNK-- 128

WP_036584307_1 ----------MENK-DLFTQMGKLETGMSALLGEIGDMKQQIKSLLEENKRLSIENQQLRKLFKRE--A---A-G-HPPE-VKQ-NAEG-SAIID-PESYPYPL---Q-PP----------------------------VGEGYDNLARLYHEGFHICNVYYGHLRTEGDCLFCLSFLNK-- 126

WP_007428002_1 ----------MDKL-NVFARIHEMETQMGQLYSDLGELKLAVKELLEENQRLTIENEQVRKMLKRE-----TSGE-EKPA-SKP-KLSP-PIVIK----DEEGT---G-EV----------------------------VGEGYDNLARLYHEGFHICNVYYGHLRTEGDCLFCLSFLNK-- 124

WP_013368663_1 ----------MDKL-NVFARIHEMETQMGQLHSDLGELKLAVKELLEENQRLTIENEQVRKMLKRE-----TSGE-EKTA-SKP-KLPP-PIVVK----DEEET---G-EV----------------------------VGEGYDNLARLYHEGFHICNVYYGHLRTEGDCLFCLSFLNK-- 124

WP_014279024_1 ----------MDKL-NVFARIHEMETQMGQLHSDLGELKLAVKELLEENQRLTIENEQVRKMLKRE-----TSRE-EKTA-KRP-KLSP-PIVIK----DEEET---G-EV----------------------------VGEGHDNLARLYHEGFHICNVYYGHLRTEGDCLFCLSFLNK-- 124

WP_016818929_1 ----------MDKL-NVFARIHEMETQMGQLHSDLGELKLAVKELLEENQRLTIENEQVRKMLKRE-----TSGE-EKTA-SKP-KLPP-PIVVK----NEEET---G-EV----------------------------VGEGYDNLARLYHEGFHICNVYYGHLRTEGDCLFCLSFLNK-- 124

WP_023986425_1 ----------MDKL-NVFARIHEMETQMGQLHSDLGELKLAVKELLEENQRLTTENEQVRKMLKRE-----TSGE-EKTA-NKP-KLPP-PIVIK----DEGQT---G-EV----------------------------VGEGYDNLARLYHEGFHICNVYYGHLRTEGDCLFCLSFLNK-- 124

WP_025722558_1 ----------MDKL-NVFARIHEMETQMGQLHSDLGELKLAVKELLEENQRLTIENEQVRKMLKRE-----TSGE-EKTA-NKP-KLPP-PIVIK----DEGQT---G-EV----------------------------VGEGYDNLARLYHEGFHICNVYYGHLRTEGDCLFCLSFLNK-- 124

WP_027086734_1 ----------M--K-DIFARVNELESHLGAVHADFGSLKQKIKELLEENQRLRIENGQLRKVLKRE------NDP-TAPP-QTA-EQPA-AVKGD----KPAPV---A-LG----------------------------VGEGYDNLARLYHEGFHICNVYYGHLRMEGDCLFCLSFLSK-- 121

WP_010279439_1 ----------MDKK-ELFMKMEQLENHMQSSLLDLESLKKQLIQLTEENLRLMNENQQLRQMLKLE---------------NGQ-ENRE----------AMEGN-A-T-PV----------------------------IGEGYDNLARLYYEGFHICNVYYGHLRNEGDCLFCLSFLNK-- 112

WP_017815548_1 ----------MEKK-NLFEHIHELETQMNHMHGDMGRLKLAVKELLEENQRLTIENEQLRRVLKKE-----VPLE-VKVA--EP-QIAE-IFKDT----HSHPY---P-EV----------------------------VGEGYDNLARLYHEGFHICNVYYGHLRTEGDCLFCLSFLNKSE 125

WP_038692825_1 ----------MEKK-NIFAHIQEIEAQMETMRLNLEDWKQAVKELMEANQRLTLENEQLRRILKRE--A-----PRQNAA-PQP-VKAG-KRQTA----PETPA---E-EV----------------------------VGEGYDNLARLYHEGFHICNVNFGHLRTEGDCLFCLSFLNK-- 123

WP_005545507_1 ----------MEKK-DIFAQIHELETHMGDLHQELGALKLVVKELLEENQRLWMENQQLRKVLHHE--H---HEH-LEVE-PEV-KGDS-TPAVH-PDPNGDSK---E-NV----------------------------VGEGYDNLARLYHEGFHICNVYYGHLRTEGDCLFCLSFLNK-- 127

WP_089525350_1 ----------MDNK-DLFLHLDELETKLGGFHKELRELKLSVKQLLEANKQLSIENRQLRKVLKRE------TNP------HLD-PARQ-PAKAG----SVASA-----SN--SLPEDPSREADVSA------------VGEGHDNLARLYYEGFHICNVNYGHLRTEGDCLFCLSFLNK-- 132

WP_091190494_1 ----------MENK-EIFNQMEQLESQMGNIHAHIGVLKQRVKELLEENQQLSMENEQLRKILKRE------TSP------------KP-KSAAN----RNEGQ-----AAQQPVRREIAAHAEEDTVNDTSQQAGL--VGEAHDNLARLYHEGFHICNVYYGHLRTEGDCLFCLSFLNK-- 139

WP_012772020_1 ----------MENK-EIFNQMDELESRMGDFHVHLGVLKQRVKELLEENQQLSMENEQLRKILKRE------TSP------------KP-KTAAN----RQGGQ-----AV-QPARREVAVHAEEDTVNDTSQQAGL--VGEAHDNLARLYHEGFHICNVYYGHLRTEGDCLFCLSFLNK-- 138

WP_046234513_1 ----------MEKK-EIFVQMDQLDSQMGEFHRQVGDLKLRVKELLEENKRLSMENHQLRKILKRE------TVP-PQVE-EPL-PVKT-AGAAK----SNAGK-----TM--GTSAKVEQQTETADLFTSAEDAAAYSVGEGHDNLARLYHEGFHICNVYYGHLRTEGDCLFCLSFLNK-- 148

WP_099521531_1 ----------MEKK-EIFVQMDQLDTQMGEFHRQVGDLKLRVKELLEENKRLSMENHQLRKILKR--------IP-QQAE-EPL-PVKT-AGASK----TTAGK-----TM--GTSTKVEQQTETADLFTSAEDAAAYSVGEGHDNLARLYHEGFHICNVYYGHLRTEGDCLFCLSFLNK-- 146

WP_042171183_1 ----------MEKK-EIFEQMDELDTRMGQFHTELGALKQRVKQLLEENKRLSLENSQLRKILKRE------TVP-VQAA-SAA-RTET-AEAKP----KRQTE-----AA--SHEVSTADHSDGLDPFESSQQA----VGEGHDNLARLYHEGFHICNVYYGHLRTEGDCLFCLSFLNK-- 144

WP_045670340_1 ----------MEKK-DLFLQMNELENRLGGIHGELGLMKQLVKQLLEENKKLSIENQQLRDVLKRE------TSP------KPE-PVKS-ASAAR----RSSSP-----AG--AASVTESAAADSGA------------VGEGYDNLARLYHEGFHICNVYYGHLRTEGDCLFCLSFLNK-- 132

WP_087434314_1 ----------MEKK-DIFAQIHELETHMGDLHQELGALKLVVKELLEENQRLWMENQQLRKVLHHE--H---HEH-LEAL-PET-EGES-APAADDPELFNEAK---E-NV----------------------------VGEGYDNLARLYHEGFHICNVYYGHLRTEGDCLFCLSFLNK-- 128

WP_062489687_1 ----------MENK-DIFLEMDELESKLGTFHRELGELKLRIKWLLEENKRLSMENNQLRKILKRE------TIA-AAVG-DH----RQ-KEANG----ETAGR-----PE--EAKDQAASYAAA--------------VGEGYDNLARLYHEGFHICNVYYGHLRTEGDCLFCLSFLNK-S 132

WP_041065520_1 ----------M--K-DIFLRMDELESHMGTLHSNFGELKLKIKELLEENQRLRIENEQLRKVLKRG------AEP-AELQ-HTA-DEPA-AVKGD----K-LPG---A-MG----------------------------VGEGYDNLARLYHEGFHICNVYYGHLRMEGDCLFCLSFLSK-- 120

WP_036651655_1 ----------MEKK-NIFAHMQDMEAQMESMRTNLSNWKQTVKELMEANQRLSLENEQLRKILKRE-----APIP-SDSP-AAD-SADP-AAILT----TGEGT---G-EV----------------------------VGEGYDNLARLYHEGFHICNVYFGHLRTEGDCLFCLSFLNK-- 124

WP_025694070_1 ----------MEKK-NIFAHIQEIEAQMETMRLNLGDWKQAVKELMEANQRLTLENEQLRKILKKK--A-----P-PASS-SEK-RNSG-KLPVQ----NEGGQ---D-EI----------------------------VGEGYDNLARLYHEGFHICNVNFGHLRTEGDCLFCLSFLNK-- 122

WP_042204553_1 ----------MEKK-NIFAHIQEIEAQMETMRLNLGDWKQAVKELMEANQRLTLENEQLRKILKKK--A-----P-PGSS-SEK-RNPG-KLPVQ----NESGQ---D-EI----------------------------VGEGYDNLARLYHEGFHICNVNFGHLRTEGDCLFCLSFLNK-- 122

WP_025332572_1 ----------MEKK-NIFAHIQELETQMETMRLNLGDWKQAVKELMEANQRLTLENEQLRKILKRE--A-----P-SGPS-SEK-KAAG-KPPAP----SEITS---D-EV----------------------------VGEGYDNLARLYHEGFHICNVNFGHLRTEGDCLFCLSFLNK-- 122

WP_044879177_1 ----------MEKK-NVFQRMQEMEAQMGQMQSNLGELKLVIKELLEDNNRLLLENEQLRKILKRE------VQP-AE-N-GVI-TSLP-LHASE----DIILP---E-DI----------------------------VGEGYDNLARLYHEGFHICNVYYGHLRTEGDCLFCLSFLNK-- 122

WP_036653262_1 ----------MEKK-NIFVQIQEMETQMGQLHSDLGEMKLVIKELLEENNRLMLENERLRKILKEA--L-----P-VEEG--SL-LPKP--DVLL----ENDQP---S-DV----------------------------VGEGYDNLARLYHEGFHICNVYYGHLRTEGDCLFCLSFLNK-- 120

WP_068651579_1 ----------MEKK-NVFLRMQEMEEQMEHMHSDLGELKLLVKELLEENNRLVLENERLRIILKRE--A---QLM-EERV-ASSIPHHP-HEVL-------------E-DV----------------------------VGEGYDNLARLYHEGFHICNVYYGHLRTEGDCLFCLSFLNK-- 119

WP_025706635_1 ----------MEKM-NIFAHMQEMEAQMESMRASLGEWKLTVKELMEANQRLSLENEQLRKILKRE--A-----P-LDPA--AH-SAEA-EALLA----ASEGV---E-EV----------------------------VGEGYDNLARLYHEGFHICNVYFGHLRTEGDCLFCLSFLNK-- 121

WP_036678835_1 ----------MEKK-NIFAHMQDMEAQMETMRANLGEWKQTVKELMEANQRLTLENEQLRKILKRD--A-----P-LDPT--VD-SAEA-EALLA----TGEGT---E-EV----------------------------VGEGYDNLARLYHEGFHICNVYFGHLRTEGDCLFCLSFLNK-- 121

WP_038584339_1 ----------MEKI-NIFAHMQEMEAQMESMRASLGDWKQTVKELMETNQRLSLENEQLRKILKRD--A-----P-LDPA--VD-TAEA-EALLA----AREGT---E-EV----------------------------VGEGYDNLARLYHEGFHICNVYFGHLRTEGDCLFCLSFLNK-- 121

WP_039787597_1 ----------MEKM-NIFAHMQEMEAQMESMRASLGEWKLTVKELMEANQRLSLENEQLRKILKRE--A-----P-LDPV--AH-SAEA-EALLA----ASEGT---E-EV----------------------------VGEGYDNLARLYHEGFHICNVYFGHLRTEGDCLFCLSFLNK-- 121

WP_039868839_1 ----------MEKK-NIFAHMQEMEAQMETMRATLGDWKQTVKELMEANQRLSLENEQLRKILKRE--A-----P-LDPA--VD-TAEA-EALLA----AAEGT---E-EV----------------------------VGEGYDNLARLYHEGFHICNVYFGHLRTEGDCLFCLSFLNK-- 121

WP_042123089_1 ----------MEKK-NIFAHMQDMEAQMETMRANLGEWKQTVKELMEANQRLTLENEQLRKILKRD--A-----P-LDPT--VD-SAEA-EALLA----AGEGT---E-EV----------------------------VGEGYDNLARLYHEGFHICNVYFGHLRTEGDCLFCLSFLNK-- 121

WP_087918982_1 ----------MEKK-NIFAQMQDMEAQLEHMRATLGDWKQTVKELMEANQRLNLENEQLRKILKRE--A-----P-LDPK--VD-SAEA-EALLA----AREGA---E-EV----------------------------VGEGYDNLARLYHEGFHICNVYFGHLRTEGDCLFCLSFLNK-- 121

WP_039305729_1 ----------MEKK-NIFAHMQEMEAQMDEMRATLGDWKQTVKELMEANQKLSLENEQLRIILKRE--A-----P-LDKA--AL-SAEA-EAILA----A-EGK---E-EV----------------------------VGEGYDNLARLYHEGFHICNVYFGHLRTEGDCLFCLSFLNK-- 120

WP_042209904_1 ----------MEKK-NIFAHMQEMEAQMDEMRTTLGDWKQTVKELMEANQKLSLENEQLRIILKRE--A-----P-LDKA--AL-SAEA-EAILA----A-EGK---E-EV----------------------------VGEGYDNLARLYHEGFHICNVYFGHLRTEGDCLFCLSFLNK-- 120

WP_076176647_1 ----------MEKK-NIFAHIQEMETQMEQVHSDLGEMKLLIKELLEENNRLSLENEQLRKILKGA--I-----P--EEG--SP-LPQPAHATAE----QEHEI---E-DV----------------------------VGEGYDNLARLYHEGFHICNVYYGHLRTEGDCLFCLSFLNK-- 121

WP_068661303_1 ----------MEKR-NVFLRMQEMEAQMEHLHSDLGDLKLLVKELLEENNRLALENERLRIILKRE--A---QLQ-EDGD-PSP-TNHP-HVIP-------------E-DV----------------------------VGEGYDNLARLYHEGFHICNVYYGHLRTEGDCLFCLSFLNK-- 118

WP_017691386_1 ----------MEKK-NLFTHIHEMETQLGQLHGDLGELKMIVKELLEDNQRLTIENEQLRKLLKRE------A-P-ADLP-ISS-ALAP-VARPA----GPATG---E-DV----------------------------VGEGYDNLARLYHEGFHICNVYYGHLRTEGDCLFCLSFLNK-- 122

WP_024633697_1 ----------MEKK-NLFTHIHEMETQLGQLHSDLGELKMIVKELLEDNQRLTIENEQLRKLLKRE------A-P-ADLP-IAS-ALAP-VARPA----GPATG---E-DV----------------------------VGEGYDNLARLYHEGFHICNVYYGHLRTEGDCLFCLSFLNK-- 122

WP_006207404_1 ----------MEKK-NIFVLIQELETQMGHIHSELGGLKLVIKELLEENHRLSLENEQLRKILKRE------VNP-AEFE-NPV-AIQP-QPHVK----DEEHG---E-DV----------------------------VGEGYDNLARLYHEGFHICNVYYGHLRTEGDCLFCLSFLNK-- 123

WP_077570918_1 ----------MEKK-NIFVLIQELESQMGHIHSELGGLKLVIKELLEENHRLSLENEQLRKILKRE------VNP-AELE-HPV-AAMP-LPHAE----KEDQE---E-DV----------------------------VGEGYDNLARLYHEGFHICNVYYGHLRTEGDCLFCLSFLNK-- 123

WP_012818303_1 ----------MEKK-NIFVLIQELETQMGHIHSELGGLKLVIKELLEENHRLSLENEQLRKILKRE------VNP-AELE-SPV-ALQP-QPHVK----DEEH----E-DV----------------------------VGEGYDNLARLYHEGFHICNVYYGHLRTEGDCLFCLSFLNK-- 122

WP_068699013_1 ----------MEKI-DIFAHLQQMESQMGEAQTELGALKLEVKKLLEENQRLTTENEQLRRVLKRE--T-----G--EPGSDLV-AGKE-AEAAS----EEERK---A-DV----------------------------IGEGHDNLARLYHEGFHICNVYYGHLRTEGDCLFCLSFLNK-- 122

WP_068621680_1 ----------MEKK-DIYTNLLDLESKMESLHSELGILKLEVKKLLEENQRYSLENEQLRKVLKRE-----AEAG-GQPP-VQT-ESHP--ELHE----HGEHI---DIDI----------------------------VGEGYDNLARLYHEGFHICNVYYGHLRTEGDCLFCLSFLNK-- 124

WP_044482620_1 ----------MENK-DIFKHVQALETQVGKFHGDLGALMLEVKRLVEENQQLRLENEKMRKIMMGE------AKE-AAPA-STS-SSRS-AAPST----GGKEA---V-DV----------------------------VGEGYDNLARLYHEGFHICNVYYGHLRTEGDCLFCLSFLNK-- 123

WP_108463744_1 ----------MEKR-DIFTHLHELELQMGTAQEELSVLKLEVKKLLEENQRLSLENEQLRKILKRE--A-----G--EPG-DVI-PTEG-EAVSG----ETEEG---E-DI----------------------------VGEGYDNLARLYHEGFHICNVYYGHLRTEGDCLFCLSFLNK-- 121

WP_036618150_1 ----------MENK-DIFNHMQALESQMGKVHGELGSLMLEVKKLVEENQRLLLENEQLRKILKRE------SAE-SRVP-LTP-AGKA-ADPVL----AGKEA---I-DV----------------------------VGEGYDNLARLYHEGFHICNVYYGHLRTEGDCLFCLSFLNK-- 123

WP_016314715_1 ----------MENK-DIFTSMQALETQMGKVHGDLSSLMLEVKKLVEENQRLRLENEQLRKILKRE------STE-GPLP-MPA-SVRT-DSPVP----AGKEA---I-DV----------------------------VGEGYDNLARLYHEGFHICNVYYGHLRTEGDCLFCLSFLNK-- 123

WP_015256479_1 ----------M-TR-DLFDQVERMEAVLGQMLRETADIKRNIKSLIEENKRLSIENQQLRKLLKTS--A---V-T-VRAE-LAA-AGLA-EEAS--ADRRDEES---H-VH----------------------------VGEGYDNLARLYNEGFHICNVYYGHLRTEGDCLFCLSFLHK-- 124

WP_040953016_1 ----------MDSK-EIFQHMEEIEDKMGLTYADIGVLKKKIVLLLEENNRLEMENRQLRGLLQQN---------------GPA--PEK----------EPEPR---E-ST----------------------------VGEGHDNLARLYNEGFHICNVYYGHLRTEGDCLFCLSFLNK-- 110

WP_068605371_1 ----------MEKR-DVFAAIDEIEGQMGNVHAELGSLKKQVIHLLEENKRLSLENQHLRKLLRLEELPEGAPSE-QRAQ-DQR-PAVP-ELVVE----D---------AV----------------------------VGEGYDNLARIYNEGFHICNVYYGHLRTEGDCLFCLSFLNK-- 124

WP_023482256_1 ----------MDKQ-DIFVEVGQIEEQMGKAYEELGALKKKIIALLEENKHLMLENQQLRAVLQEK---------------ENE-YIQR----------TSKED---V-PS----------------------------IGEGHDNLARLYNEGFHICNVYYGHLRTEGDCLFCLSFLNK-- 111

WP_042235332_1 ----------MEKH-EMFAQIDGLEEQIGKAYEELGDLKKTIISLLEDNQRLMMENQQLRKLLKQG---------------DAE-ETDS----------EPEKG-I-E-PV----------------------------IGEGYDNLARLYYEGFHICNVYYGHLRTEGDCLFCLSFLNK—- 112

**paen9.02368, Translation initiation factor IF-2**

|  |
| --- |

WP_042138118_1 MTKE-----D--N-KD-KLRVYEYAKSLNMSSKEIITILKRLNVPVNNHMSVMENGSVNKVEQFFKDIKSNAAAKRDT-GTSSRPVTTG---------AVTA--------EPQSAQNAN-K--NQPEK----QV------------G-----MNSNQN------NNQSTTSPRPQSGQDSRRTQT--------------- 128

WP_042214813_1 MTKE-----D--N-KD-KLRVYEYAKSLNMSSKEIITILKRLNVPVNNHMSVMENGSVNKVEQFFKDIKSNAAAKRDT-GTSSRPVTTG---------AVTA--------EPQSAQNAN-K--NQPEK----QV------------G-----MNSNQN------NNQSTTSPRPQSGQDSRRTQT--------------- 128

WP_042237107_1 MTKE-----D--N-KD-KLRVYEYAKSLNMSSKEIITILKRLNVPVNNHMSVMENGSVNKVEQFFKDIKSNAAAKRDT-GTSSRPVTTG---------AVTA--------EPQSAQNAN-K--NQPEK----QV------------G-----MNSNQN------NNQSTTSPRPQSGQDSRRTQT--------------- 128

WP_039874189_1 MTKE-----D--N-KD-KLRVYEYAKSLNMSSKEIITILKRLNVPVNNHMSVMEDGSVNKVEQFFKDIKSNAAAKRDT-GTSSRPVSTG---------TVTA--------EPQSAQNAN-K--NQPEK----QV------------G-----MNSNQN------NNQSTTSSRPQSGQDSRRTN---------------- 127

WP_038593413_1 MTKE-----D--N-KD-KLRVYEYAKSLNMSSKEIITILKRLDVPVNNHMSVMENGSVTKVEQFFKDIKSNAAAKRDP-GTSSRPVTTG---------SVTA--------EPQSAQNAN-K--NQPEK----QV------------G-----MNSNQN------NNQSTTSPRPQSGQDSRRTT---------------- 127

WP_042177491_1 MTKE-----D--N-KD-KLRVYEYAKSLNMSSKEIITILKRLNVPVNNHMSVMEDGSVNKVEQFFKDIKSNAAAKRDT-GTSSRPAGAG---------TVTA--------EPHSAQNAN-K--NQPEK----QV------------G-----MNSNQN------NNQSTTSSRPQSGQDSRRTN---------------- 127

WP_036688463_1 MTKE-----E--N-KD-KLRVYEYAKSLNMSSKEIITILKRLNVPVNNHMSVMENGSVNKVEQFFKDIKSNAAAKRDT-GTSSRPVTTG---------AVTA--------EPQSAQNAN-K--NQQEK----QV------------G-----MNSNQN------NNQSTTSPRPQSGQDSRRTQ---------------- 127

WP_042266949_1 MTKE-----D--N-KD-KLRVYEYAKSLNMSSKEIITILKRLNVPVNNHMSVMENGSVSKVEQFFKDIKSNAAAKRDS-GTSSRPASAG---------TVTA--------EPQSAQNAN-K--NQPEK----QV------------G-----MNSNQN------NNQSTTSPRPQSGQDSRRAT---------------- 127

WP_042128858_1 MTKE-----E--N-KD-KLRVYEYAKSLNMSSKEIITILKRLNVPVNNHMSVMENGSVNKVEQFFKDIKSNAAAKRDT-SSSSRPVTTG---------AVTA--------EPQSAQNAN-K--NQQEK----QV------------G-----MNSNQN------NNQSTTSPRPQSGQDSRRTQ---------------- 127

WP_042189415_1 MTKE-----E--N-KD-KLRVYEYAKSLNMSSKEIITILKRLNVPVNNHMSVMENGSVNKVEQFFKDIKSNAAAKRDT-SSSSRPVTTG---------AVTA--------EPQSAQNAN-K--NQQEK----QV------------G-----MNSNQN------NNQSTTSPRPQSGQDSRRTT---------------- 127

WP_046504296_1 MTKE-----D--N-KD-KLRVYEYAKSLNMSSKEIITILKRLNVPVNNHMSVMENGSVSKVEQFFKDIKSNAAAKRDS-GTSSRPASAG---------TVTA--------EPQSAQNAN-K--NQPEK----QV------------G-----MNSNQN------NNQSTTSPRPQSGQDSRRAT---------------- 127

WP_016362856_1 MTKQ----DN--N-KD-KLRVYEYAKSLNMSSKEIITILKRLGRPVNNHMSVMENDAVDSVEKFFKDVKANAAAKRSE-NNAV---------------PASN--------KTVAVTPKP-E------Q----RS------------G-----QESAPA------RSSEGSQ-ESRPAAERSEGGP--------------- 118

WP_068608410_1 MSKQ-----DKDN-KD-KLRVYEYAKSLNMSSKEIITILKRLDLPVNNHMSVMENDSVSRVEGFFRDIKANAAAKRAT-ETSSKSSQQAPSKT-----ADKS-V------ASSPSQPKQ-E--DQPKT----TISSASRDVSSPATG-------ASSG------SGNSTQDRNNDRNQGERR------------------ 142

WP_062408002_1 MTKQ----DN--N-KD-KIRVYEYAKSLNMSSKEIITILKRLGKPVNNHMSVMENDAVESVEKFFKDVKANAAAKRND--------------------GAAA--------AKSADKPAG-R---------------------------------QEPK------QQTVAVQSKPEQRKSAAQVQ---------------- 108

WP_005550539_1 MSKQ----EN--T-KD-KTRVYEYAKSLNMSSKEIITILKRLNIPVNNHMSVMENDAVGKVESYMRTIKENAQAKREG-NSTSQPQVNISQTKT----SEVP--------DKTASQPAR-K-TSQQAD----QR------------DSH---AGQQQQ------MQRDGRDHQREGNRGGRNERQ--------------- 137

WP_015254924_1 MSKQ-----E--N-KD-KLRVYEYAKSQNMSSKEILTILKRLGLQVNNHMSVMEDHMVEKVEGFFRSIKENAAAKRAQ-QELQAAQQRQ---------AKAQ-E------APRQQGAPS-A--NRPAT------------------------VNAAPQ------TNAAGAQRRGDRPQSGQN---------------A-- 124

WP_042233063_1 MGLT--NKQD--P-KD-KLRVYEYAKSLNMSSKEIITILKRLNIPVNNHMSVMENNAVSSVENFFRDIKANAAAKRAG-SDGGNVSPNS---------NQPK--------PDKQSNPKA-E-------------------------------VSAKQP------VQQTGSEQAKKEERPAAEARP--------------- 123

WP_040948716_1 MNLS---KQD--N-KD-KLRVYEYAKSLNMSSKEIITILKRQGITVNNHMSVMENDAVGAVERFFHDVKANAAARRAAGESGPAKSGGSQAAQQGASQSPRQ--------NAAPAQPAA-Q-QPQAAE----RS------------S-------SQPQ------AAVSGQSPQPARSEQAEQTSP--------------- 139

WP_087432880_1 MTKQ-EK--D--N-KD-KLRVYEYAKSLNMSSKEIITILKRINIPVNNHMSVMENDAVAKVEQFFKDVKANAQAKREG-GPQSKPQAQA---------QVSQ-V------SSKTAETPK-R--TENSS----KE------------N-----NDNKQR---Q--GQREGQRHPQRDGRDQQR------------------ 129

WP_036579329_1 MTKQ-----D--N-KD-KLRVYEYAKSLNMSSKEIITILKRLDLPVNNHMSVMENEMVSRVEGFFRNIKENAAAKRAQ-EGS----------------GAGR--------QQAAGKPSG-D-RPQAAA----RS------------NE----PQASLR------SNTLQSGERPAQGQQSGGQRP--------------- 123

WP_068650597_1 MTKQ-----ES-N-KE-KFRVYEYAKSLNMSSKEIITILKRLDLPVNNHMSVMENDAVTKVEKFFENIKSNAAAKRDS-GDNHAMAAT----------SSVK--------KDAA------V--VKPNE----AS------------V-----SLQENS------TVPVQSETKKQQEKQVGM------------------ 120

WP_046230755_1 MNKP-QDSKD--N-KD-KLRVYEYAKSLSMSSKEIITILKRLDMPVNNHMSVMENEMVNKVEGFFRDIKANAAAKRAK-ESSGATVSST---------SQAP-S------KPAQPKQQP-V--SQQVS-I--KP------------E-----LEASGL------NNKKITQDRQEPMNSIKTT----------------- 132

WP_099520650_1 MNKP-QDSKD--N-KD-KLRVYEYAKSLSMSSKEIITILKRLDMPVNNHMSVMENEMVNKVEGFFRDIKANAAAKRAK-ESSGATVSST---------SQAP-S------KPAQPKPQP-V--SQQEA-I--KP------------E-----LEASGL------NNKKITQDRQEPMNSIKTT----------------- 132

WP_036622334_1 MSKQ-----E--K-QD-KLRVYEYAKSLNMSSKEIITILKRLNIPVNNHMSVMENDAVTRVEQFFKDIKTNAAAKREN-SGRAETPSK----------SPAP--------ASAGGSQAA-K--NPAPE----GA------------G-----NRGEQGREAKVHGNPGGQAKKNQHEKQVSM------------------ 130

WP_082083971_1 MTKP-TESKD--N-KD-KLRVYEYAKSLNMSSKEIITILKRLDLPVNNHMSVMENEMVGKVEGFFRDIKANAAAKRAK-DGATVSA------------TAAA--------AAGQTRAGT-E---KRQQ----QQ------------G------VHNIQ------TQQSSNKNQQDNQVTMNTTQS--------------- 127

WP_079940754_1 MSNK-----Q--NSKD-KLRVYEYAKSLNMSSKEIITILKRLNIPVNNHMSVMENEAVHSVEKFFRDIKANAAAKRAV-NDGGHENLNQ---------STTN-Q------EPKQEQSKS-Q--KQGAN------------------------TSAAP-----------GARRNQQNGQQRQN------------------ 119

WP_006036631_1 MTKQPDSKDN--N-KD-KLRVYEYAKSLNMSSKEIITILKRLDLPVNNHMSVMENQMVSKVEGFFRDIKQSAAVKRAS-ESATVSSAATQRQPEHRKPSAAS--------EQAAVQPSS-EPKNLIQD----RQ------------GTMNSIKTTTNT------SNQQGDQQEPREEQRNSAQQP--------------- 149

WP_089523112_1 MTKP-QDGKE--G-KE-KLRVYEYAKSLNMSSKEIITILKRLDTPVNNHMSVMESGMVGKVEGFFRDIKANAAAKRAQ-GNTQANTAATRPAEK----RPEQ--------QGVHTIPRQ-E-QSQPTN----QQ------------DRQVTMNTTTNN------QGSNSTEQRPSQGSNSTEQRP--------------- 143

WP_016312402_1 MSKQ-----E--K-QD-KLRVYEYAKSLNMSSKEIITILKRLNIPVNNHMSVMENDAVARVEQFFKDIKSNAAAKRES-SGRNDSSAK----------SPAS--------ASAGGSQTP-K--ATAGE----SS------------G-----HRGDQGR-----GANAPVKNQNQQEKQVSM------------------ 125

WP_037282270_1 MSKP----------ES-KVRVYEYAKSLDMSSKEIITILKKLEMPVNNHMSVMEPEAIGKVEQFFRKIKSNAS-------------------------KPAS--------PSSKPQSNG-D-----------------------------S-QIKNSQ------EKQVGMNNKPQTPSSSQ------------------- 100

WP_068694645_1 MSKQ-----E--N-KD-KLRVYEYAKSLNMSSKEIITILKRLDIPVNNHMSVMENDAVSKVEQFFKDIKNNAAAKRDS-AAASG--------------GGQA--------KAASVSSSG-N--------------------------------GRTPE------SKSQTQAGQDNSAIRSTSNNK--------------- 114

WP_055108420_1 MSKQ-----E--N-KD-KLRVYEYAKSLNMSSKEIITILKRLNIPVNNHMSVMENDAVSKVEQFFKDIKSNAAAKREG-SEPAKSSAK----------SSAP--------ASAGGSQTP-K--TGAGA----DA------------G-----QREEQGQGHRA-GSSGSGNTKNQQEKQVSM------------------ 129

WP_068619253_1 MSKQ-----D--N-KD-KLRVYEYAKSLNMSSKEIITILKRLDIPVNNHMSVMENDAVSRVEKFFKDIKTNAAVKKEG-SDSRVAQAK----------SPAP--------VSAGGDPKP-N--TQTRE----QG------------G-----QRSEQA------VQRNEDSNKNQQEKQVRM------------------ 124

WP_015844984_1 MSKQQDNGKD--N-KD-KTRVYEYAKSLNMSSKEIITILKRLNLPVNNHMSVMENEMVHKVEGFFRDIKQNAAAKRAQ-ESGSATVSAAPQQQ-----AQNS--------KPQQNQTVQ-K--NLSQD----RQ------------GPMNS-IKTTSE------TNQSQQEQRPQSQQETTSN----------------- 139

WP_091181685_1 MSKQQDNSKD--N-KD-KTRVYEYAKSLNMSSKEIITILKRLNLPVNNHMSVMETEMVNKVEGFFRDIKQNAAAKRAQ-ESGSATVSAAPQPQA----QSAN--------KPQSSQPVQ-K--NLSQD----RQ------------GPMNS-IKTTSE------TNQSQQEQRPAQSQQENSTPT--------------- 142

WP_044877552_1 MSKQ-----D--N-KD-KTRVYEYAKSLNMSSKEIITILKRLDIPVNNHMSVMESESVHKVEQFFKNIKSNAAAKRDS-GDNRVTAST----------SSSN--------NGSAGTAKVET--PDPKQ----ET------------V-----STSAVN------SVNDQEQSKKQQEKQVGM------------------ 125

WP_094155440_1 MSKQ-----E--S-KD-KVRVYEYAKSLNMSSKEIITILKRLDIPVNNHMSVMEHDAVGKVENFFKNIKSNAAAKQGG--------------------ADTA--------QVSSNQAGS-S-QNGSSRTGSSQT------------G-------SNQN------SNHSTEQTKNQQEKQVSM------------------ 118

WP_014281121_1 MSKQ-----E--S-KD-KVRVYEYAKSLNMSSKEIITILKRLDIPVNNHMSVMEHDAVGKVEKFFKDIKSNAAAKQGG--------------------AGTA--------QVSSN-----------------QA------------G-------SSQN------SNHSTEQTKNQQEKQVSM------------------ 103

WP_023988217_1 MSKQ-----E--S-KD-KVRVYEYAKSLNMSSKEIITILKRLDIPVNNHMSVMEHDAVGKVESFFKNIKSNAAAKQGG--------------------AGTA--------QVSSN-----------------QA------------G-------SSQN------SNHSTEQTKNQQEKQVSM------------------ 103

WP_028542714_1 MSKQ-----E--S-KD-KVRVYEYAKSLNMSSKEIITILKRLDIPVNNHMSVMEHDAVGKVESFFKNIKSNAAAKQGG--------------------AGTA--------QVSSN-----------------QA------------G-------SSQN------SNHSTEQTKNQQEKQVSM------------------ 103

WP_071640528_1 MSKQ-----E--S-KD-KVRVYEYAKSLNMSSKEIITILKRLDIPVNNHMSVMEHDAVGKVESFFKNIKSNAAAKQGG--------------------AGTA--------QVSSN-----------------QA------------G-------SSQN------SNHSTEQTKNQQEKQVSM------------------ 103

WP_013370722_1 MSKQ-----E--S-KD-KVRVYEYAKSLNMSSKEIITILKRLDIPVNNHMSVMEHDAVGKVENFFKNIKSNAAVKQGE--------------------AGTA--------QVSSN-----------------QT------------G-------SSQN------SNHSTEQTKNQQEKQVSM------------------ 103

WP_016820981_1 MSKQ-----E--S-KD-KVRVYEYAKSLNMSSKEIITILKRLDIPVNNHMSVMEHDAVGKVENFFKNIKSNAAVKQGE--------------------AGTA--------QVSSN-----------------QT------------G-------SSQN------SNHSTEQTKNQQEKQVSM------------------ 103

WP_068655661_1 MTKQ-----DK-A-QE-KLRVYEYAKSLNMSSKEIITILKRLDIPVNNHMSVMETDSVSKVEQFFKNIKSNAAAKRDS-GEQHAVAAT----------SSVK--------KDEI------A--VKGTV----VA------------D-----TQSDKN------TVTEAVETKKQLEKQVGM------------------ 120

WP_060534524_1 MSTQ-----E--Q-KN-KLRVYEYAKSLNMSSKEIITILKKLNVPVNNHMSVMENDAVTKVEGFFKNIKSTAAEQQGN--------------------GQST--------IADASSTSR-E-KNRQEK----QV------------G-------MDIK------NQNNSTNGTTRSQSGTSS------------------ 114

WP_074094581_1 MSKQ-----E--N-KD-KLRVYEYAKSLNMSSKEIITILKKLEIPVNNHMSVMENGSVGKVEQFFKDIKSTAASKQGN-DAKPVATSAV---------RSDK--------PVDSNKPAG-G--------V--NT------------P-------ISSN------PSGSPVQTKIQQEKQVGM------------------ 119

WP_062326090_1 MSKQ-----E--N-KD-KLRVYEYAKSLNMSSKEIITILKKLEIPVNNHMSVMENGSVGKVEQFFKDIKSTAASKQSS-EAKSVATSSV---------RSDK--------TVDSNKPAG-G--------A--NT------------P-------KSSN------TSGSPVQTKIQQEKQVGM------------------ 119

WP_015736303_1 MSKQ-----E--N-KDNKLRVYEYAKSLNMSSKEIITILKRLNVPVNNHMSVMENDTVSKVEQFFKDIKSNAAAKRDS-GDAPRTENRKPSGSGAN--AAASVSQAKTEGTSTNVNADG-N-RNGQDG-MR-HS------------G-------QRVS------GQQGQSQPKKQQEKQVGM------------------ 142

WP_036642094_1 MSKQ-----E--N-KDNKLRVYEYAKSLNMSSKEIITILKRLNIPVNNHMSVMENDTVSKVEQFFKDIKSNAAAKRDS-SESSRNENRKPSVSGAS--PAAAVSNIKSEGTTSNVSTEG-S-RNGQDG-MR-NS------------G-------QRAP------GQQGQSQPKKQQEKQVGM------------------ 142

WP_108465278_1 MSKQ-----D--N-KD-KLRVYEYAKSLNMSSKEIITILKRLDIPVNNHMSVMENSAVARVEQFFKDIKSSAAVKKAG-SGSVE--------------ASAQ--------SAVSASAGG-S---------------------------------HKPN------TSGSSSGSQRHGASISA------------------- 109

WP_036651540_1 MSKQ-----D--S-KE-KLRVYEYAKSLNMSSKEIITIMKRLDIPVNNHMSVMENGTVTKVEQFFKDIKSNAAAKRES-G-DNKPVTSAPNVQ-----KDTS-VVRNLG-TGSSGQPQS-Q--KQQEK----QV------------G-------MNNN------TNNTTQSGSQRPQGGQDS------------------ 132

WP_099479276_1 MSKQ-----E--N-KDNKLRVYEYAKSLNMSSKEIITILKRLNIPVNNHMSVMENDTVSKVEQFFKDIKSNAAAKRDG-ADASRGESRKPPVSVAS--SSSA--------ADSKVSS-------GQDG-SR-NA------------G-------QR-----------GQGQPKKQQEKQVGM------------------ 124

WP_076168052_1 MSKQ-----D--S-KD-KLRVYEYAKSLNMSSKEIITILKRLDIPVNNHMSVMENDAVSKVEQFFKDIKSNAAAKREG-GD-----------------------------RQTVASTSA-V-RNEAGT-QR-NQ------------G-------GRAA------GSD---PSQKQQEKQVGM------------------ 111

WP_038696662_1 MTKQ----QD--S-KD-KLRVYEYAKSLNMSSKEIITILKRLNVPVNNHMSVMENDAVSKVEQFFKDIKSNAAAKREN-AGGGRPV------------QQAA--------TATADAPAN-N-KNQTEK----QV------------G------MNKPN------QNQSTMSSRPQSGQDSRRN----------------- 124

WP_042207090_1 MTKQ-----D--S-KD-KLRVYEYAKSLNMSSKEIITILKRLNVPVNNHMSVMENDAVSKVEQFFKDIKSNAAAKRES-GGASRSA------------SSAT--------ATVEAKSVD-K--NQIEK----QV------------G------MNKPH------NNSSTMSSRPQSGQDSRR------------------ 121

WP_082210233_1:7-883 LTKQ-----D--S-KD-KLRVYEYAKSLNMSSKEIITILKRLNVPVNNHMSVMENDAVSKVEQFFKDIKSNAAAKRES-GGGGRPA------------STAT--------ATVEVKNVN-Q--NQLEK----QV------------G------MNRPN------NNSSTTSSRPQSGQDSRR------------------ 121

WP_025335323_1 MTKQ-----D--S-KD-KLRVYEYAKSLNMSSKEIITILKRLNVPVNNHMSVMENDAVSKVEQFFKDIKSNAAAKRES-GGGSRPN------------QTAT--------ATIEAQSSN-K--NQTEK----QV------------G------MNKPN------NNSSTMSSRPQSGQDSRRN----------------- 122

WP_036656399_1 MSKE-----D--N-KD-KLRVYEYAKSLNMSSKEIITILKRLDVPVNNHMSVMENGSVNKVEQFFKDVKSNAAAKRDS-GPSSRPVNTS---------APAA--------DSAKAQNVN-Q--NQPEK----QV------------G-----MNSNPN------NNPSTTSPRPQSGQDSRSTQ--TT------------ 129

WP_087920295_1 MTKE-----D--N-KD-KLRVYEYAKSLNMSSKEIITILKRLDVPVNNHMSVMENGSVNKVEQFFKDVKSNAAAKRDT-GTSSRPAAAG---------AVTA--------EPASAQNAN-K--NQPEK----QV------------G-----MNSNQN------NNQSTTSPRPQSGQDSRSAQSGTTQNSRPQQSGAPR 143

WP_042138118_1 TGTAQNSRP--Q--GSSTG-SRPQ-G-S---------S-TG---SRPQGS--S--T-TGSRP--Q---GS-----------ST--GG-SRPQGS----------------------STG----GS--RP-QG-------S----------STGGSRPQG--SST----TG-----------SR-PQG--S-STGGS---R 214

WP_042214813_1 TGSTQNSRP--Q--GSSTG-SRPQ-G-S---------S-TG---SRPQGS--S--T-GGSRP--Q---GS-----------ST--GG-SRPQGS----------------------STG----GS--RP-QG-------S----------STTGSRPQG--SST----TG-----------SR-PQG--S-STTGS---R 214

WP_042237107_1 TGTTQNSRP--Q--GSSTG-SRPQ-G-S---------STTG---SRPQGS--S--T-GGSRP--Q---GS-----------ST--G--SRPQGS----------------------ST-----GS--RP-QG-------S----------STTGSRPQG--SST----GG-----------SR-PQG--S-STGGS---R 213

WP_039874189_1 SGSTQNSRP--Q--GSSTG---------------------G---NRPQGS--S--T-GGNRP--Q---GS-----------ST--GG-NRPQGS----------------------STG----GN--RP-QG-------S----------STGGNRPQG--GST----GG-----------NR-PQG--S-SAGGN---R 205

WP_038593413_1 TGSTQNSRP--Q--GSSTG-SRPQ-G-S---------S-TG---SRPQGS--S--T-SSSRP--Q---GS-----------ST--SG-SRPQGS----------------------STG----GS--RP-QG-------S----------STGGNRPQG--SST----GG-----------NR-PQG--S-STGGN---R 213

WP_042177491_1 SGSTQNSRP--Q--GSSTG---------------------G---NRPQGS--S--T-GGSRP--Q---GS-----------ST--GG-NRPQGS----------------------STG----GN--RP-QG-------S----------STGGNRPQG--GST----GG-----------NR-PQG--G-STGGN---R 205

WP_036688463_1 TGSTQNARP--Q--QSGA--PRTN-S-T---------S--STSSSRPQSS--T--T-GGNRP--Q---GS-----------NT--GG-SRPQGS----------------------NTG----GN--RP-QG-------S----------NTSGSRPQG--SNT----GG-----------SR-PQG--S-NTGGS---R 214

WP_042266949_1 SGSAQNSRP--Q--SSTSG-NRPQ-G-G---------S-TG---SRPQGS--S--T-GGNRP--Q---GS-----------ST--G--SRPQGS----------------------ST-----GS--RP-QG-------S----------ST-----------------G-----------SR-PQG--S-ST-GS---R 199

WP_042128858_1 TGSTQNARP--Q--QSGA--PRTN-S-T---------S--G---SRPQGS--S--T-GGNRP--Q---GS-----------NT--GG-SRPQGS----------------------NTG-----S--RP-QG-------S----------STGGSRPQG--SNT----GG-----------SR-PQG--S-NTGGS---R 210

WP_042189415_1 TGSTQNARP--Q--QSGA--PRTN-S-T---------S--G---SRPQGS--S--T-GGNRP--Q---GS-----------NT--GG-SRPQGS----------------------NT-----GS--RP-QG-------S----------STGGSRPQG--SST----GG-----------SR-PQG--S-NTGGS---R 210

WP_046504296_1 SGSTQNSRP--H--SSTGG-------------------------NRPQGS-----T-NGSRS--Q---GS-----------SN--NG--RPQGN----------------------STG----GS--RP-QG------------------NSAGNRSQG---ST----NG-----------SR-PQG--S-ST-GS---R 199

WP_016362856_1 SRGGQGGSG--G--GQGG--GGYR-G-G---------Q-GG-G-GYRGGQ--G----GGGQG------GG---GYRG-GQGGS--GG-GQGGGGYRGGQGGSSGGGQGGGGYRGGQGGS----GGG-QG-GGGY-----RGGQGGSSGGGQGGGGYRGG--QGG--SSGG----GQGGGGYRGGQGG--G-GYRGG---Q 254

WP_068608410_1 SQPTERQGH--M--NSTR--SNDRNS-S--G------N-YN-Q-NRTGTS--G----TGTST--N---RT------------G--QS-TTGTGS------------QSGGQRTYSGQSS-TG-GA--RTYSG-Q-----G---S------QGGQGSRTS--SGT--------------------SSQ--S-GGRTY---S 239

WP_062408002_1 SAATRPSNA--P--SSGR--PQGQ-G-G---------G-QS-G-RPQGGQ--G----QGGPS------SS------------------NRPQGD----SRQGQGQHRQSGQ-----GNQ----GGG-RP-QG------------------ERRGPAGSG--QGQ--------------------GQG--G-GFNRG---G 197

WP_005550539_1 ERITRGGGH--D--GQKD--HNRQ-G-E---ININM-D-TR-Q-GNQQGN--M----NGQEQ------RR--SGEQQ----HN--QG-NRSNGQ----QRQGQGAPRQGGGPR---QGQ----GGN-SR-PG------------------QGGAPRQGG--QGN-----G--------------PRQ--G-GASRP---G 243

WP_015254924_1 QGGGRSDRP-----QAGQ-------G-E---------R-------------------RGDRP--Q---GG---------------RN-DRPQGG-----------RPQGGQ---G-DRR----GD--RP-QS---G---Q-AHG------GGRNDRPQG--GRN-----------------DR-PQG--G-RGDRPQGGR 204

WP_042233063_1 QGSQDNRAP--R--PSGD--GR---------------T-GA-P-RGDRPQ--G----QGAPR------SG----------GQG--GG-QRPQGG---QGGYNRGPQSGGQG-----RPQ----GGQ-RP-QG------------------QGGAPRSGG--QGG-----G--PRPQGG-------QG--G-GFNRG---P 220

WP_040948716_1 APSAQGAGQ--G--GQGG--QSGQ-G-GNRPQGERSYQ-GG-Q-GGNRPQ--GERSYQGGQG------GNRPQGERS-YQGGQ--GG-NRPQGE-RSYQGGQGGNRPQGGSYQ---GGQ----GGN-RP-QG-E-----RSYQGGQGGNRPQGGSYQGG--QGGNRSQGGSYQGGQGGNR----PQG--G-SYQGG---Q 288

WP_087432880_1 DGNRSGSRD--G--RPSS--PAAS---S----------------DRNANR-------GGSRD------GQ---------------RE-QQRQGD-R--------------------QTS-M--NT--RQ---------------------GNNQGKPNG--QGD--Q--------------RR-PQQ--G-GQSNN---M 203

WP_036579329_1 NDQVNRINP--N--QTEK--QETM-N-S---------T-TT-T-TNTQPQ--N----NANRQ-----------------QGGS--NG--RPQGQ--------SGQAQQGGGYN---RPQ----GGG-RP-QG-------------QGGQGQQGGGYNRP--QGQ----GGQNRGAQGGG------QG--G-QNRGP---Q 228

WP_068650597_1 NNNPNNNNGTGT--VTSR--PQSS--------------------TGTPRP-------QNS---------------------NN--GT-PRPQST-G--------------------GTT--------RP------A---S----------SQGGQRPSG--QQG-------------TSS-VN-RSS--SPSQGGG---Q 195

WP_046230755_1 TQNNQQQRP--S--QSSP-------S-S----------------NRSAAP-------SGNRP--A-Q-GS---GQ------SQ--GS-SRPSQG-G--------------------QTQ----NS--RP----------S----------QGGQSQNSR-------------------------PSQ--G-SGQGN---R 203

WP_099520650_1 TQNNQQQRP--S--QSSP-------S-S----------------NRSAAP-------SGSRP--A-Q-GS---GQ------SQ--SS-SRPSQG-G--------------------QTQ----NS--RP----------S----------QGGQSQNSR-------------------------PNQ--G-SGQGN---R 203

WP_036622334_1 NNKPNQNQN--A--KGGT--TAVQ--------------------ERNQRP-------NGGHQ-----------------------QG-PRASAQ-N--------------------AGN--------AG------G---R----------NPQGNRGQG--QTG-------------GDS-QR-RDG--G-GNRQG---N 202

WP_082083971_1 NSQANGAST--G--TQQR--TSSH-G-G-------------------NRQ--G--Q-QVGRS------GQ-----------GQ--GG-SRPQGS-----GTGRPQGQGAGRP----QGQ----GG--RP-QG-Q----------------SGGGSRPQG--QGQ----GG-RPQGTAGSR----PAQ--G-GGSRP---Q 225

WP_079940754_1 QGNGQGQSQ-----RKGS-------G-H---------N-------------------QG---------------------------N-ARPT------------------------ERN----NN--RS-QS------------------PGQNQQRRQ--NQG-----------------NK-PVS--D-NHNKP--SR 176

WP_006036631_1 TQQAPRQQQ--S--GQGG--GGYR-G-G---------Q-GG-Q-GGSRGP--G----QGGQG------GG---GYRG--QGGQ--GG-SRPPGQ----------GGQGGGGYR-GQGGQ----GGS-RP-PG---------------QGGQGGGGYRGG--QGG--GQGGSRPPGQGGQG-----GG--G-GYRGP---S 262

WP_089523112_1 SQGSNSTEQ--R--PSQG--SNST-E-Q---------R-PS-Q-GSAQGR--GPAP-SGGQP------RS----------GGQ--GGQSRPQGQ-GSGYGGNRPAGQGGGGSR--PAGQ----GGQ-SR-PA-----------------GQGGGSRPAG--QGG--GYGGNRPAGQGGGGSRPAGQG--G-GYGGS---R 266

WP_016312402_1 NNKPKTNQT--N--AGGS--TAVQ--------------------ERSSRP-------NGGNH-----------------------QN-SRPQGQ-G--------------------GGN--------TS------GAN-R----------NPQGNRGAG--QGG------------------Q-RND--S-GQRPA---Q 195

WP_037282270_1 SQSQGGNRP--Q--GNRS--SSSV-G-G--GQSRPQ-S-GG-A-SRPQSS--G----QGARP------SS---------GGGQ--GG-NRTGGG-------------QGGQ-----GGQ----GA--RP-GG------------------TSTGSQNRT--GGG--------------------ASS--S-QSRGP---S 191

WP_068694645_1 NQSEKQVSM--N--NNKT--TNQT-K-S---------A-GV-A-TQTRSN--Q--S-NGSSQ------NN---------------SG-SRPSSQ--------------GG------DRQ----GSG-RP-QG--------------------QNNRPNP--SGG--PKRG--------------PQN--S-GSRPS---Q 197

WP_055108420_1 NNRTDTNTS--S-HAGGA--QATQ--------------------ERNKRP-------GGGPQ-----------------------GN-R--------------------------------------------------N----------SSQGGRGSQ--PSG-------------GQR-AN-TNA--G-GARQG---Q 190

WP_068619253_1 NNNKSQNQS--N---RSA--QATQ--------------------VRNNPS-------SGGRE-----------------------QN-QGRPQQ-G--------------------NGG--------RN----------Q----------TSAGGQSRP--QGG-------------G----Q-HTA--S-TPRPQ---Q 191

WP_015844984_1 TASSQQANP--T--ASQT--NSTA-G-G---------S-SS-S-NNNRSQ--H----SGNRQ------GS-YQGNRPQGQGGQ--GG-NRPQGG----GGYNRPQGQ---------GGQ----GGN-RP-QG--------------------GGGYNRP--QGQ------------GGNR----PQG--G-GFNRP---Q 240

WP_091181685_1 SSSPSNAGA--S--SSSA--SSNN-S-S---------S-SS-N-NRPQNS--GN---SGNRQ------GS-YQGNRP-QGGGQ--GG-NRPQGG----GGYNRPQGQGGQG-----GGQ----GGS-RP-QG--------------------GGGYNRP--QGQ----GG--QGGQGGNR----PQG--G-GFNRP---Q 253

WP_044877552_1 NNNPSNNKS--A--VSST--PQTS--------------------QGASRP-------QSSTS----S-SR--------PQSNA--GT-SRPQSS-T--------------------GTQ--------RP------S---S----------TQGGQRTTG--QQG-------------GQS-AN-RGS--S-QQGGG---Q 205

WP_094155440_1 NRTNNNQNS--S--RSSA--PKAQ-G-S---------------------------Q-QGGQN------RS-----------QQ--SA-NKPSGQ----------------------QGS----QN--RS-NG-Q-----Q----------QGGQQRS--------------------------------A-SNTGT---R 181

WP_014281121_1 NRTNNNQNS--N--RSSA--PKAQ-G-S---------------------------Q-QGGQN------RS-----------QQ--ST-SRPSGQ----------------------QGS----QN--RS-GG-Q-----Q----------QGGQQRPST--NTS-----------------SR-PQG--S-QSTNS---R 176

WP_023988217_1 NRTNNNQNS--N--RSSA--PKAQ-S-S---------------------------Q-QGGQN------RS-----------QQ--ST-SRPTGQ----------------------QST----QN--RS-NG-Q-----Q----------QGGQQRPSG--NTG-----------------SR-PQG--N-QSTNS---R 176

WP_028542714_1 NRTNNNQNS--N--RSSA--PKAQ-S-S---------------------------Q-QGGQN------RS-----------QQ--ST-SRPTGQ----------------------QST----QN--RS-NG-Q-----Q----------QGGQQRPGG--NTG-----------------SR-PQG--N-QSTNS---R 176

WP_071640528_1 NRTNNNQNS--N--RSSA--PKAQ-S-S---------------------------Q-QGGQN------RS-----------QQ--ST-SRPTGQ----------------------QST----QN--RS-NG-Q-----Q----------QGGQQRPSG--NTG-----------------SR-PQG--N-QSTNS---R 176

WP_013370722_1 NRTNNNQNS--N--RSSA--PKAQ-S-S---------------------------Q-QGGQN------RS-----------QQ--GS-SRPGGQ----------------------QGT----QN--RS-NG-Q-----Q----------QGGQQRT-----------------------------G--S-NNTGS---R 167

WP_016820981_1 NRTNNNQNS--N--RSSA--PKAQ-S-S---------------------------Q-QGGQN------RS-----------QQ--GS-SRPSGQ----------------------QGT----QN--RS-NG-Q-----Q----------QGGQQRT-----------------------------G--S-NNTGS---R 167

WP_068655661_1 NNNSNNTNV--S--VTSR--PESN--------------------TSTPRT-------QSTTS----A-PR--------AQGNNNAGS-QRPQGA-G--------------------GPS--------RP------A---S----------SQGGQRPTS--QQG-------------TQT-AN-RTT--T-SSQGD---N 202

WP_060534524_1 NRSNSSSSQ--Q--GNRS--AQG--G-S---------------------------S-SGQQG------NR-----------SS--QG-GSSTGQ----------------------QGN----RS--NS-QG-------------------GNRQPS--------------------------------S-AASRP---Q 173

WP_074094581_1 NNRPNSNNN--N--GTQR--PSGQ-D-S---------R-NR-T-NSSQGS--S----QGGQS--T-----------------------NRPRPA-------------QGGQ-----SST----AS--RP-QG------------------TGQRPNNSG--GGQ-VRTSG--------------PNS--G-GNTGT---A 202

WP_062326090_1 NNRPNSNNN--N--GSQR--PSGQ-D-S---------R-NR-T-NSSQGS--S----QGGQS--S-----------------------NRPRPA-------------QGGQ-----SST----AS--RP-QS------------------TGQRPTNSG--GGQ-TRSAG--------------PNS--G-GNTGT---G 202

WP_015736303_1 NSRTNSNTQ--Q--ASGS--QRPQ-G-G---------Q-DT-R-RNQSGS--A----QGSQQ------NN-----------RS--QG-SRPGGS----------------------TQT----GS--RP-QG-------------------------GG--QGG-----------------GR-PQG--N-NQGGS---R 216

WP_036642094_1 NSRTNQNNQ--S--ASGS--QRPQ-G-G---------Q-DT-R-RSQSGS--T----QGVQQ------NN-----------RS--QA-SRPAGS----------------------TQT----GS--RP-QG-------------------------GG--QGG-----------------AR-PQG--S-NQTGS---R 216

WP_108465278_1 DQTKEGNSS--N--NKIQ--MEKQ-V-S---------M-NK-N-TTTNNQ--R----SGAAS------SQ---SNHN-----------SQQGGQ----------------------RQN----SGA-RS-SQ------------------QGGQNRSGG--QGQ---------------------SQ--G-GASRP---N 188

WP_036651540_1 RRPSSQQSS--S--STSR--PQGQ-S-S--G------Q-GN-R-PQGQSS--G----QGNRT--Q---GS---------TYQG--NN-PRPQGQ------------QSGGQ------------GN--RT-QG-S-----S---S------QGGYTSRPS--SSP--------------------SQG--G-GQSRP---Q 220

WP_099479276_1 NSRNHQNNQ--NQNTSGS--QRPQ-GQG---------Q-DS-R-RSQSGP--A----QGSQN------QN-----------RG--NQ-APRQGG----------------------TAS----GS--RP-QG-------------------------GG--QGG-----------------AR-PQN--N-GA------- 197

WP_076168052_1 NNKLNNTTQ------TGS--QRPQ-G-G---------Q-DS-R-RSHSGS--Q----QSRPQGQSNGSGN-----------RP--QG-AGAGSN----------------------NRNQGSQGG--RP-QG-------------------------SG--QGG-----------------NR-PQG--S-GQGGN---R 193

WP_038696662_1 QSGSVQQRP--Q--GQGG--AQRQ-S-G------------------GRPQ--G----QGGAP------RQ---------------GE-GRPQGQ--------------GGAPR---QGE----G---RP-QG------------------QGGAPRPSS--GNR--------PQGQGG------APR--Q-GENRP---Q 205

WP_042207090_1 NQTGSGPRP--Q--GQGG--ASRQ-G-G-------------------RPQ--G----QGGAP------RQ---GESR-PQGQG--GA-SRPQGQ----GGASRPQGQGGASRP---QGQ----GGAPRP-QG------------------QGGAPRPQG--QGG--------------------APR--Q-GDSRP---Q 217

WP_082210233_1:7-883 NQTGSGPRP--Q--GQGG--ASRQ-G-G-------------------RPQ--G----QGGAP------RQ---------------GE-ARPQGQ----GGTPRPQGQGGG------QRQ----GDS-RP-QG------------------QGGAPRPQG--QGG----------------------------APRP---Q 199

WP_025335323_1 QSGSVQQRP--Q--GQGG--AQRQ-G-G-------------------RPQ--G--Q-GGARP------GE------------------SRPQGQ--------------GGAPR-----Q----GDS-RP-QG------------------QGGASRPQG--QGG--------------------PSR--P-GDSRP---Q 195

WP_036656399_1 AGSTQNARP--Q--QSGA--PRPS-N-T---------G--G---SRPQGSTSS--T-GGSRP--Q---GS-----------NT--SS-NRPQ-S----------------------GTG----GS--RP-QGSTGTRPQS----------STGGSRPQGGTSST----GG-----------SR-PQGATS-SAGGS---R 225

WP_087920295_1 TSSTSSPRP--Q--GSSTGGNRPQ-G-S---------STGG---NRPQGS--S--T-GGTRP--Q---GS-----------ST--GG-TRPQGS----------------------STG----GT--RP-QG-------S----------STTGARPQG--SST----GG-----------TR-PQG--S-STAGA---R 231

WP_042138118_1 PQG----------------SSTTG-SRPQG-QG--G------APR-----T-G----D-RPQG-Q--G--TAPR-TG-DRPQG-QGN-AG--GDFSR-----------G---G--DR-GPKK----------------------NA-T-GG----------------RP-NN-------NS---------G-QK--R-FD 288

WP_042214813_1 PQG----------------SS-TG-SRPQG-QGS-G------APR-----T-G----D-RPQG-Q--G--SAPR-TG-DRPQG-QGN-AG--GDFSR-----------G---G--DR-GPKK----------------------NT-T-GG----------------RP-NN-------NS---------G-QK--R-FE 288

WP_042237107_1 PQG----------------SSTTG-SRPQG-QG--G------APR-----T-G----D-RPQG-Q--G--TAPR-TG-DRPQG-QGN-AG--GDFSR-----------G---G--DR-GPKK----------------------NA-T-GG----------------RP-NN-------NS---------G-QK--R-FD 287

WP_039874189_1 PQG----------------SS-TGGNRPQG-GST-G----------------G----N-RPQG-QG-S--SAPR-TD-SRPQG-QG---G-GGDFSR-----------G---G--DR-GPKK----------------------NT-T-GN----------------RP-NN-------NS---------G-QK--R-FE 276

WP_038593413_1 PQG----------------GS-TG-SRPQA-QG--G------APR-----T-G----ENRPQA-Q--G-----------------GG-TG--GDFSR-----------G---GGGDR-GAKR----------------------NT-T-GG----------------RP-NN-------NS---------G-QK--R-FE 277

WP_042177491_1 PQG----------------GS-TG-SRPQG-QGG-G------APR-----T-G----D-RPQ-----S--SAPR-TD-SRPQG-QG---G--GDFSR-----------G---G--DR-GPKK----------------------NT-T-GN----------------RP-NN-------NS---------G-QK--R-FE 275

WP_036688463_1 PQG----------------SN-----------------------------TSG----S-RPQG-Q--N--SAPR-TD-SRPQG-QSG-TG--GGFTR-----------G---D--DR-GPKK----------------------NT-T-GG----------------RP-NN-------NQ------------R--R-FD 273

WP_042266949_1 PQG----------------SS-TG-SRPQG-QGG-G------APR-----T-G----D-RPQG-Q--G--GTRT-AD-SRPQG-QG---G--GDFSR-----------G---G--DR-GPKK----------------------NA-T-GG----------------RP-NN-------NS---------G-QK--R-FE 271

WP_042128858_1 PQG----------------SN-----------------------------T-G----S-RPQG-Q--S--SAPR-TD-SRPQG-QSN-TG--GGFTR-----------G---D--DR-GPKK----------------------NT-T-GG----------------RP-NT-------NN------------R--R-FD 268

WP_042189415_1 PQG----------------SN-----------------------------T-G----S-RPQG-Q--S--SAPR-TD-SRPQG-QSG-TG--GGFTR-----------G---D--DR-GPKK----------------------NT-T-GG----------------RP-NT-------NN------------R--R-FD 268

WP_046504296_1 PQG----------------QG--G-----------G------APR-----T-G----D-RTQG-Q--G--GART-AD-SRPQG-QG---G--GDFSR-----------G---G--DR-GPKK----------------------NT-T-GG----------------RP-NN-------NS---------G-QK--R-FE 262

WP_016362856_1 GGGGQGGGGYRGGQGGSGGGQGGG-GYRGG-QG--GSSGGQGGGGYRG----G----Q-GGGG-Q--G--GGYR----GGQGGSSGG-QG--GGGYR-----------G---G--QG-GGARTGAAPAPAAAARPD--------SR-R-GNFDQRNRTAATGGKPAAAVIDK-------DGEEKFKRSRPA-GK--R-FD 391

WP_068608410_1 GQG----------------SQGGG-QRTSS-TGQGG-----------Q----G----A-GRSY-S--G--QGSQ----GGQGQ-----GG--GNRFG-----------N---S--SG-SGAPRSSQPARPAFGSSP--A-----PA-S-GG-LNQKQPNRKS--------TK--P----TG------------K--R-FE 329

WP_062408002_1 GQG----------------GRGPG-QRPQG-QQ--G-------GG-QQ----G----Q-RPDA-R--R--NGLD-QR-GGSNR-TPG-SG--ESRSL-----------G---S--VL-QVDVDDNVAAKGAANKK---------TK-L-GQ------------------------------------------K--R-FD 276

WP_005550539_1 GQG----------------GAPRQ-GGQGG-QG--G----------QG----G----A-PRPG-Q--G--GGSR----PGQGS-QQS-SG--QARFA-----------Q---G--GQ-QRQGDQARAGQGGQQRPN--RNFG--ER-S-DD-FSGGRNQSR---------NN-------NN---------N-KK--R-FD 338

WP_015254924_1 PQG-------------------GG-GMRGG-AG--G----------------G-F----RGAA-L--A--PAAP----PQPDR-KKG-SG--KDKDK-----------D------RR---------------------------NK-N-GE-------------------NS---------------------R--K-FE 258

WP_042233063_1 ---------------------------QGG-SG--G--QRPQGGG-------------------Q--G--GQNR----GGSAG--AG-AR--PGQAP-----------R---R--TD-GRPAGGADAGRGGQRTGD--------NR-Q-G--NRDDRSKSS-----------------------------------R-FD 291

WP_040948716_1 GGNRPQGGSYQGGQGG-NRPQGER-SYQGG-QG--G--NRPQGGSYQG----G----Q-GGNRPQ--G--GSYQ----GGQGGSNRS-AS--PGASS-----------Q---S--TM-AQTPARAADRPQRKPEPARTADKDRKPR-V-GE-GRPNRNASVPGVDAMDVKAN-------EG-IGGKRTKQG-GR--R-FD 429

WP_087432880_1 NRG--------------------------------G----------------Q----Q-RTAP-Q-----QSQR----QQQPA---------RSFDS------------------NN-DSAS----------------------QK-P-GN-KNQSR----------KP-NS-------GS------------K--R-FD 254

WP_036579329_1 GGNRPAGQG---GQGGFRSGQAQG-GGQGG-QG--G--NRPVGAG-------------------Q--G--GFRS----GGPGG---------AGVSQ-----------G---G--AR-PQQNRFNDSKPGSGR-----------PG-E-G--GDSSRNR-----------NN-------NN-----RSKPG-GG--R-FD 324

WP_068650597_1 RSS----------------------------SP--T--TRPSSPA-------A------RPQT-P--N-------RT-TGAPT---------RDASRTEGAAQGDNRGG---A--NK---------------------------NR---SN-------------------NN-------GS------------R--R-FD 254

WP_046230755_1 PSS--------------------------------G----------------G-P--S-RPPA-S--Q--GANR----PSGTG-PSR-PA--GDASR-----------S------TN-GNSN----------------------NK-P-GA--------------R-KP-GA-------NQ---------G-QK--R-FE 260

WP_099520650_1 PSS--------------------------------G----------------G-P--S-RPPA-S--Q--GANR----PSGTG-PSR-PA--GDASR-----------S------TN-GNAN----------------------NK-P-GA--------------R-KP-GA-------NQ---------G-QK--R-FE 260

WP_036622334_1 GQG----------------------------NG--G-ANRGGQAP-------N------RNFV-S--A----------GSNNN---------NGGGK-----------G---Q--GQ---------------------------GR---KG-------------------NH-GG-K--NQ------------K--G-FD 252

WP_082083971_1 GQG-------------------GA-GQGQS-FG--G--NRP-----------------------A--G--G--R----SGQGG---------PARTF-----------S---D--NR-PASGGSGRPGGGDSN-------F---SR-N-SG-GGAGKRKP----------GE-------NK---------------R-FD 295

WP_079940754_1 VQA-------------------GG-A--------------------------------------------------------------NN--TSEKR-----------G------KN---------------------------KH-T-GG-------------------NQ---------------------K--R-FD 203

WP_006036631_1 GQGGSRPPG--------QGGQGGG-GYRGG-QG--G----------QG----G----S-RPPG-Q--G--GGRP----GGFGG--GG-SQ--GSAPR-----------T---G--AP-SGTSRATDSRSGQGARPS--------DN-S-GN-NWASRKTKPGGN------NN-------NG-----------SR--R-FD 362

WP_089523112_1 -----------------PVGQGGG-SRPPG-QG--G----------------G----Y-GGAR---------PQ----GQSSAPSAG-AA--PRGES-----------R---S--TA-PAPRTFGENKPAGGARTD----FNRSSG-P-G--GGAGRSGGPGGAGA----NR-------RG-----GGKPG-GQNGR-FD 365

WP_016312402_1 GQN----------------------------GG--G----------------N------RSFA-P--S----------NNLGG---------GNGSK-----------G---Q--GQ---------------------------RK---GN-------------------NN-GG-K--NQ------------R--G-YD 237

WP_037282270_1 SSN----------------SSYGG-NRSGG-QG--G----------QG----G----N-RTGG-Q--G--G----Q--GGNRT-----GG--PGGRP-----------G---G--QR-RSD-----------------------DS-R-G----------------------------------------------G-FR 245

WP_068694645_1 GGG------------------QGG-NRPAQ-GG--G----------------------------Q--G--GANR----PQSSA-NRS-GG--DSGRP-----------G---S--TQ-DRA-----------------------RR-D-G--GGNNR-------------GG-------QK---------------R-FE 255

WP_055108420_1 GSG----------------------------NT--A------AAP-------N------RNNG-P--AARQPQSSND-SNAAG---------RNQDR-----------G---Q--GQ---------------------------RK---GN-------------------NN--R-P--GQ------------R--R-FD 243

WP_068619253_1 NQN----------------------------RS--S------QPQ-------A------RTAS-S--G-------ND-NYSSN---------RGQGQ-----------G---Q--GQ---------------------------KR---NN-------------------NN-GR-P--GQ------------K--R-FD 238

WP_015844984_1 GQG----------------GAPGG-NRPQG-QG--G---AP------G----G----N-RPQG-Q--G--G-AP----GGNRPQGQG-GG--QNRSF-----------D---S--SR-PAPTSRGTAAGESN------------NR-K-GN-NAVNKNRT----------NN-------TG---------G-QK--R-FD 327

WP_091181685_1 GQG-------------------------AG-QG--G--NRP-----------------------Q--G--GGAP----GGNRPPQGG-GG--PARSF-----------D---S--SR-PAPTSRGTAAAET-------------NR-K-GN-NTVNKNKS----------TN-------NG---------S-QK--R-FD 326

WP_044877552_1 RSS----------------------------ST--T----------------A------RPQT-Q--N-------RA-GGAQT---------TDSSKP----------G---V--NK---------------------------NRPGNSG-------------------NN-------GA------------R--R-YD 250

WP_094155440_1 SQG-------------------------SS-QG--G-----------T----A----V-RTSS-S--T--GSGN----ANRNGGGNR-TG--SNFSN-----------G---N--NR-SGQGRFDD------------------NR-Q-GG-------------------GR-------GG---------------N--- 240

WP_014281121_1 PQG-------------------------SN-QG--G-----------T----A----V-RTAS-S--T--GSSN----ANRSGGGNR-TG--SNSNN-----------G---G--NR-SGQGRFDD------------------NR-Q-GG-------------------GR-------GG---------------N--- 235

WP_023988217_1 PQG-------------------------SS-QG--G-----------T----A----V-RTSS-S--T--GSNN----ANRSG-GNR-TG--SNSSN-----------G---G--NR-SGQGRFDD------------------NR-Q-GG-------------------GR-------GG---------------N--- 234

WP_028542714_1 PQG-------------------------SS-QG--G-----------T----A----V-RTSS-S--T--GSNN----ANRSG-GNR-TG--SNSSN-----------G---G--NR-SGQGRFDD------------------NR-Q-GG-------------------GR-------GG---------------N--- 234

WP_071640528_1 PQG-------------------------SS-QG--G-----------T----A----V-RTSS-S--T--GSNN----ANRSG-GNR-TG--SNSNN-----------G---G--NR-SGQGRFDD------------------NR-Q-GG-------------------GR-------GG---------------N--- 234

WP_013370722_1 PQG-------------------------SS-QG--G-----------A----A----V-RTSS-S--A--GSNN----SNRSG-GSR-TG--GNSNN-----------S---G--NR-SGQGRFDD------------------NR-Q-GG-------------------GR-------GG---------------N--- 225

WP_016820981_1 PQG-------------------------SS-QG--G-----------A----A----V-RTSS-S--A--GSNN----SNRSG-GNR-TG--GNSNN-----------S---G--NR-FGQGRFDD------------------NR-Q-GG-------------------GR-------GG---------------N--- 225

WP_068655661_1 R-----------------------------------------------------------------------------------------------------------G---A--NK---------------------------NR---SN-------------------NN-------GS------------K--R-FD 219

WP_060534524_1 SSS-------------------------QN-RQ--P-----------S----G----Q-VRTA-Q--S--GDTS----GNRNSGGTG-SN--SGGNR-----------G---G--N--SGGNRNNN------------------NR-G-GS-------------------GG-------GG---------------GRFD 234

WP_074094581_1 GNR----------------------------SG--G----------------------------Q--G--QSQG-Q--GQRRS-GPGGTT--GSNNN-----------S---G--NR-S-------------------------NS-G-GG-------------------GR------------------------R-YD 246

WP_062326090_1 GNR----------------------------TG--G----------------------------Q--G--QSSG-Q--GQRRG-GPG-NS--GNNNN-----------S---G--NR-S-------------------------NS-G-GG-------------------GR------------------------R-YD 245

WP_015736303_1 PQG-------------------------NN-QG--G--TRP-----QG----G----N-NP------G--GARP----QSKPG-GTG-GG--FSGSN-----------N---S--NG-QNRG----------------------SR-N-NN-------------------NNRSD----SK---------------R-FD 276

WP_036642094_1 PQG-------------------------SNNQG--G--AR---------------------------------P----QSKPG-GTG-GGFSSSNNN-----------S---N--NS-QNRG----------------------SR-T-NN-------------------NN--NNRQDSK---------------R-FD 270

WP_108465278_1 PNR-----------------PGQS-RPAQG-QG--G----------QQ----N----S-RPQS-G--G--GGQS-R--GGQGQ-TRS-NS--DGARK-----------N---N--NN-N-------------------------GR-P-GQ------------------------------------------K--R-FD 247

WP_036651540_1 GSQ----------------GAGAG-QRSSS-----G-----------T----S----S-RPPQ-N--R--SGFQ----SGQPS-----GG--GDSF-------------------------------------SKP--G-----EN-R-GG-NNNKS---RG--------SN--N----GS------------K--R-FD 283

WP_099479276_1 -------------------------------QG--A--SR---------------------------------P----QGRTG-RTD-SG-NQNNNN-----------S---N--SQ-NNRG----------------------GR-N-NN-------------------NN--N----SK---------------R-FD 240

WP_076168052_1 PQG-------------------------SQ-QG--G--GQR-----SG----A----G-RPQQ-QSTG--NRGP----QGNAG-GDN-NF--SRSND-----------N---R--GG-GNRG----------------------QN-R-GN-------------------NN--G----SK---------------R-FD 256

WP_038696662_1 GQG-------------------GG-QRQGG-FG--G-----------G----G----N-RPQG-Q--G--SAPR----SGESR--SQ-QG--GDFNR-----------G---G--DR-NK------------------------NR-S-GG-GGGQK---------------------------------------R-FE 264

WP_042207090_1 GQG------------------GGG-QRQGG-FG--G----------------G----N-RPQG-Q--G--GASR----QGDSR-PQG-QG--GDFSR-----------G---G--DR-NSK-----------------------NR-P-G--------------------SN-------QK---------------R-FD 276

WP_082210233_1:7-883 GQG------------------GGG-QRQGG-FG--G----------------G----N-RPQS-Q--G--GGQR----QGDSR-PQG-QG--GDFSR-----------G---G--DR-NSK-----------------------NR-P-GS-GGTNK---------------------------------------R-FE 260

WP_025335323_1 GQG------------------GGG-QRQGG-FG--G----------------G----N-RPQS-Q--G--GAPRPQSQGGTPR-PQG-QG--SGDSF-----------R---G--DR-NSK-----------------------NR-Q-G--------------------GN-------NK---------------R-FE 258

WP_036656399_1 PQG----------------SA-PR-TDNRP-QG--G------APR-----T-GAPSTP-RPQGAQ--G--GAPR-TG-DRPQG-AGQ-GG--GDFSR-----------GTGTG--DR-GPKK----------------------TA-T-GG----------------RP-GP-------NQ---------GGQR--R-FD 307

WP_087920295_1 PQG----------------SSTGG-TRPQG-SGT-A------GARPQGSSP-G----S-RPQG-Q--G--GAPR-T------G-QGG-TG--GDFSR-----------G---G--DKGGPKK----------------------NT-T-GG----------------GG-RP-------GT---------G-QR--R-FD 307

WP_042138118_1 ----DGKG-GNF----R------GRG-GK-NNRG-R-NQPM---VHREKIDNTPKKIIVRGSMTVGETAKLLHKDASEVIKKLISMGVMATINQELDIDTILLLAGEFGV-EVEVKIPVDEDSFETVEENDSDD-DLQSRPPVVTIMGHVDHGKTTLLDAIRSTNVTGGEAGGITQHIGAYQVEINHKKITFLDTPGHEA 464

WP_042214813_1 ----DGKG-GNF----R------GRG-GK-NNRG-R-NQPM---VHREKIDNTPKKIIVRGSMTVGETAKLLHKDASEVIKKLISMGVMATINQELDIDTILLLAGEFGV-EVEVKIPVDEDSFETVEENDSED-DLQTRPPVVTIMGHVDHGKTTLLDAIRSTNVTGGEAGGITQHIGAYQVEINHKKITFLDTPGHEA 464

WP_042237107_1 ----DGKG-GNF----R------GRG-GK-NNRG-R-NQPM---VHREKIDNTPKKIIVRGSMTVGETAKLLHKDASEVIKKLISMGVMATINQELDIDTILLLAGEFGV-EVEVKIPVDEDSFETVEENDSDD-DLQSRPPVVTIMGHVDHGKTTLLDAIRSTNVTGGEAGGITQHIGAYQVEINHKKITFLDTPGHEA 463

WP_039874189_1 ----DGRG-GNY----R------GRGNGK-NNRGGR-NQPM---VHREKIDNTPKKIIVRGSMTVGETAKLLHKDASEVIKKLILMGVMATINQELDIDTILLLAGEFGV-EVEVKIPVDEDSFETVEENDSDE-ELMTRPPVVTIMGHVDHGKTTLLDAIRSTNVTGGEAGGITQHIGAYQVEINHKKITFLDTPGHEA 454

WP_038593413_1 ----DGKG-GNY----R------GRG-GK-NNRG-R-NQSM---ERREKIDNTPKKIIVRGSMTVGETAKLLHKDASEVIKKLISMGVMATINQELDIDTILLLAGEFGV-EVEVKIPVDEDSFETVEENDSEE-DLQARPPVVTIMGHVDHGKTTLLDAIRSTNVTGGEAGGITQHIGAYQVEINHKKITFLDTPGHEA 453

WP_042177491_1 ----DGRG-GNY----R------GRGNGK-NGRGGR-NQPM---VQREKIDNTPKKIIVRGSMTVGETAKLLHKDASEVIKKLILMGVMATINQELDIDTILLLSGEFGV-EVEVKIPVDEDSFETVEENDSDE-ELMTRPPVVTIMGHVDHGKTTLLDAIRSTNVTGGEAGGITQHIGAYQAEINHKKITFLDTPGHEA 453

WP_036688463_1 ----DGKG-GNY----R------GRG-GK-NGRG-K-NQPM---VHREKIDNTPKKIIVRGSMTVGETAKLLHKDASEVIKKLISMGVMATINQELDIDTILLLAAEFGV-EVEVKIPVDEDSFETVEENDNEE-DLQTRPPVVTIMGHVDHGKTTLLDAIRSTNVTLGEAGGITQHIGAYQVEINQKKITFLDTPGHEA 449

WP_042266949_1 ----DGKG-GNF----R------GRG-GK-NNRG-RSNQPV-----REKIDNTPKKIIVRGSMTVGETAKLLHKDASEVIKKLISMGVMATINQELDIDTILLLSGEFGV-EVEVKIPVDEDSFETVEENDSEE-DLQTRPPVVTIMGHVDHGKTTLLDAIRSTNVTGGEAGGITQHIGAYQVEINHKKITFLDTPGHEA 446

WP_042128858_1 ----DGKG-GNY----R------GRG-GK-NGRG-K-NQPM---VHREKIDNTPKKIIVRGSMTVGETAKLLHKDASEVIKKLISMGVMATINQELDIDTILLLAAEFGV-EVEVKIPVDEDSFETVEENDTED-ELQSRPPVVTIMGHVDHGKTTLLDAIRSTSVSLGEAGGITQHIGAYQVEINQKKITFLDTPGHEA 444

WP_042189415_1 ----DGKG-GNY----R------GRG-GK-NGRG-K-NQPM---VHREKIDNTPKKIIVRGSMTVGETAKLLHKDASEVIKKLISMGVMATINQELDIDTILLLAAEFGV-EVEVKIPVDEDSFETVEENDAEE-ELQSRPPVVTIMGHVDHGKTTLLDAIRSTSVSLGEAGGITQHIGAYQVEINQKKITFLDTPGHEA 444

WP_046504296_1 ----DGKG-GNF----R------GRG-GK-NNRG-RNNQPV---ERREKIDNTPKKIIVRGSMTVGETAKLLHKDASEVIKKLISMGVMATINQELDIDTILLLSSEFGV-EVEVKIPVDEDSFETVEENDSEE-DLQTRPPVVTIMGHVDHGKTTLLDAIRSTNVTGGEAGGITQHIGAYQVEINHKKITFLDTPGHEA 439

WP_016362856_1 ----DNKP-GTG----RGAGR--NNR-GK-GGRN---QEPPKP-----KIDNTPKKIIVRGTMTVGELAKALHKDASEVIKKLLFLGTMATINQELDLDAIQLLADDYKV-EVELKIPVEEDKFEQFEENDEEA-DLLERPPVVTIMGHVDHGKTTLLDAIRSTNVTEGEAGGITQHIGAYQVEIHNKKITFLDTPGHEA 568

WP_068608410_1 ----DGKP-GGFGGGNR--NNGFNRN-NK-GKGR---HQSNNQ-EKREKIDNTPKKIIVRGNMTVGEMAKSLHKDASEVIKKLMSLGTMATINQELDLDAIQLLAGDFGV-EVEIKIAVEEDRFENFEESDEAE-DLMDRPPVVTIMGHVDHGKTTLLDAIRETNVTGGEAGGITQHIGAYQVEVHGKKITFLDTPGHEA 514

WP_062408002_1 ----DFKS-GGK-GL-Q--NR--GNQ-KN-QGKG---GKNHSE-PPKPKIDNTPKKIIVRGTMTVGELAKALHKDASEVIKKLLFLGVMATINQELDLDAIQLLASDYGV-EVELKIPVEEDKFEQFEEIDDEA-DLLERPPVVTIMGHVDHGKTTLLDAIRKTNVTEGEAGGITQHIGAYQVEAHGKKITFLDTPGHEA 457

WP_005550539_1 ----DNRN-GGG-M--RGRGG--KNR-GR-NNQQ---Q------VQREKIDNTPKKIIVRGAMTVGELAKLLHKDASEVIKKFLLMGVMATINQEVDLDTIQLIADEFKV-EVDLKLPVSEDPFEETEEKEEDAADLVERPAVVTIMGHVDHGKTTLLDSIRKTNVTGGEAGGITQHIGAYQVEFNSKKITFLDTPGHEA 516

WP_015254924_1 ----E-PR-GGF----K--GK--LNA-GK-GGKK---GQE-----RREKVDNTPKKIIVRGEMTVGELAKLLHKDVSDVIKKLMFLGVLATINQVLDLDAIQLVAGEYGV-EVEIKLPVDEDAFETVEEQDDPA-DLKPRPPVVTIMGHVDHGKTTLLDAIRHTNVTSGEAGGITQHIGAYQVEVNGKKITFLDTPGHEA 432

WP_042233063_1 ----DGRG-GRGGAGNRG-GN--NRG-GR-NNGR---GRGQEPMVKKEKIDNTPKKIIVRGAMTVGELAKLLHKDVSEVIKKLLFLGVMATINQELDLDTIQLVAGEYNV-EVDLKIRVEDDRFELEEEVDDPA-DLKERPPVVTIMGHVDHGKTTLLDAIRHTSVTEGEAGGITQHIGAYQVEANGKKITFLDTPGHEA 476

WP_040948716_1 ----DGKP-GMG----KG-GR--FGK-GK-QQMQ---MK-------KEKVDNTPKKIIVRGEMTVGDLAKLLHKDLSEVIKKLLLMGTMATINHELDLETIQLVAQEFGV-EVELKIAVDEDNFELIEETDEDG-DLQERPPVVTIMGHVDHGKTTLLDAIRETHVTEGEAGGITQHIGAYQVEINNKKITFLDTPGHEA 603

WP_087432880_1 -D--NRTG-GNM----RG-GRG-GRG-GK--NRG-R-GNNQQQ-VQREKIDNTPKKIIVRGEMTVGELAKLLHKDASEVIKKLLFLGVMATINQEVDLETIQLIADEFKV-EVDLKLPVSEDPFEDTEEKDDEA-DLQERPAVVTIMGHVDHGKTTLLDAIRKTNVTSAEAGGITQHIGAYQVEFNNKKVTFLDTPGHEA 436

WP_036579329_1 ----DNRNYKGG----RG-GK--NNR-GG-RGNQ---NQ-----ERREKIDNTPKKVIVRGSMTVGELAKLLHKDASEVIKKLLLMGVMATINQELDLDTIQLVIGDYGV-EAEIKLPVDEDSFETVEENDEDA-DLETRPPVVTIMGHVDHGKTTLLDAIRETNVTGGEAGGITQHIGAYQAEINQKKITFLDTPGHEA 501

WP_068650597_1 ----DNKG-GNY-------RN--NRG-GK-NGRG-K-ANYQA----REKIDNTPKKIIVRGVTTVGESAKLLHKDASEVIKKLITMGVMATINQELDVETVLLLAAEFGV-EVEVKIAVEDDRFETMEEVDEAA-DLKPRAPVVTIMGHVDHGKTTLLDAIRSANVSGGEAGGITQHIGAYQVEINNKKITFLDTPGHEA 430

WP_046230755_1 ----DGRP-GGF-G--RN-NNN-NRG-GR-GRNQ-R-NQPQ---VFREKIDNTPKKIIVRGTLTVGELAKLLHKDASEVIKKLIVLGVMATINQEIDMDAILLLAGEYGV-EVEVKIPVEEDTFETVEETDEEA-DLLPRPPVVTIMGHVDHGKTTLLDAIRKTNVTGGEAGGITQHIGAYQIEANHKKITFLDTPGHEA 441

WP_099520650_1 ----DGRP-GGF-G--RN-NNN-NRG-GR-GRNQ-R-NQPQ---VFREKIDNTPKKIIVRGTLTVGELAKLLHKDASEVIKKLIVLGVMATINQEIDMDAILLLAGEYGV-EVEVKIPVEEDTFETVEETDEEA-DLLPRPPVVTIMGHVDHGKTTLLDAIRKTNVTGGEAGGITQHIGAYQIEANHKKITFLDTPGHEA 441

WP_036622334_1 ----DNRS-GNF--K----NN--NRG-GK-GGRGGR-GGYQQ--PPREKIDNTPKKIIVRGTMTVGETAKLLHKDASEVIKKLMLLGVMATINQELDIETIQLLAGDFGV-EVEVKIPVEEDRFETIEEVDDEA-DLKERPPVVTIMGHVDHGKTTLLDAIRSTNVTGGEAGGITQHIGAYQVEINGKKITFLDTPGHEA 432

WP_082083971_1 ----DQRG-GNG-F-----RN--NRG-GK-GNNR---GRGAVQ-PPREKIDNTPKKIIVRGNMTVGDLAKLLHKDASEVIKKLIFLGVMATINQELDLDAIQLVADEYKV-EVEVKIPVEEDTFENEEEKDDEA-DLESRPPVVTIMGHVDHGKTTLLDSIRKTNVTGGEAGGITQHIGAYQVEINHKKITFLDTPGHEA 474

WP_079940754_1 ----DNNR-GQN----R--GN--NYK-GN-RGRG---RNQEPV-VKKEKIDNTPKKIIVRGTMTVGEVAKALHKDASEVIKKLLFLGVMATINQELDLDAIELVATEFGV-AVEVKIPVEEDKFELVEEQDDEK-DLRDRPPVVTIMGHVDHGKTTLLDAIRHTSVTEGEAGGITQHIGAYQVEVNGKKITFLDTPGHEA 382

WP_006036631_1 ----DGKG-GNF----RG-GN--ARG-GK-GGRN---GRGSVPQVHREKIDNTPKKIIVRGTMTVGELAKLLHKDASEVIKKLIFMGVMATINQEVDLDTIQLIATELGVEEVEIKIRVEEDQFETVEENDDES-ELEARPAVVTIMGHVDHGKTTLLDAIRQSNVTGGEAGGITQHIGAYQVEVNHKKITFLDTPGHEA 544

WP_089523112_1 ----D-RG-GSF----RNRGK--NGR-GK-SQQP---QR--------EKIDNTPKKIIVRGNMIVAELAKLLHKDASEVIKKLISLGVMATINQELDIETIQLVASEFKVEEVEIKIPVEEDDFETVEEKDNEE-DLQSRAPVVTIMGHVDHGKTTLLDAIRSTNVTSGEAGGITQHIGAYQVEINNKKITFLDTPGHEA 539

WP_016312402_1 ----DNRN-GAN--F----KN--NRG-GK-GGRG-K-GAPQQ--PPREKIDNTPKKIIVRGTMTVGETAKLLHKDASEVIKKLMLLGVMATINQELDLDTIQLLASDFGV-EVEVKIPVEEDRFETIEEVDDEA-DLKERPPVVTIMGHVDHGKTTLLDAIRSTNVTGGEAGGITQHIGAYQVEINGKKITFLDTPGHEA 416

WP_037282270_1 ----GGQG-GRG-------GK--GGR-GG-NNRN---NQ-----PPREKIDNTPKKIIVRGTMTVGETAKLLHKDASEVIKKLIMLGVMATINQELDLETIQLLAGDFGV-EVEIKIPVEDDRFETLEETDEEA-DLRERPPVVTIMGHVDHGKTTLLDAIRSTNVTGGEAGGITQHIGAYQVEINNKKITFLDTPGHEA 419

WP_068694645_1 ----DGNR-GGF----RGGNN--NRG-GR-GGRG---GKGGQQQERREKIDNTPKKIIVRGEMTVGETAKLLHKDASEVIKKLISLGVMATINQELDMDTILLLAGDFGV-EVEVKIPVEDDRFETLEENDDPA-DLEVRPPVVTIMGHVDHGKTTLLDTIRSTSVTAGEAGGITQHIGAYQVEINHKKITFLDTPGHEA 437

WP_055108420_1 ----DNRS-GNF-------KN--NRG-GK-GGRG-K-GGAQQ--PPREKIDNTPKKIIVRGNMTVGEAAKLLHKDASEVIKKLIMLGVMATINQELDIDTILLLAGDFGV-EVEVKIPVEEDRFETLEENDDEA-DLMERPPVVTIMGHVDHGKTTLLDAIRSTNVSGGEAGGITQHIGAYQVEINNKKITFLDTPGHEA 421

WP_068619253_1 ----DNKP-GGY-------KN--NRG-GK-NNRG-R-GPQQP--ERREKIDNTPKKIIVRGEMTVGETAKLLHKDASEVIKKLISLGVMATINQELDLDTIQLLAGDYGV-EVEIKIHVEDDRFENVEETDDEA-DLKERPPVVTIMGHVDHGKTTLLDAIRSTNVTGGEAGGITQHIGAYQVEINNKKITFLDTPGHEA 416

WP_015844984_1 ----DGKP--NF----RT-NP--NGR-GR-GGRN---NRNHSQQPPREKIDNTPKKIIVRGTMTVGDLAKLLHKDASEVIKKLISLGVMATINQELDMDTILLIAQEFGV-EVEVKIPVEEDTFETVEEVDDEA-DLTTRPPVVTIMGHVDHGKTTLLDAIRHTNVTGGEAGGITQHIGAYQVEINHKKITFLDTPGHEA 507

WP_091181685_1 ----DGKP--NF----RT-NP--NNR-GK-GGRN---NRNHSQQPPREKIDNTPKKIIVRGTMTVGDLAKLLHKDASEVIKKLISLGVMATINQELDMDTILLIASEFGV-EVEVKIPVEEDTFETVEEVDDEA-DLSTRPPVVTIMGHVDHGKTTLLDAIRHTNVTGGEAGGITQHIGAYQVEINHKKITFLDTPGHEA 506

WP_044877552_1 ----DNKG-GNF-------RN--NRG-GRNNGRG-K-NQYQA--ERREKVDNTPKKIIVRGAMTVGESAKLLHKDASEVIKKLIMMGVMATINQELDLETVLLLAAEFGV-EVEVKIAVEDDRFETVEEIDEEA-DLKSRPPVVTIMGHVDHGKTTLLDAIRSTNVTSGEAGGITQHIGAYQVEINNKKITFLDTPGHEA 429

WP_094155440_1 ---GGGRG-GNN----RG-GN--NRG-GK-FNNR---GKGQPQ-ERREKIDNTPKKIIVRGEMTVGETAKLLHKDASEVIKKLIMLGTMATINQELDMDTILLLAGDFGV-EVEVKIPVEEDRFETVEENDDPA-DLKTRPPVVTIMGHVDHGKTTLLDAIRSTNVTGGEAGGITQHIGAYQVEINGKKITFLDTPGHEA 421

WP_014281121_1 ----GGRG-GNN----RG-GS--NRG-GK-FNNR---GRGQQQ-ERREKIDNTPKKIIVRGEMTVGETAKLLHKDASEVIKKLITLGTMATINQELDMDTILLLAGDFGV-EVEVKIPVEEDRFETVEENDDPD-LLKTRPPVVTIMGHVDHGKTTLLDAIRSTNVTGGEAGGITQHIGAYQVEINSKKITFLDTPGHEA 415

WP_023988217_1 ----GGRG-GNN----RG-GS--NRG-GK-FNNR---GKGQQQ-ERREKIDNTPKKIIVRGEMTVGETAKLLHKDASEVIKKLIVLGTMATINQELDMDTILLLAGDFGV-EVEVKIPVEEDRFETVEENDDPA-DLKTRPPVVTIMGHVDHGKTTLLDAIRSTNVTGGEAGGITQHIGAYQVEINSKKITFLDTPGHEA 414

WP_028542714_1 ----GGRG-GNN----RG-GS--NRG-GK-FNNR---GKGQQQ-ERREKIDNTPKKIIVRGEMTVGETAKLLHKDASEVIKKLIVLGTMATINQELDMDTILLLAGDFGV-EVEVKIPVEEDRFETVEENDDPA-DLKTRPPVVTIMGHVDHGKTTLLDAIRSTNVTGGEAGGITQHIGAYQVEINSKKITFLDTPGHEA 414

WP_071640528_1 ----GGRG-GNN----RG-GS--NRG-GK-FNNR---GKGQQQ-ERREKIDNTPKKIIVRGEMTVGETAKLLHKDASEVIKKLIVLGTMATINQELDMDTILLLAGDFGV-EVEVKIPVEEDRFETVEENDDPA-DLKTRPPVVTIMGHVDHGKTTLLDAIRSTNVTGGEAGGITQHIGAYQVEINSKKITFLDTPGHEA 414

WP_013370722_1 ----GGRG-GNN----RG-GN--NRG-GK-FNNR---GKGQQQ-ERREKIDNTPKKIIVRGEMTVGETAKLLHKDASEVIKKLIMLGTMATINQELDMDTILLLAGDFGV-EVEVKIPVEEDRFETVEENDDPA-DLRTRPPVVTIMGHVDHGKTTLLDAIRSTNVTGGEAGGITQHIGAYQVEINSKKITFLDTPGHEA 405

WP_016820981_1 ----GGRG-GNN----RG-GN--NRG-GK-FNNR---GKGQQQ-ERREKIDNTPKKIIVRGEMTVGETAKLLHKDASEVIKKLIMLGTMATINQELDMDTILLLAGDFGV-EVEVKIPVEEDRFETVEENDDPA-DLRTRPPVVTIMGHVDHGKTTLLDAIRSTNVTGGEAGGITQHIGAYQVEINSKKITFLDTPGHEA 405

WP_068655661_1 ----DNKG-GNF-------RN--NRG-GRNNGRG-K-AQYPV----REKIDNTPKKIIVRGAMTVGESAKLLHKDASEVIKKLITMGVMATINQELDIETVLLLAAEFGV-EVEVKIAVEDDRFETVEEVDDEA-DLKSRPPVVTIMGHVDHGKTTLLDAIRSSNVSSGEAGGITQHIGAYQVENNGKKITFLDTPGHEA 396

WP_060534524_1 NNRGGGGN-RNG----RG-GK--GRG-GR---NQ---NYNQPP---REKIDNTPKKIIVRGEMTVGETAKLLHKDASEVIKKLISSGVMATINQELDLDTIQLLADDYGV-EVEVKIAVEDDRFETVEEVDDEA-DLKARPPVVTIMGHVDHGKTTLLDAIRSTNVTGGEAGGITQHIGAYQVEINKKKITFLDTPGHEA 414

WP_074094581_1 ----DNRG-GNF----RG-----NRG-GK-NNRN---RNQQQY-QQREKIDNTPKKIIVRGDMTVGETAKLLHKDASEVIKKLIAMGVMATINQELDIETILLLSGEFGV-EVEVKIVLEDDRFETLEENDDAA-DLQARPPVVTIMGHVDHGKTTLLDAIRSTNVSDGEAGGITQHIGAYQVEINNKKITFLDTPGHEA 424

WP_062326090_1 ----DNRG-GNF----RG-----NRG-GK-NNRN---RNQQQY-QQREKIDNTPKKIIVRGDMTVGETAKLLHKDASEVIKKLIAMGVMATINQELDIETILLLAGEFGV-EVEVKIVLEDDRFETLEENDDPA-DLQSRPPVVTIMGHVDHGKTTLLDAIRSTNVTGGEAGGITQHIGAYQVEINNKKITFLDTPGHEA 423

WP_015736303_1 -D--NRQ--GGF----R--GN--NRG-GK-NNRG---RNQHNQQPPREKIDNTPKKIIVRGNMTVGETAKLLHKDASEVIKKLIFLGVMATINQELDIDTILLLAGEFGV-EVEVKIPVEEDRFETVEENDAPE-ELKERPPVVTIMGHVDHGKTTLLDAIRSTNVSSGEAGGITQHIGAYQVEINNKKITFLDTPGHEA 456

WP_036642094_1 -D--NNRQ-GGF----R--GN--NRG-GK-NNRG---RNQYNQQPPREKIDNTPKKIIVRGNMTVGETAKLLHKDASEVIKKLIFLGVMATINQELDIDTILLLAGEFGV-EVEVKIPVEEDRFETVEENDTPE-ELKERPPVVTIMGHVDHGKTTLLDAIRSTNVTGGEAGGITQHIGAYQVEINNKKITFLDTPGHEA 451

WP_108465278_1 ----DNRN-GGN-F--R--GN--NRG-GK-GGRG---GKAQPQ-ERREKIDNTPKKIIVRGDMTVGETAKLLHKDASEVIKKLIGMGVMATINQELDIDTILLLAGEFGV-EVEVKIPVEEDRFETVEENDEPA-DLKERPPVVTIMGHVDHGKTTLLDAIRSTNVTGGEAGGITQHIGAYQVEINNKKITFLDTPGHEA 427

WP_036651540_1 ----DNKS-GNF----R--NN--NRG-GR-NNGR---GRGQNQ-PPREKIDNTPKKIIVRGVMTVGETAKLLHKDASEVIKKLISMGVMATINQELDMDTILLLAGEFGV-EVEVKIVLEDDRFETVEESDDDK-DLKSRPPVVTIMGHVDHGKTTLLDAIRSTNVTGGEAGGITQHIGAYQVEINHKKITFLDTPGHEA 462

WP_099479276_1 -D--NNRQ-GGF----R--GN--NRG-GK-NNRG---RNQNNQQPPREKIDNTPKKIIVRGNMTVGETAKLLHKDASEVIKKLIFLGVMATINQELDIDTIQLLAGEFGV-EVEVKIPVEEDRFETVEENDAPE-DLKERPPVVTIMGHVDHGKTTLLDAIRSTNVTGGEAGGITQHIGAYQVEINGKKITFLDTPGHEA 421

WP_076168052_1 -D--NRQG-GGF----N--RN--NRG-GK-NNRG---R-QQSQQPPREKIDNTPKKIIVRGNMTVGETAKLLHKDASEVIKKLIQMGVMATINQELDIDTILLLAGEFGV-EVEVKIVVEDDRFETVEENDDPA-DLKSRPPVVTIMGHVDHGKTTLLDAIRSTNVTGGEAGGITQHIGAYQVEINNKKITFLDTPGHEA 436

WP_038696662_1 ----DGKN-GGF-------RN--NGR-GG-KNQR---GKYQQQTERREKIDNTPKKIIVRGNMTVGETAKLLHKDASEVIKKLILMGVMATINQELDIDTIQLLAGEFGV-EVEVKIPVEEDRFETVEENDAPE-DLKARPPVVTIMGHVDHGKTTLLDAIRSTNVTSGEAGGITQHIGAYQVEINHKKITFLDTPGHEA 443

WP_042207090_1 ----DGRG-GNY----R--NN--GRG-GK-NQRG---GRNQQPMERREKIDNTPKKIIVRGNMTVGETAKLLHKDASEVIKKLILMGVMATINQELDIETIQLLAGEFGV-EVEVKIPVEEDRFETVEENDAPE-DLQARPPVVTIMGHVDHGKTTLLDAIRSTNVTGGEAGGITQHIGAYQVEINHKKITFLDTPGHEA 456

WP_082210233_1:7-883 ----DGRG-GNF----R--NN--GRG-GK-NQRG---GRNQQQVERREKIDNTPKKIIVRGNMTVGETAKLLHKDASEVIKKLILMGVMATINQELDIDTIQLLAGEFGV-EVEVKIPVEEDRFETVEENDAPE-HLQSRPPVVTIMGHVDHGKTTLLDAIRSTNVTGGEAGGITQHIGAYQVEINHKKITFLDTPGHEA 440

WP_025335323_1 ----DGKG-GNF----R--NN--GRG-GK-NQRG---GRNQQPVERREKIDNTPKKIIVRGNMTVGETAKLLHKDASEVIKKLILMGVMATINQELDIETIQLLAGEFGV-EVEVKIPVEEDRFETVEENDAPE-DLRARPPVVTIMGHVDHGKTTLLDAIRSTNVTGGEAGGITQHIGAYQVEINQKKITFLDTPGHEA 438

WP_036656399_1 ----DGKG-GNY----R------GRG-GK-NGRG-K-NQQM---ERREKIDNTPKKIIVRGSMTVGETAKLLHKDASEVIKKLITMGVMATINQELDIDTILLLAGEFGV-EVEVKIPVDEDSFETVEENDMDE-ELITRPPVVTIMGHVDHGKTTLLDAIRSTNVTGGEAGGITQHIGAYQVEINHKKITFLDTPGHEA 483

WP_087920295_1 ----DGKG-GNY----R------GRG-GK-NGRG-K-NQQM---ERREKIDNTPKKIIVRGSMTVGETAKLLHKDASEVIKKLISMGVMATINQELDIDTILLLAGEFGV-EVEVKIPVDEDSFETVEENDTDE-ELQSRPPVVTIMGHVDHGKTTLLDAIRSTNVSGGEAGGITQHIGAYQVEINHKKITFLDTPGHEA 483

WP_042138118_1 FTAMRARGAQVTDMTIIVVAADDGVMPQTVEAINHAKAAGLPIIVAVNKIDKPGADPDKVKQELTSYELVPEEWGGDTIFVNLSAKQRINLEELLEMILLVAEVNEYKANPDKRARGTVIEAELDKNRGPVARILVQNGTLKVGDAFVAGNCFGRVRAMVNDKGRKIKEAGPSTPVEITGLTEVPQAGDPFMAFEDERKA 664

WP_042214813_1 FTAMRARGAQVTDMTIIVVAADDGVMPQTVEAINHAKAAGLPIIVAVNKIDKPGADPDKVKQELTSYELVPEEWGGDTIFVNLSAKQRINLEELLEMILLVAEVNEYKANPDKRARGTVIEAELDKNRGPVARILVQNGTLKVGDAFVAGNCFGRVRAMVNDKGRKIKEAGPSTPVEITGLTEVPQAGDPFMAFEDERKA 664

WP_042237107_1 FTAMRARGAQVTDMTIIVVAADDGVMPQTVEAINHAKAAGLPIIVAVNKIDKPGADPDKVKQELTSYELVPEEWGGDTIFVNLSAKQRINLEELLEMILLVAEVNEYKANPDKRARGTVIEAELDKNRGPVARILVQNGTLKVGDAFVAGNCFGRVRAMVNDKGRKIKEAGPSTPVEITGLTEVPQAGDPFMAFEDERKA 663

WP_039874189_1 FTAMRARGAQVTDMTIIVVAADDGVMPQTVEAINHAKAAGLPIIVAVNKIDKPGADPDRVKQELTNYELVPEEWGGDTIFVNLSAKQRINLEELLEMILLVAEVNEYKANPDKRARGTVIEAELDKNRGPVARILVQNGTLKVGDAFVAGNCFGRVRAMVNDKGRKIKEAGPSTPVEITGLTEVPQAGDPFMAFEDERKA 654

WP_038593413_1 FTAMRARGAQVTDMTIIVVAADDGVMPQTVEAINHAKAAGLPIIVAVNKIDKPGADPDKVKQELTSYELVPEEWGGDTIFVNLSAKQRINLEELLEMILLVAEVNEYKANPDKRARGTVIEAELDKNRGPVARILVQNGTLKVGDAFVAGNCFGRVRAMVNDKGRKIKEAGPSTPVEITGLTEVPQAGDPFMAFEDERKA 653

WP_042177491_1 FTAMRARGAQVTDMTIIVVAADDGVMPQTVEAINHAKAAGLPIIVAVNKIDKPGADPDRVKQELTNYELVPEEWGGDTIFVNLSAKQRINLEELLEMILLVAEVNEYKANPDKRARGTVIEAELDKNRGPVARILVQNGTLKVGDAFVAGNCFGRVRAMVNDKGRKIKEAGPSTPVEITGLTEVPQAGDPFMAFEDERKA 653

WP_036688463_1 FTAMRARGAQVTDMTIIVVAADDGVMPQTVEAIAHAKAAGLPIIVAVNKIDKPGADPDKVKQELTSYELVPEEWGGDTIFVNLSAKQRINLEELLEMILLVAEVNEYKANPDKRARGTVIEAELDKSRGPVARILVQNGTLKVGDAFVAGNCFGRVRAMVNDKGRKIKEAGPSTPVEITGLTEVPQAGDPFMAFEDERKA 649

WP_042266949_1 FTAMRARGAQVTDMTIIVVAADDGVMPQTVEAINHAKAAGLPIIVAVNKIDKPGADPDRVKQELTNYELVPEEWGGDTIFVNLSAKQRINLEELLEMILLVAEVNEYKANPDKRARGTVIEAELDKNRGPVARILVQNGTLKVGDAFVAGNCFGRVRAMVNDKGRKIKEAGPSTPVEITGLTEVPQAGDPFMAFEDERKA 646

WP_042128858_1 FTAMRARGAQVTDMTIIVVAADDGVMPQTVEAIAHAKAAGLPIIVAVNKIDKPGADPDKVKQELTSYELVPEEWGGDTIFVNLSAKQRINLEELLEMILLVAEVNEYKANPDKRARGTVIEAELDKNRGPVARVLVQNGTLKVGDAFVAGNCFGRVRAMVNDKGRKIKEAGPSTPVEITGLTEVPQAGDPFMAFEDERKA 644

WP_042189415_1 FTAMRARGAQVTDMTIIVVAADDGVMPQTVEAIAHAKAAGLPIIVAVNKIDKPGADPDKVKQELTSYELVPEEWGGDTIFVNLSAKQRINLEELLEMILLVAEVNEYKANPDKRARGTVIEAELDKNRGPVARVLVQNGTLKVGDAFVAGNCFGRVRAMVNDKGRKIKEAGPSTPVEITGLTEVPQAGDPFMAFEDERKA 644

WP_046504296_1 FTAMRARGAQVTDMTIIVVAADDGVMPQTVEAINHAKAAGLPIIVAVNKIDKPGADPDRVKQELTNYELVPEEWGGDTIFVNLSAKQRINLEELLEMILLVAEVNEYKANPDKRARGTVIEAELDKNRGPVARILVQNGTLKVGDAFVAGNCFGRVRAMVNDKGRKIKEAGPSTPVEITGLTEVPQAGDPFMAFEDERKA 639

WP_016362856_1 FTSMRARGAQVTDITIIVVAADDGVMPQTVEAIAHAKAAKVPIIVAVNKIDKPDSNPDKIKQELTEYELVPEEWGGDTIFCNISAKQRMGLENLLDMILLVAEVQEFKANPNKRARGTVIEAELDKGKGPVARVLIQHGTLKVGDSFVAGVCFGRVRAMVNDKGKKLKEAGPSTPVEITGLTEVPQAGDPFLAFEDERKA 768

WP_068608410_1 FTSMRARGAQVTDITILVVAADDGVMPQTVEAINHAKAAKVPIIVAINKIDKEGANPDRIKQDLTEYELVPEEWGGDTIFVEVSAKQRLNLEGLLETILLIAEVNEYKANPNKRARGTVIEAELDKGRGAVARVLIQHGTLKVGDAFIAGVYFGRVRTMVNDKGRRLKEAGPSTPIEITGLTDVPLAGDPFIVFEDERKA 714

WP_062408002_1 FTSMRARGAQVTDITIIVVAADDGVMPQTVEAINHAKAAKVPIIVAVNKIDKPDANPDRIKQELTEYELVPEEWGGDTIFCNISAKQRIGLENLLDMILLVAEVQELKANPNKRARGTVIEAELDKGKGPVARILIQHGTLKVGDSFVAGVCFGRVRAMVNDRGRKLKEAGPSTPVEITGLTEVPQAGDPFLVFEDERKA 657

WP_005550539_1 FTLMRARGAQVTDITIIVVAADDGVMPQTVEAISHAKAANCPIIVAVNKVDKPEANVDRVKQELTEYELVPEEWGGDTIFVNVSAKQRTGLEELLEMILLVSEVNEFKANPNKDARGTVLEAELDKGRGPVARVLVQQGTLKVGDPFVAGTCFGRVRAMVNDRGRRLKEAGPSTPIEITGLTDVPQAGDMFVAFEDERKA 716

WP_015254924_1 FTTMRARGAQVTDITVLVVAADDGVMPQTVEAINHAKAANVPIIVAVNKIDKPTANPDRIMQELTEYGLVPEAWGGDTIYVNISAKQRINLEELLEMILLVAEVNDYKANPDKRARGTVIEAELDKGKGPVARVLVQHGTLHVGDAFVAGNCFGRVRAMVNDRGRRLKEAGPSTPVEITGLTEVPQAGDPFMVFEDERKA 632

WP_042233063_1 FTTMRARGAQVTDITIIVVAADDGVMPQTVEAISHAKAAGVPIIVAVNKIDKPEANVEKIKQALTEYELVPEEWGGDTIFCEVSAKQRIGLENLLEMILLVAEVQEYKANPDKRARGTVIEAELDKGRGPVARILVQHGTLKVGDSFVAGVCFGRVRAMVNDKGKRLKEAGPSTPVEITGLTEVPQAGDPLLAFEDERKA 676

WP_040948716_1 FTSMRARGAQVTDITILVVAADDGVMPQTVEAINHAKAAAVPIIVAVNKIDKPGAEPDKIKQELTKYELVPEEWGGDTVFVNISAKQRTNLDELLEMILLVAEVKELRANPDKRARGTVIEAELDKGKGPVARVLIQNGTLKVGDSFIAGVCFGRIRAMVNDRGKKLKEAGPSTPVEITGLSEVPGAGDPFIVYEDERKA 803

WP_087432880_1 FTLMRSRGAQVTDITIIVVAADDGVMPQTVEAISHAKAANCPIIVAVNKIDKPEANPDRVKQELTEYELVPEEWGGDTIFVNVSAKQRMGLDELLEMILLVAEVNEYKANPDKRARGTVIEAELDKGRGPVARILVQHGTLKVGDAFVAGTCFGRVRAMVNDRGRRLKEAGPSTPVEITGLTDVPQAGDPFMAFEDERKA 636

WP_036579329_1 FTTMRARGAQVTDITILVVAADDGVMPQTIEAINHAKAAGVPIIVAVNKIDKEGANPDKIMQALTEYELVPEAWGGETIFVNISAKQRMNLEELLEMILLVAEVNDYKANPNKRARGTVIEAELDKGKGPVARVLVQHGTLKIGDAFVAGNCFGRIRAMVNDKGRRMKEAGPSTPVEITGLTEVPLAGDPFMVFEDERKA 701

WP_068650597_1 FTAMRARGAQVTDITIIVVAADDGVMPQTVEAISHAKAAELPIIVAVNKIDKPGADPDKVKQELTEYGLVPEEWGGDTIFVNVSAKQKTGLEDLLEMILLVAEMNEYKANPDKRARATVLEAELDKGRGPVARVLVQHGTLRVGDAFVAGNCFGRIRAMVNDKGRRLKEAGPSTPVEITGLTEVPQAGDPFMVFEDERKA 630

WP_046230755_1 FTLMRARGAQVTDITIIVVAADDGVMPQTVEAVNHAKAAGVPIIVAVNKIDKPDADADKIKQALTEYELVPEEWGGDTIFVNVSAKQRIGLEELLDMILLVAEVNDFKANPDKRARGTVIEAELDKGKGPVARVLVQHGTLKIGDAFVAGNCFGRIRAMVNDKGRRLKEAGPSTPVEITGLTEVPLAGDPFMVFEDERKA 641

WP_099520650_1 FTLMRARGAQVTDITIIVVAADDGVMPQTVEAVNHAKAAGVPIIVAVNKIDKPDADADKIKQALTEYELVPEEWGGDTIFVNVSAKQRIGLEELLDMILLVAEVNDYKANPDKRARGTVIEAELDKGKGPVARVLVQHGTLKIGDAFVAGNCFGRIRAMVNDKGRRLKEAGPSTPVEITGLTEVPLAGDPFMVFEDERKA 641

WP_036622334_1 FTAMRARGAQLTDITIIVVAADDGVMPQTVEAINHAKAAGLPIIVAVNKIDKPEANPDKVKQELTEYELVPEEWGGDTIFVNVSAKQKMGLEDLLEMILLVAEVNEYKANPDKRARGAVIEAELDRNRGPVARVLVQHGTLKVGDAFVAGNCFGRVRAMVNDRGRRLKEAGPSTPVEITGLTEVPQAGDPFMVFEDERKA 632

WP_082083971_1 FTMMRARGAQVTDITIIVVAADDGVMPQTVEAINHAKAAGVPIIVAVNKIDKPEANPDKIKQELTEYELVPEEWGGDTIFVNVSAKQRLGLEELLEMILLVAEVNDYKANPNKRARGTVLEAELDKGKGPVARILVQHGTLKIGDAFVAGNCFGRVRAMINDKGRRLKEAGPSTPVEITGLTEVPQAGDPFMVFEDERKA 674

WP_079940754_1 FTTMRARGAQVTDITIIVVAADDGVMPQTVEAISHAKAANVPIIVAVNKIDKPTADPGKIKQALTEYELVPEEWGGDTIFVEISAKQRIGLENLLEMILLVAEVQEFKANPNKRARGTVIEAELDKGKGPVARILVQHGTLKVGDSFVAGTCFGRVRAMMNDRGKRLKEAGPSSPVEITGLAEVPQAGDPFMVFEDERKA 582

WP_006036631_1 FTLMRARGAQVTDITIIVVAADDGVMPQTVEAVNHAKAAGVPIIVAVNKIDKEGANPDKIKQELTEYGLVPEEWGGDTIFVNLSAKQRINLEGLLEMILLVAEVNDYKANPNKRARGTVLEAELDKGKGPVARVLVQHGTLKVGDAFIAGNSFGRVRAMVNDKGRRLKEAGPSTPVEITGLTEVPLAGDPFLAFEDERKA 744

WP_089523112_1 FTMMRARGAEVTDITIIVVAADDGVMPQTVEAISHAKAAKVPIIVAVNKIDKPDANPDTIKQELTKYELVPEEWGGDTIFVNVSAKQRTNLEELLEMILLVAEVNDYKANPNKRARGTVLEAELDKGKGPIARVLVQHGTLKIGDAFVAGNTFGRIRAMINDKGRRLKEADPSTPVEITGLTEVPKAGDPFMVFEDERKA 739

WP_016312402_1 FTAMRARGAQLTDITIIVVAADDGVMPQTVEAVNHAKAAGLPIIVAVNKIDKPEANPDRVKQELTEYELVPEEWGGDTIFVNVSAKQKIGLENLLEMILLVAEVNEYKANPDKRARGAVIEAELDKTRGPVARVLVQHGTLKVGDAFVAGNCFGRVRAMVNDRGRRLKEAGPSTPVEITGLTEVPQAGDPFMVFEDERKA 616

WP_037282270_1 FTAMRARGAQVTDMTIIVVAADDGVMPQTVEAVNHAKAANLPIIVAVNKIDKPGSDPDRVKQELTEYGLVPEEWGGDTIFVNVSAKQRMGLEGLLEMILLVAEVNDYRANPDKRARGTVIEAELDKGRGPVARILVQHGTLKVGDAFVAGNCFGRVRAMVNDKGRRLKEAGPSTPVEITGLTEVPQAGDPFMVFEDERKA 619

WP_068694645_1 FTAMRARGAQVTDMTIIVVAADDGVMPQTVEAINHAKAAGLPIIVAVNKIDKPGANPDKVKQELTEYELVPEEWGGDTIFVNVSAKQKLGIEELLEMILLVAEVNEYKANPNKRARGTVIEAELDKGRGPVARVLVQHGTLKIGDAFVAGNCFGRVRAMVNDKGRRLKEAGPSTPVEITGLTEVPGAGDPFMVFEDERKA 637

WP_055108420_1 FTAMRARGAQLTDITIIVVAADDGVMPQTVEAVNHAKAAGLPIIVAVNKIDKPTANPDKVKQELTEYGLVPEEWGGDTIFVNVSAKQRMGLEDLLEMILLVAEVNEYKANPDKRARGAVIEAELDKSRGPVARVLVQHGTLKVGDAFVAGNCFGRIRAMVNDKGRRIKEAGPSTPVEITGLTEVPLAGDPFMVFEDERKA 621

WP_068619253_1 FTTMRARGAQVTDITIIVVAADDGVMPQTVEAISHAKAAGVPIIVAVNKIDKPEADPDRVKQELTNYELVPEEWGGDTIFVNVSAKQRIGLEGLLEMILLVAEVNEYKANPDKRARGTVIEAELDKGRGPVARVLVQHGTLKVGDAFVAGNYFGRVRAMVNDKGRRLKEAGPSTPVEITGLTEVPLAGDPLMVFEDERKA 616

WP_015844984_1 FTLMRARGAQVTDITIIVVAADDGVMPQTVEAVNHAKAAGVPIIVAVNKIDKPDADPDKIKQALTEYELVPEEWGGDTIFVNVSAKQRLGLEELLEMILLVAEVNDYKANADKRARGTVIEAELDKGKGPVARVLVQHGSLKIGDAFVAGNCFGRVRAMVNDKGRRIKEAGPSTPVEITGLTEVPLAGDPFMVFEDERKA 707

WP_091181685_1 FTLMRARGAQVTDITIIVVAADDGVMPQTVEAVNHAKAAGVPIIVAVNKIDKPDADPDKIKQALTEYELVPEEWGGDTIFVNVSAKQRLGLEELLEMILLVAEVNDYKANSDKRARGTVIEAELDKGKGPVARVLVQHGSLKIGDAFVAGNCFGRVRAMVNDKGRRIKEAGPSTPVEITGLTEVPLAGDPFMVFEDERKA 706

WP_044877552_1 FTAMRARGSQITDIAIIVVAADDGVMPQTVEAISHAKAAELPIIVAVNKIDKPGADADKVKQELTEHGLVPEEWGGDTIFVNVSAKQRMGLEELLEMILLVAEMNEYKANPDKRARGAVIEAELDKGRGPVARVLVQHGTLRVGDAFVAGNCFGRIRAMVNDKGRRLKEAGPSTPVEITGLTEVPQAGDPFMVFEDERKA 629

WP_094155440_1 FTAMRARGAQVTDMTIIVVAADDGVMPQTVEAINHAKAAGLPIIVAVNKIDKPDANPDKVKQELTEYELVPEEWGGDTIFVNVSAKQRMGLEDLLEMILLVAEVNEYKANPDKRARGTIIEAELDKGRGSVARVLVQNGTLKVGDAFVAGNCFGRVRAMVNDKGRRLKEAGPSTPVEITGLTEVPLAGDPFMVFEDERKA 621

WP_014281121_1 FTAMRARGAQVTDMTIIVVAADDGVMPQTVEAINHAKAAGLPIIVAVNKIDKPDANPDKVKQELTEYELVPEEWGGDTIFVNVSAKQRMGLEDLLEMILLVAEVNEYKANPDKRARGTIIEAELDKGRGSVARILVQNGTLKVGDAFVAGNCFGRVRAMVNDKGRRLKEAGPSTPVEITGLTEVPLAGDPFMVFEDERKA 615

WP_023988217_1 FTAMRARGAQVTDMTIIVVAADDGVMPQTVEAINHAKAAGLPIIVAVNKIDKPDANPDKVKQELTEYELVPEEWGGDTIFVNVSAKQRMGLEDLLEMILLVAEVNEYKANPNKRARGTIIEAELDKGRGSVARILVQNGTLKVGDAFVAGNCFGRVRAMVNDKGRRLKEAGPSTPVEITGLTEVPLAGDPFMVFEDERKA 614

WP_028542714_1 FTAMRARGAQVTDMTIIVVAADDGVMPQTVEAINHAKAAGLPIIVAVNKIDKPDANPDKVKQELTEYELVPEEWGGDTIFVNVSAKQRMGLEDLLEMILLVAEVNEYKANPNKRARGTIIEAELDKGRGSVARILVQNGTLKVGDAFVAGNCFGRVRAMVNDKGRRLKEAGPSTPVEITGLTEVPLAGDPFMVFEDERKA 614

WP_071640528_1 FTAMRARGAQVTDMTIIVVAADDGVMPQTVEAINHAKAAGLPIIVAVNKIDKPDANPDKVKQELTEYELVPEEWGGDTIFVNVSAKQRMGLEDLLEMILLVAEVNEYKANPNKRARGTIIEAELDKGRGSVARILVQNGTLKVGDAFVAGNCFGRVRAMVNDKGRRLKEAGPSTPVEITGLTEVPLAGDPFMVFEDERKA 614

WP_013370722_1 FTAMRARGAQVTDMTIIVVAADDGVMPQTVEAISHAKAAGLPIIVAVNKIDKPDANPDKVKQELTEYELVPEEWGGDTIFVNVSAKQRMGLEDLLEMILLVAEVNEYKANPDKRARGTIIEAELDKGRGSVARILVQNGTLKVGDAFVAGNCFGRVRAMVNDKGRRLKEAGPSTPVEITGLTEVPLAGDPFMVFEDERKA 605

WP_016820981_1 FTAMRARGAQVTDMTIIVVAADDGVMPQTVEAISHAKAAGLPIIVAVNKIDKPDANPDKVKQELTEYELVPEEWGGDTIFVNVSAKQRMGLEDLLEMILLVAEVNEYKANPDKRARGTIIEAELDKGRGSVARILVQNGTLKVGDAFVAGNCFGRVRAMVNDKGRRLKEAGPSTPVEITGLTEVPLAGDPFMVFEDERKA 605

WP_068655661_1 FTAMRARGSQITDIAIIVVAADDGVMPQTVEAINHAKAAELPIIVAVNKIDKPGSDPDKVKQELTEYGLVPEEWGGDTIFVNVSAKQKMGLEELLEMILLVAEMNEYKANPDKRARAAVIEAELDKGRGPVARVLVQHGTLRVGDAFVAGNCFGRIRAMVNDKGRRVKEAGPSTPIEITGLTEVPQAGDPFMVFEDERKA 596

WP_060534524_1 FTAMRARGAQVTDITIIVVAADDGVMPQTVEAINHAKAAGLPIIVAVNKIDKPGANPDKVKQELTEYELVPEEWGGDTIFVNLSAKQRTGLEELLEMILLIAEVNDYKANPDKRARGAVIEAELDKGRGPVARILVQNGTLKVGDAFVAGNCFGRIRAMVNDKGRRLKEAGPSTPVEITGLTEVPQAGDPFMVFEDERKA 614

WP_074094581_1 FTAMRARGAQVTDMTIIVVAADDGVMPQTIEAINHAKAAGLPIIVAVNKIDKPGADPDKVKQELTSYELVPEEWGGDTIFVNVSAKQRMGLEGLLEMILLVAEVNEYKANPDKRARGTVIEAELDKGRGPVARILVQHGTLKVGDAFVAGNCFGRVRAMVNDKGRKLKEAGPSTPVEITGLTEVPGAGDPFMVFEDERKA 624

WP_062326090_1 FTAMRARGAQVTDITIIVVAADDGVMPQTVEAINHAKAAGLPIIVAVNKIDKPGADPDKVKQELTNYELVPEEWGGDTIFVNVSAKQRMGLEGLLEMILLVAEVNEYKANPDKRARGTVIEAELDKGRGPVARILVQHGTLKVGDAFVAGNCFGRVRAMVNDKGRRLKEAGPSTPVEITGLTEVPGAGDPFMVFEDERKA 623

WP_015736303_1 FTAMRARGAQVTDMTIIVVAADDGVMPQTVEAINHAKAAGLPIIVAVNKIDKPDANPDKVKQELTEYELVPEEWGGDTIFVNVSAKQRMGLEDLLEMILLVAEVNEYKANPDKRARGTVIEAELDKGRGPVARILVQHGTLKVGDAFVAGNCFGRIRAMVNDKGRRLKEAGPSTPVEITGLTEVPGAGDPFMVFEDERKA 656

WP_036642094_1 FTAMRARGAQVTDMTIIVVAADDGVMPQTVEAINHAKAAGLPIIVAVNKIDKPDANPDKVKQELTEYELVPEEWGGDTIFVNVSAKQRMGMEDLLEMILLVAEVNEYKANPDKRARGTVIEAELDKGRGPVARILVQHGTLKVGDAFVAGNCFGRIRAMVNDKGRRLKEAGPSTPVEITGLTEVPGAGDPFMVFEDERKA 651

WP_108465278_1 FTAMRARGAQITDITIIVVAADDGVMPQTVEAINHAKAAGLPIIVAVNKIDKPGANPDKVKQELTEYELVPEEWGGDTIFVNVSAKQRMGLEDLLEMILLVAEVNDYKANPDKRARGTVIEAELDKGRGPVARVLVQHGTLKVGDAFVAGNCFGRVRAMVNDKGRRLKEAGPSTPVEITGLTEVPGAGDPFMVFEDERKA 627

WP_036651540_1 FTAMRARGAQVTDITIIVVAADDGVMPQTVEAINHAKAAELPIIVAVNKIDKPDANPDKVKQELTEYGLVPEEWGGDTIFVNVSAKQRMGLEELLEMILLVAEMNEYKANPDKRARATVIEAELDKGRGPVARILVQHGTLKVGDAFVAGNCFGRIRAMVSDKGRRLKEAGPSTPVEITGLTEVPQAGDPFMVFEDERKA 662

WP_099479276_1 FTAMRARGAQVTDMTIIVVAADDGVMPQTVEAINHAKAAGLPIIVAVNKIDKPTANPDKVKQELTEYELVPEEWGGDTIFVNVSAKQRMGLEDLLEMILLVAEVNEYKANPDKRARGTVIEAELDKGRGPVARILVQHGTLKVGDAFVAGNCFGRIRAMVNDKGRRLKEAGPSTPVEITGLTEVPGAGDPFMVFEDERKA 621

WP_076168052_1 FTAMRARGAQITDITIIVVAADDGVMPQTVEAINHAKAAELPIIVAVNKIDKPDANPDKVKQELTEYGLVPEEWGGDTIFVNVSAKQRMGLEDLLEMILLVAEVNEYKANPDKRARGAVIEAELDKGRGPVARVLVQHGTLKVGDAFVAGNCFGRVRAMVNDKGRRLKEAGPSTPVEITGLTEVPLAGDPFMVFEDERKA 636

WP_038696662_1 FTAMRARGAQVTDMTIIVVAADDGVMPQTVEAINHAKAAGLPIIVAVNKIDKPGADPDRVKQELTNYELVPEEWGGDTIFVNVSAKQRIGLEDLLEMILLVAEVNEYKANPDKRARGTVIEAELDKSRGPVARILVQNGTLKVGDAFVAGNCFGRVRVMVNDKGRRLKEAGPSTPIEITGLTEVPQAGDPFMVFEDERKA 643

WP_042207090_1 FTAMRARGAQVTDITIIVVAADDGVMPQTVEAINHAKAAGLPIIVAVNKIDKPGADPDRVKQELTNYELVPEEWGGDTIFVNVSAKQRIGLEDLLEMILLVAEVNEYKANPDKRARGTVIEAELDKSRGPVARILVQNGTLKVGDAFVAGNCFGRVRVMVSDKGRRLKEAGPSTPIEITGLTEVPQAGDPFMVFEDERKA 656

WP_082210233_1:7-883 FTAMRARGAQVTDITIIVVAADDGVMPQTVEAINHAKAAGLPIIVAVNKIDKPGADPDRVKQELTNYELVPEEWGGDTIFVNVSAKQRIGLEDLLEMILLVAEVNEYKANPDKRARATVIEAELDKSRGPVARILVQNGTLKVGDAFVAGNCFGRVRVMVNDKGRRLKEAGPSTPIEITGLTEVPQAGDPFMVFEDERKA 640

WP_025335323_1 FTAMRARGAQVTDITIIVVAADDGVMPQTVEAINHAKAAGLPIIVAVNKIDKPGADPDRVKQELTNYELVPEEWGGDTIFVNVSAKQRIGLEDLLEMILLVAEVNEYKANPDKRARGTVIEAELDKSRGPVARILVQNGTLKVGDAFVAGNCFGRVRVMVNDKGRRLKEAGPSTPIEITGLTEVPQAGDPFIVFEDERKA 638

WP_036656399_1 FTAMRARGAQVTDMTIIVVAADDGVMPQTVEAISHAKAAGLPIIVAVNKIDKPGADPDKVKQELTKYELVPEEWGGDTIFVNLSAKQRTGLEDLLEMILLVAEVNEYKANPDKRARGTVIEAELDKNRGPVARILVQHGTLRVGDAFVAGNCFGRVRAMVNDKGRKIKEAGPSTPVEITGLTEVPQAGDPFMAFEDERKA 683

WP_087920295_1 FTAMRARGAQVTDMTIIVVAADDGVMPQTVEAINHAKAAGLPIIVAVNKIDKEGADPDKVKQELTGYELVPEEWGGDTIFVNLSAKQRINLEELLEMILLVAEVNEYKANPDKRARGTVIEAELDKSRGPVARILVQHGTLKVGDAFVAGNCFGRVRAMVNDKGRRLKEAGPSTPVEITGLTEVPQAGDPFMAFEDERKA 683

WP_042138118_1 RAIADRRSTTQRQSELNTNTRVTLDDLFKHIKDGEIKDLNVIIKGDVQGSVEALKGSLAKIEVEGVRVKILHSGAGAITESDITLAAASNAIVIGFNVRPDTQTKAAAEQEKVDVRLHNIIYNVIEEIESAMKGMLDPIYKENVIGHAEVRNVFKISKVGTIAGCMVTSGKIARNAEMRLIRSGIVVFTGKIDTLKRFKD 864

WP_042214813_1 RAIADRRSTSQRQSELNTNTRVTLDDLFKHIKDGEIKDLNVIIKGDVQGSVEALKGSLAKIEVEGVRVKILHSGAGAITESDITLAAASNAIVIGFNVRPDAQTKAAAEQEKVDVRLHNIIYNVIEEIESAMKGMLDPIYKENVIGHAEVRNVFKISKVGTIAGCMVTSGKIARNAEMRLIRSGIVVFTGKIDTLKRFKD 864

WP_042237107_1 RAIADRRSTTQRQSELNTNTRVTLDDLFKHIKDGEIKDLNVIIKGDVQGSVEALKGSLAKIEVEGVRVKILHSGAGAITESDITLAAASNAIVIGFNVRPDTQTKAAAEQEKVDVRLHNIIYNVIEEIESAMKGMLDPIYKENVIGHAEVRNVFKISKVGTIAGCMVTSGKIARNAEMRLIRSGIVVFTGKIDTLKRFKD 863

WP_039874189_1 RAIADRRSITQRQSELNTNTRVTLDDLFKHIKDGEIKDLNVIIKADVQGSVEALKGSLAKIEVEGVRVKIIHSGAGAITESDITLAAASNAIVIGFNVRPDAQTKAAAEQEKVDVRLHNIIYNVIEEIESAMKGMLDPVFKENIIGHAEVRNVFKISKVGSVAGCMVTDGKITRNAEMRLVRGGIVVFEGKIDTLKRFKD 854

WP_038593413_1 RAIADRRSTTQRQSELNTNTRVTLDDLFKHIKDGEIKDLNVIIKADVQGSVEALKGSLAKIEVEGVRVKIIHSGAGAITESDITLAAASNAIVIGFNVRPDAQTKAAAEQEKVDVRLHNIIYNVIEEIESAMKGMLDPIYKENVIGHAEVRNVFKISKVGTIAGCMVTSGKIARNAEMRLIRSGIVVFQGKIDTLKRFKD 853

WP_042177491_1 RAIADRRSITQRQSELNTNTRVTLDDLFKHIKDGEIKDLNVIIKADVQGSVEALKGSLAKIEVEGVRVKIIHSGAGAITESDITLAAASNAIVIGFNVRPDTQTKAAAEQEKVDVRLHNIIYNVIEEIESAMKGMLDPIFKENIIGHAEVRNVFKISKVGSVAGCMVTDGKITRNAEMRLVRGGIVVFEGKVDTLKRFKD 853

WP_036688463_1 RAIADRRSTSQRQSELNTNTRVTLDDLFQHIKDGEIKDLNVIIKADVQGSVEALKGSLAKIEVEGVRVKIIHSGAGAITESDITLAAASNAIVIGFNVRPDAQTKAAAEQEKVDVRLHNIIYNVIEEIESAMKGMLDPIYKENVIGHAEVRSVFKISKVGTIAGCMVTSGKITRNAEMRLIRSGIVVFTGKVDTLKRFKD 849

WP_042266949_1 RAIADRRSTTQRQSELNTNTRVTLDDLFKHIKEGEIKDLNVIIKGDVQGSVEALKGSLAKIEVEGVRVKIIHSGAGAITESDITLAAASNAIVIGFNVRPDAQTKAAAEQEKVDVRLHNIIYNVIEEIESAMKGMLDPVYKENIIGHAEVRNVFKISKVGTIAGCMVTSGKITRNAEMRLIRSGIVVFQGKIDTLKRFKD 846

WP_042128858_1 RAIADRRSTSQRQSELNTNTRVTLDDLFQHIKDGEIKDLNVIIKADVQGSVEALKGSLAKIEVEGVRVKIIHSGAGAITESDITLAAASNAIVIGFNVRPDAQTKAAAEQEKVDVRLHNIIYNVIEEIESAMKGMLDPIYKENVIGHAEVRSVFKISKVGTIAGCMVIDGKITRNAEMRLIRSGIVVFTGKVDTLKRFKD 844

WP_042189415_1 RAIADRRSTSQRQSELNTNTRVTLDDLFQHIKDGEIKDLNVIIKADVQGSVEALKGSLAKIEVEGVRVKIIHSGAGAITESDITLAAASNAIVIGFNVRPDAQTKAAAEQEKVDVRLHNIIYNVIEEIESAMKGMLDPIYKENVIGHAEVRSVFKISKVGTIAGCMVIDGKITRNAEMRLIRSGIVVFTGKVDTLKRFKD 844

WP_046504296_1 RAIADRRSTSQRQSELNTNTRVTLDDLFKHIKEGEIKDLNVIIKGDVQGSVEALKGSLAKIEVEGVRVKIIHSGAGAITESDITLAAASNAIVIGFNVRPDAQTKAAAEQEKVDVRLHNIIYNVIEEIESAMKGMLDPVYKENIIGHAEVRNVFKISKVGTIAGCMVTSGKITRNAEMRLIRSGIVVFQGKIDTLKRFKD 839

WP_016362856_1 RDIAEKRAVTLRQSEITANSRVTLDDLYKQIKEGEIKDLNVIIKSDVQGSAEALKGSLEKIDVEGARVKILLSGVGAITESDVILASASNAIVIGFNVRPEPQAMNTAEQEKVDIRLHSIIYKVMEEIESAMKGLLDPTYKEVVIGQAEVRNVFKVSKVGTIAGCMVTSGKISRNAQGRLIRAGIVVYDGKIDNLKRFKD 968
[truncated: 560,474 more chars]
