# Supplementary material for: Analysis of lineage-specific protein family variability in prokaryotes combined with evolutionary reconstructions
Source: Biol Direct. 2022 Aug 30;17:22. doi: 10.1186/s13062-022-00337-7 (PMC9425974; doi:10.1186/s13062-022-00337-7)
Supplement: Supplementary file 8 — Additional file 8: Table S3. Number of local COGs, broken down by ancestrality, paralogy and variability. [file 13062_2022_337_MOESM8_ESM.docx]

Number of local COGs, broken down by ancestrality, paralogy and variability

| A | v<2 | v>=2 |
| --- | --- | --- |
| p<1.25 | 14715 | 761 |
| p>=1.25 | 3286 | 1737 |
|  |  |  |
| non-A | v<2 | v>=2 |
| p<1.25 | 6357 | 860 |
| p>=1.25 | 1520 | 872 |
