# Supplementary material for: Analysis of lineage-specific protein family variability in prokaryotes combined with evolutionary reconstructions
Source: Biol Direct. 2022 Aug 30;17:22. doi: 10.1186/s13062-022-00337-7 (PMC9425974; doi:10.1186/s13062-022-00337-7)
Supplement: Supplementary file 10 — Additional file 10: Table S5. Correlation between the fraction of microsatellite regions (MSR) and low complexity regions (LCR) across csCOGs. [file 13062_2022_337_MOESM10_ESM.docx]

| **Clade** | **no. of local COGs** | **MSR-LCR Spearman correlation** |
| --- | --- | --- |
| Haloferacales | 3487 | 0.55 |
| Sulfolobales | 2402 | 0.25 |
| Thermococcales | 2222 | 0.19 |
| Methanosarcina | 2932 | 0.26 |
| Flavo | 3769 | 0.33 |
| Deinococcales | 3759 | 0.57 |
| Paenbacillaceae | 6670 | 0.45 |
| Rhodococcus | 4867 | 0.43 |
